# Supplementary figures and images for: FER-mediated phosphorylation and PIK3R2 recruitment on IRS4 promotes AKT activation and tumorigenesis in ovarian cancer cells (part 1 of 3)
Source: eLife. 2022 May 12;11:e76183. doi: 10.7554/eLife.76183 (PMC9098222; doi:10.7554/eLife.76183)

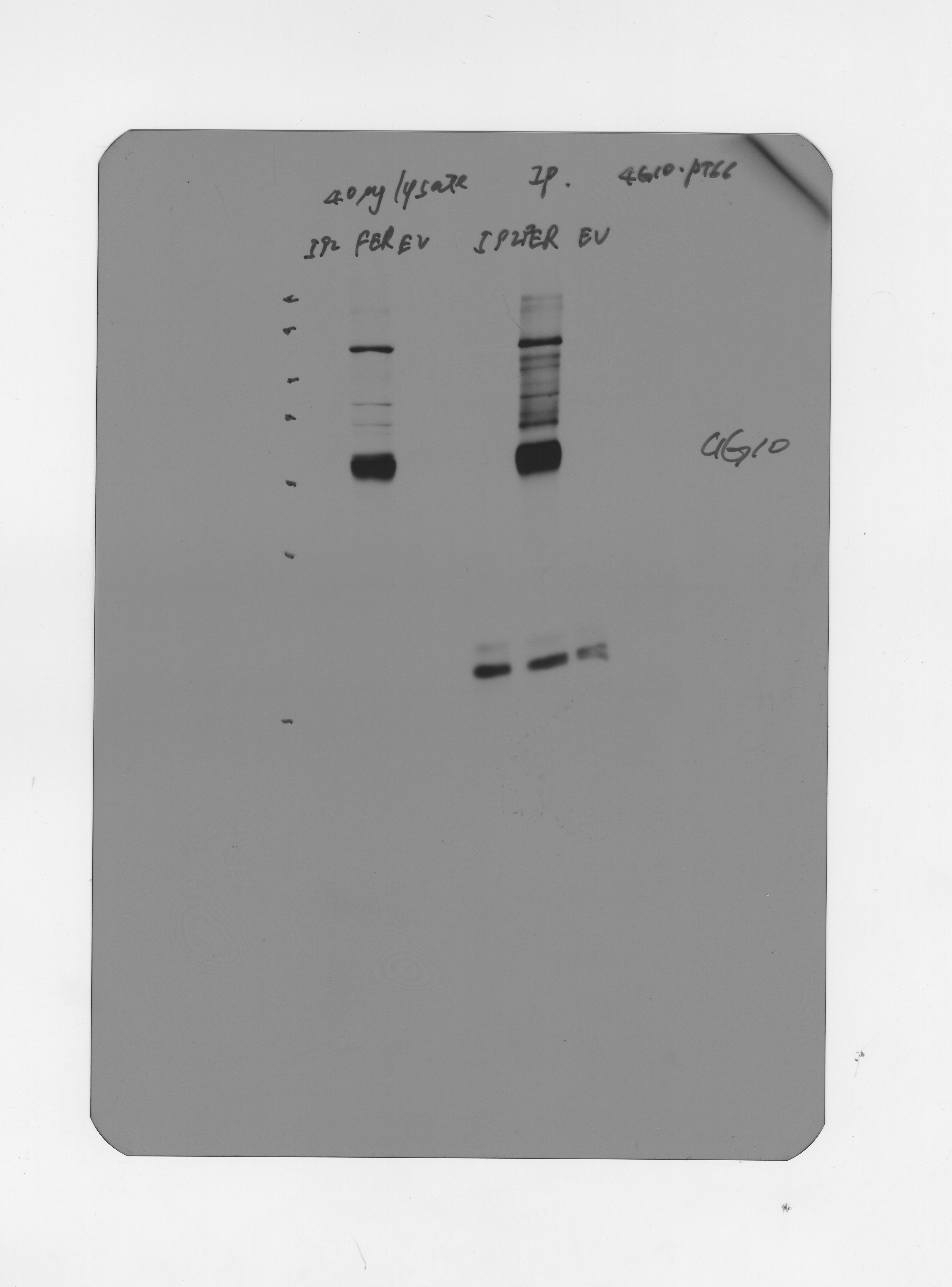

Supplement: Figure 1—source data 1. [file elife-76183-fig1-data1.zip › Figure 1-source data 1/Figure 1A 4G10.jpg]

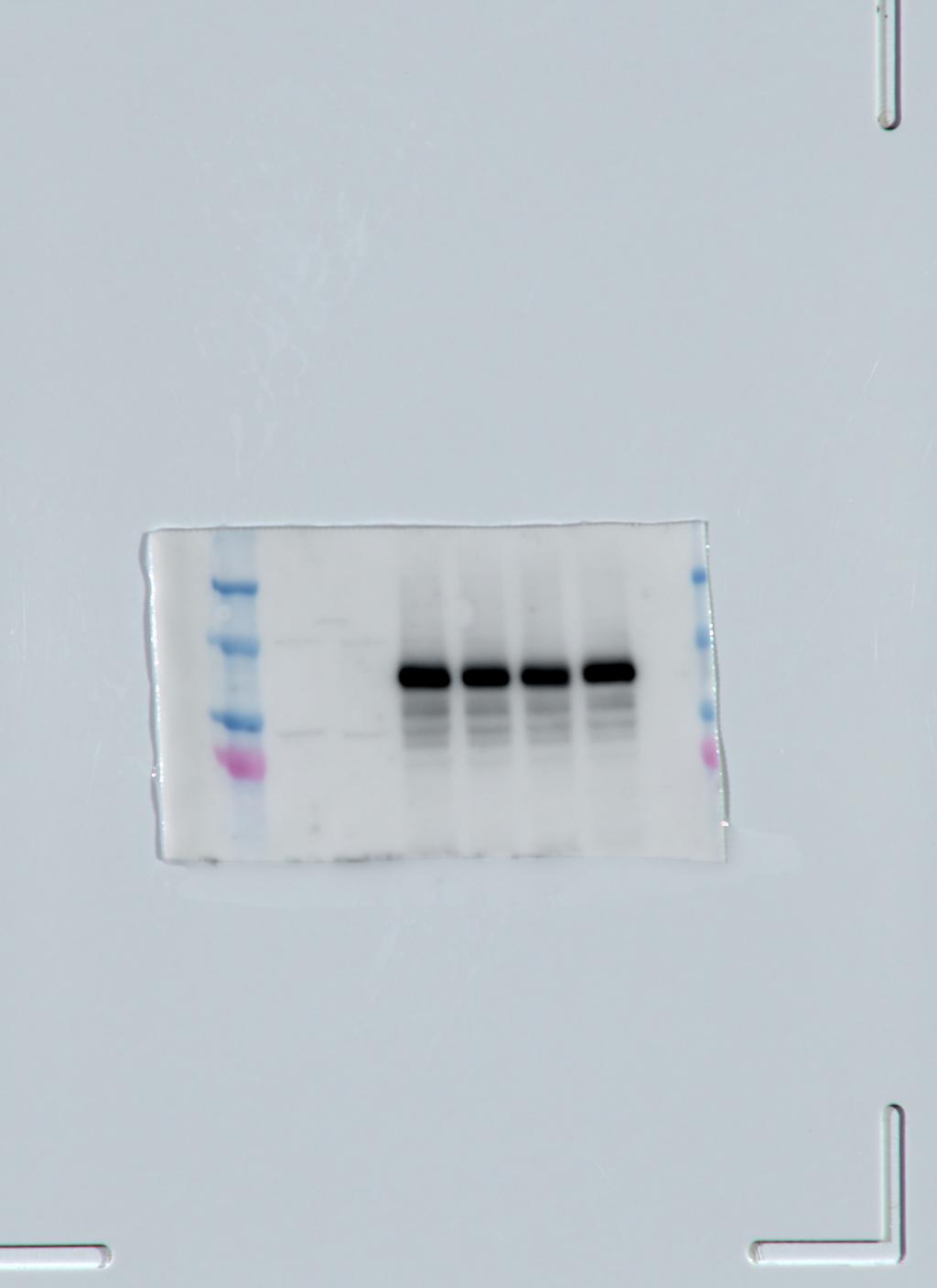

Supplement: Figure 2—source data 1. [file elife-76183-fig2-data1.zip › Figure 2-source data 1/Figure 2A Input WB FER.jpg]

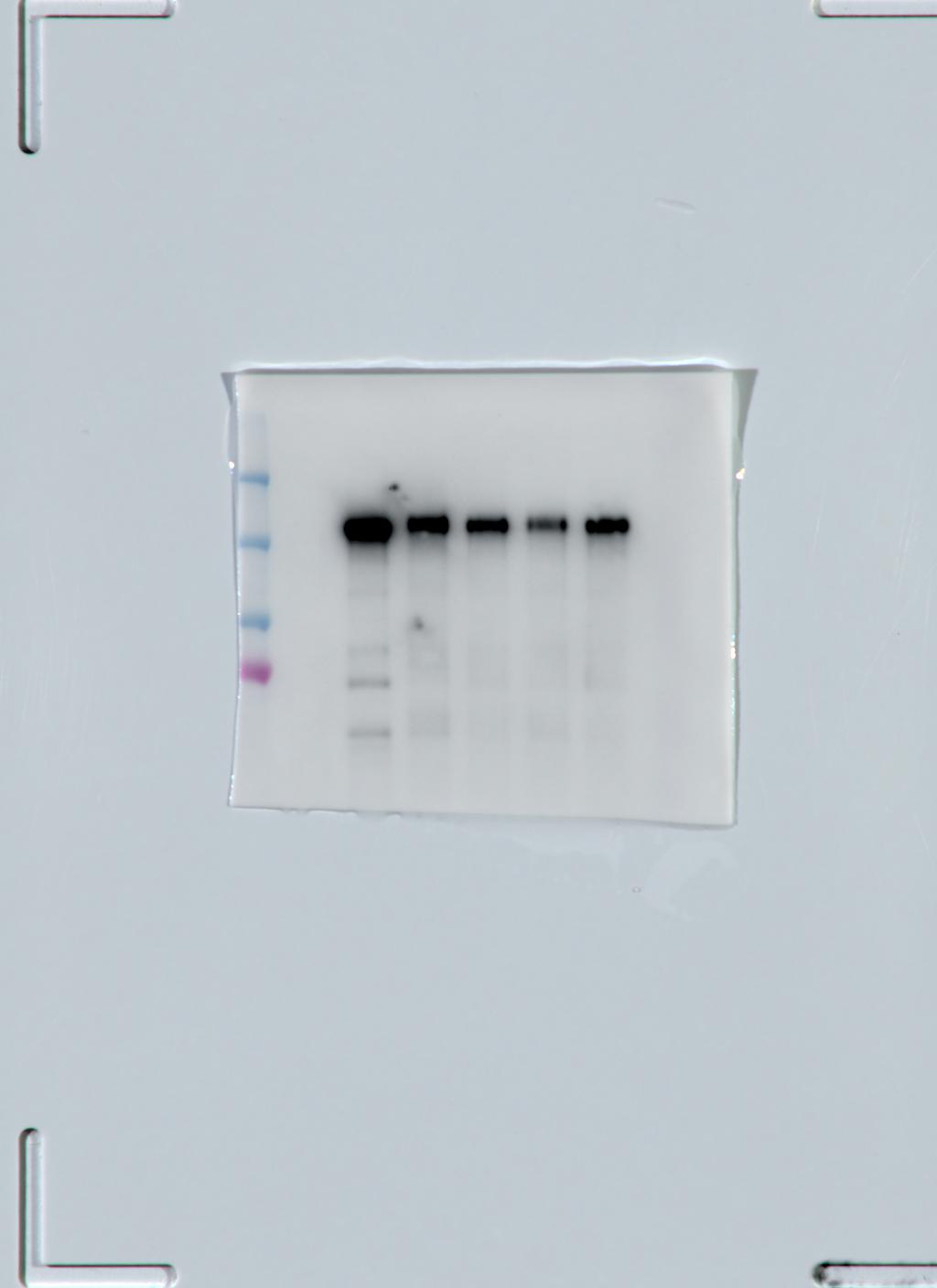

Supplement: Figure 2—source data 1. [file elife-76183-fig2-data1.zip › Figure 2-source data 1/Figure 2A IP Myc WB IRS4.jpg]

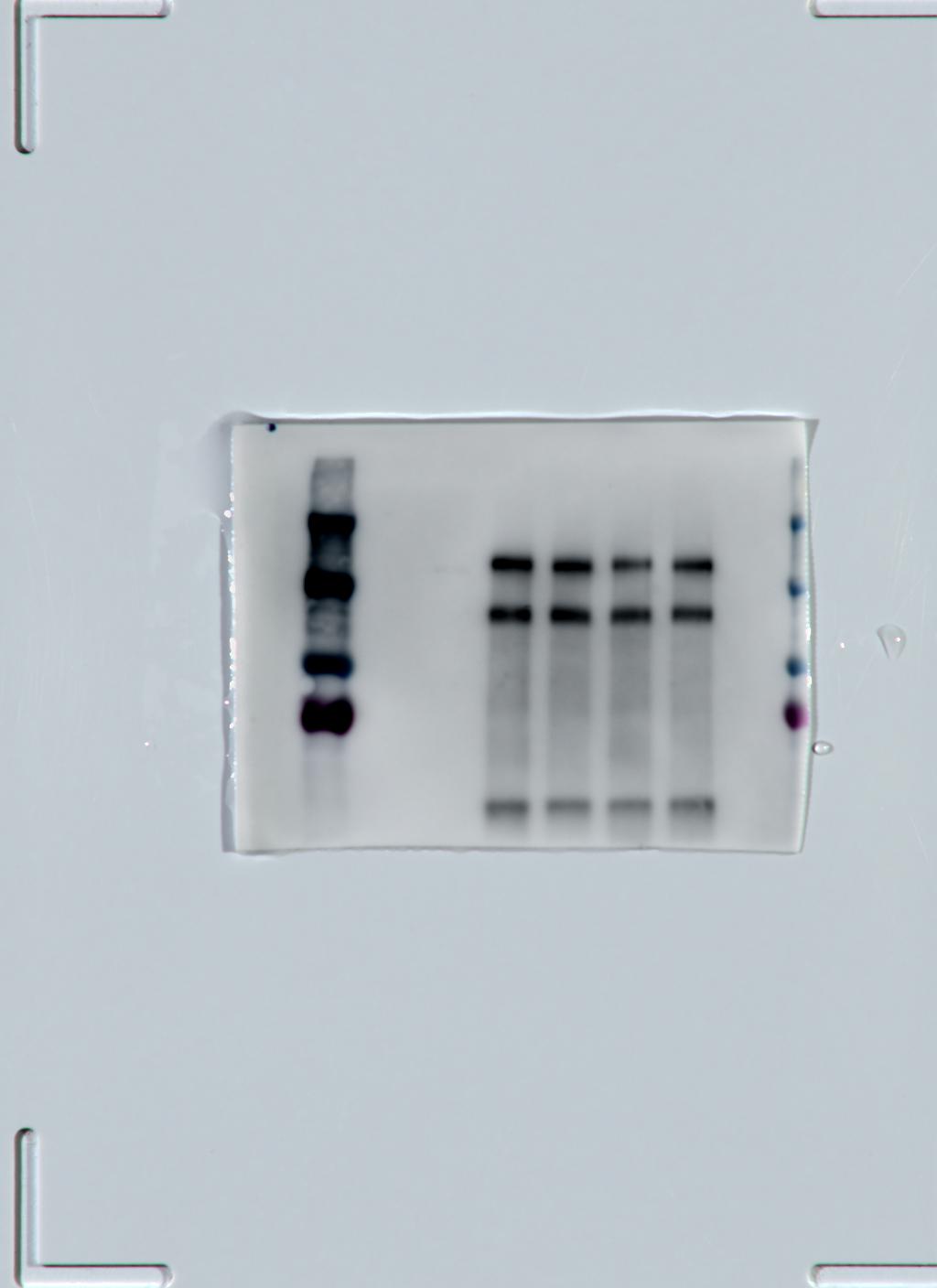

Supplement: Figure 2—source data 1. [file elife-76183-fig2-data1.zip › Figure 2-source data 1/Figure 2A IP Myc WB 4G10.jpg]

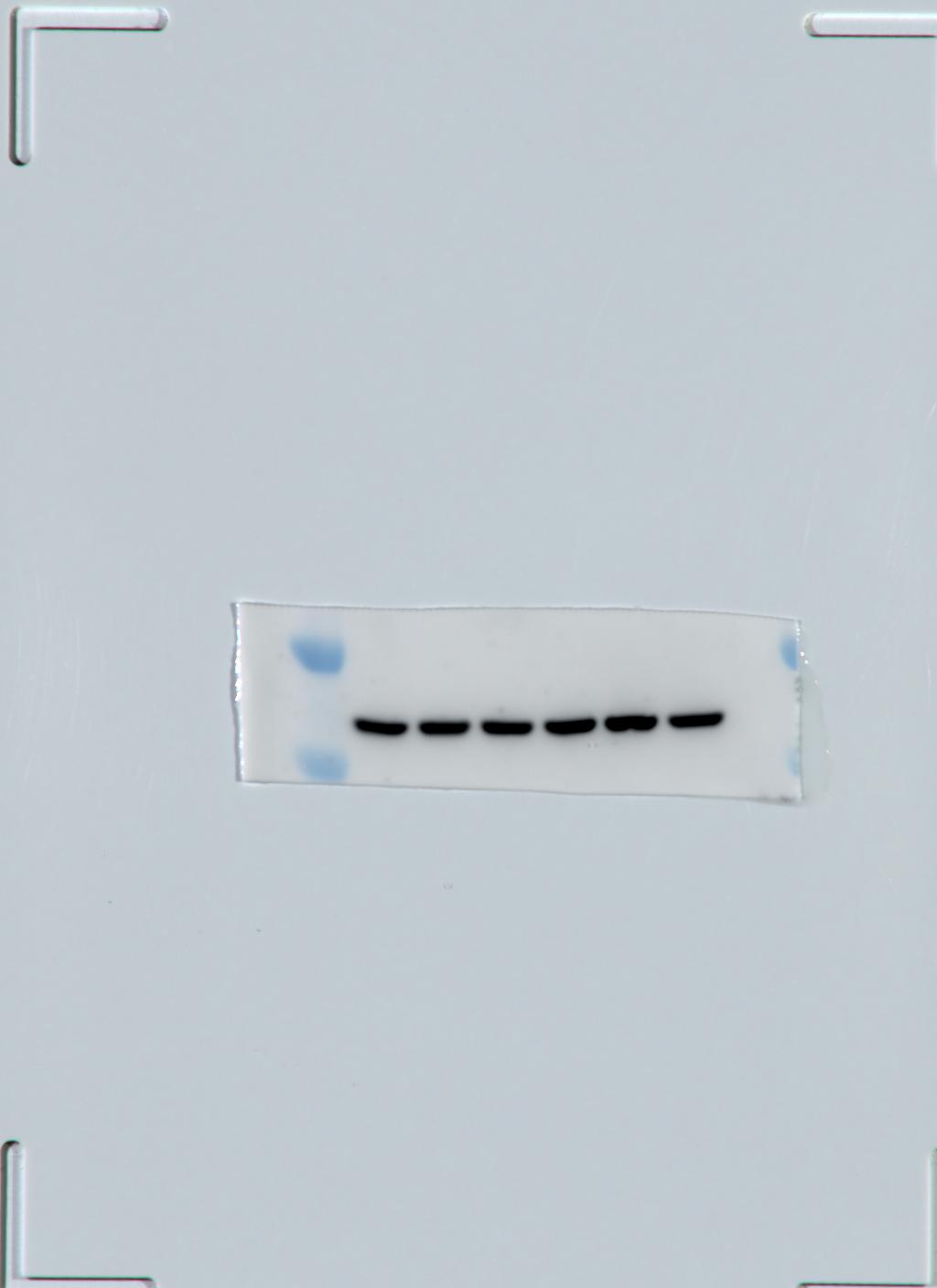

Supplement: Figure 2—source data 1. [file elife-76183-fig2-data1.zip › Figure 2-source data 1/Figure 2A Actin.jpg]

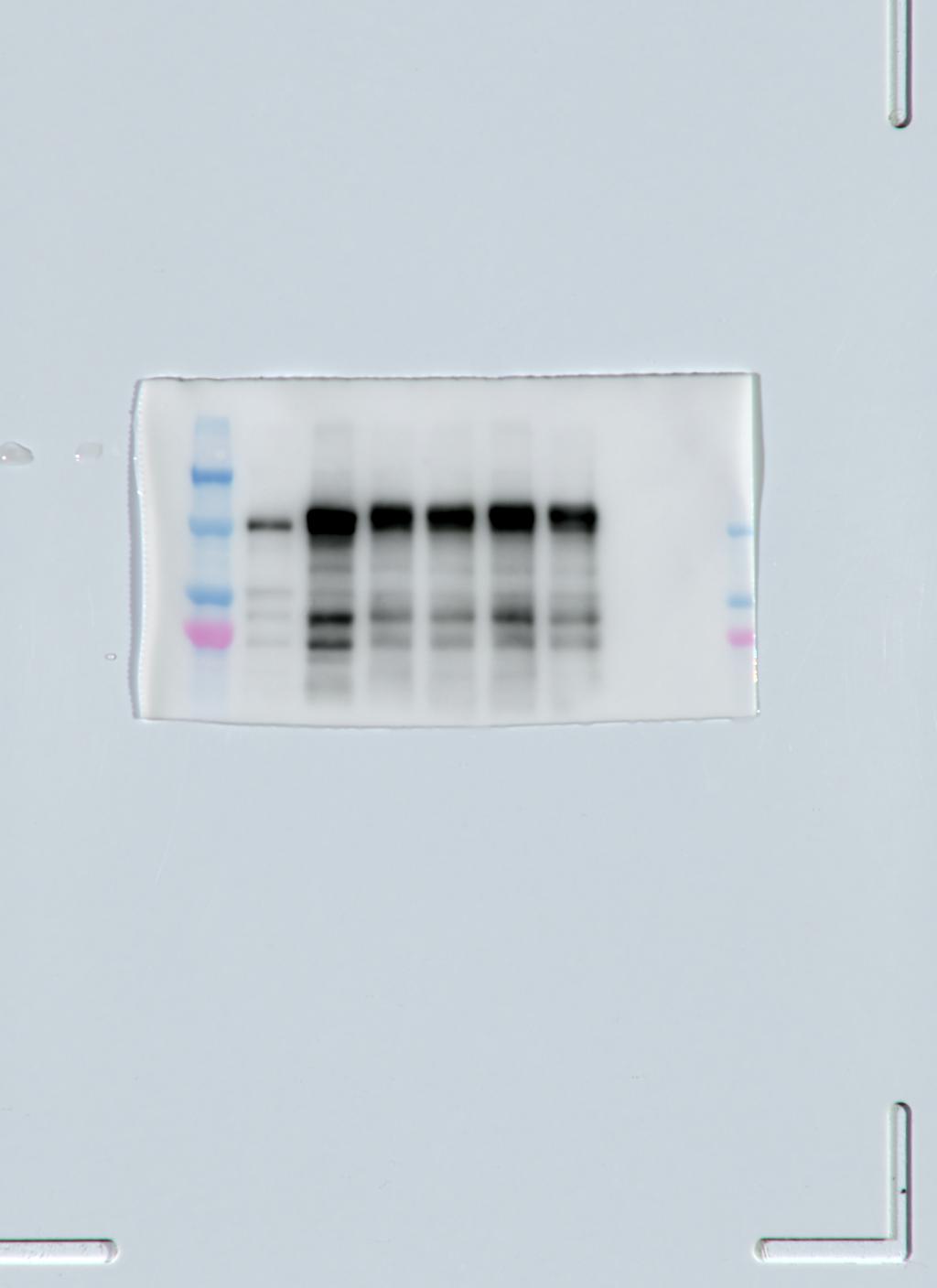

Supplement: Figure 2—source data 1. [file elife-76183-fig2-data1.zip › Figure 2-source data 1/Figure 2A Input WB IRS4.jpg]

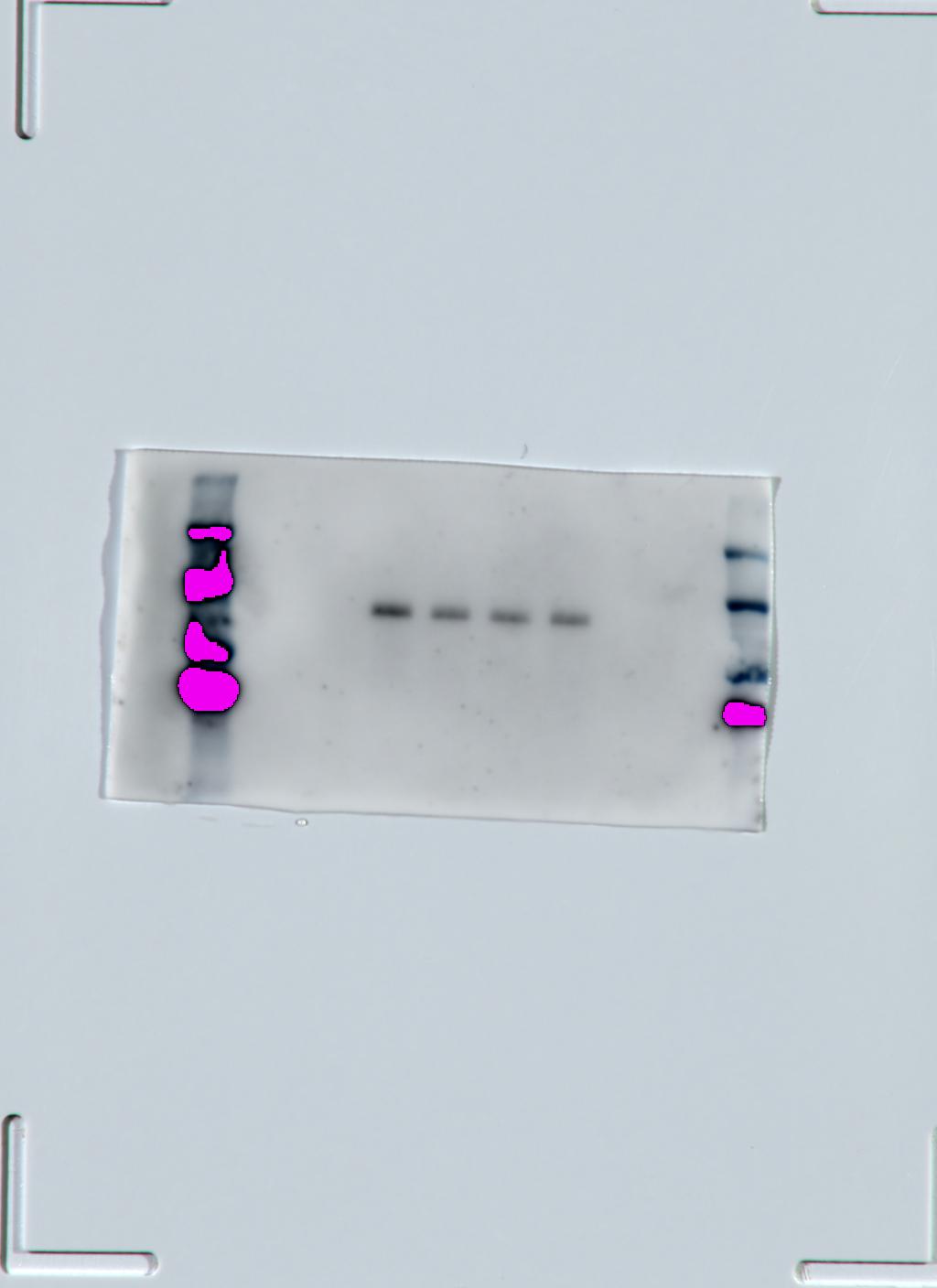

Supplement: Figure 2—source data 1. [file elife-76183-fig2-data1.zip › Figure 2-source data 1/Figure 2A IP Myc WB FER.jpg]

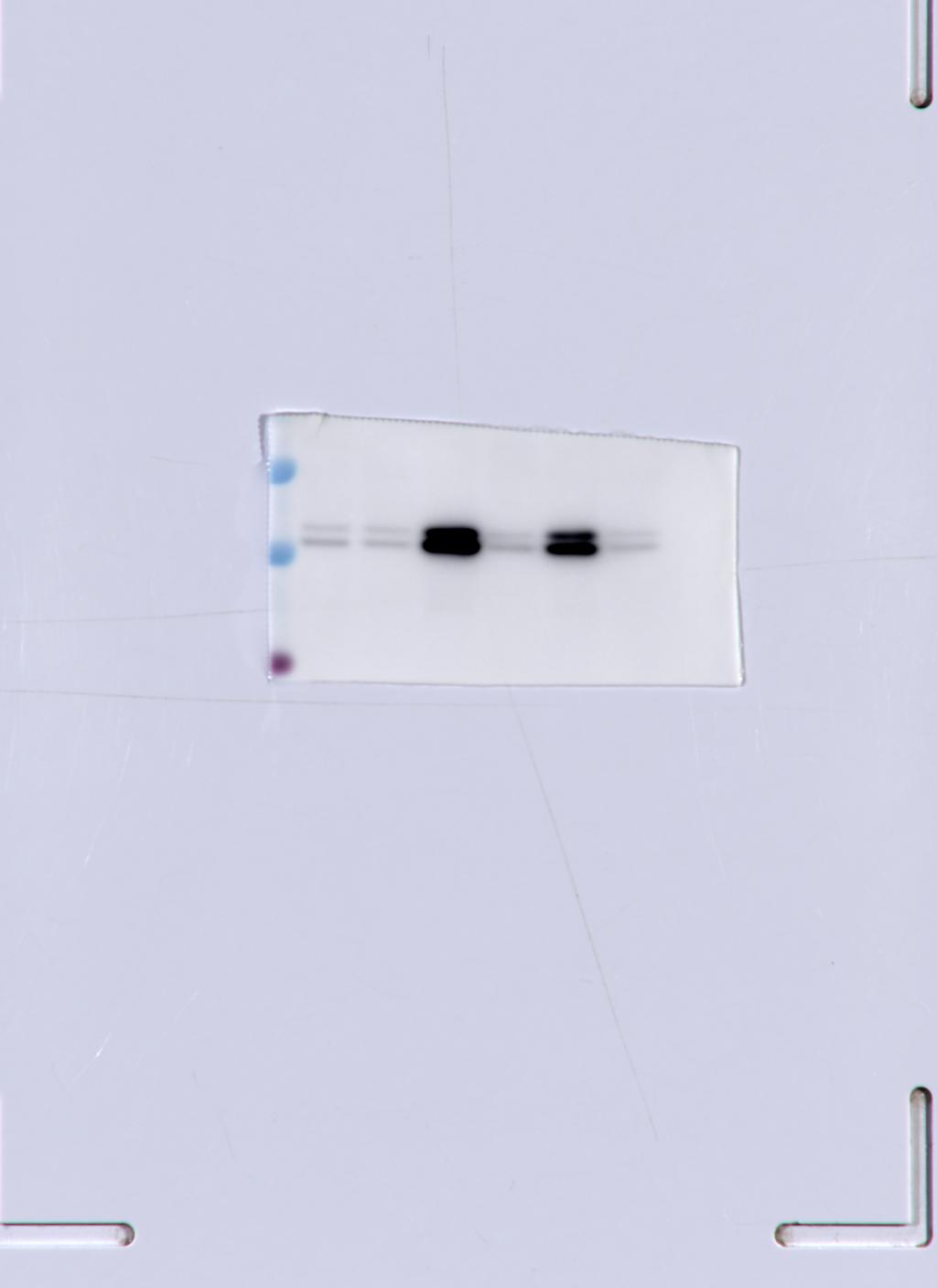

Supplement: Figure 2—source data 2. [file elife-76183-fig2-data2.zip › Figure 2-source data 2/Figure 2B Input pErk.jpg]

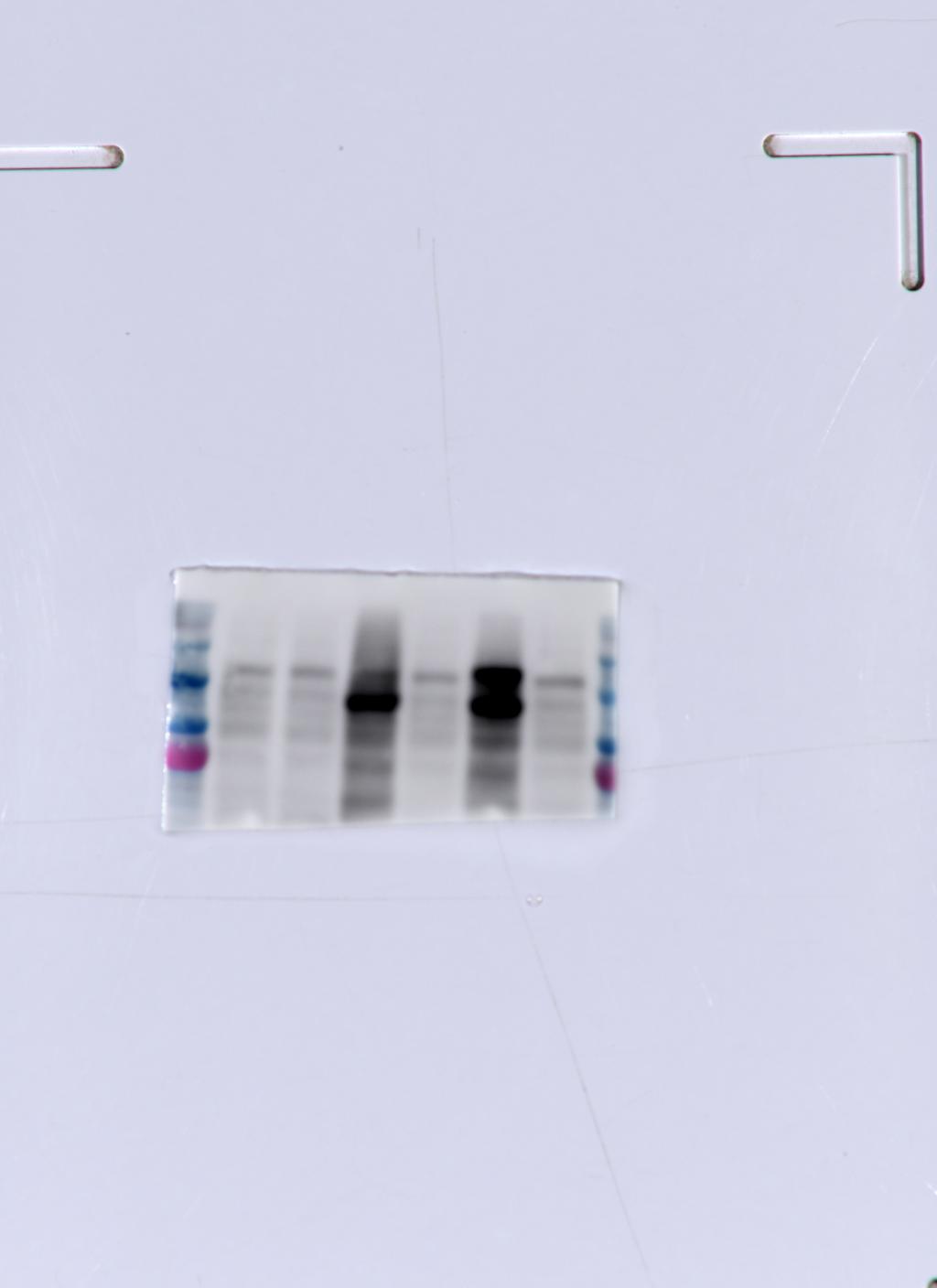

Supplement: Figure 2—source data 2. [file elife-76183-fig2-data2.zip › Figure 2-source data 2/Figure 2B Input WB 4G10.jpg]

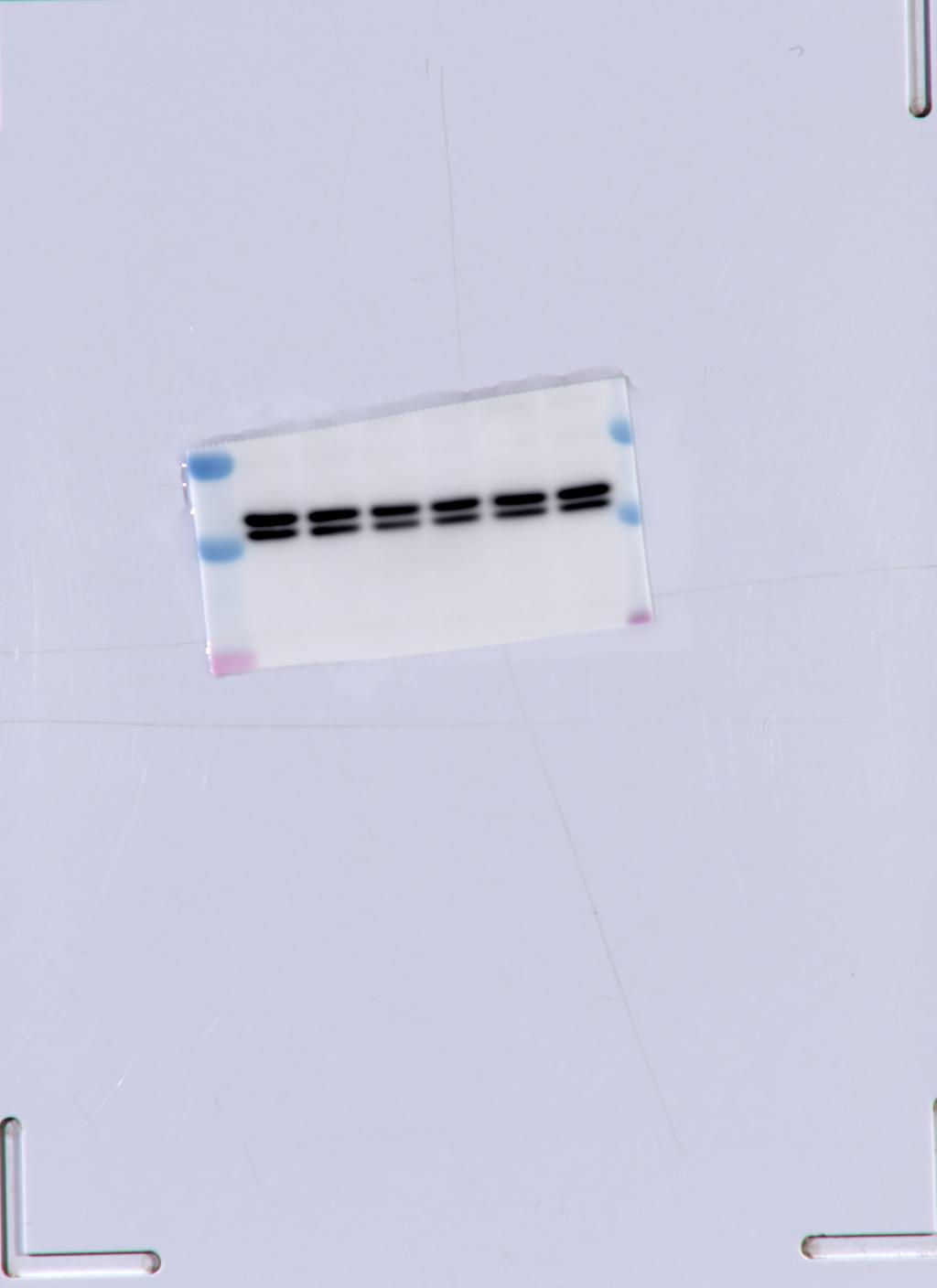

Supplement: Figure 2—source data 2. [file elife-76183-fig2-data2.zip › Figure 2-source data 2/Figure 2B Input WB Erk.jpg]

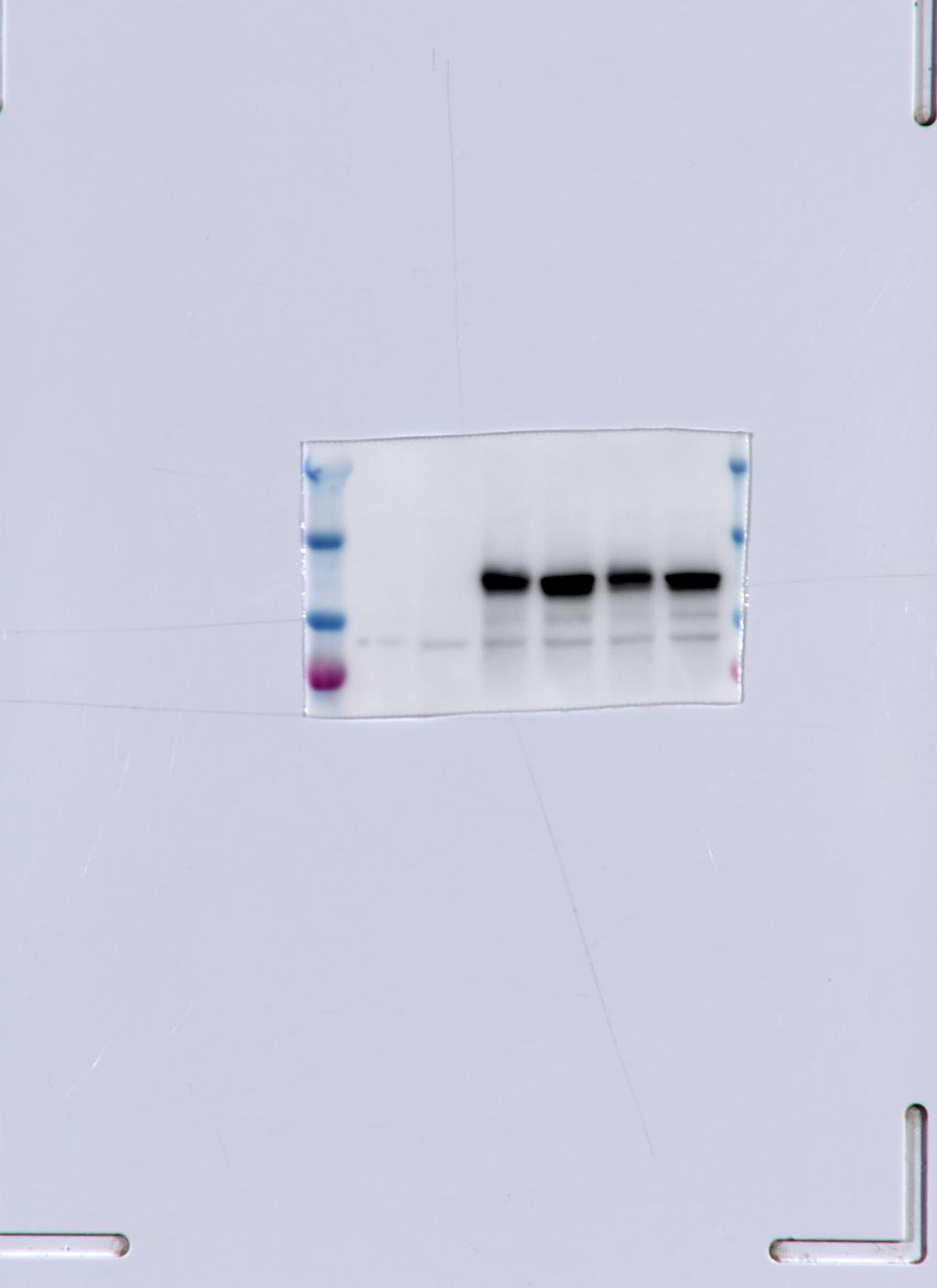

Supplement: Figure 2—source data 2. [file elife-76183-fig2-data2.zip › Figure 2-source data 2/Figure 2B Input WB FER.jpg]

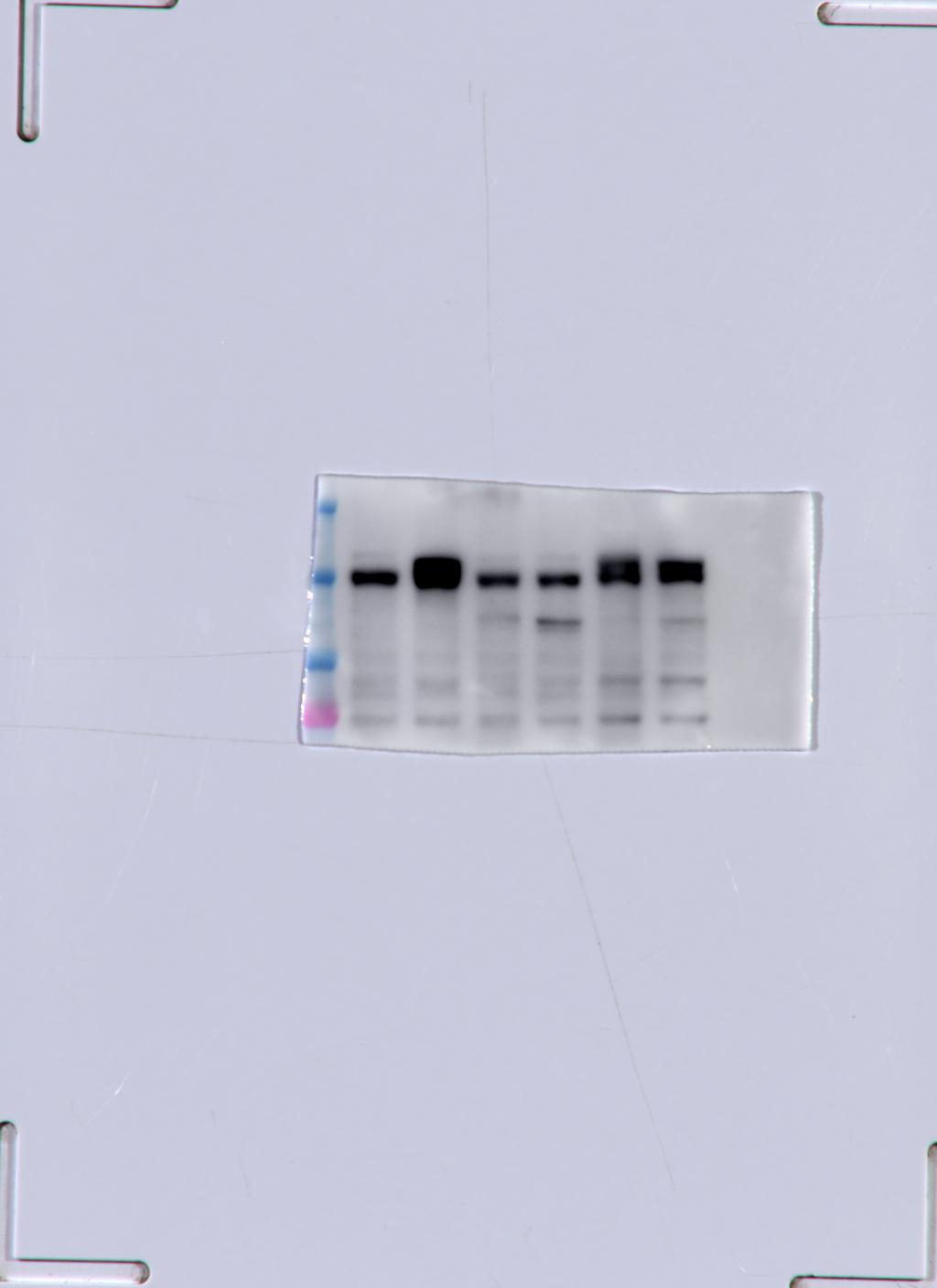

Supplement: Figure 2—source data 2. [file elife-76183-fig2-data2.zip › Figure 2-source data 2/Figure 2B Input WB IRS4.jpg]

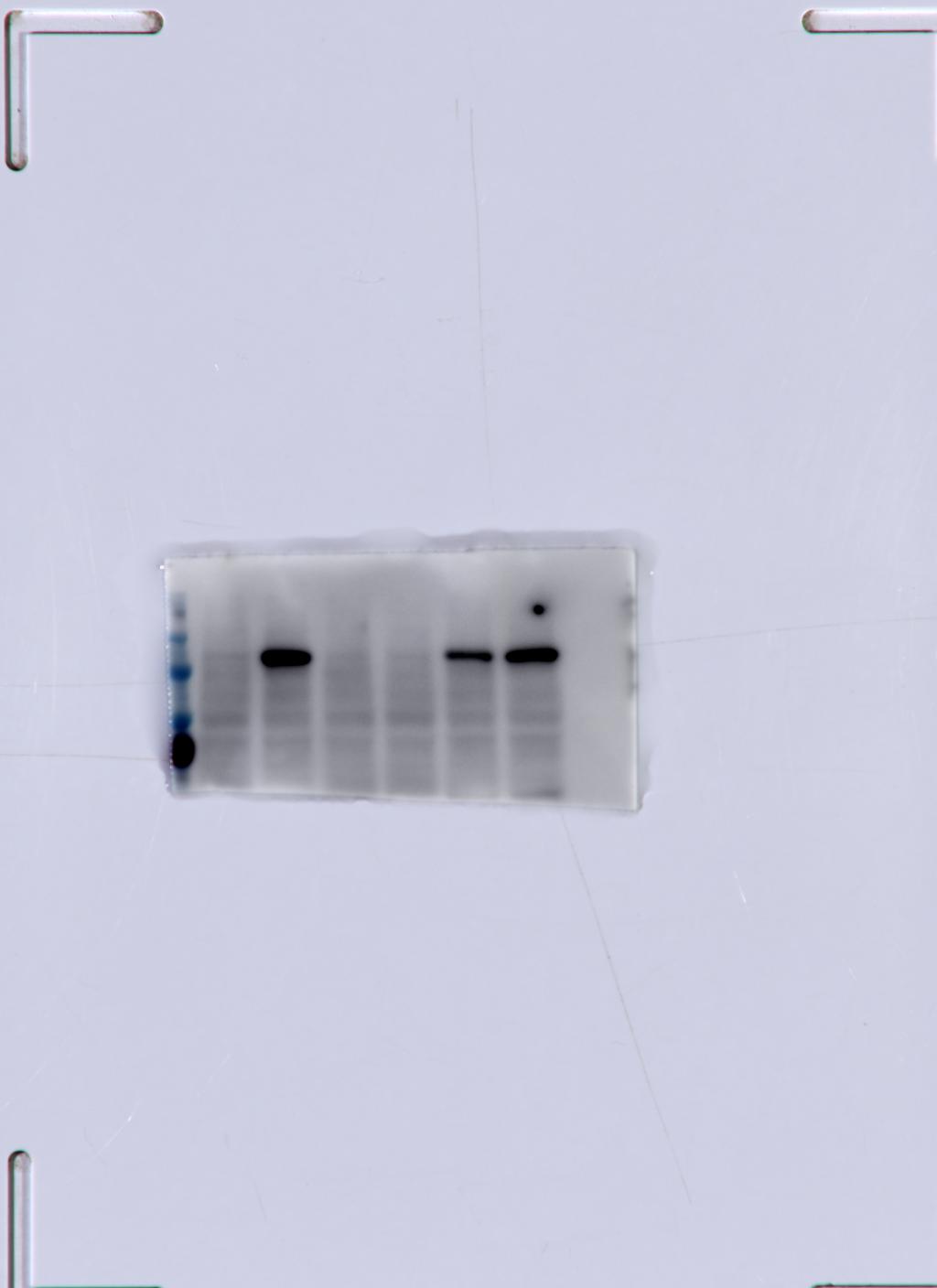

Supplement: Figure 2—source data 2. [file elife-76183-fig2-data2.zip › Figure 2-source data 2/Figure 2B Input WB Myc.jpg]

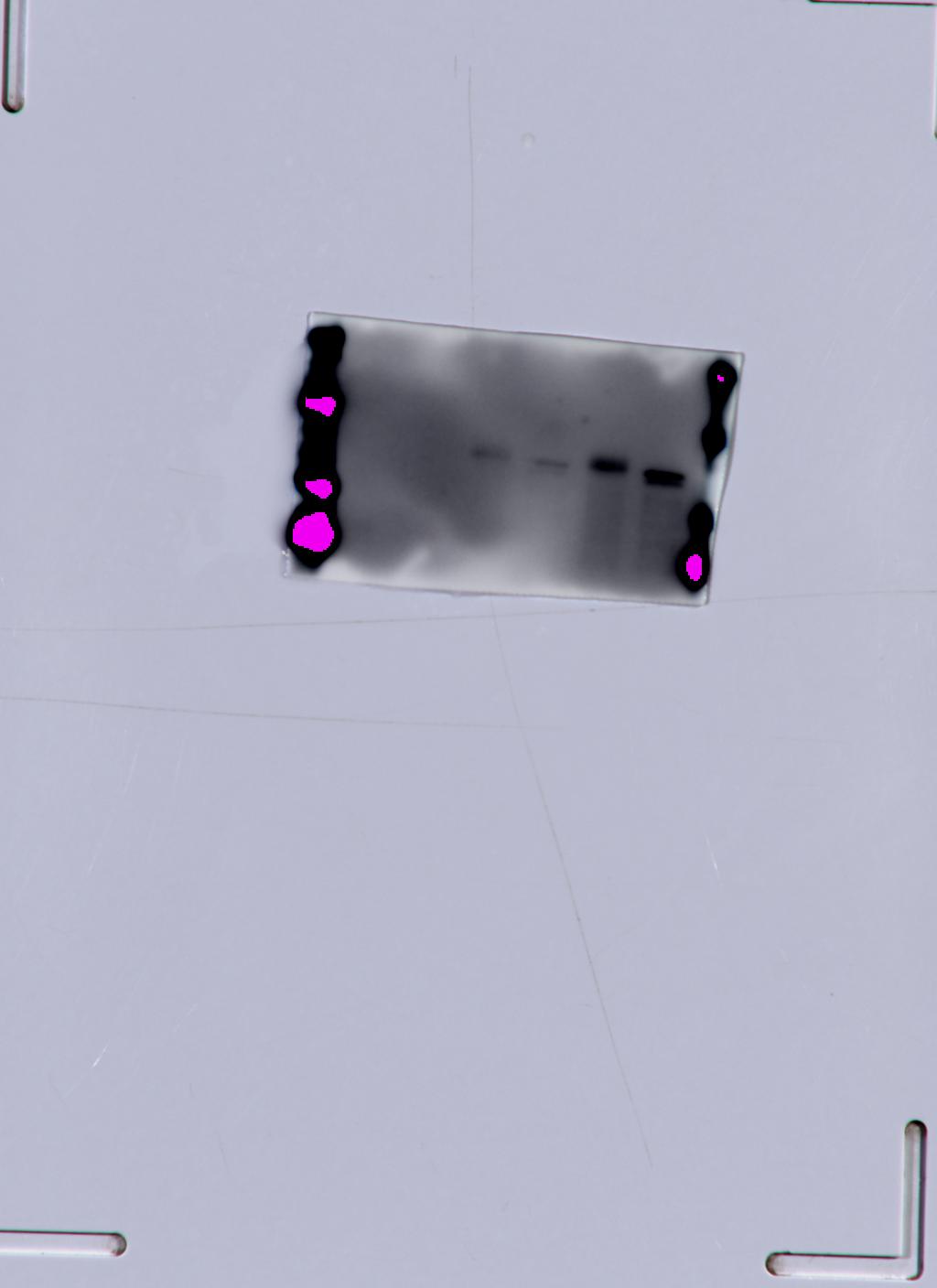

Supplement: Figure 2—source data 2. [file elife-76183-fig2-data2.zip › Figure 2-source data 2/Figure 2B IP Myc WB FER.jpg]

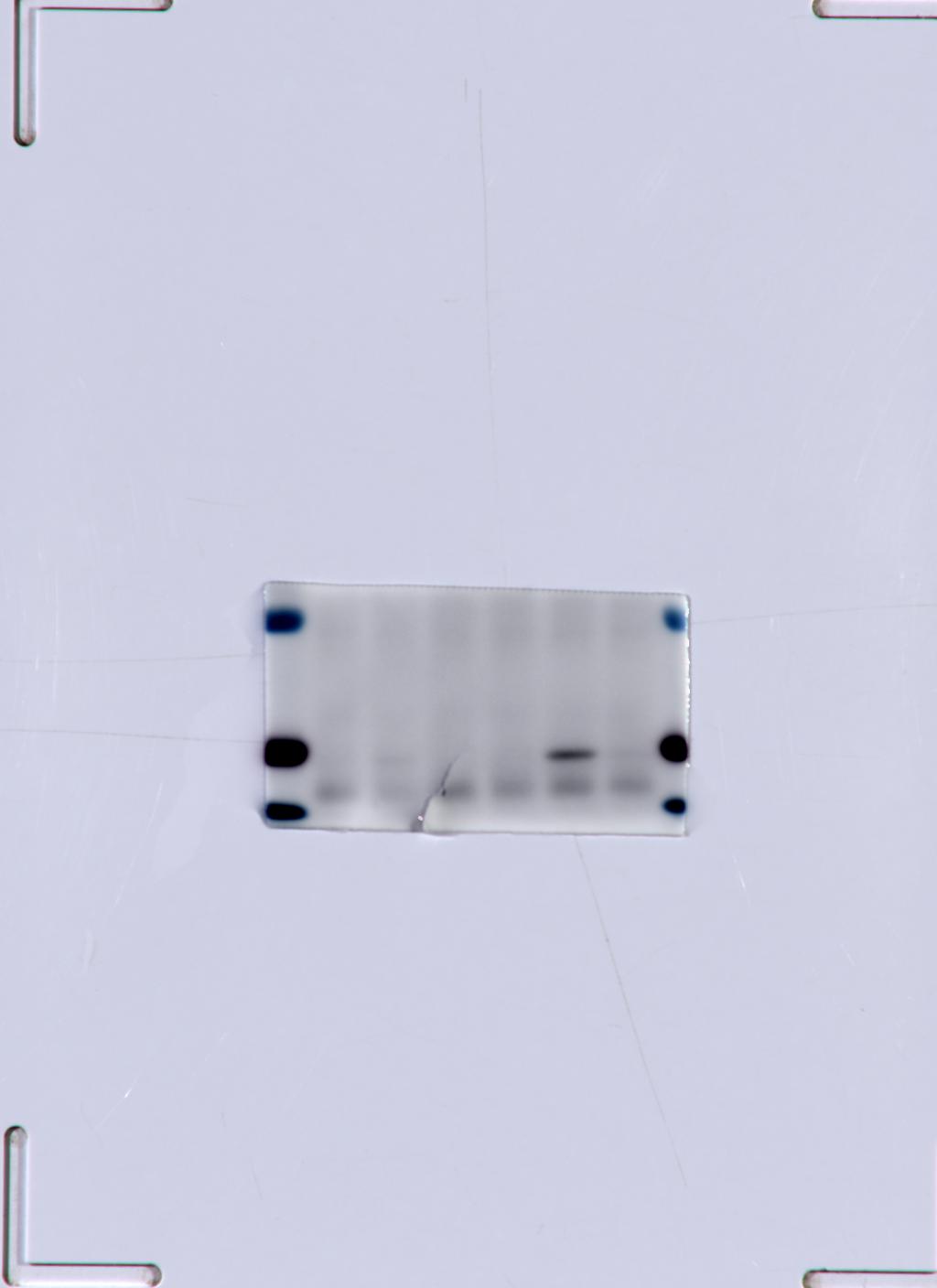

Supplement: Figure 2—source data 2. [file elife-76183-fig2-data2.zip › Figure 2-source data 2/Figure 2B IP Myc WB Grb2.jpg]

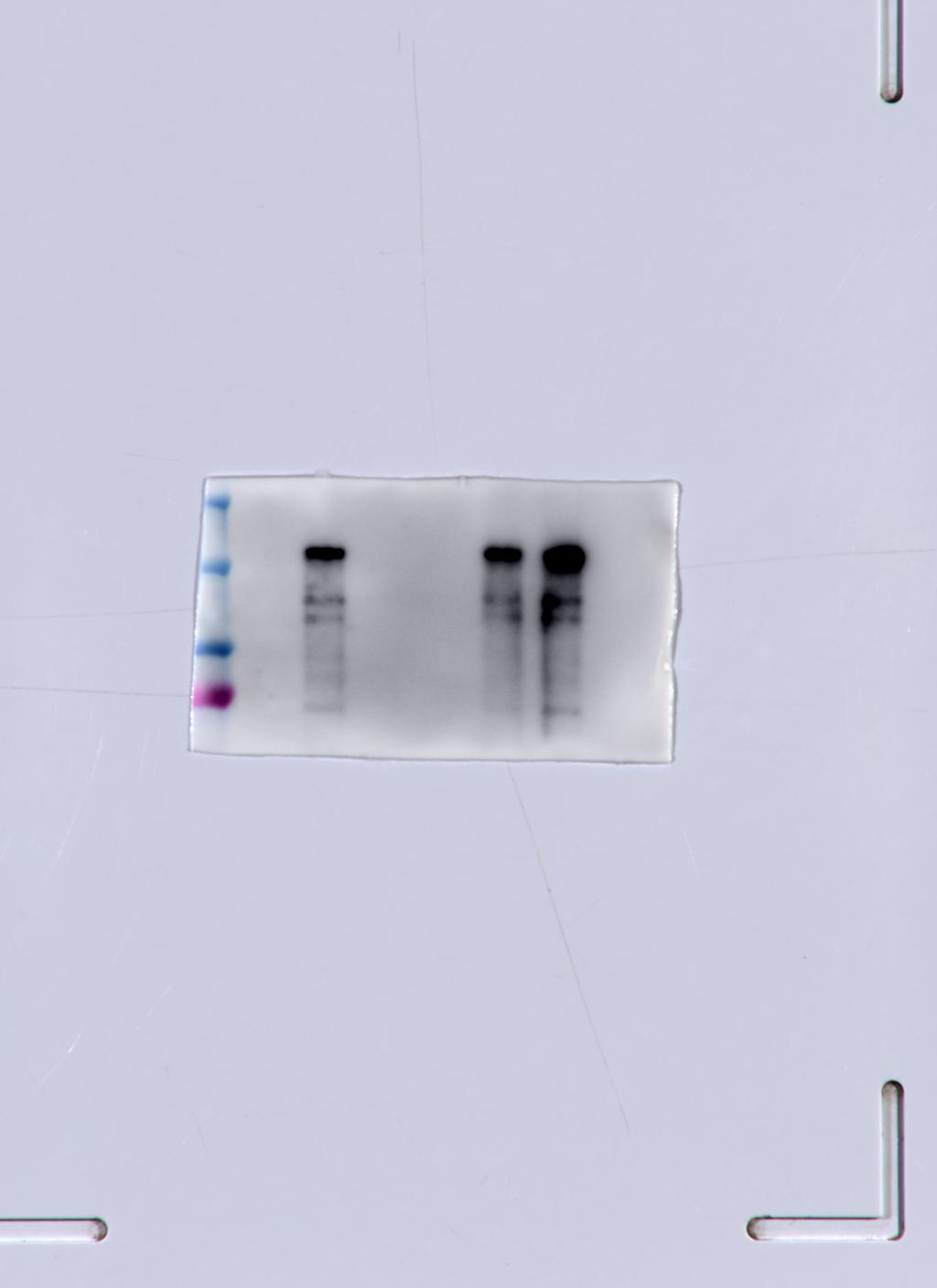

Supplement: Figure 2—source data 2. [file elife-76183-fig2-data2.zip › Figure 2-source data 2/Figure 2B IP Myc WB IRS4.jpg]

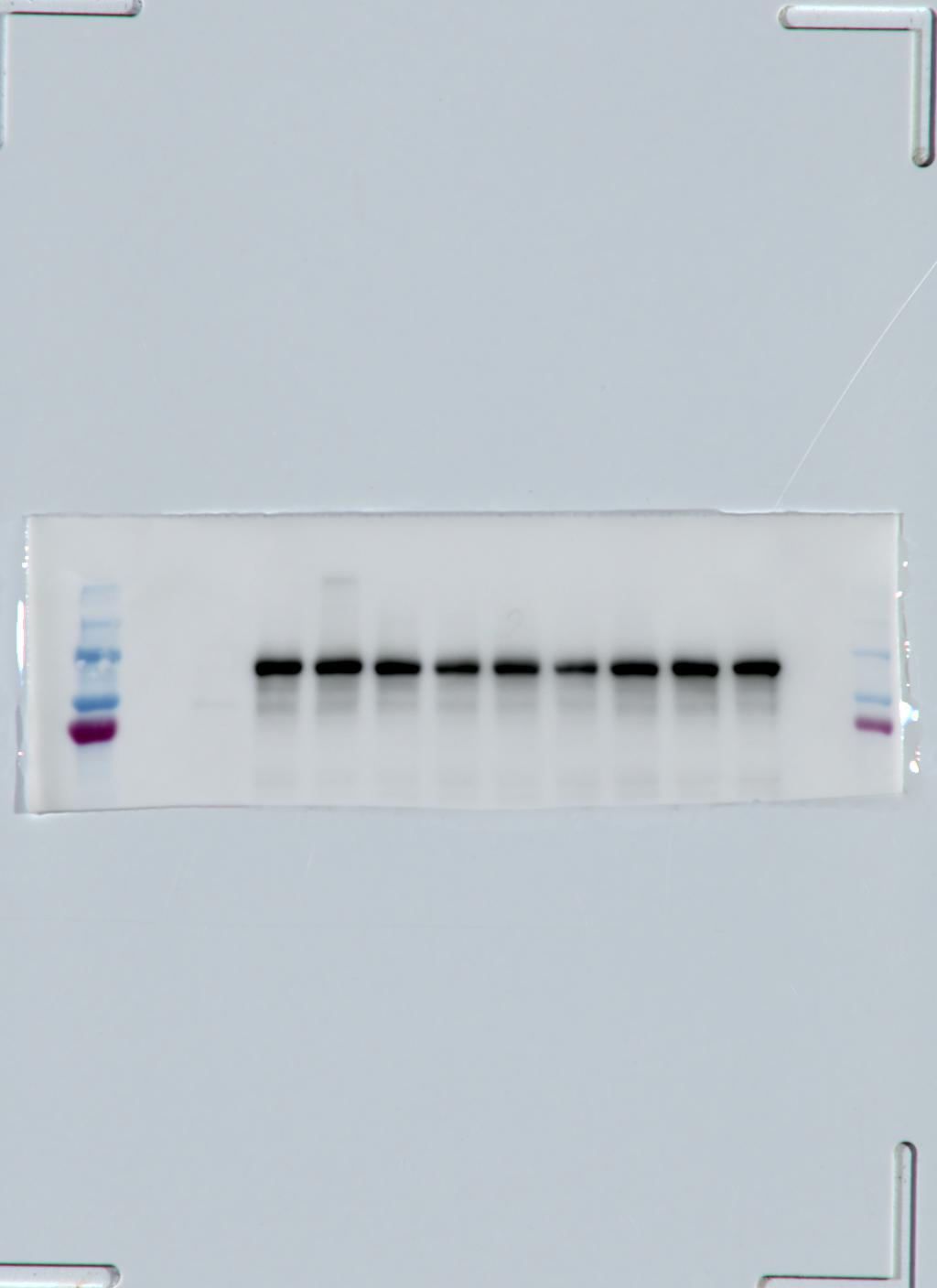

Supplement: Figure 2—source data 3. [file elife-76183-fig2-data3.zip › Figure 2-source data 3/Figure 2D input WB FER.jpg]

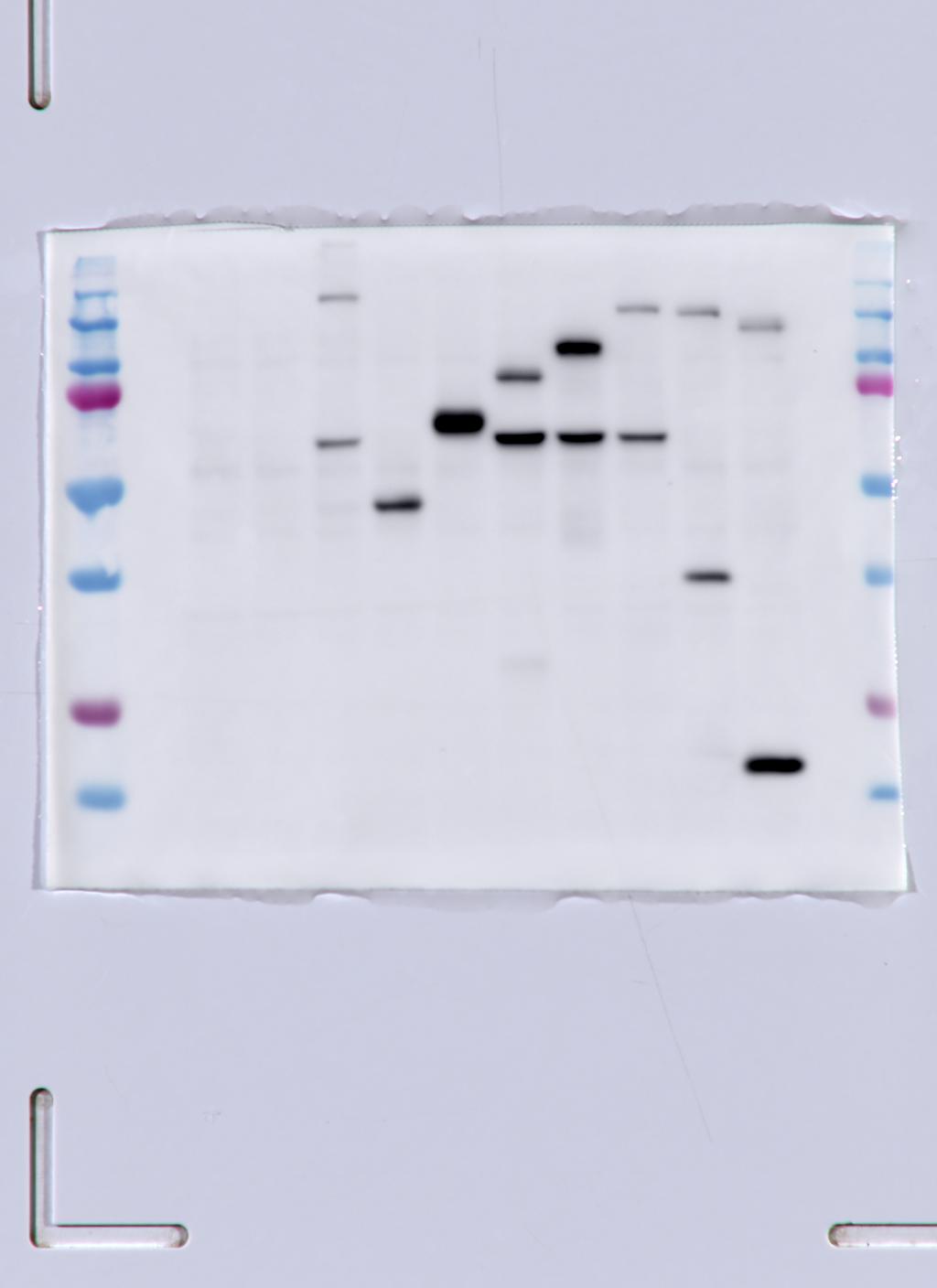

Supplement: Figure 2—source data 3. [file elife-76183-fig2-data3.zip › Figure 2-source data 3/Figure 2D input WB Myc.jpg]

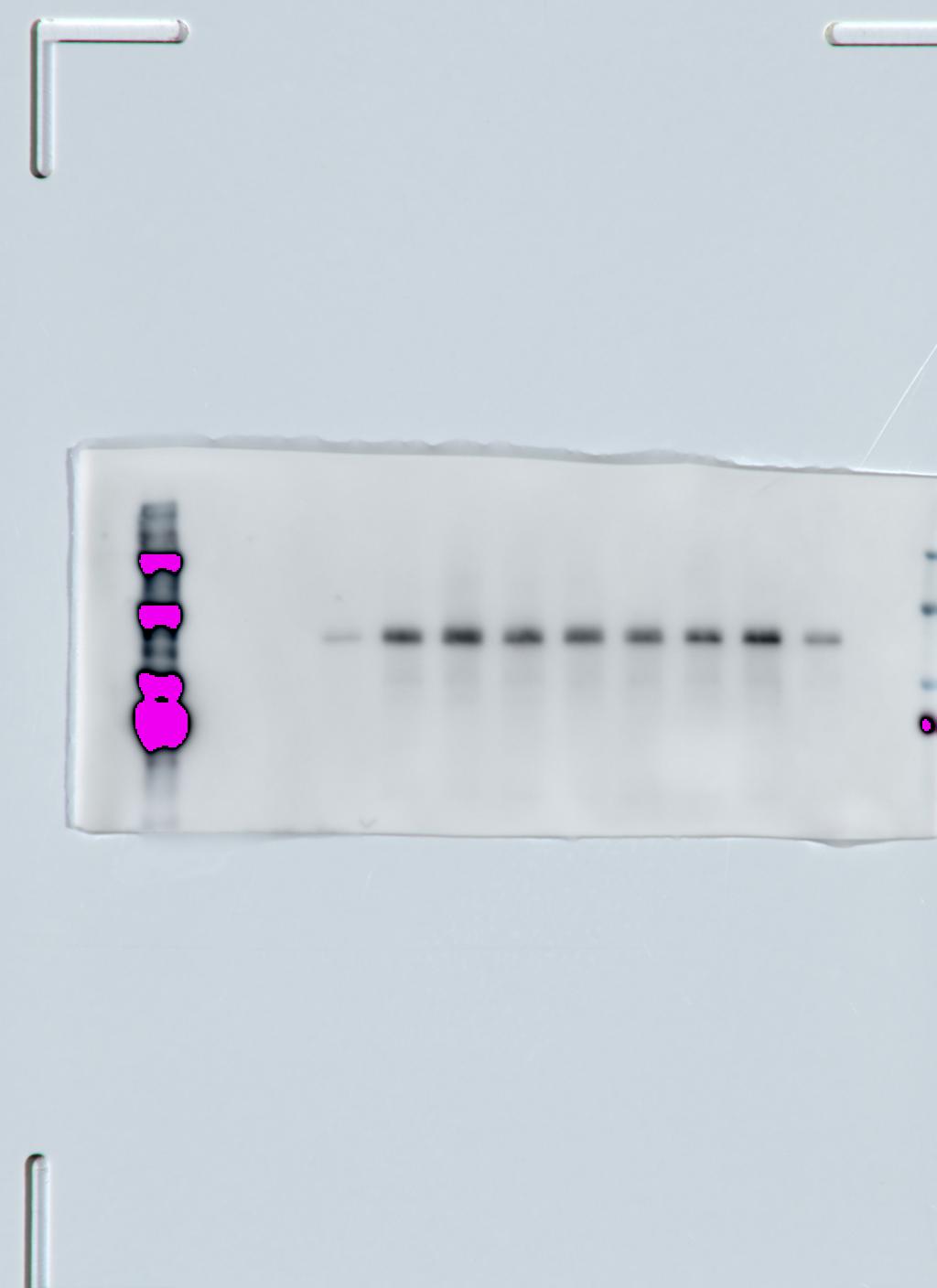

Supplement: Figure 2—source data 3. [file elife-76183-fig2-data3.zip › Figure 2-source data 3/Figure 2D IP Myc WB FER.jpg]

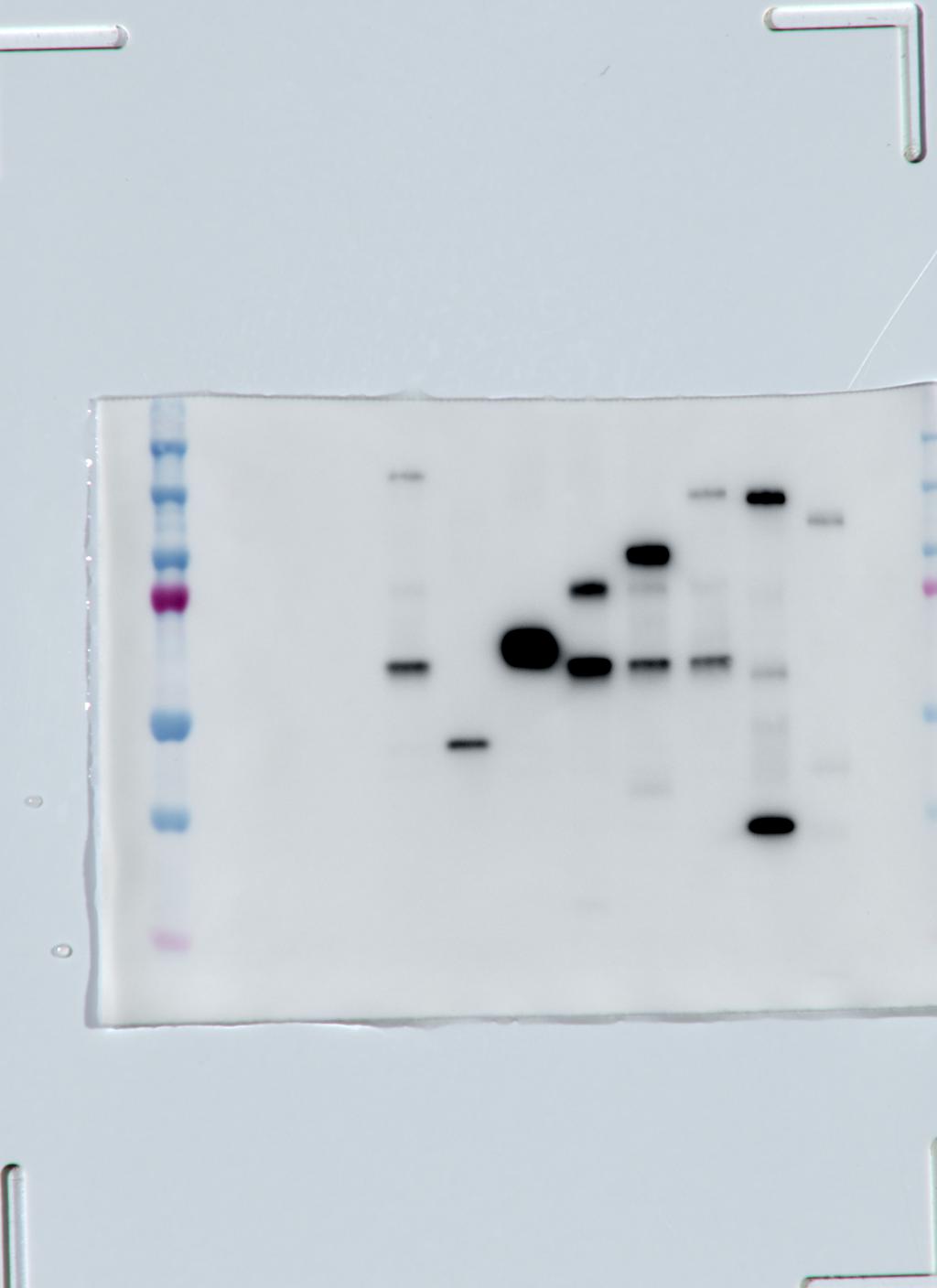

Supplement: Figure 2—source data 3. [file elife-76183-fig2-data3.zip › Figure 2-source data 3/Figure 2D IP Myc WB Myc.jpg]

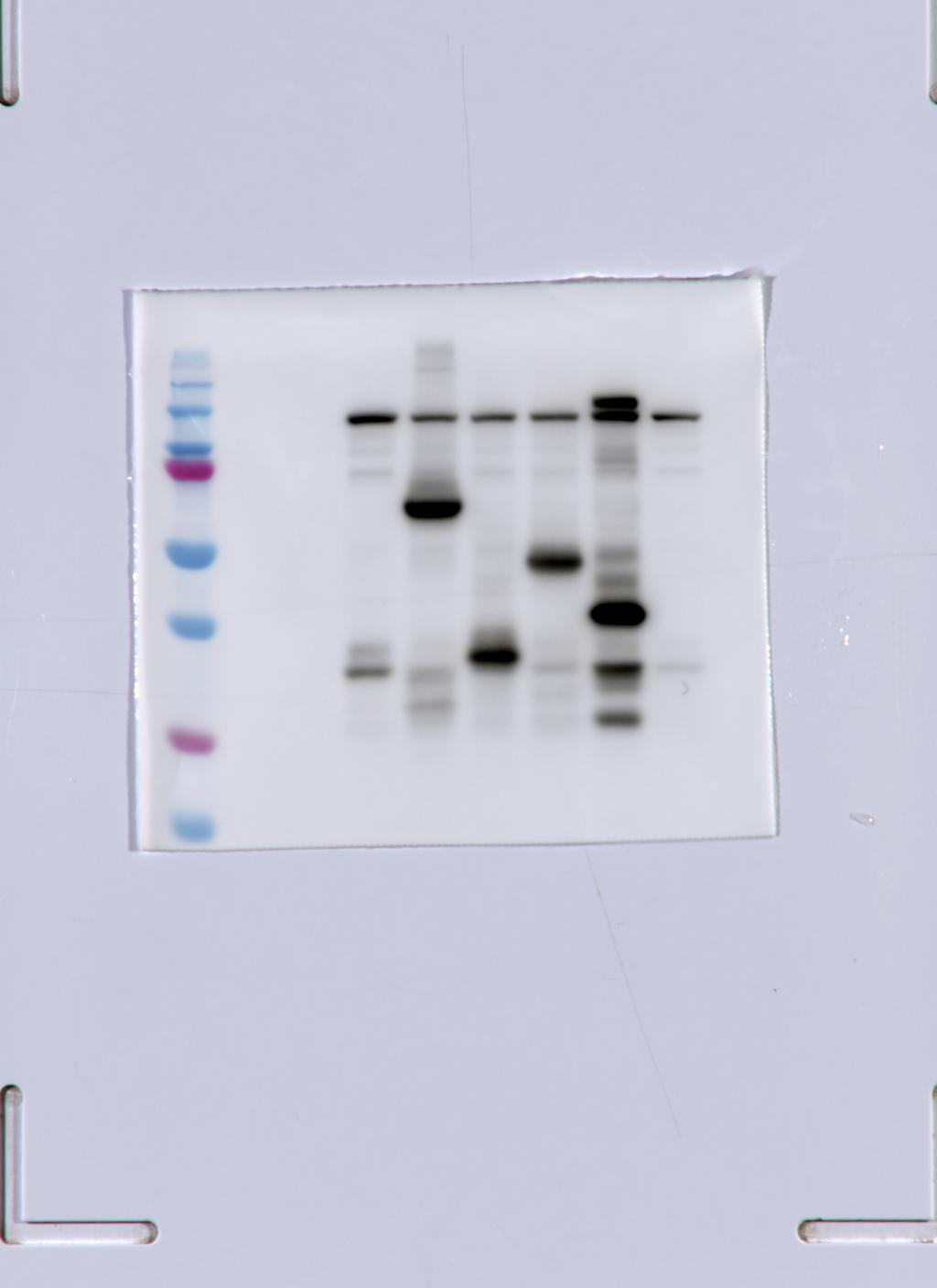

Supplement: Figure 2—source data 4. [file elife-76183-fig2-data4.zip › Figure 2-source data 4/Figure 2E input WB GFP.jpg]

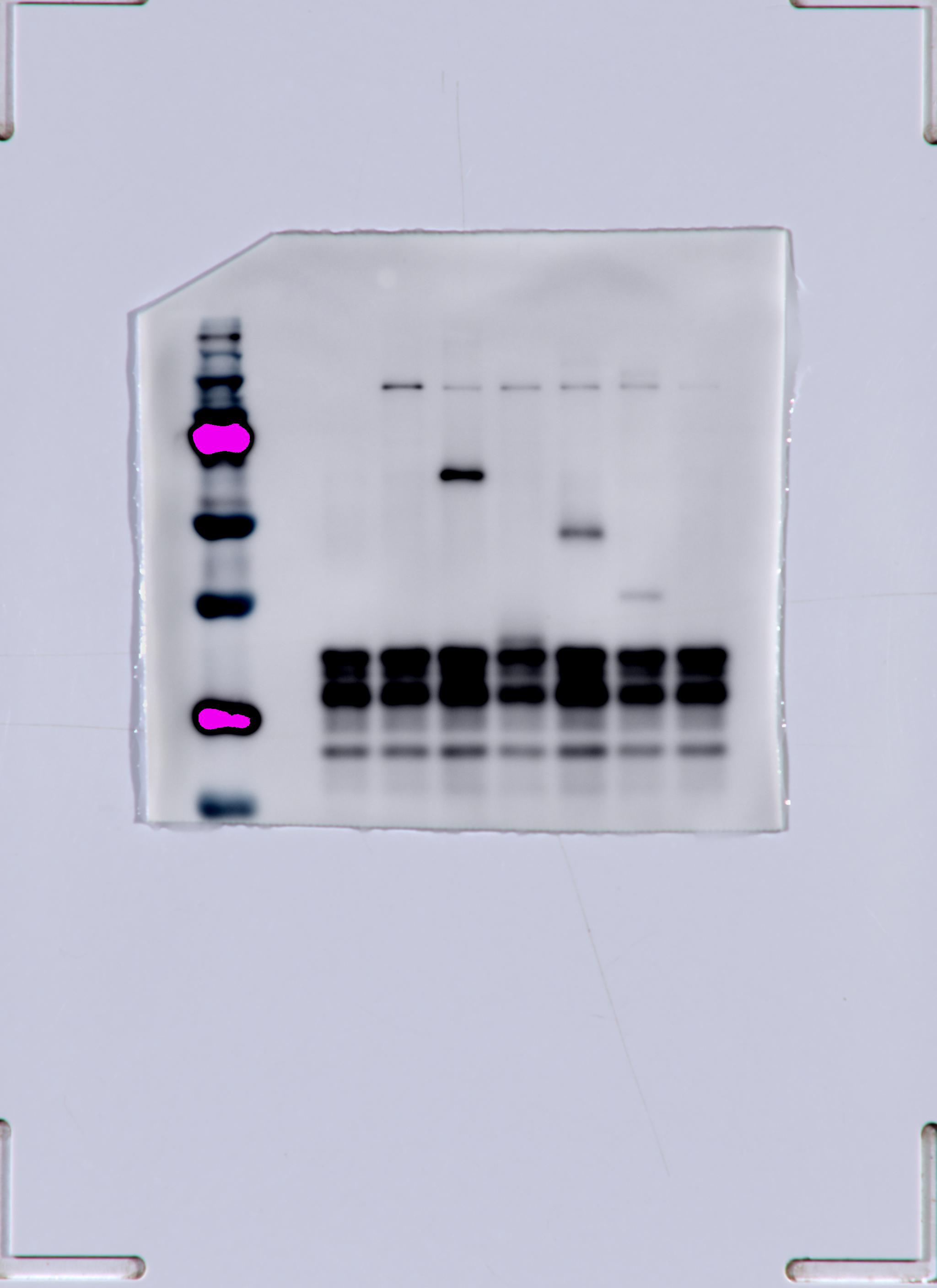

Supplement: Figure 2—source data 4. [file elife-76183-fig2-data4.zip › Figure 2-source data 4/Figure 2E IP FER WB GFP.jpg]

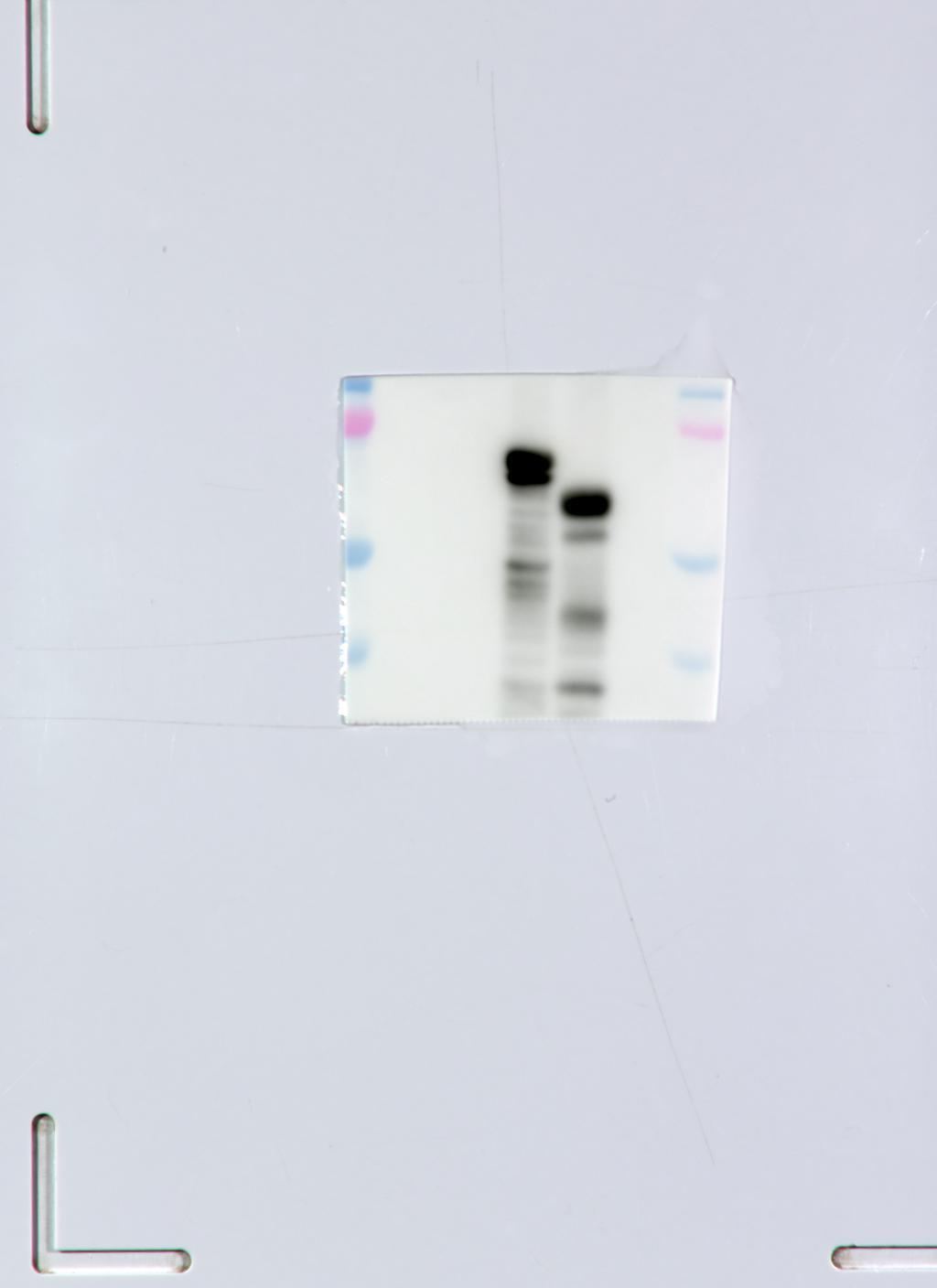

Supplement: Figure 2—source data 5. [file elife-76183-fig2-data5.zip › Figure 2-source data 5/Figure 2F Input WB GST.jpg]

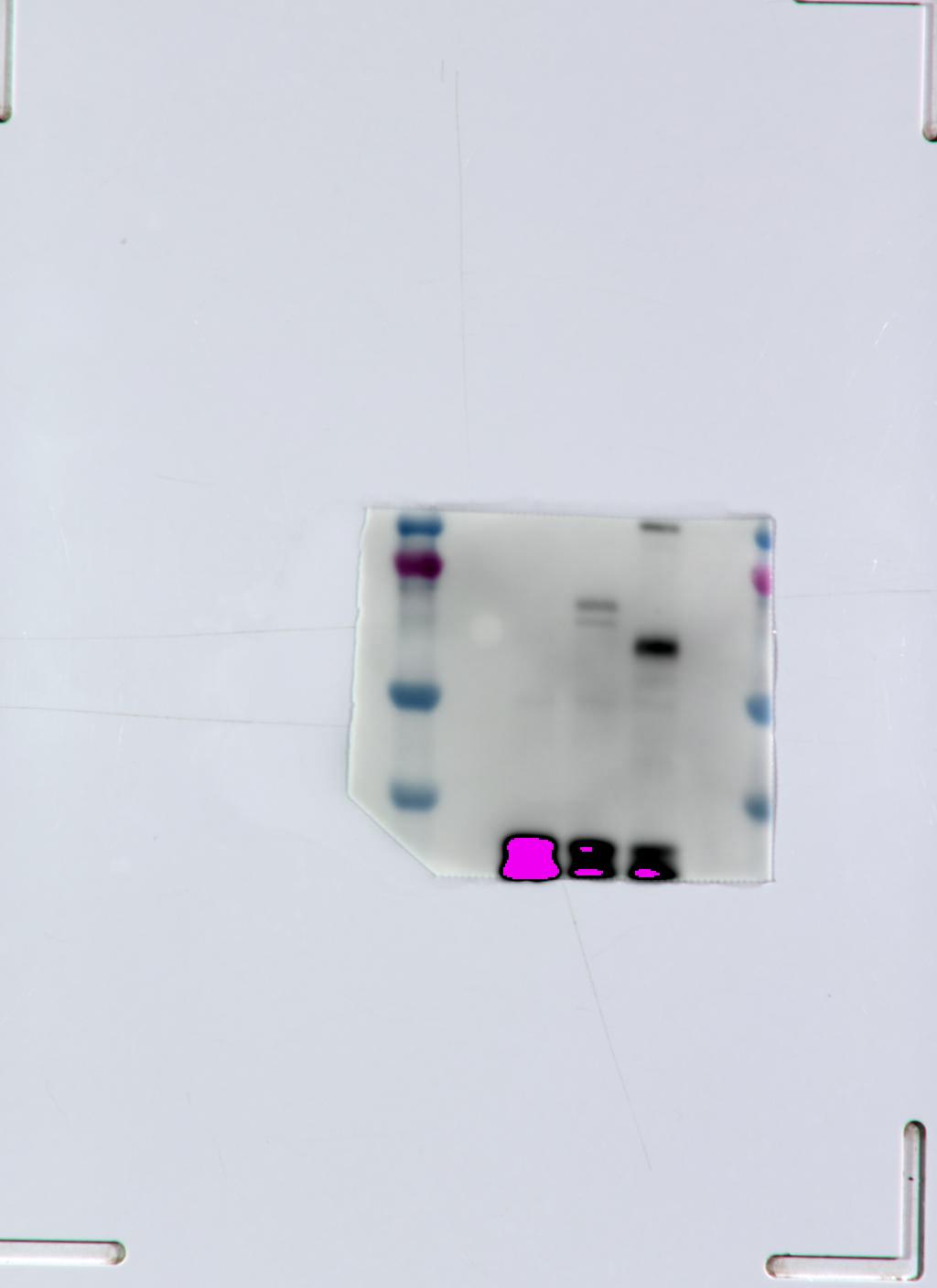

Supplement: Figure 2—source data 5. [file elife-76183-fig2-data5.zip › Figure 2-source data 5/Figure 2F IP Myc WB GST.jpg]

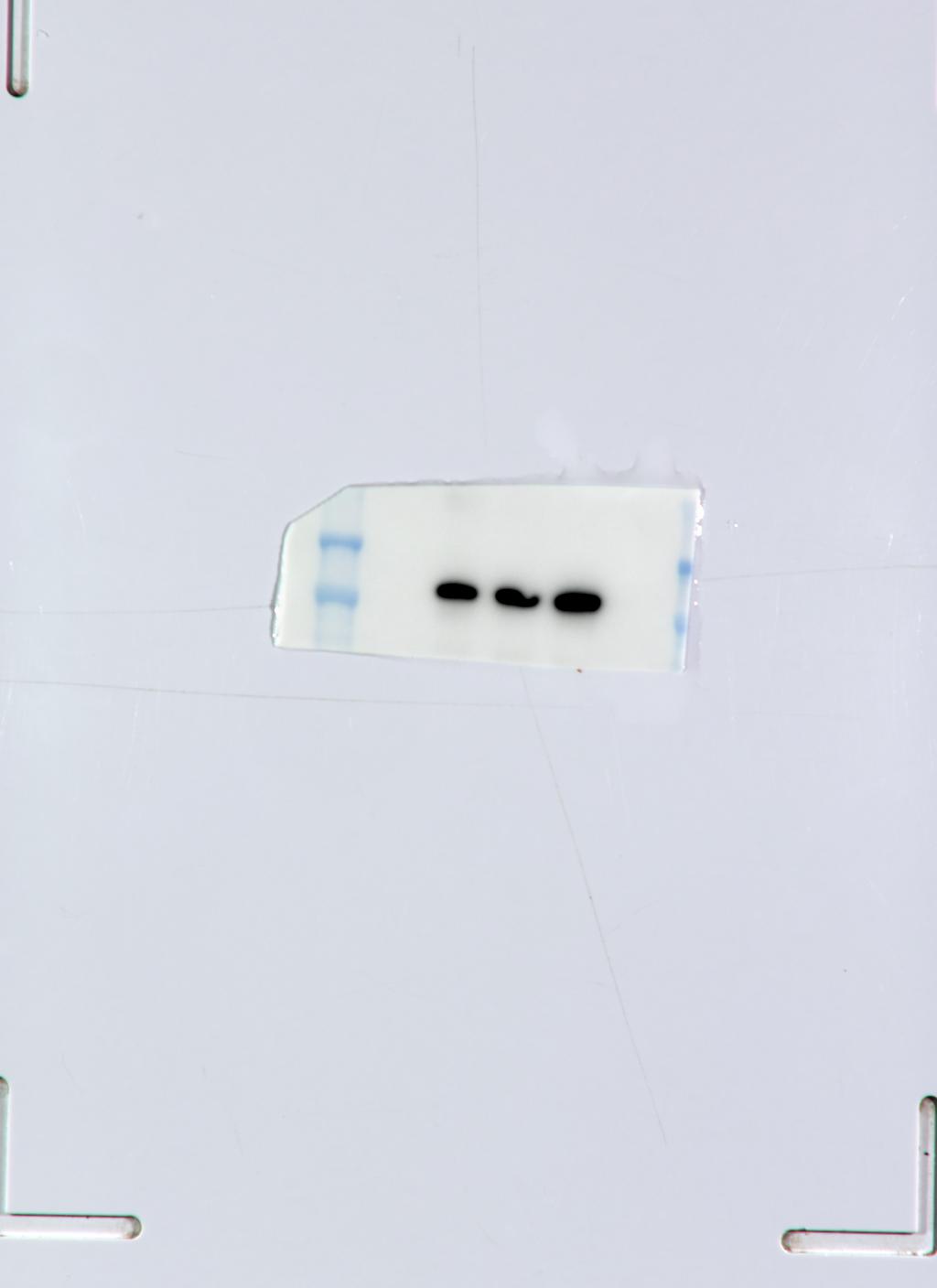

Supplement: Figure 2—source data 5. [file elife-76183-fig2-data5.zip › Figure 2-source data 5/Figure 2F IP Myc WB Myc.jpg]

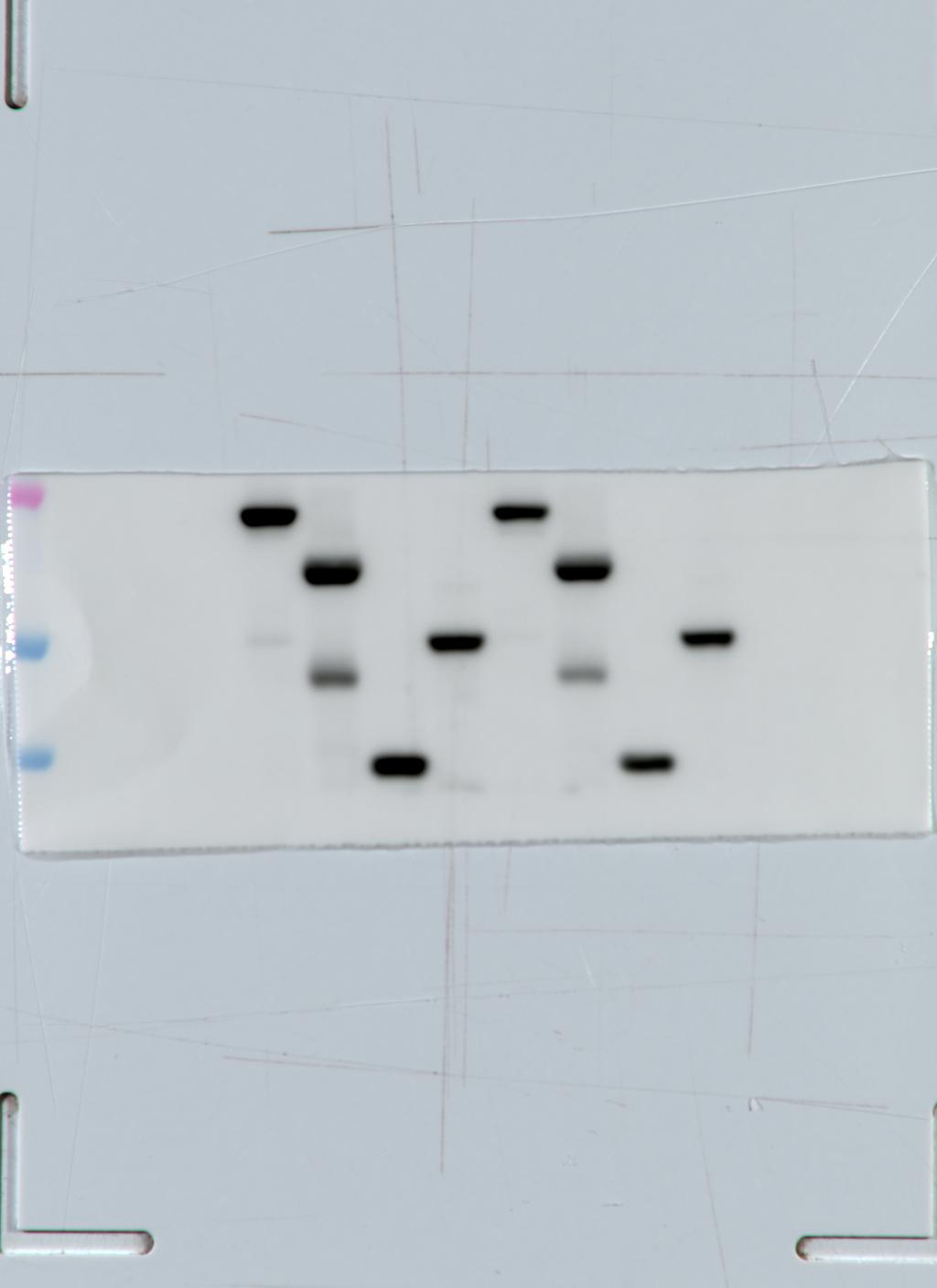

Supplement: Figure 2—source data 6. [file elife-76183-fig2-data6.zip › Figure 2-source data 6/Figure 2G INPUT-GFP.jpg]

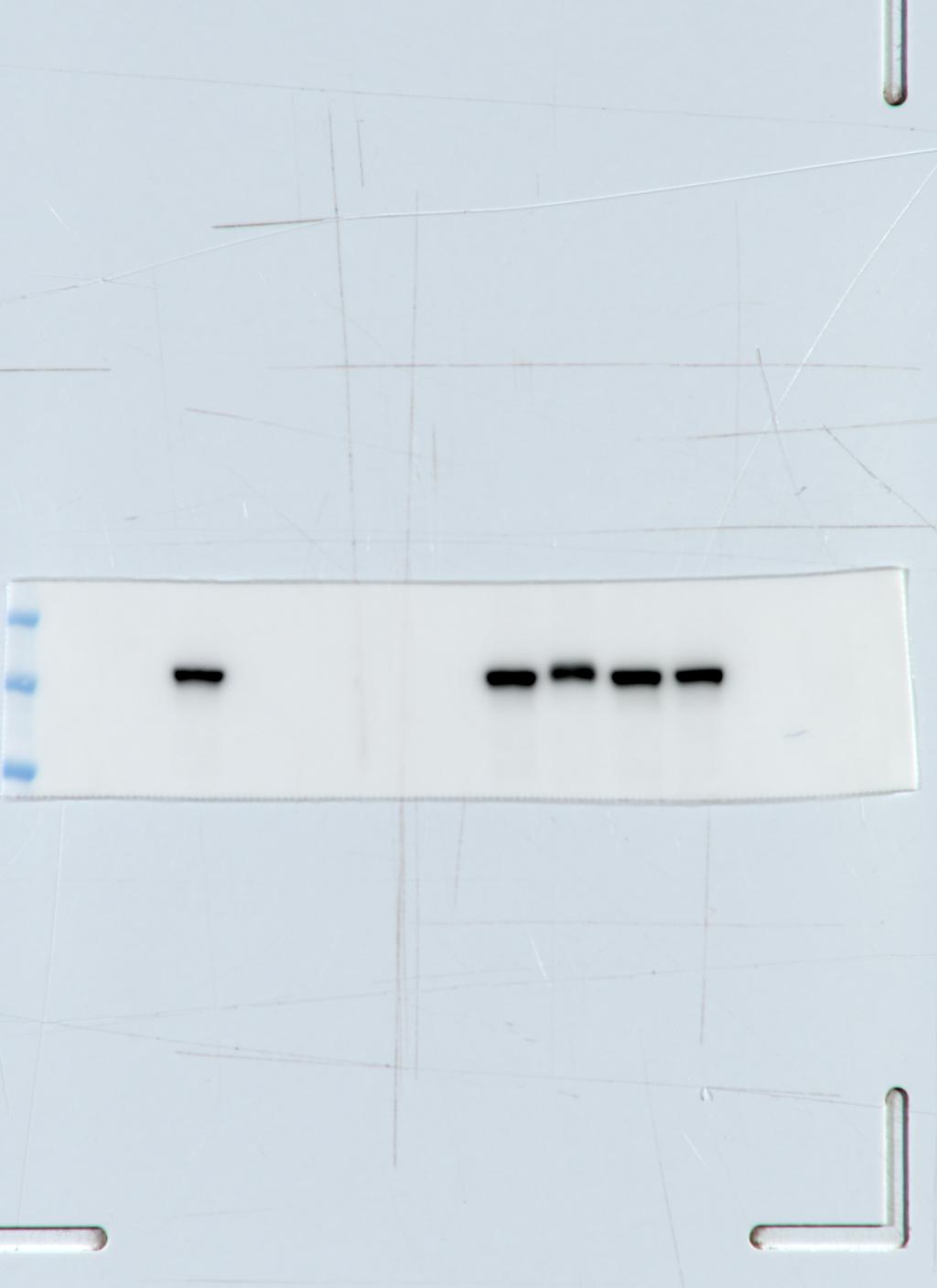

Supplement: Figure 2—source data 6. [file elife-76183-fig2-data6.zip › Figure 2-source data 6/Figure 2G INPUT-Myc.jpg]

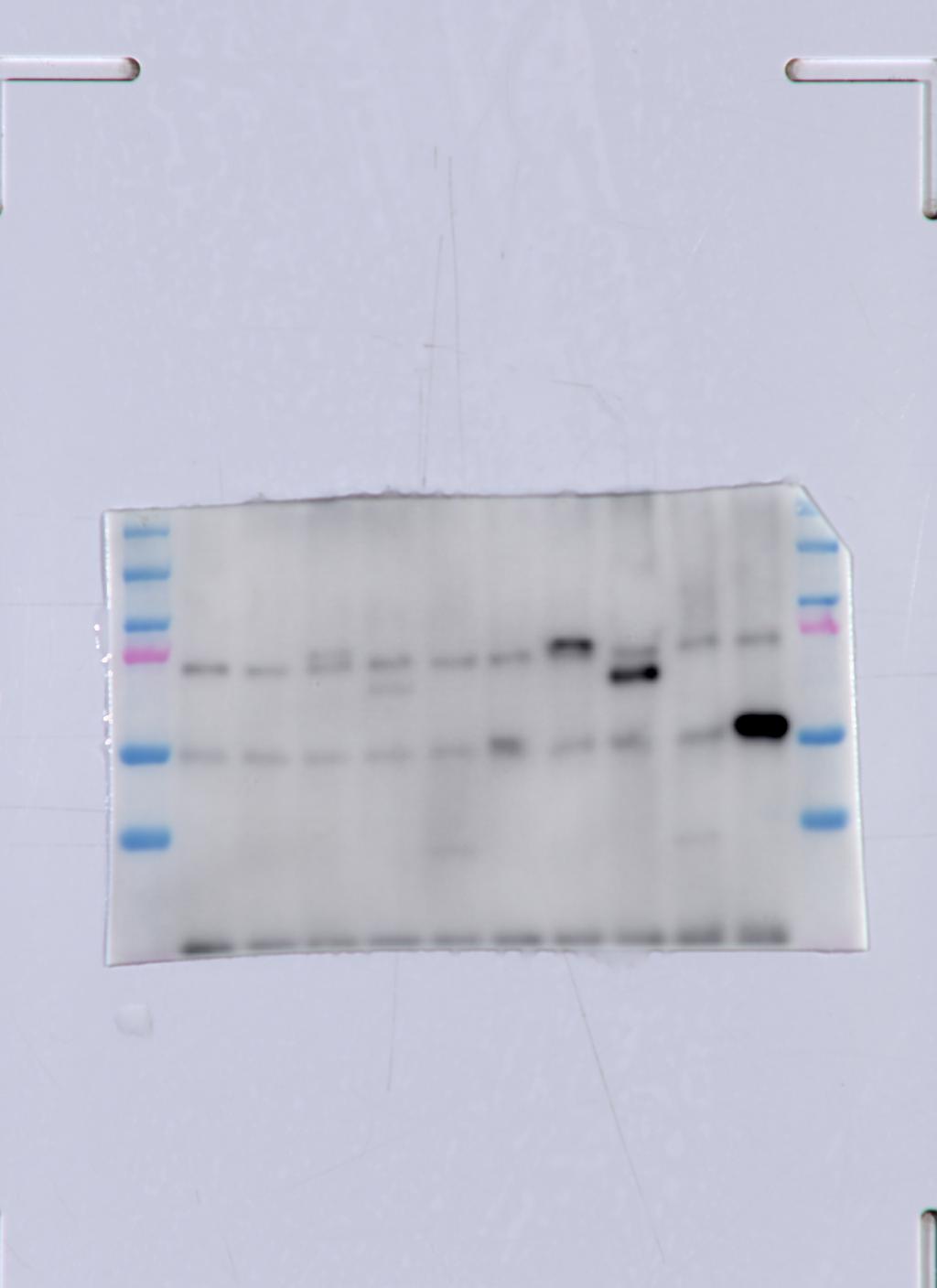

Supplement: Figure 2—source data 6. [file elife-76183-fig2-data6.zip › Figure 2-source data 6/Figure 2G IP-GFP.jpg]

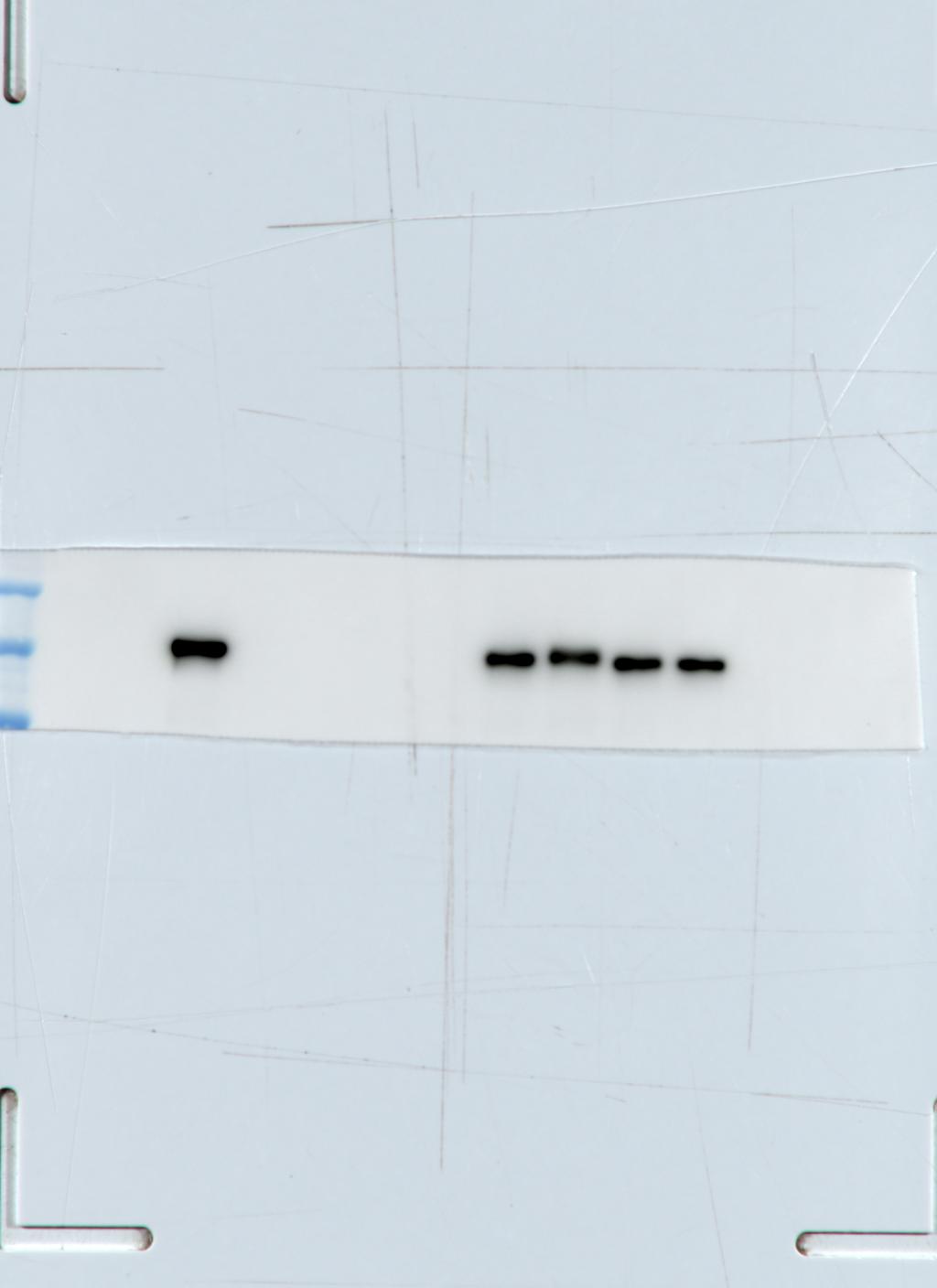

Supplement: Figure 2—source data 6. [file elife-76183-fig2-data6.zip › Figure 2-source data 6/Figure 2G IP-Myc.jpg]

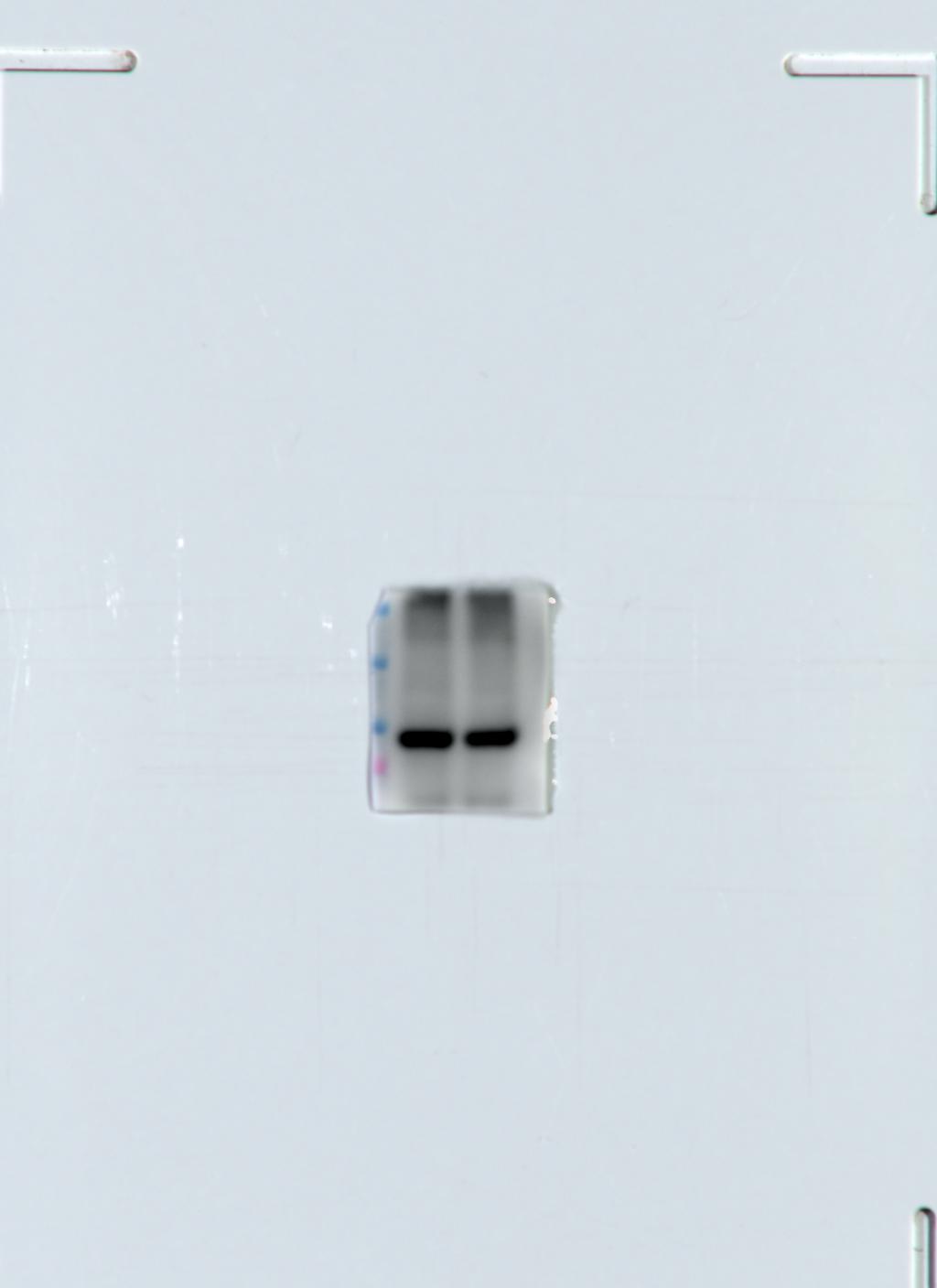

Supplement: Figure 2—figure supplement 1—source data 1. [file elife-76183-fig2-figsupp1-data1.zip › Figure 2-figure supplement 1-source data 1/Figure 2 S1A INPUT-FER.jpg]

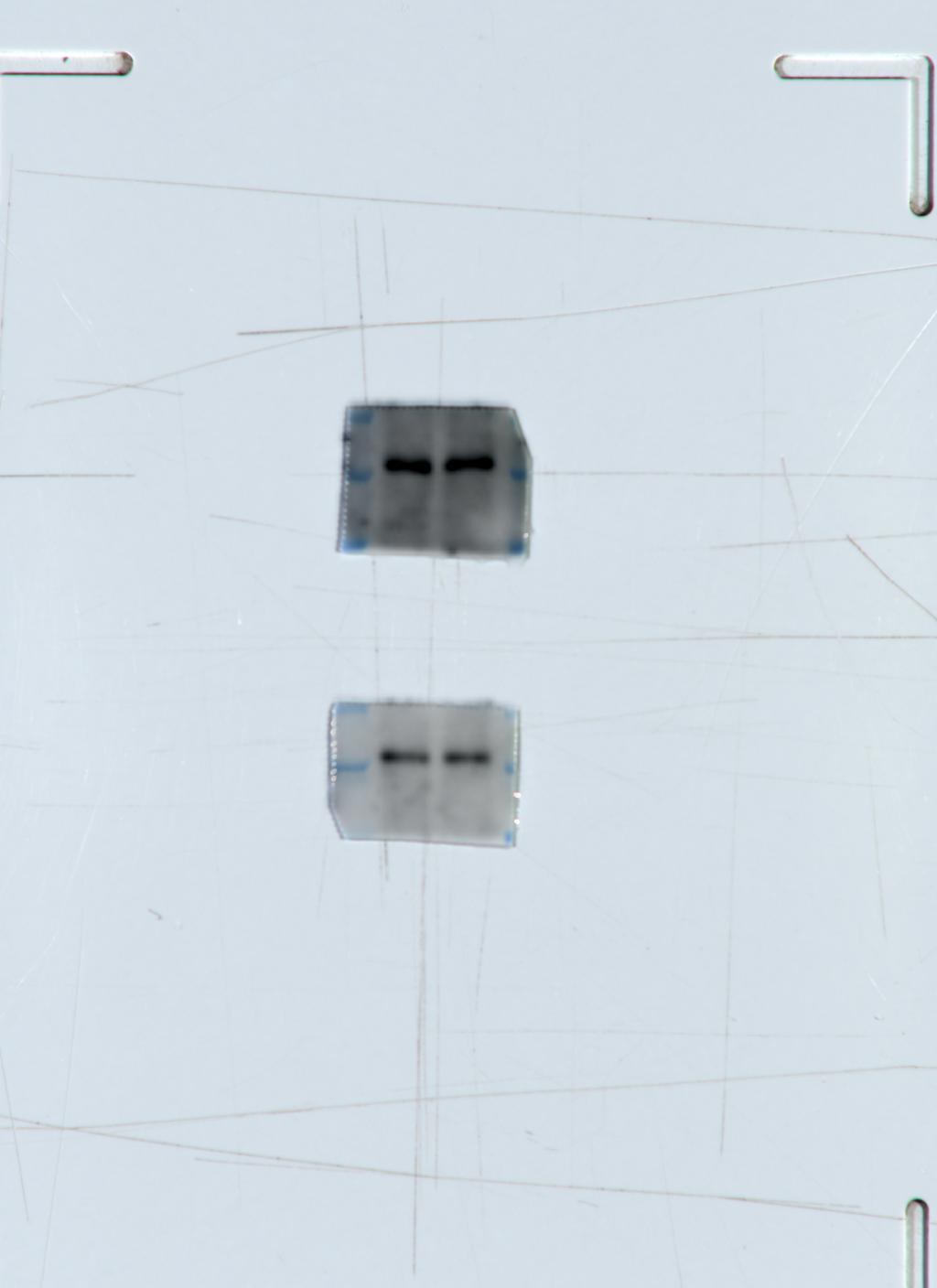

Supplement: Figure 2—figure supplement 1—source data 1. [file elife-76183-fig2-figsupp1-data1.zip › Figure 2-figure supplement 1-source data 1/Figure 2 S1A INPUT-IRS1.jpg]

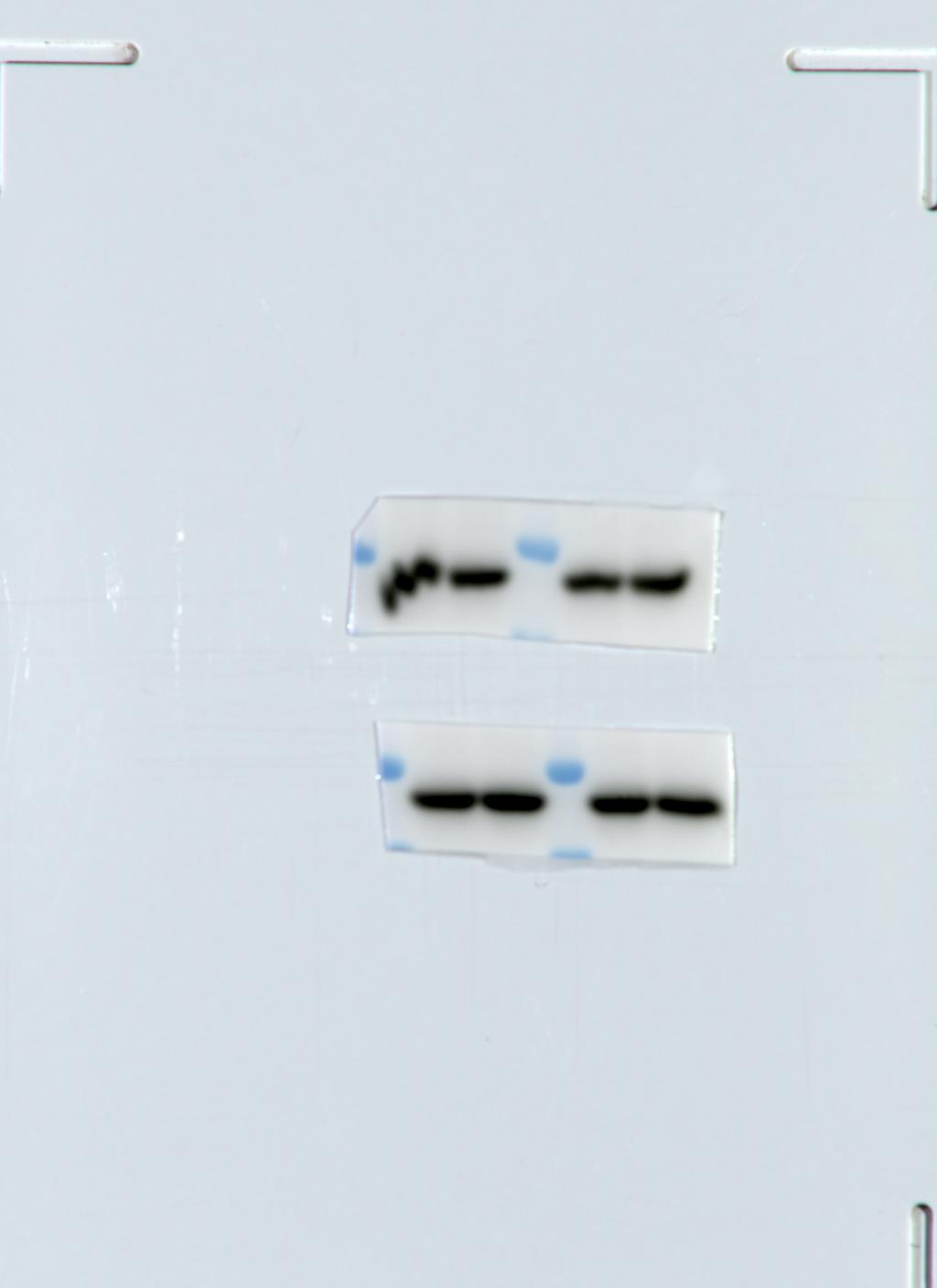

Supplement: Figure 2—figure supplement 1—source data 1. [file elife-76183-fig2-figsupp1-data1.zip › Figure 2-figure supplement 1-source data 1/Figure 2 S1A INPUT-Tubulin.jpg]

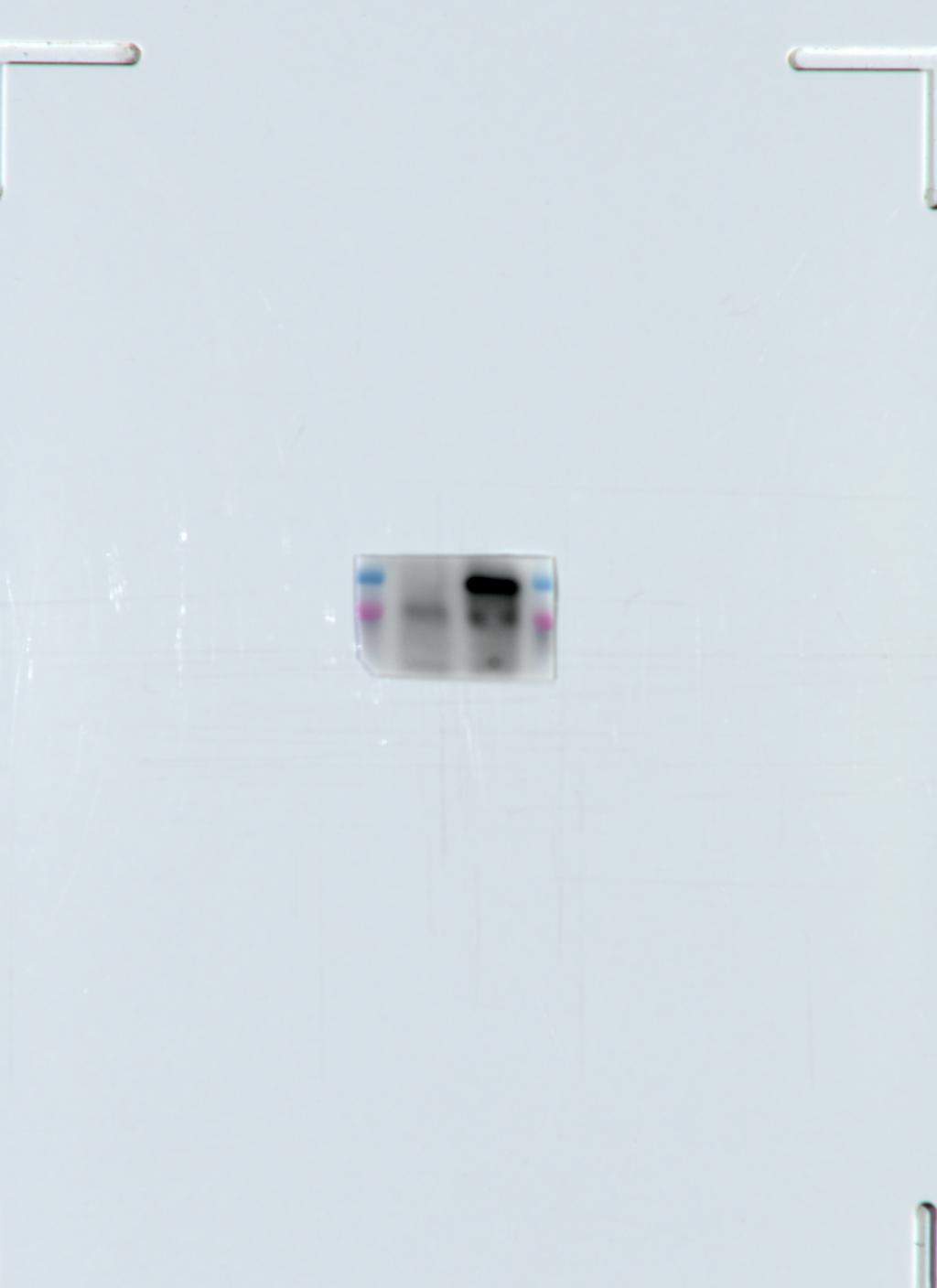

Supplement: Figure 2—figure supplement 1—source data 1. [file elife-76183-fig2-figsupp1-data1.zip › Figure 2-figure supplement 1-source data 1/Figure 2 S1A IP-FER.jpg]

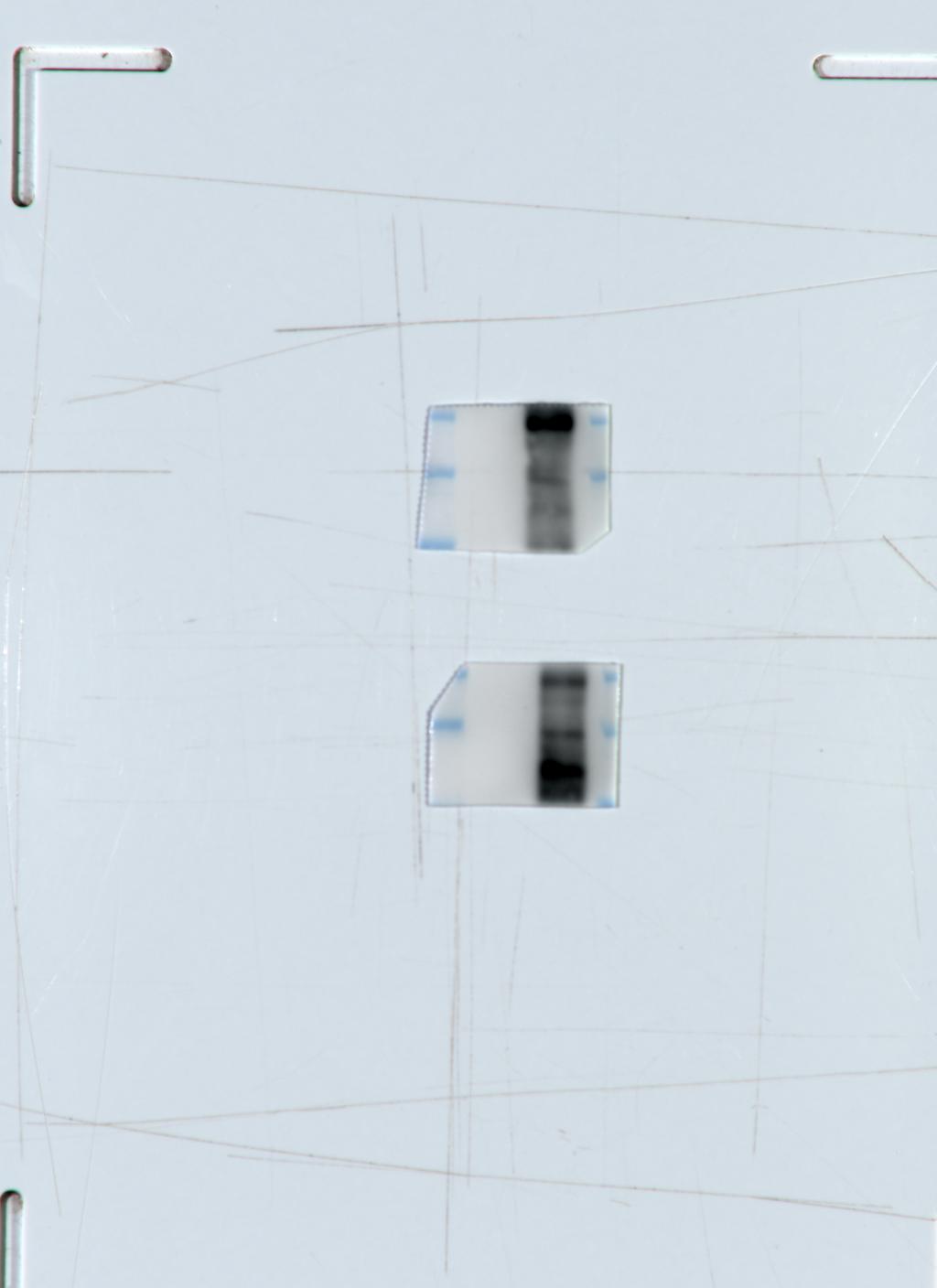

Supplement: Figure 2—figure supplement 1—source data 1. [file elife-76183-fig2-figsupp1-data1.zip › Figure 2-figure supplement 1-source data 1/Figure 2 S1A IP-IRS1.jpg]

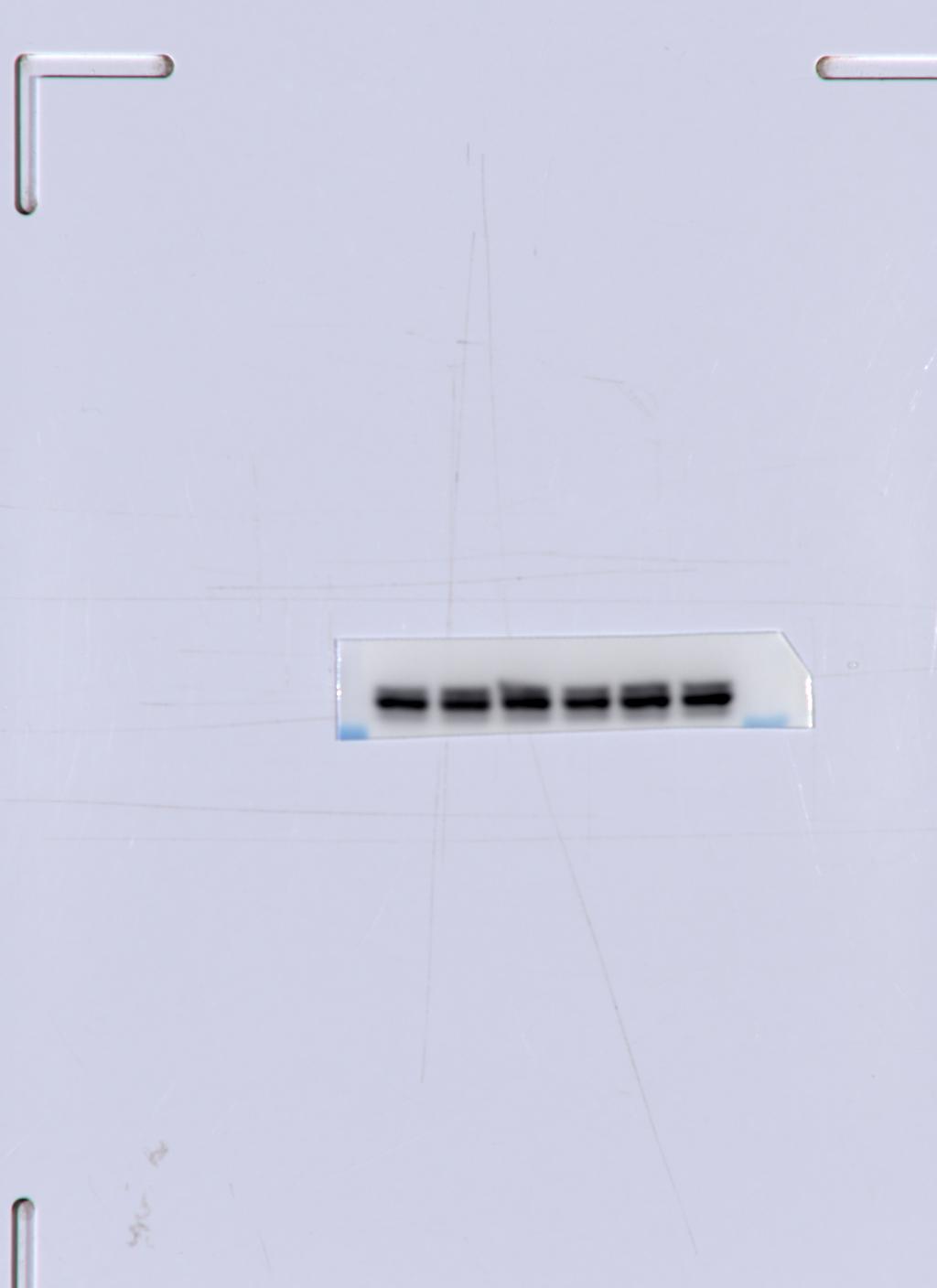

Supplement: Figure 2—figure supplement 1—source data 2. [file elife-76183-fig2-figsupp1-data2.zip › Figure 2-figure supplement 1-source data 2/Figure 2 S1B INPUT-AKT.jpg]

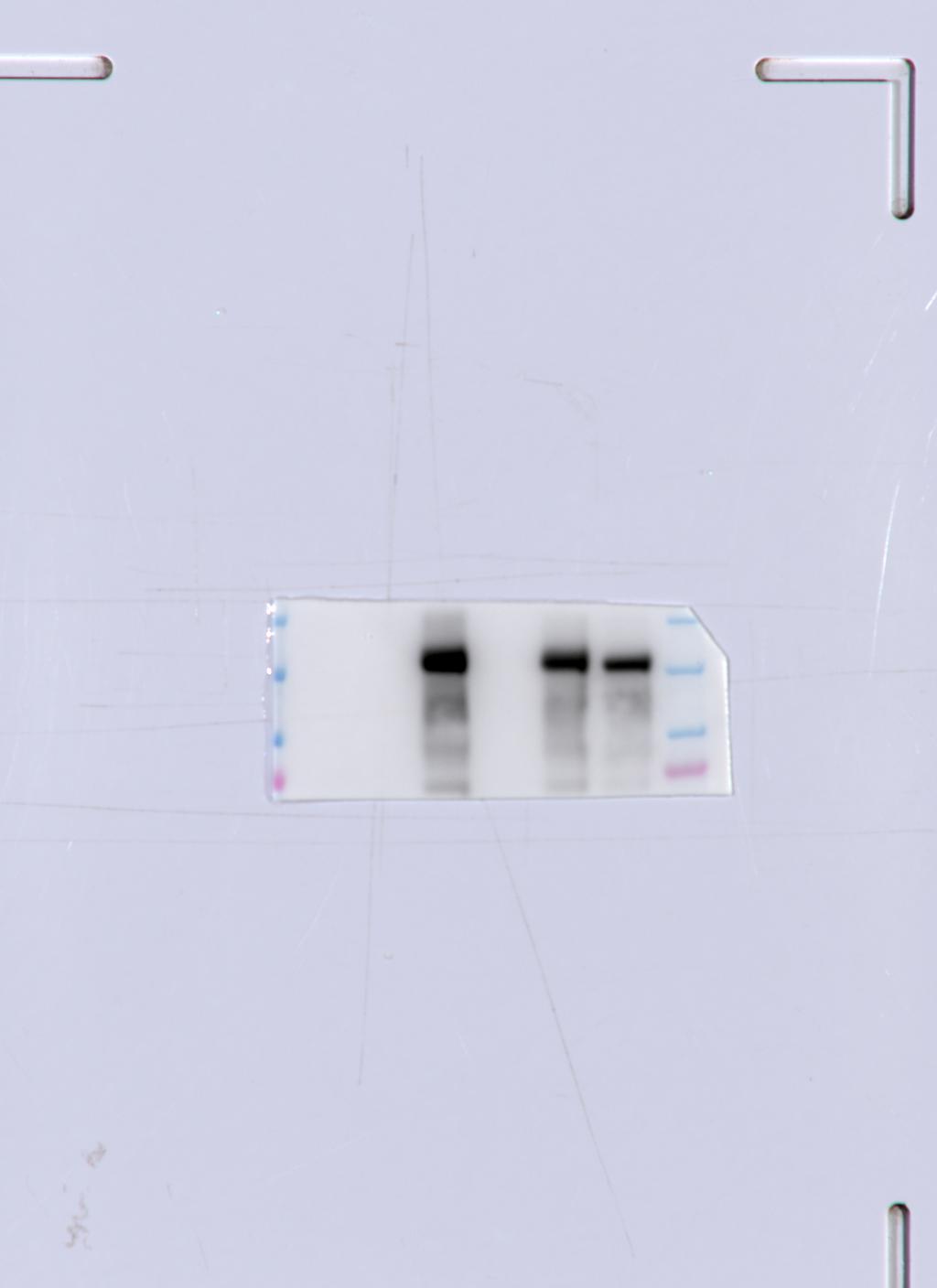

Supplement: Figure 2—figure supplement 1—source data 2. [file elife-76183-fig2-figsupp1-data2.zip › Figure 2-figure supplement 1-source data 2/Figure 2 S1B INPUT-FLAG.jpg]

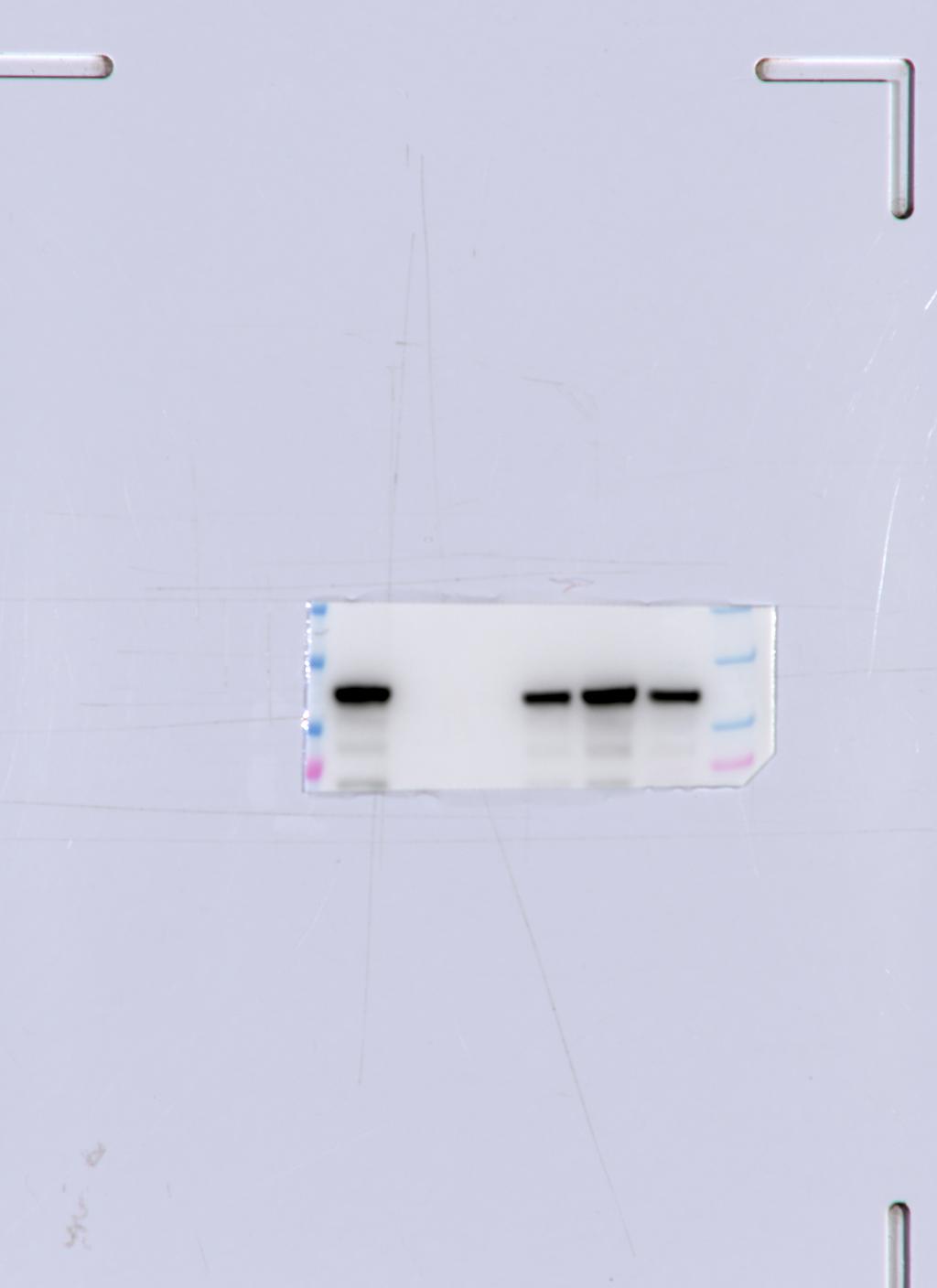

Supplement: Figure 2—figure supplement 1—source data 2. [file elife-76183-fig2-figsupp1-data2.zip › Figure 2-figure supplement 1-source data 2/Figure 2 S1B INPUT-GFP.jpg]

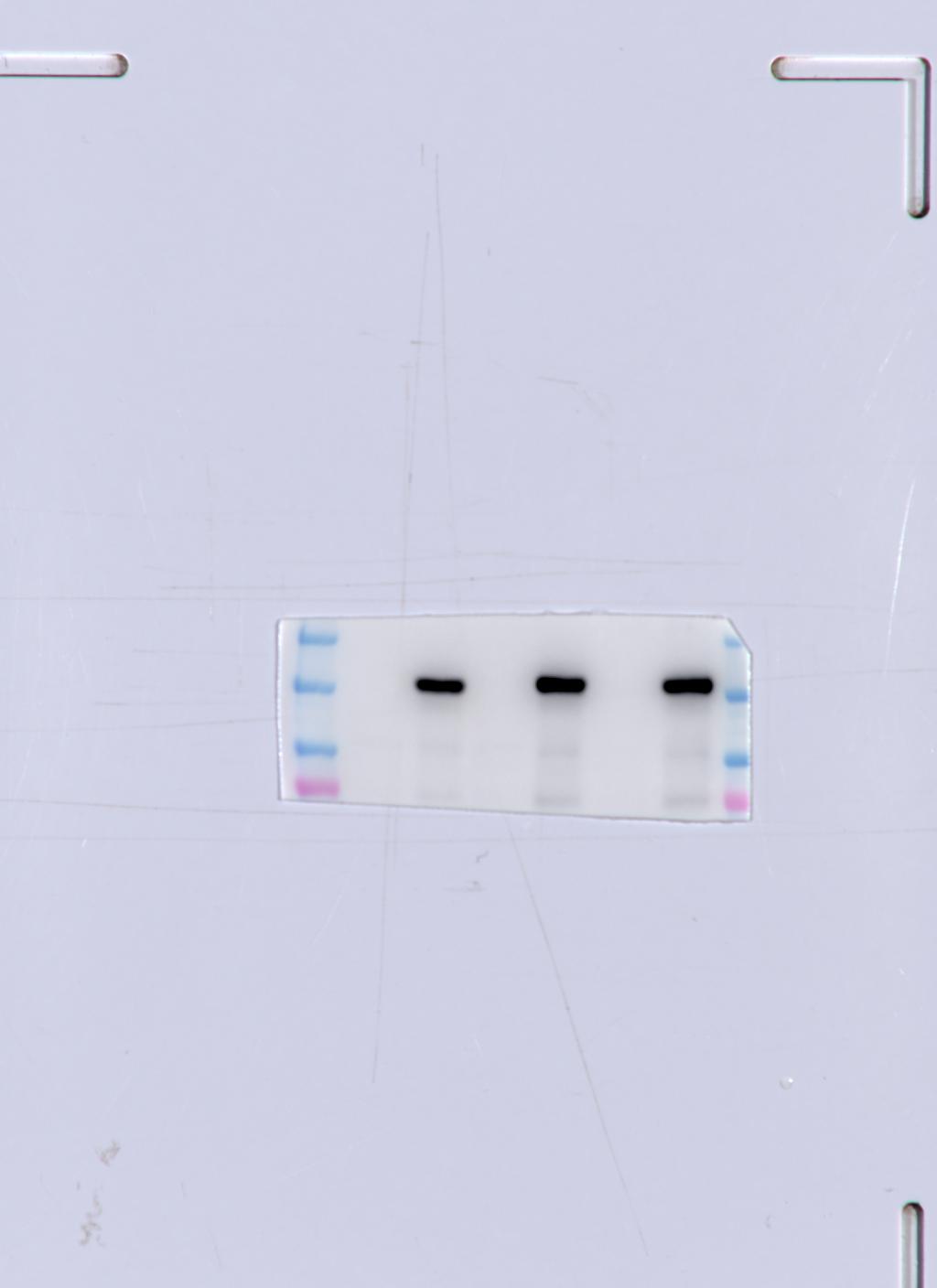

Supplement: Figure 2—figure supplement 1—source data 2. [file elife-76183-fig2-figsupp1-data2.zip › Figure 2-figure supplement 1-source data 2/Figure 2 S1B INPUT-Myc.jpg]

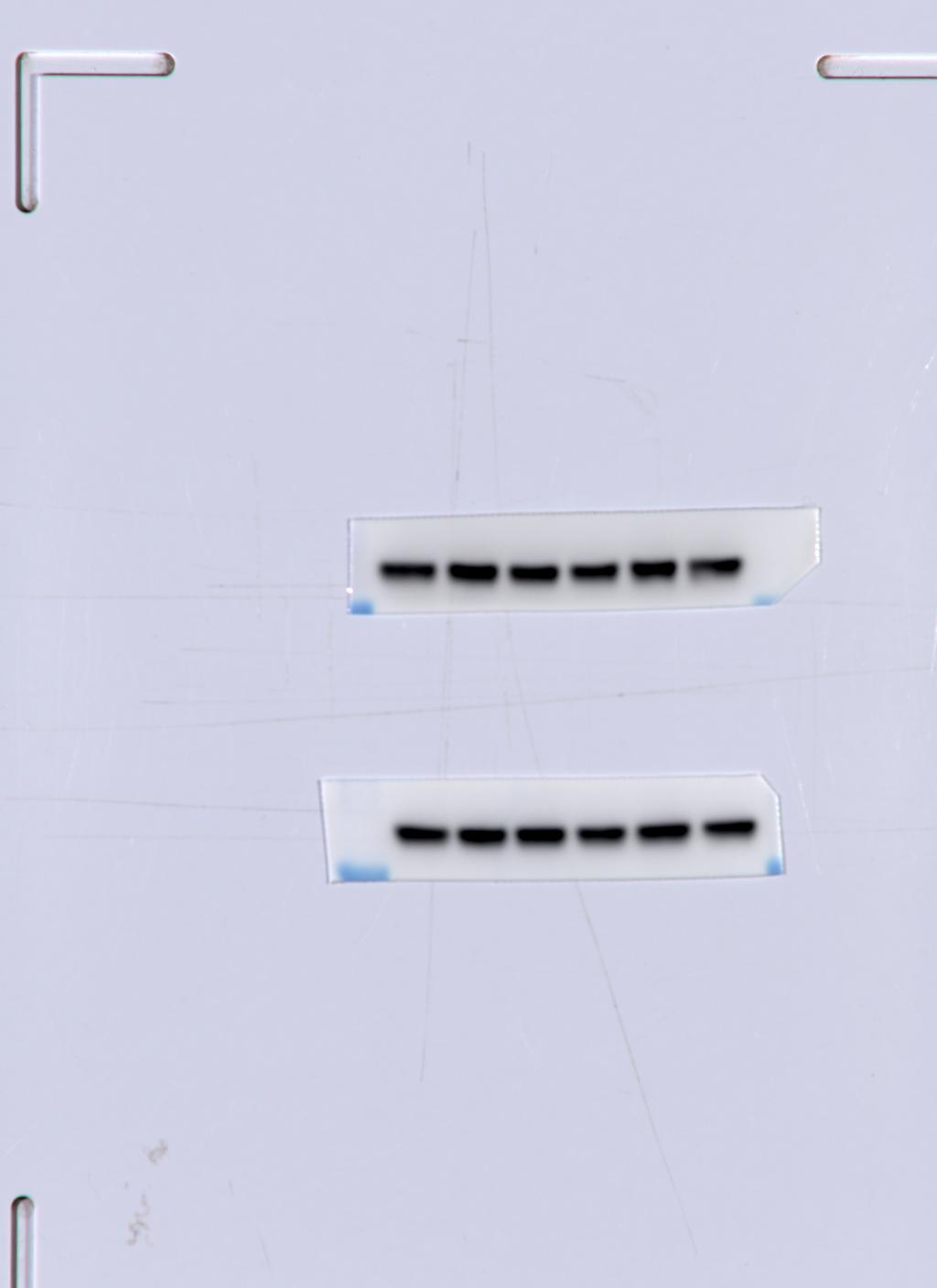

Supplement: Figure 2—figure supplement 1—source data 2. [file elife-76183-fig2-figsupp1-data2.zip › Figure 2-figure supplement 1-source data 2/Figure 2 S1B INPUT-pS473 AKT.jpg]

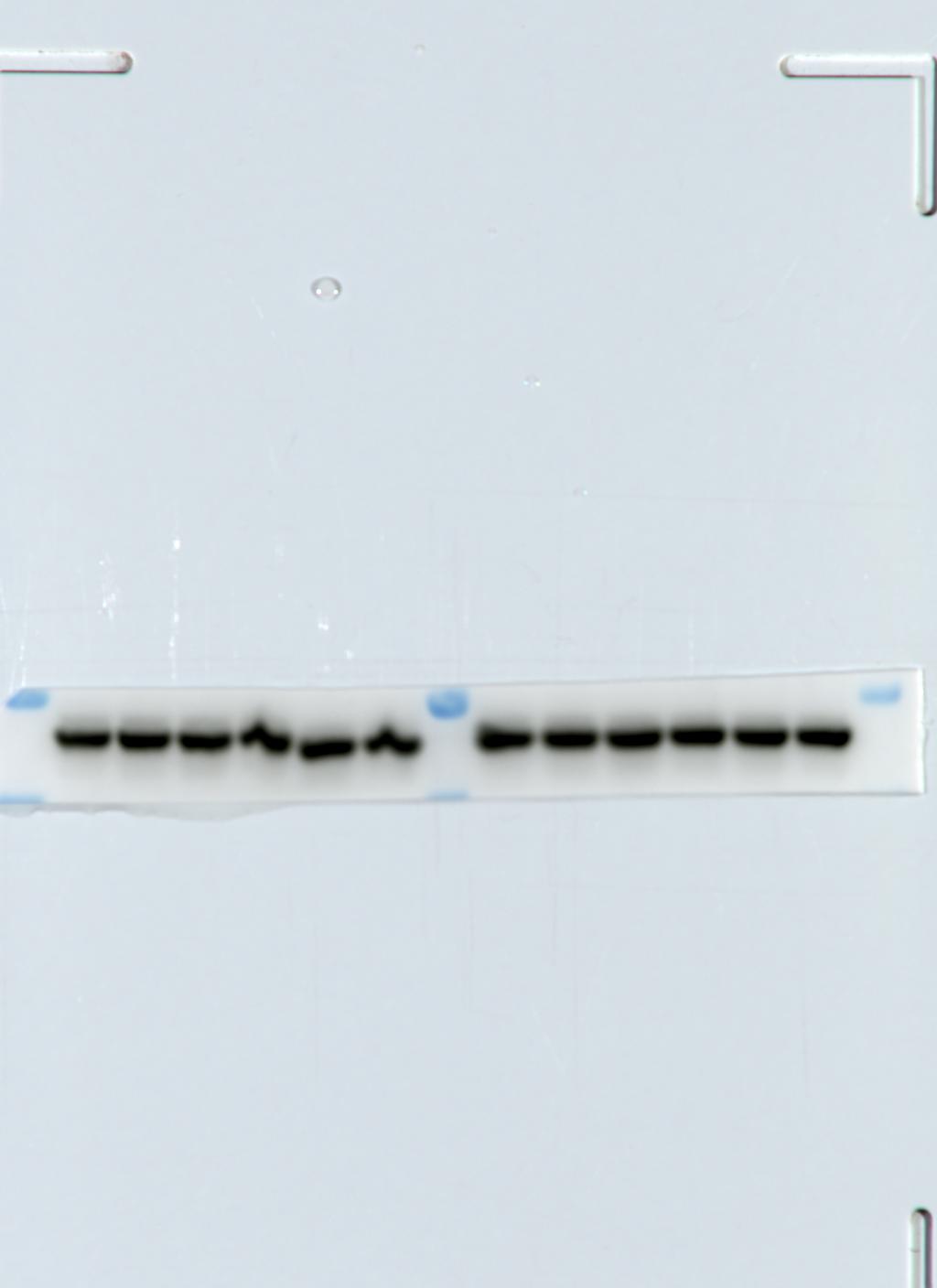

Supplement: Figure 2—figure supplement 1—source data 2. [file elife-76183-fig2-figsupp1-data2.zip › Figure 2-figure supplement 1-source data 2/Figure 2 S1B INPUT-Tubulin.jpg]

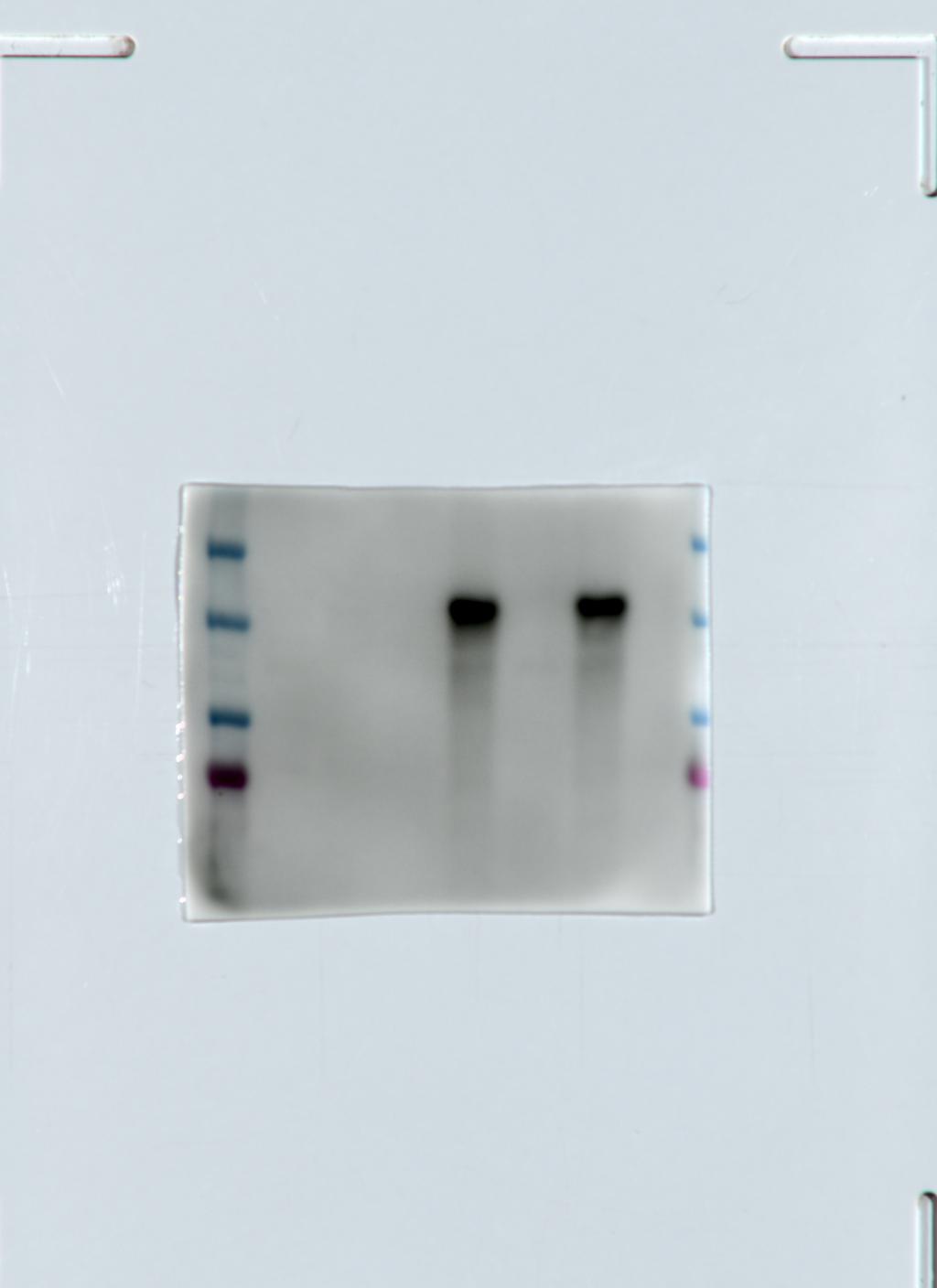

Supplement: Figure 2—figure supplement 1—source data 2. [file elife-76183-fig2-figsupp1-data2.zip › Figure 2-figure supplement 1-source data 2/Figure 2 S1B IP-4G10.jpg]

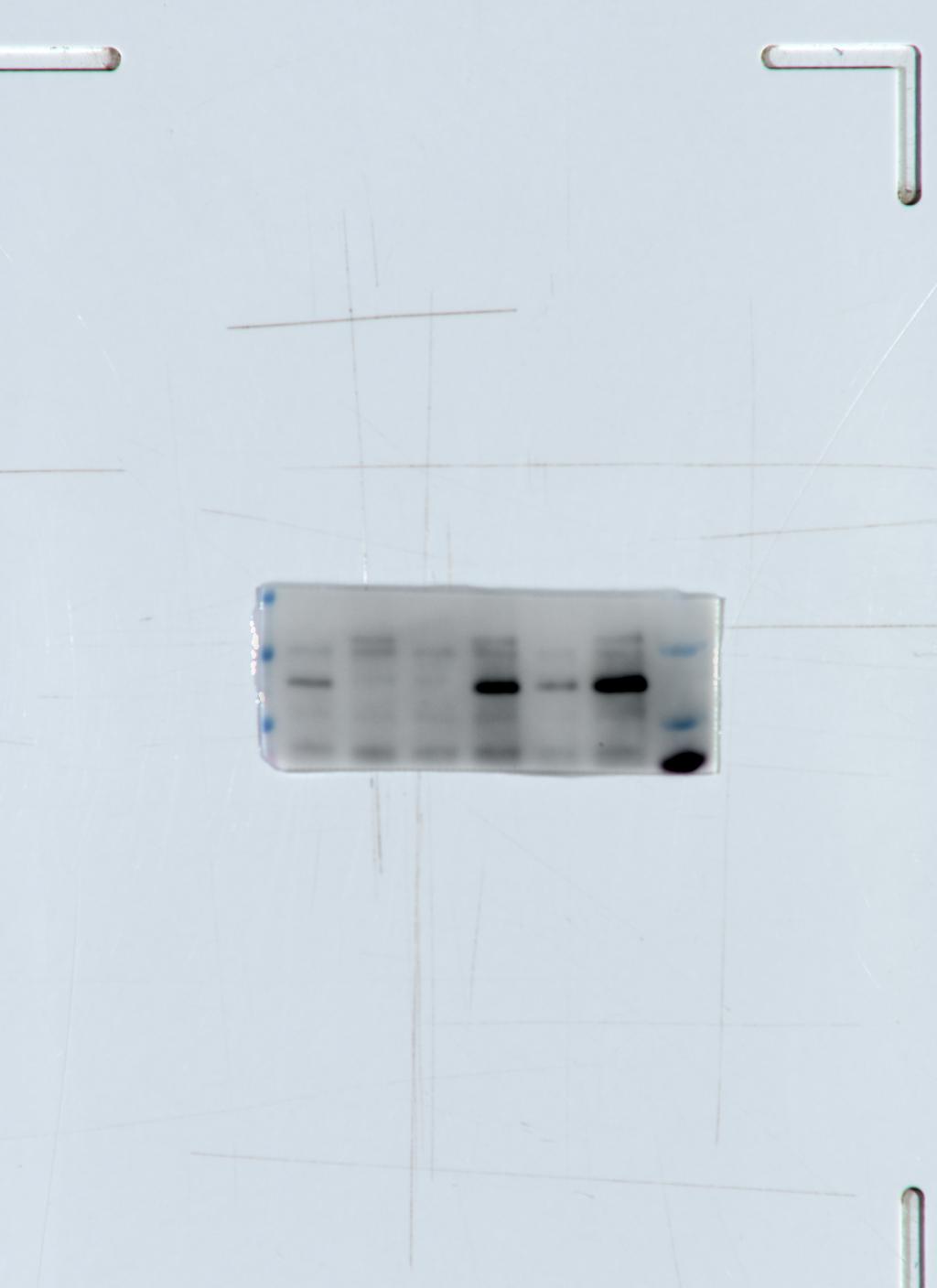

Supplement: Figure 2—figure supplement 1—source data 2. [file elife-76183-fig2-figsupp1-data2.zip › Figure 2-figure supplement 1-source data 2/Figure 2 S1B IP-GFP.jpg]

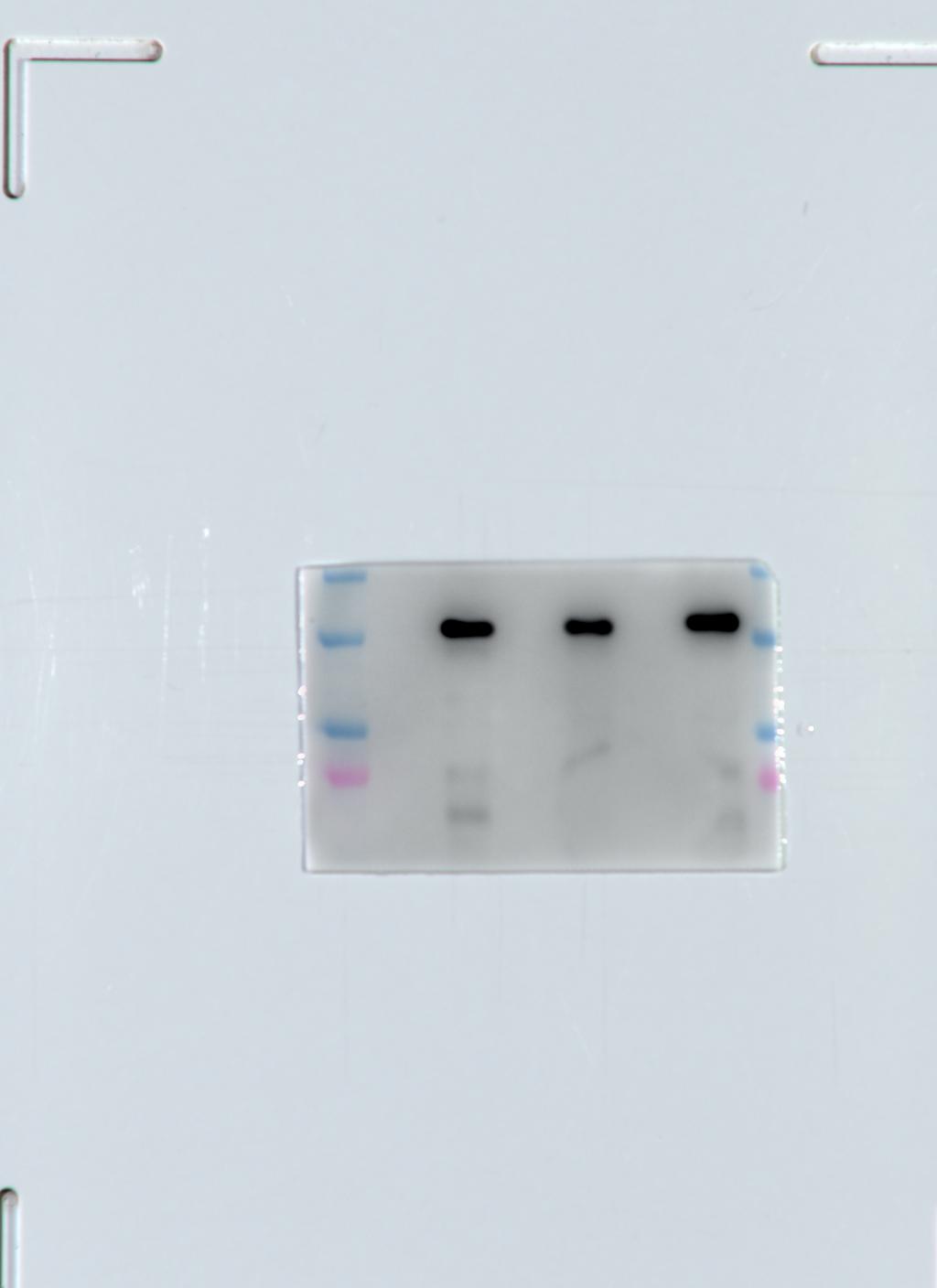

Supplement: Figure 2—figure supplement 1—source data 2. [file elife-76183-fig2-figsupp1-data2.zip › Figure 2-figure supplement 1-source data 2/Figure 2 S1B IP-Myc.jpg]

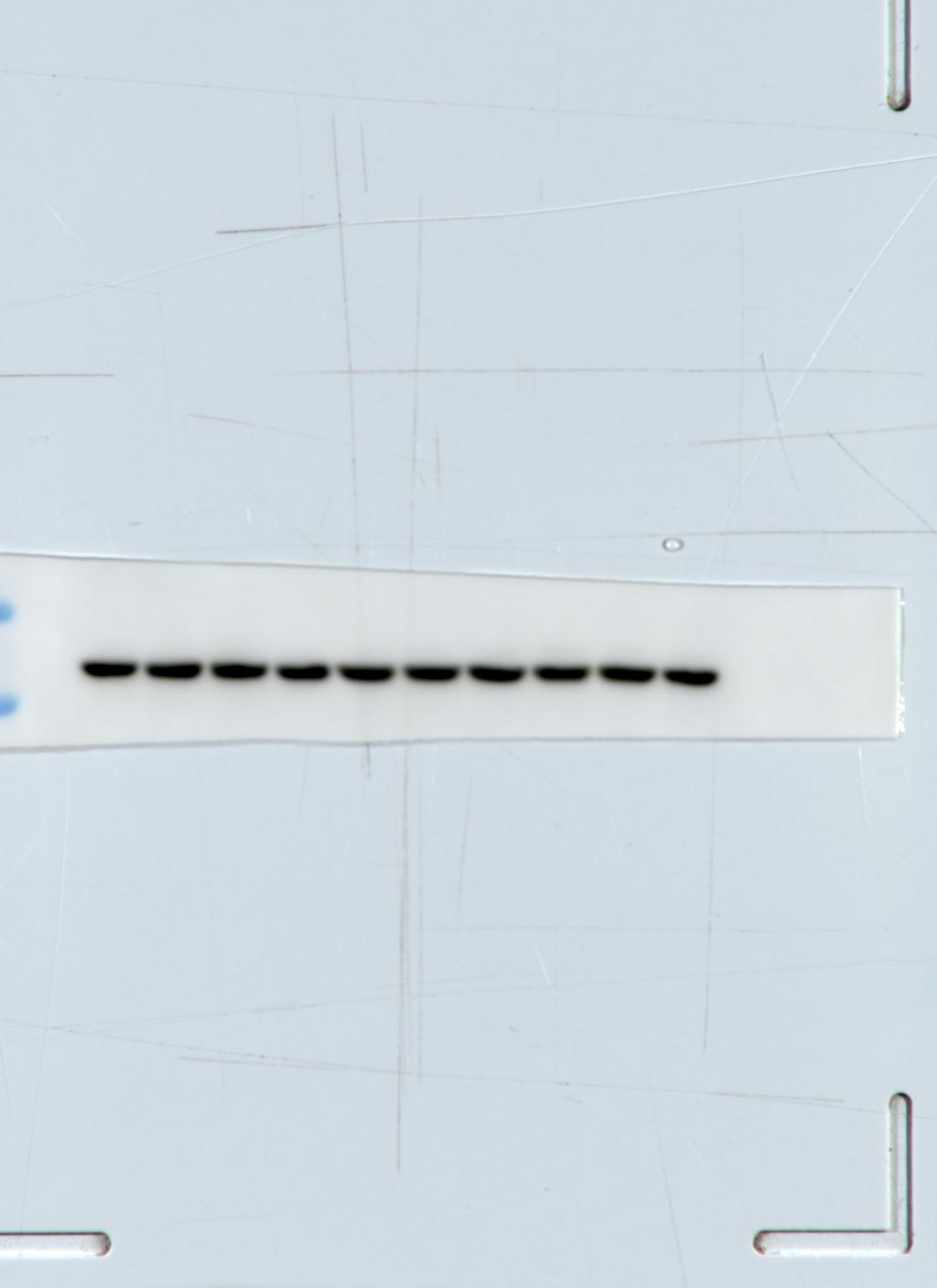

Supplement: Figure 2—figure supplement 2—source data 1. [file elife-76183-fig2-figsupp2-data1.zip › Figure 2-figure supplement 2-source data 1/Figure 2-figure supplement 2A Input Actin.jpg]

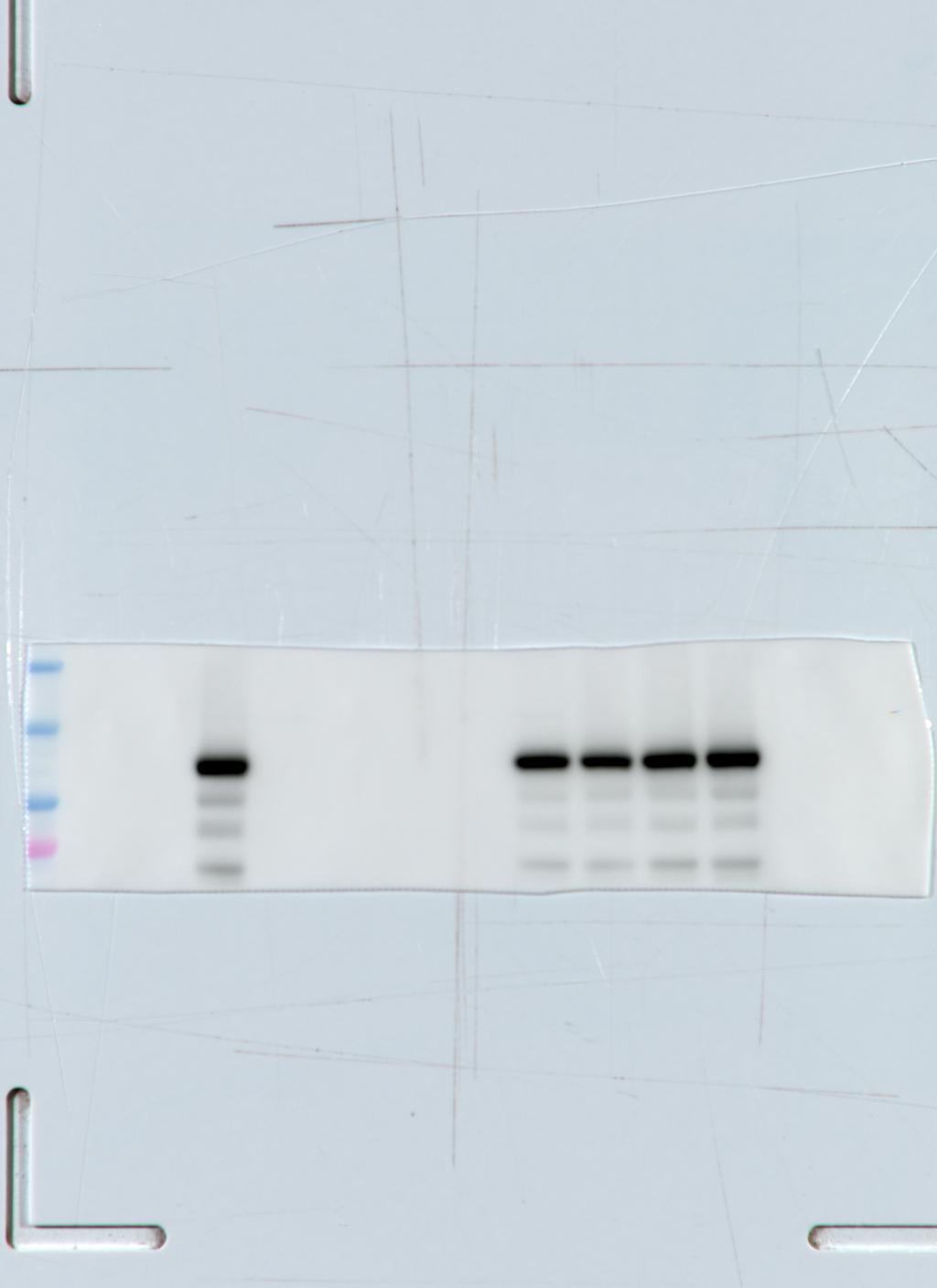

Supplement: Figure 2—figure supplement 2—source data 1. [file elife-76183-fig2-figsupp2-data1.zip › Figure 2-figure supplement 2-source data 1/Figure 2-figure supplement 2A Input WB GFP.jpg]

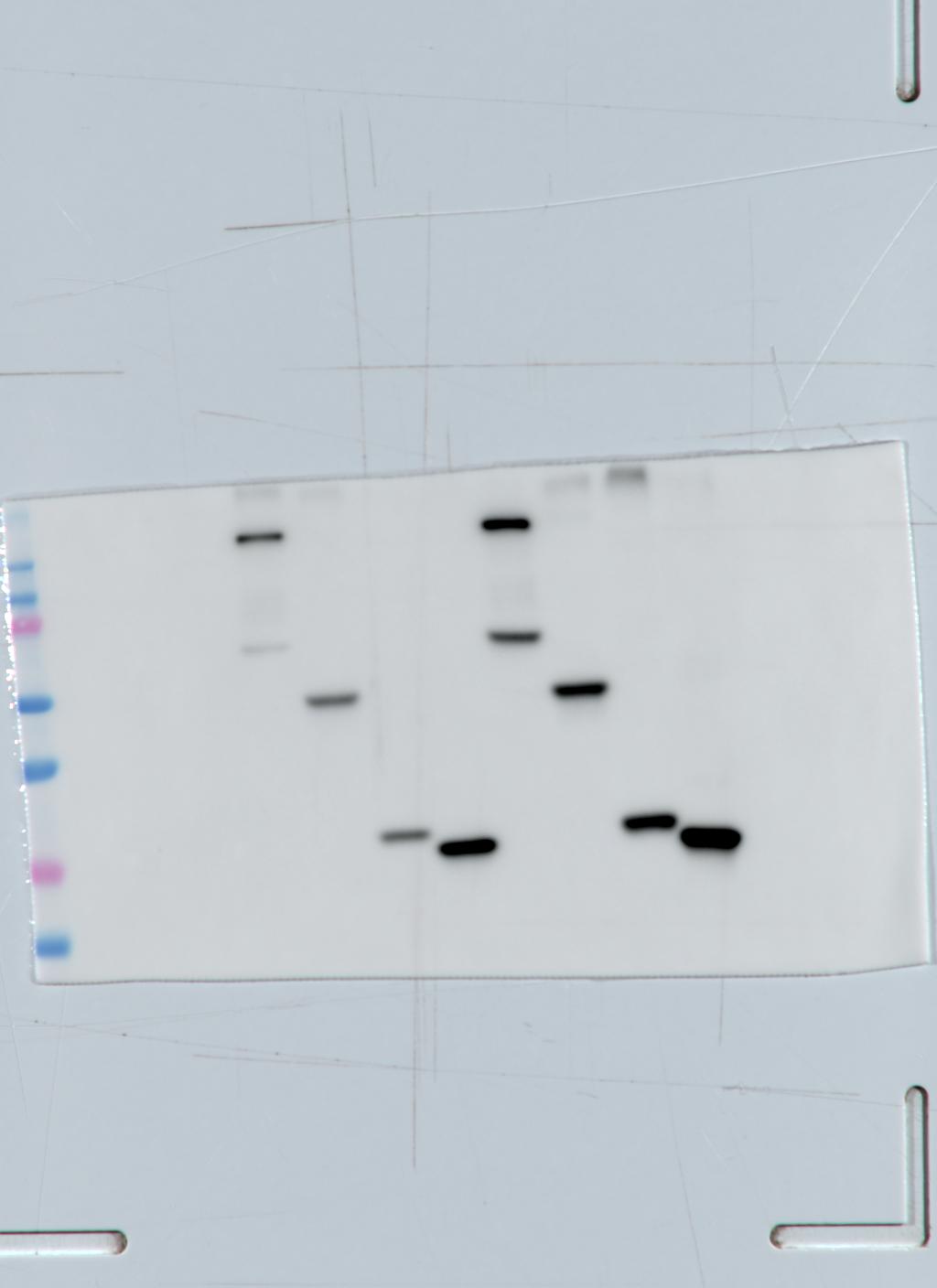

Supplement: Figure 2—figure supplement 2—source data 1. [file elife-76183-fig2-figsupp2-data1.zip › Figure 2-figure supplement 2-source data 1/Figure 2-figure supplement 2A Input WB Myc.jpg]

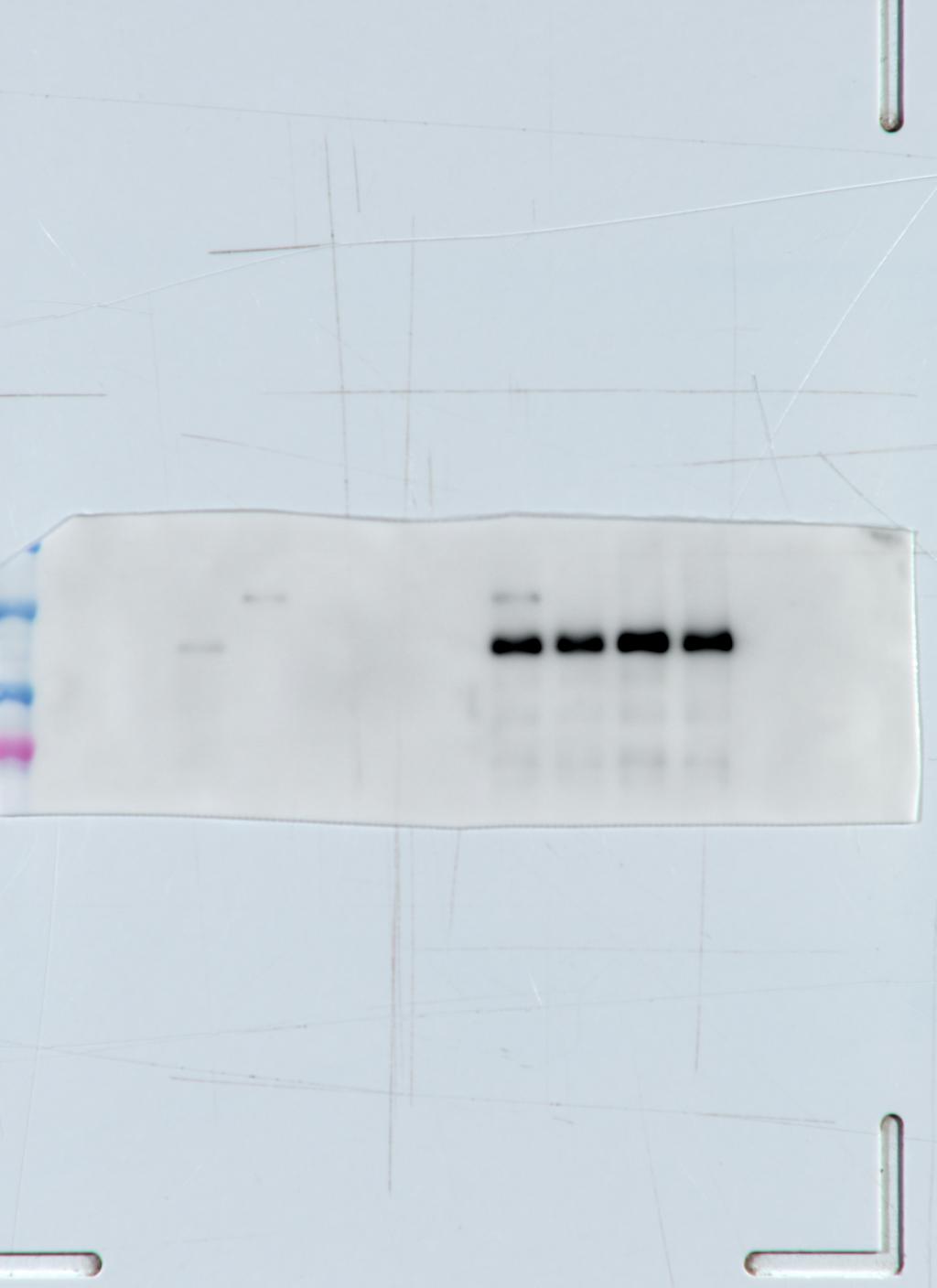

Supplement: Figure 2—figure supplement 2—source data 1. [file elife-76183-fig2-figsupp2-data1.zip › Figure 2-figure supplement 2-source data 1/Figure 2-figure supplement 2A IP Myc WB GFP.jpg]

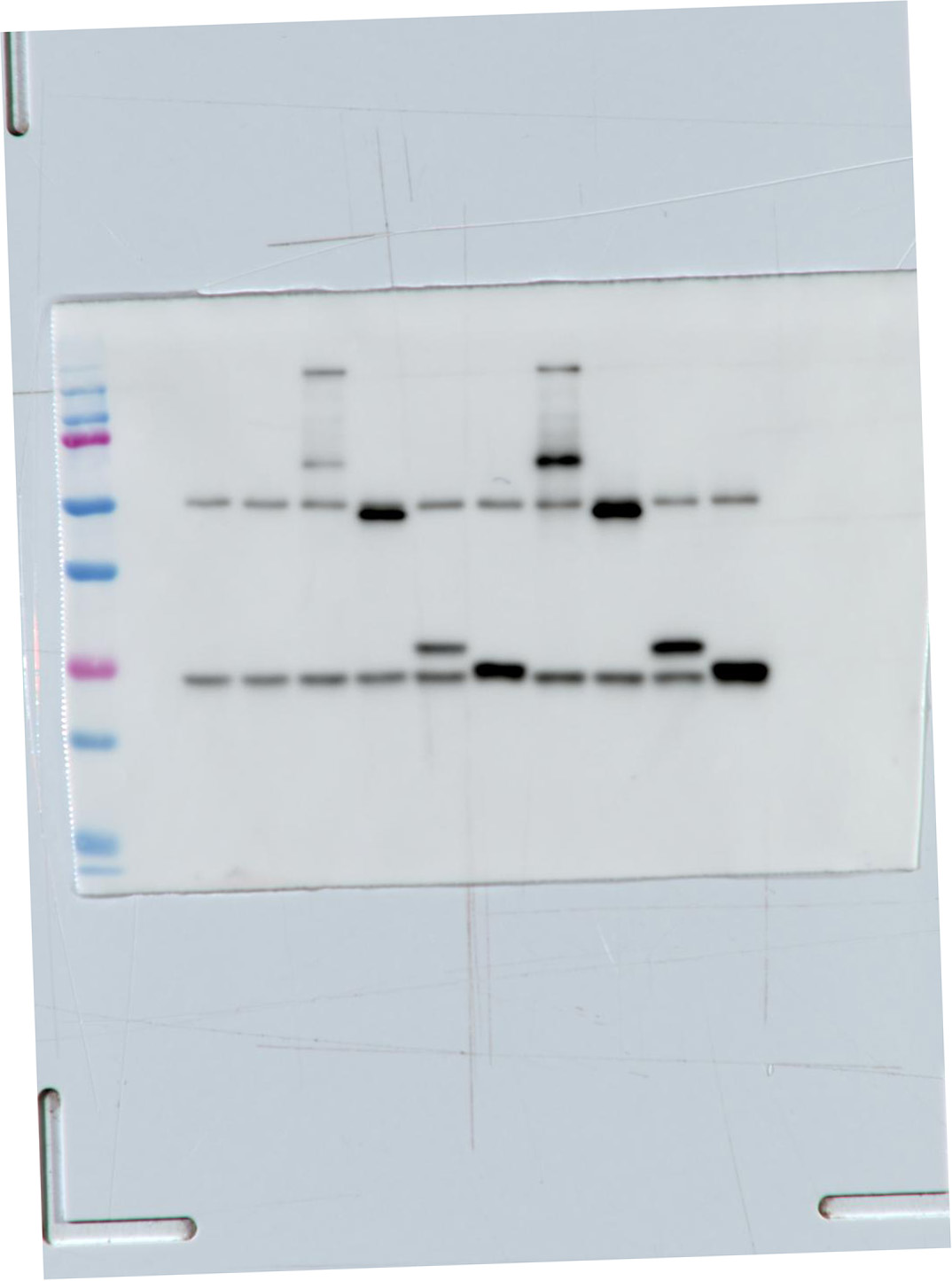

Supplement: Figure 2—figure supplement 2—source data 1. [file elife-76183-fig2-figsupp2-data1.zip › Figure 2-figure supplement 2-source data 1/Figure 2-figure supplement 2A IP Myc WB Myc.jpg]

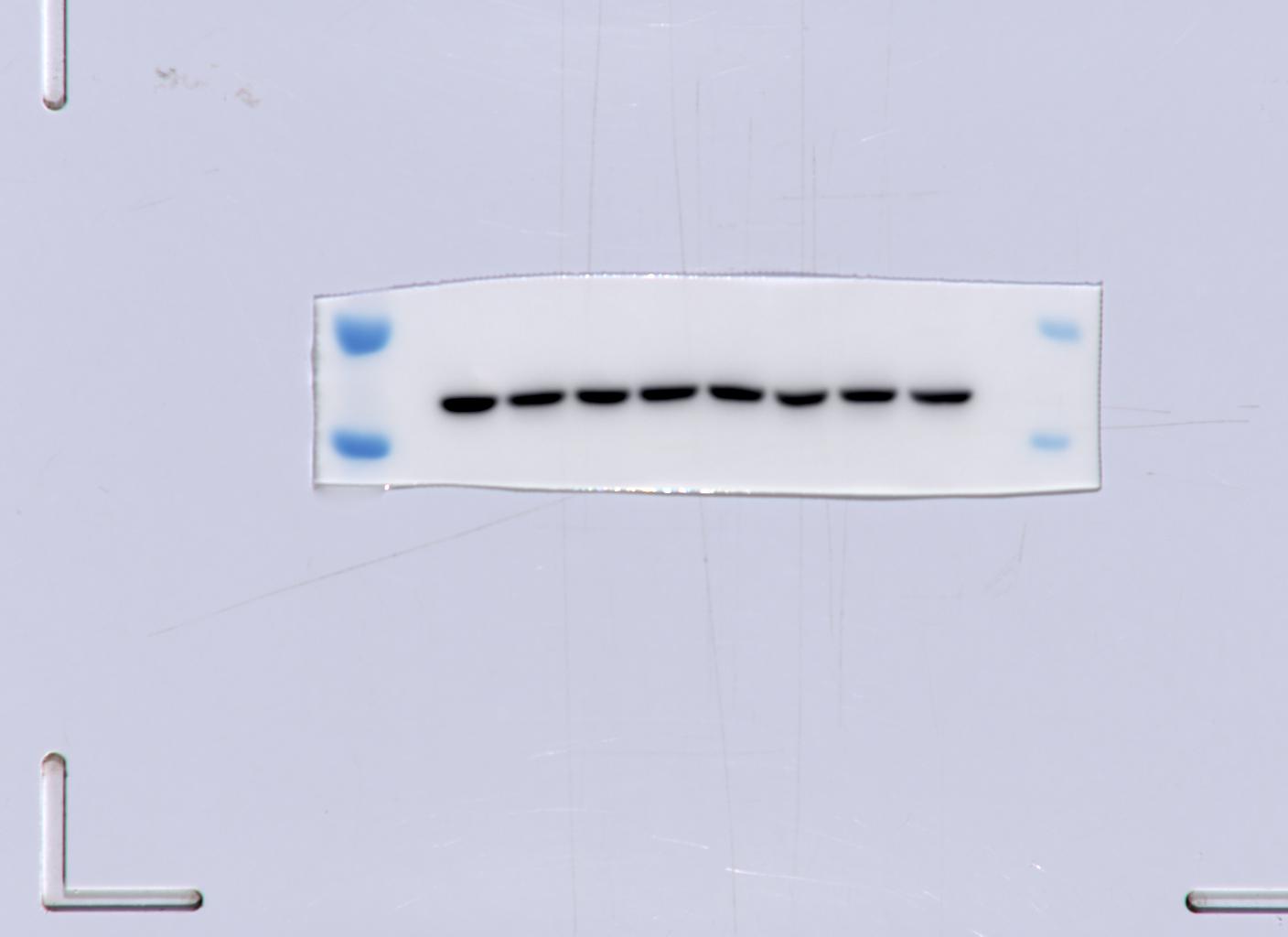

Supplement: Figure 2—figure supplement 2—source data 2. [file elife-76183-fig2-figsupp2-data2.zip › Figure 2-figure supplement 2-source data 2/Figure 2-figure supplement 2B Input Actin.jpg]

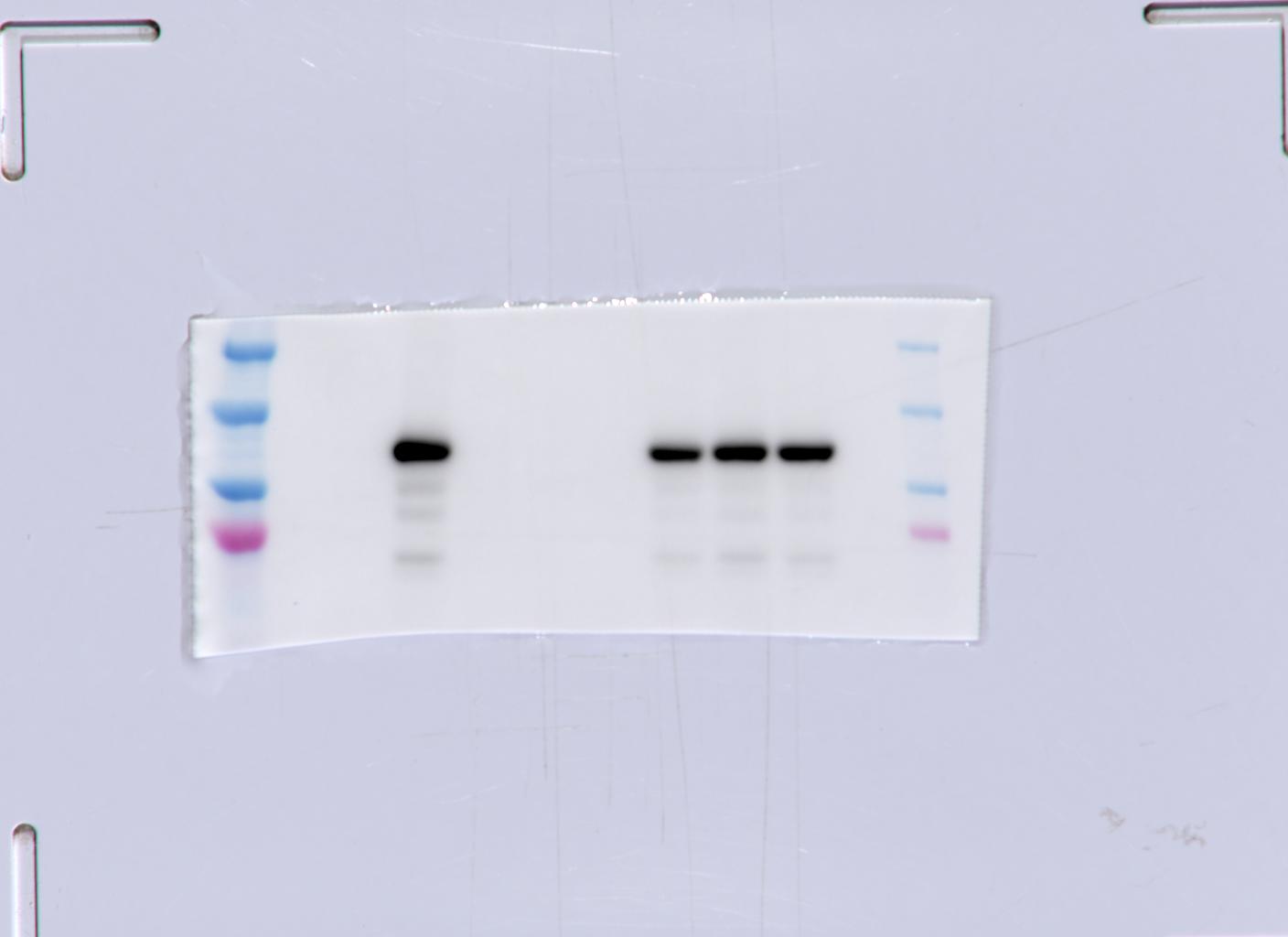

Supplement: Figure 2—figure supplement 2—source data 2. [file elife-76183-fig2-figsupp2-data2.zip › Figure 2-figure supplement 2-source data 2/Figure 2-figure supplement 2B Input WB GFP.jpg]

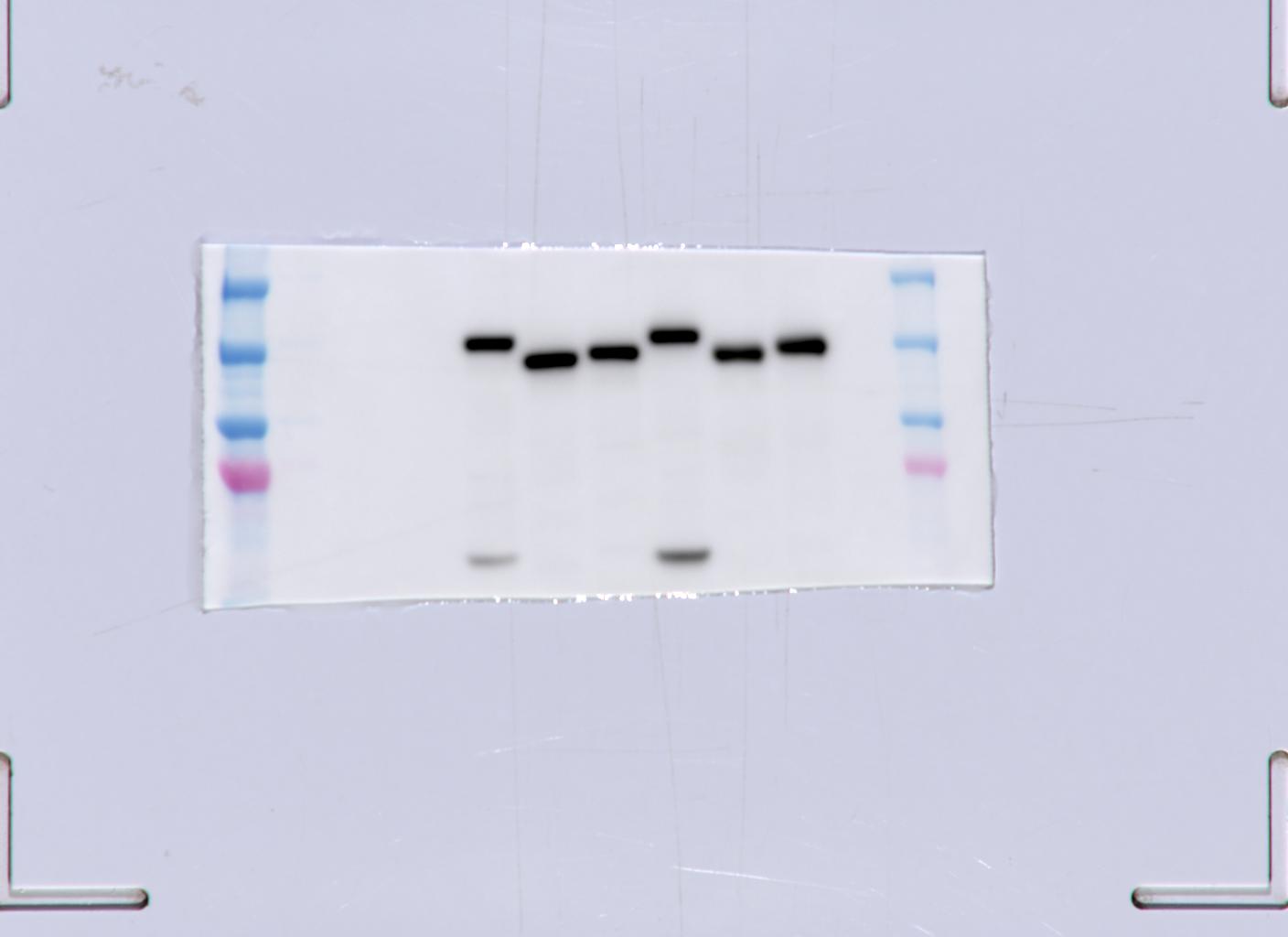

Supplement: Figure 2—figure supplement 2—source data 2. [file elife-76183-fig2-figsupp2-data2.zip › Figure 2-figure supplement 2-source data 2/Figure 2-figure supplement 2B Input WB Myc.jpg]

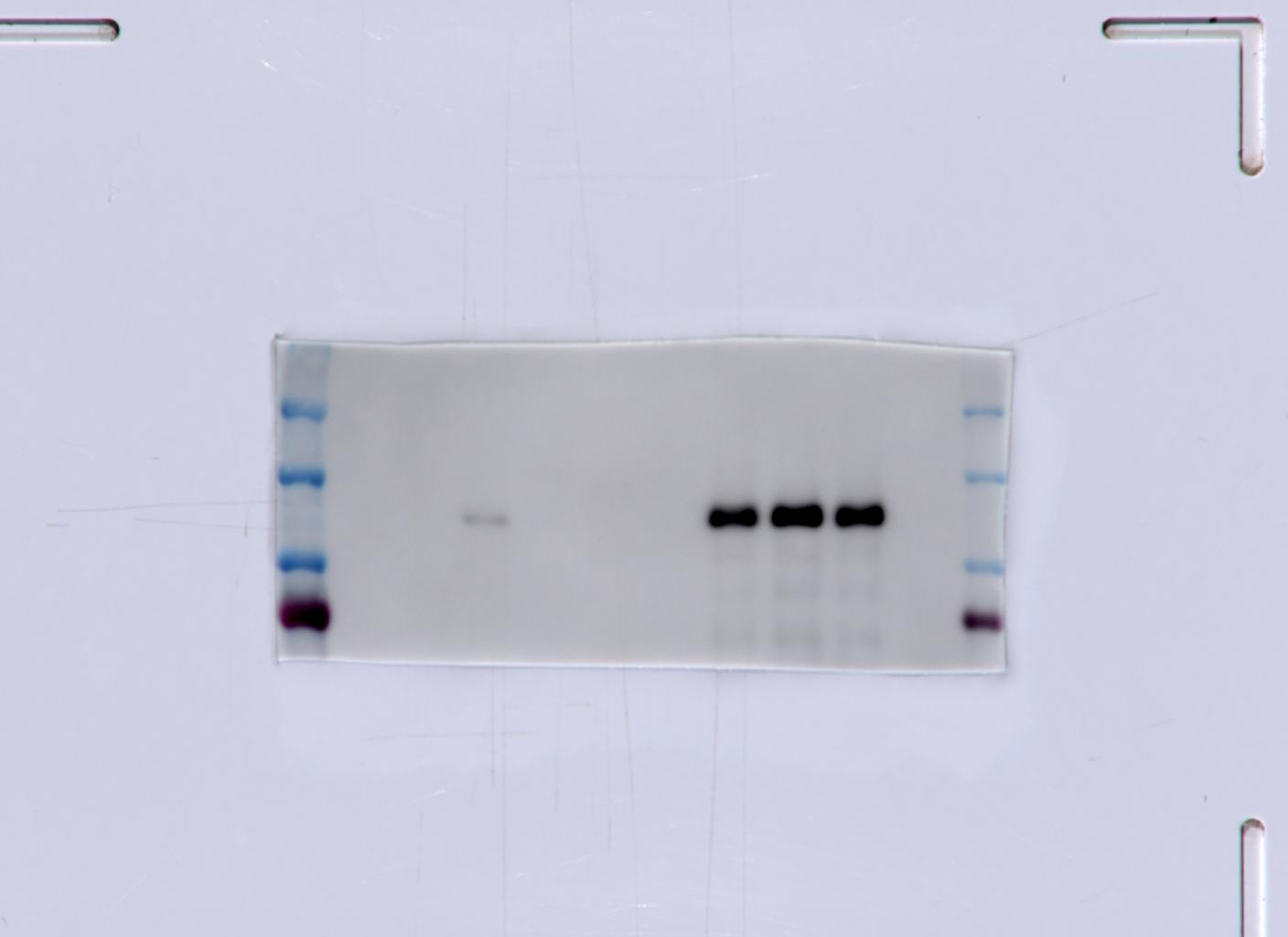

Supplement: Figure 2—figure supplement 2—source data 2. [file elife-76183-fig2-figsupp2-data2.zip › Figure 2-figure supplement 2-source data 2/Figure 2-figure supplement 2B IP Myc WB GFP.jpg]

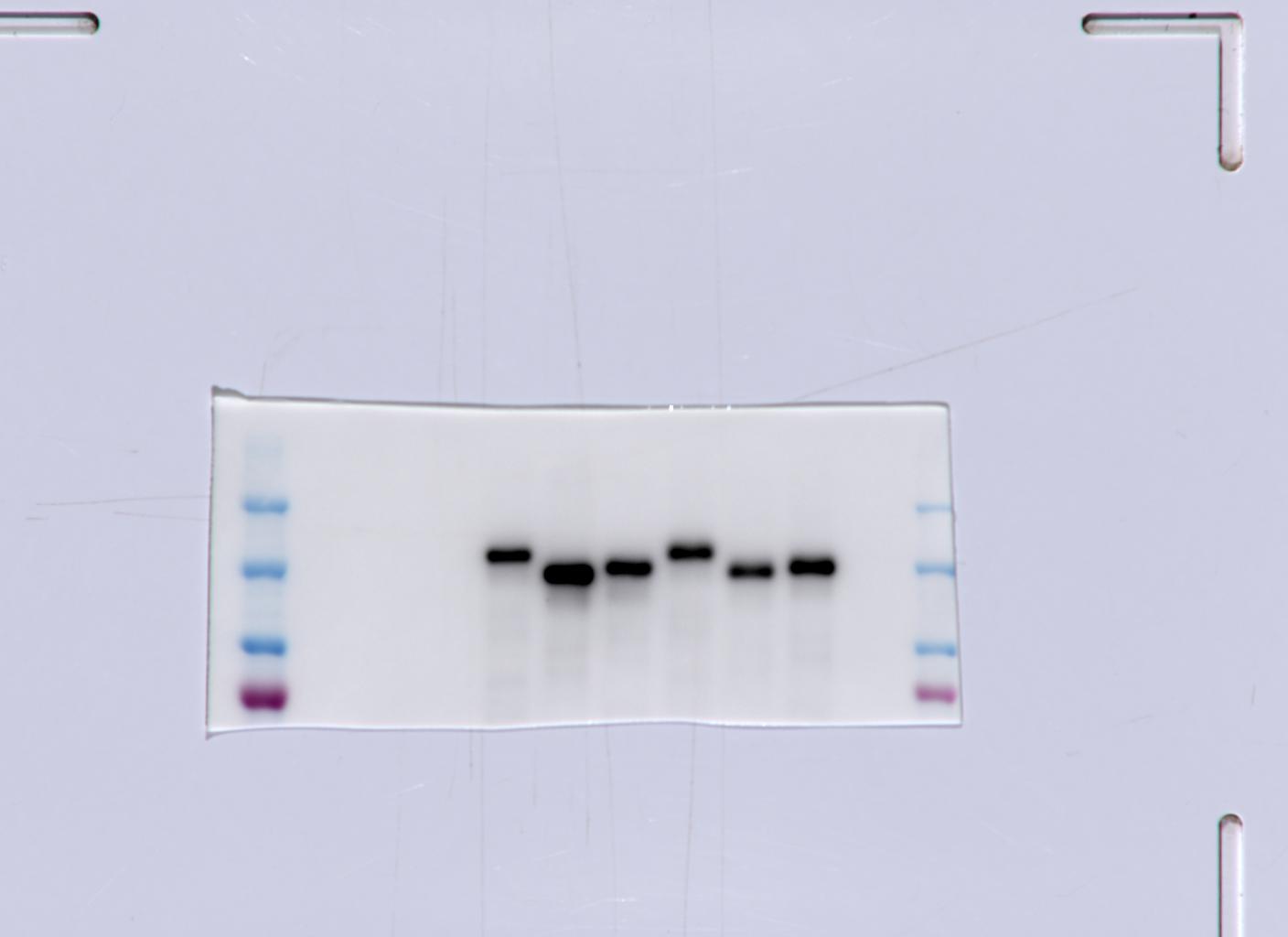

Supplement: Figure 2—figure supplement 2—source data 2. [file elife-76183-fig2-figsupp2-data2.zip › Figure 2-figure supplement 2-source data 2/Figure 2-figure supplement 2B IP Myc WB Myc.jpg]

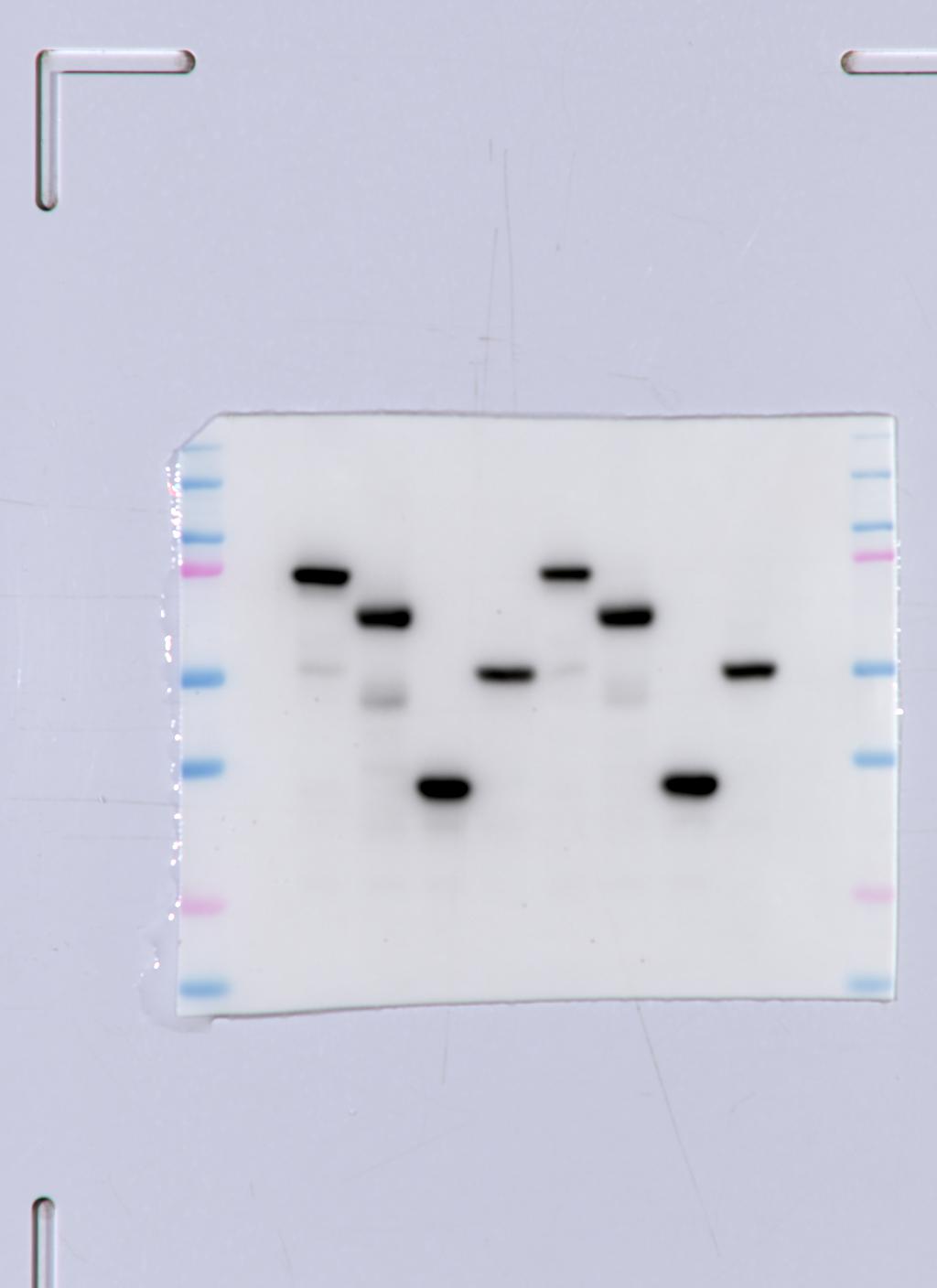

Supplement: Figure 2—figure supplement 2—source data 3. [file elife-76183-fig2-figsupp2-data3.zip › Figure 2-figure supplement 2-source data 3/Figure 2 S2C INPUT-GFP.jpg]

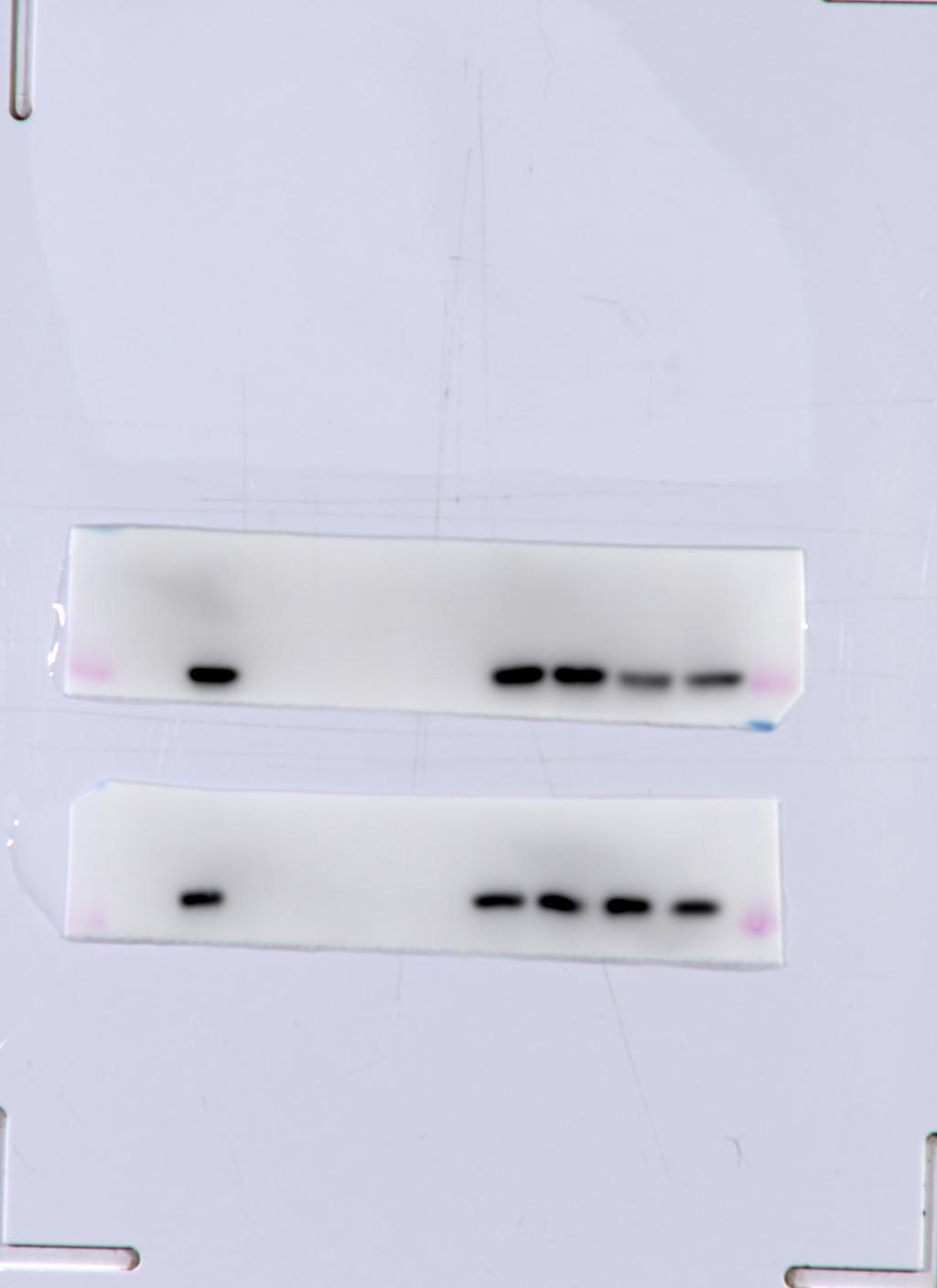

Supplement: Figure 2—figure supplement 2—source data 3. [file elife-76183-fig2-figsupp2-data3.zip › Figure 2-figure supplement 2-source data 3/Figure 2 S2C INPUT-Myc.jpg]

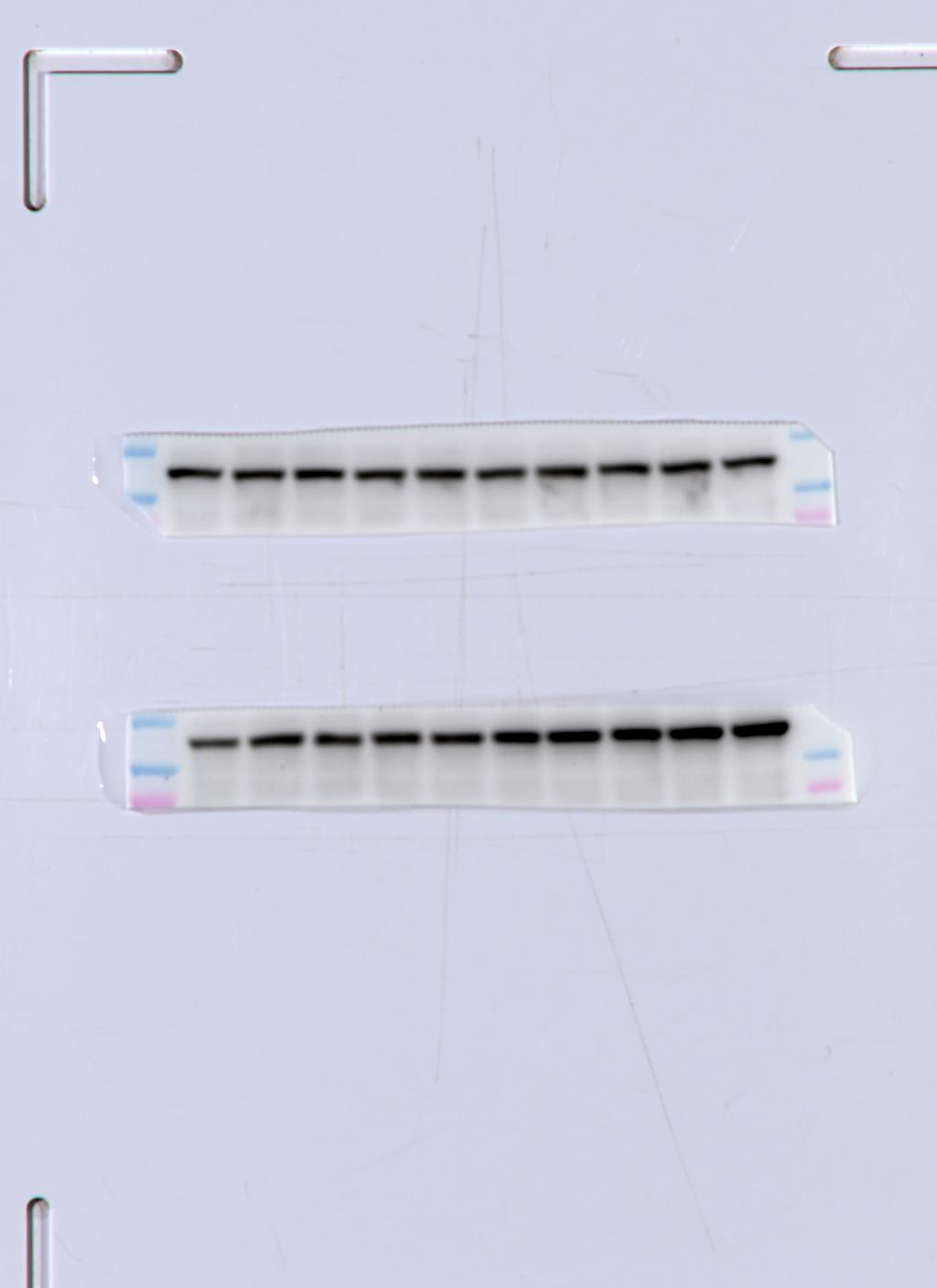

Supplement: Figure 2—figure supplement 2—source data 3. [file elife-76183-fig2-figsupp2-data3.zip › Figure 2-figure supplement 2-source data 3/Figure 2 S2C INPUT-Vinculin.jpg]

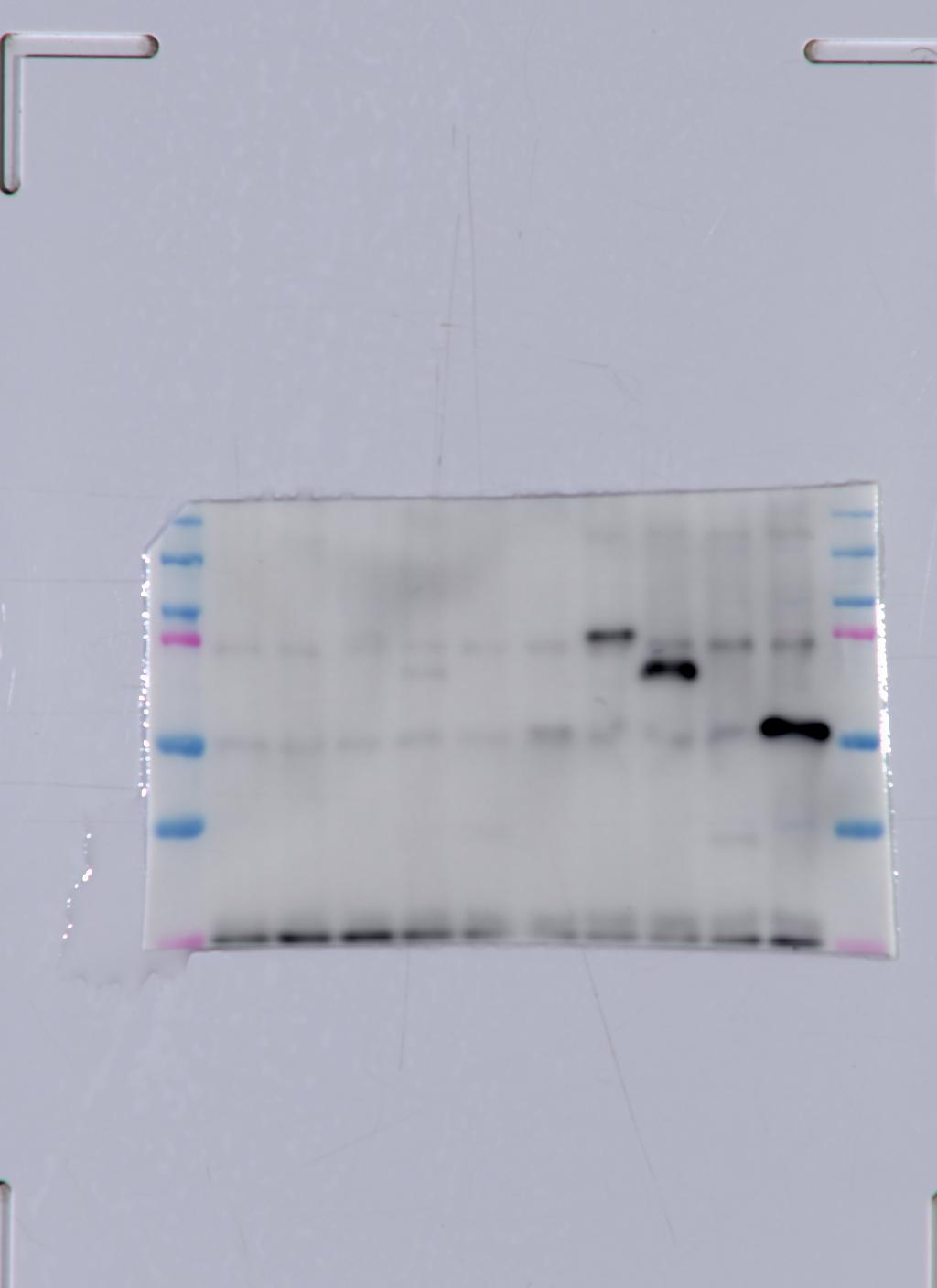

Supplement: Figure 2—figure supplement 2—source data 3. [file elife-76183-fig2-figsupp2-data3.zip › Figure 2-figure supplement 2-source data 3/Figure 2 S2C IP-GFP.jpg]

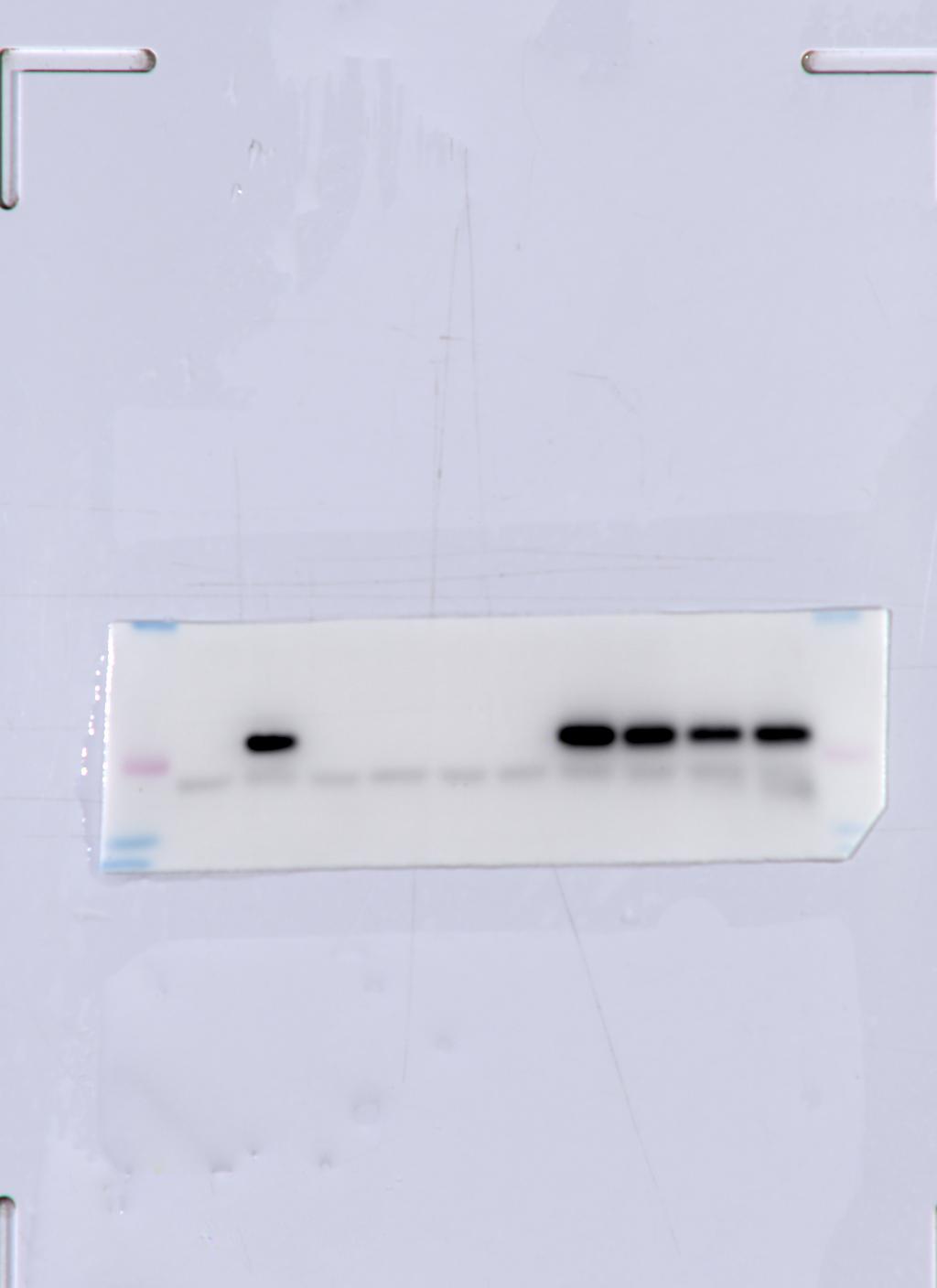

Supplement: Figure 2—figure supplement 2—source data 3. [file elife-76183-fig2-figsupp2-data3.zip › Figure 2-figure supplement 2-source data 3/Figure 2 S2C IP-Myc.jpg]

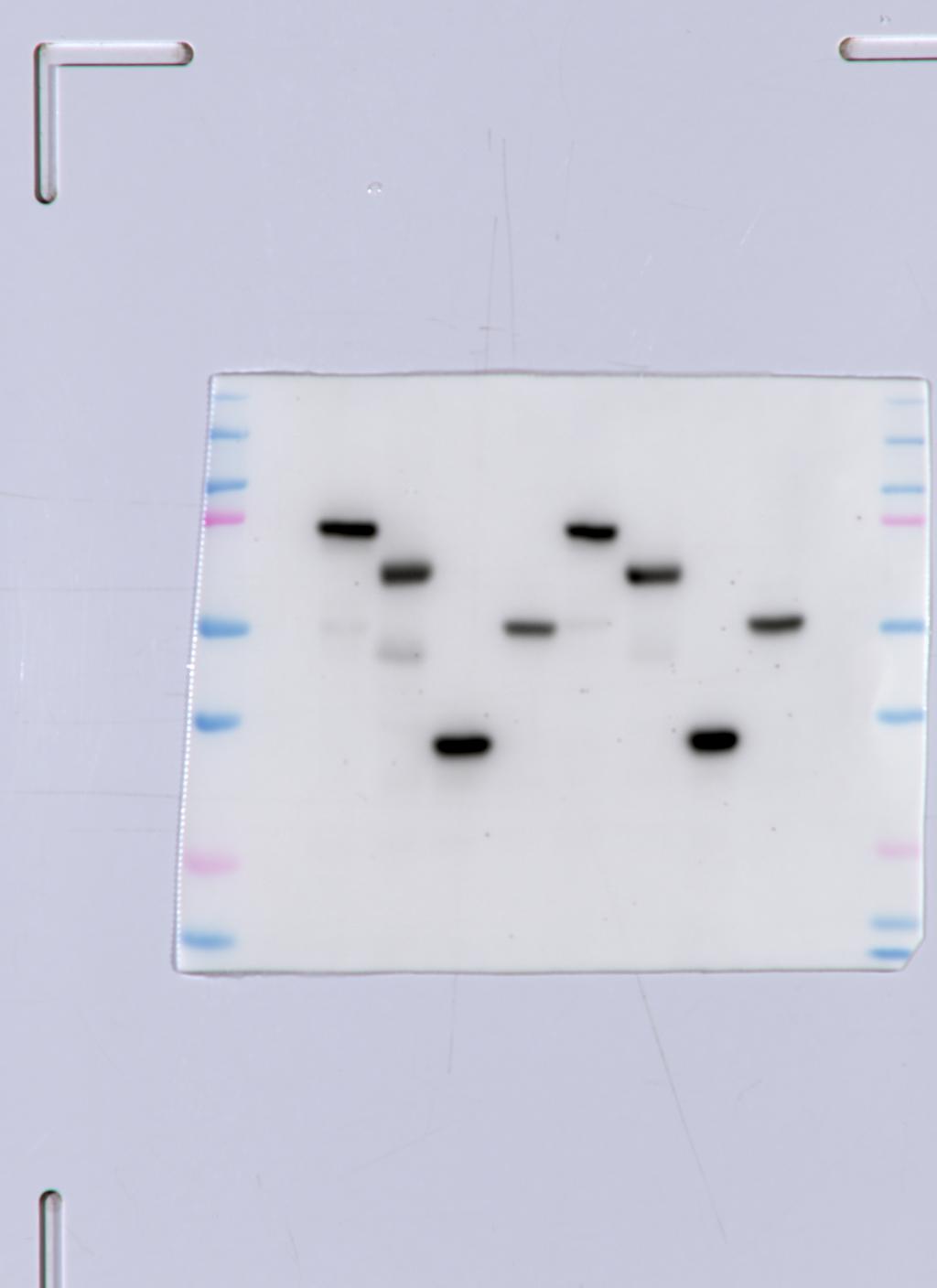

Supplement: Figure 2—figure supplement 2—source data 4. [file elife-76183-fig2-figsupp2-data4.zip › Figure 2-figure supplement 2-source data 4/Figure 2 S2D INPUT-GFP.jpg]

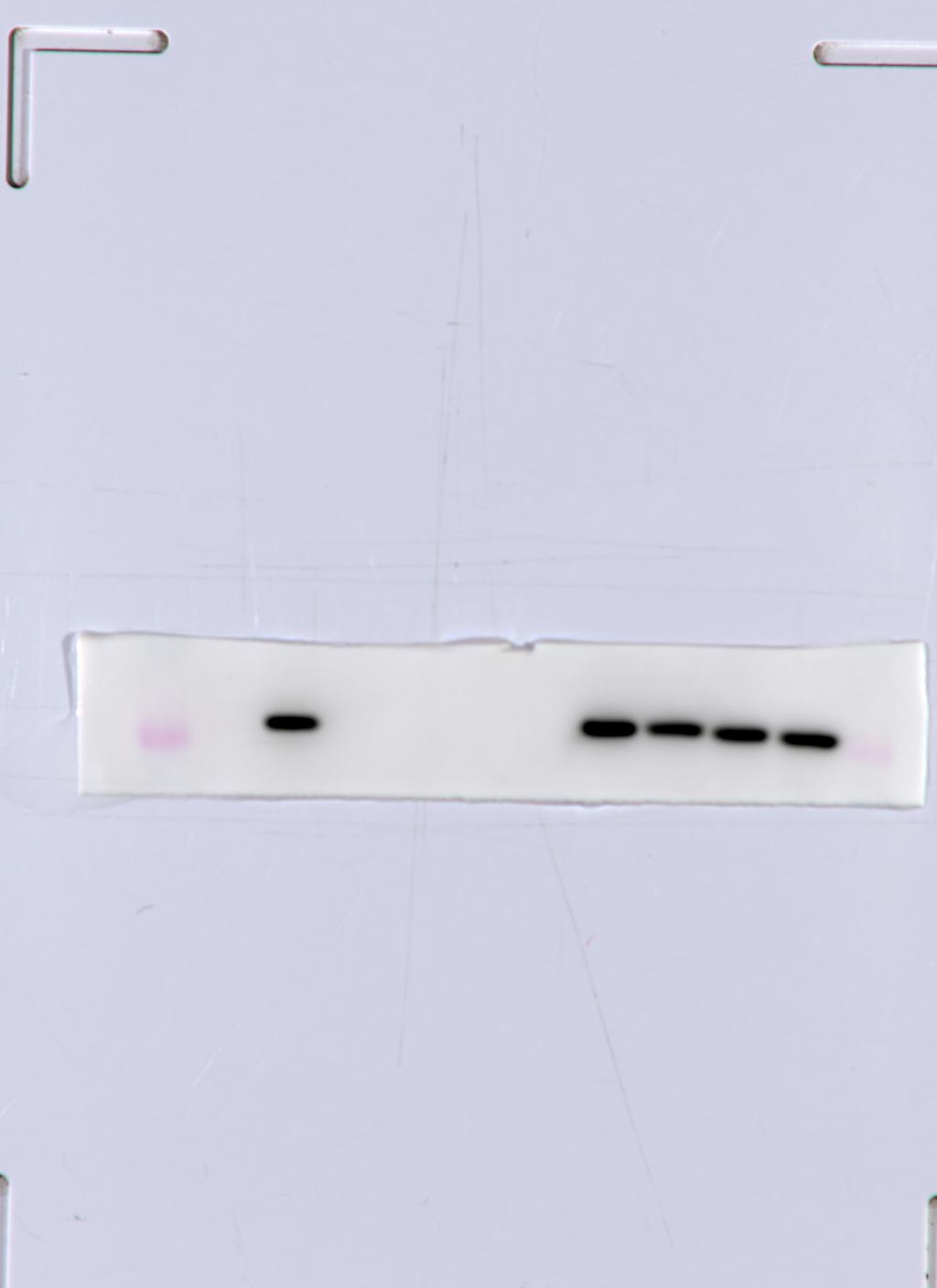

Supplement: Figure 2—figure supplement 2—source data 4. [file elife-76183-fig2-figsupp2-data4.zip › Figure 2-figure supplement 2-source data 4/Figure 2 S2D INPUT-Myc.jpg]

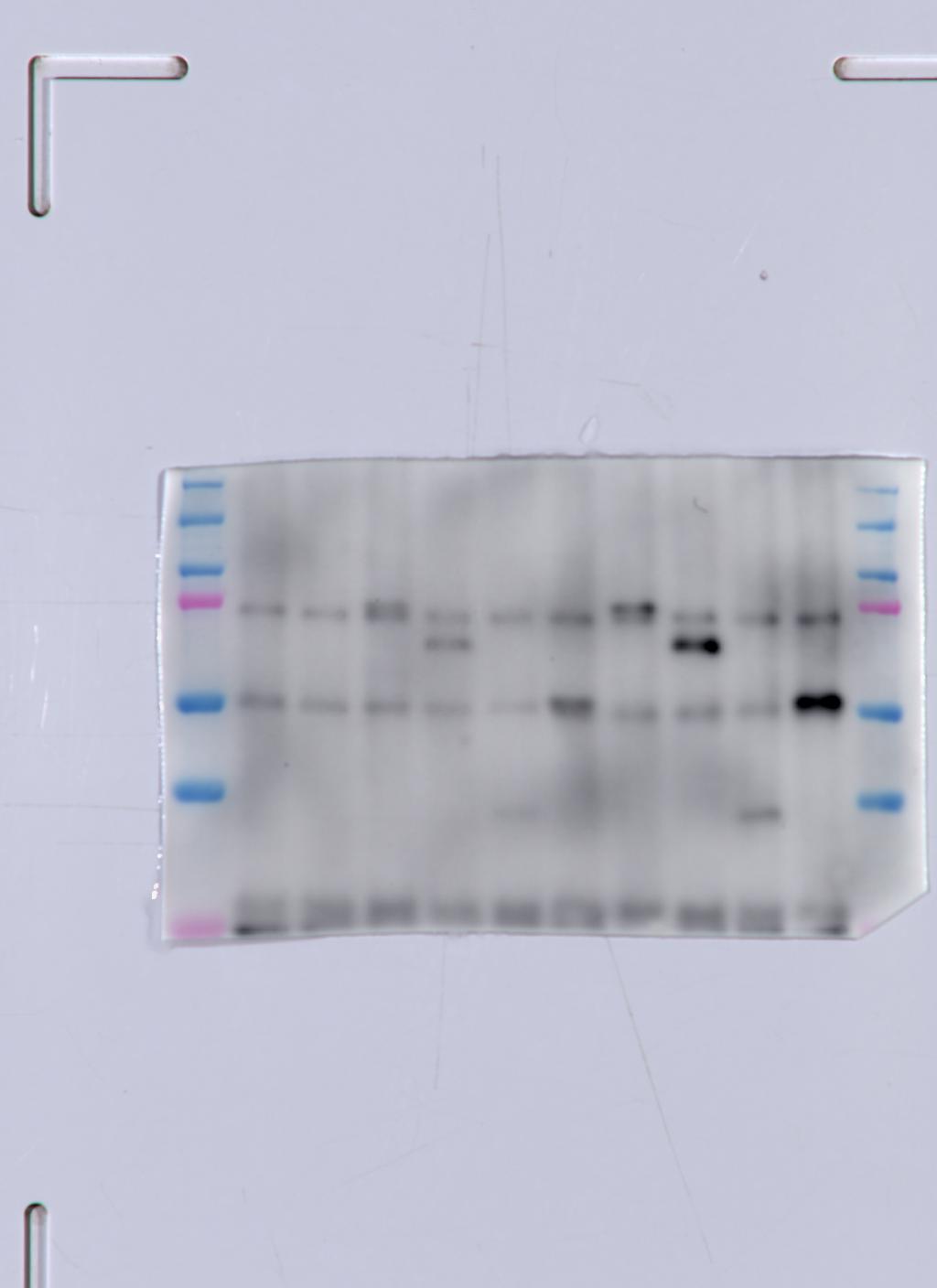

Supplement: Figure 2—figure supplement 2—source data 4. [file elife-76183-fig2-figsupp2-data4.zip › Figure 2-figure supplement 2-source data 4/Figure 2 S2D IP-GFP.jpg]

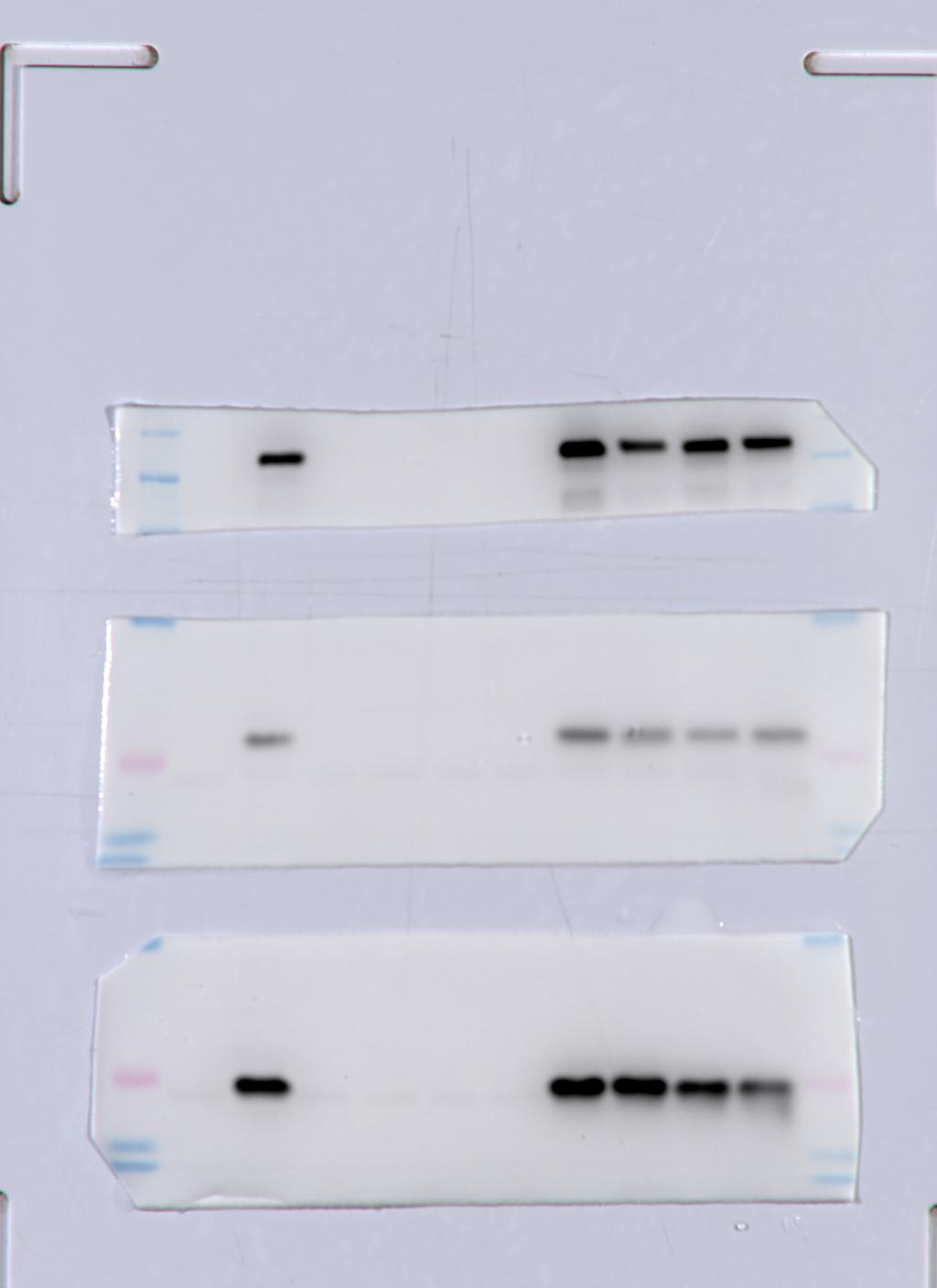

Supplement: Figure 2—figure supplement 2—source data 4. [file elife-76183-fig2-figsupp2-data4.zip › Figure 2-figure supplement 2-source data 4/Figure 2 S2D IP-Myc.jpg]

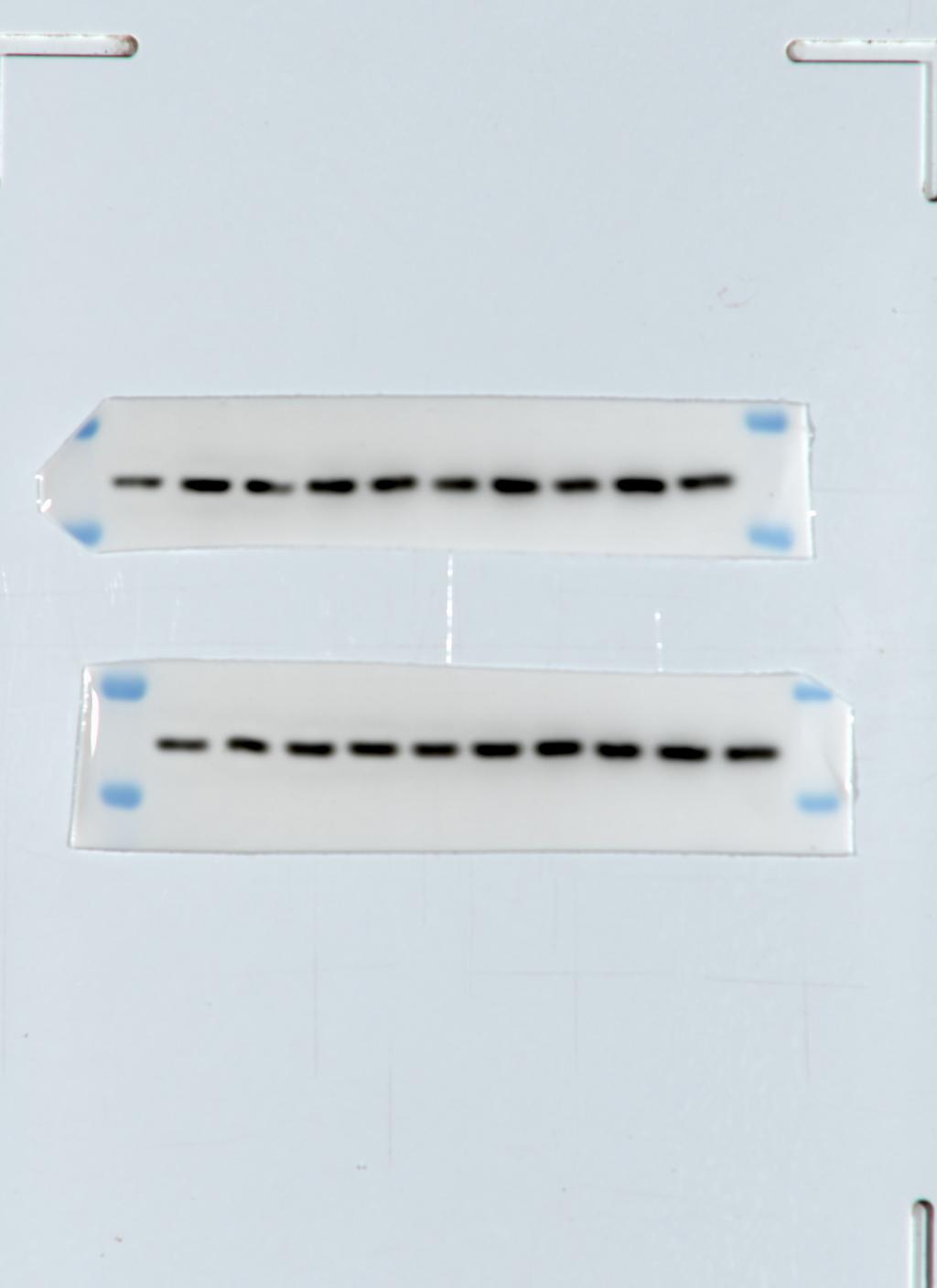

Supplement: Figure 2—figure supplement 3—source data 1. [file elife-76183-fig2-figsupp3-data1.zip › Figure 2-figure supplement 3-source data 1/Figure 2 S3C INPUT-Actin.jpg]

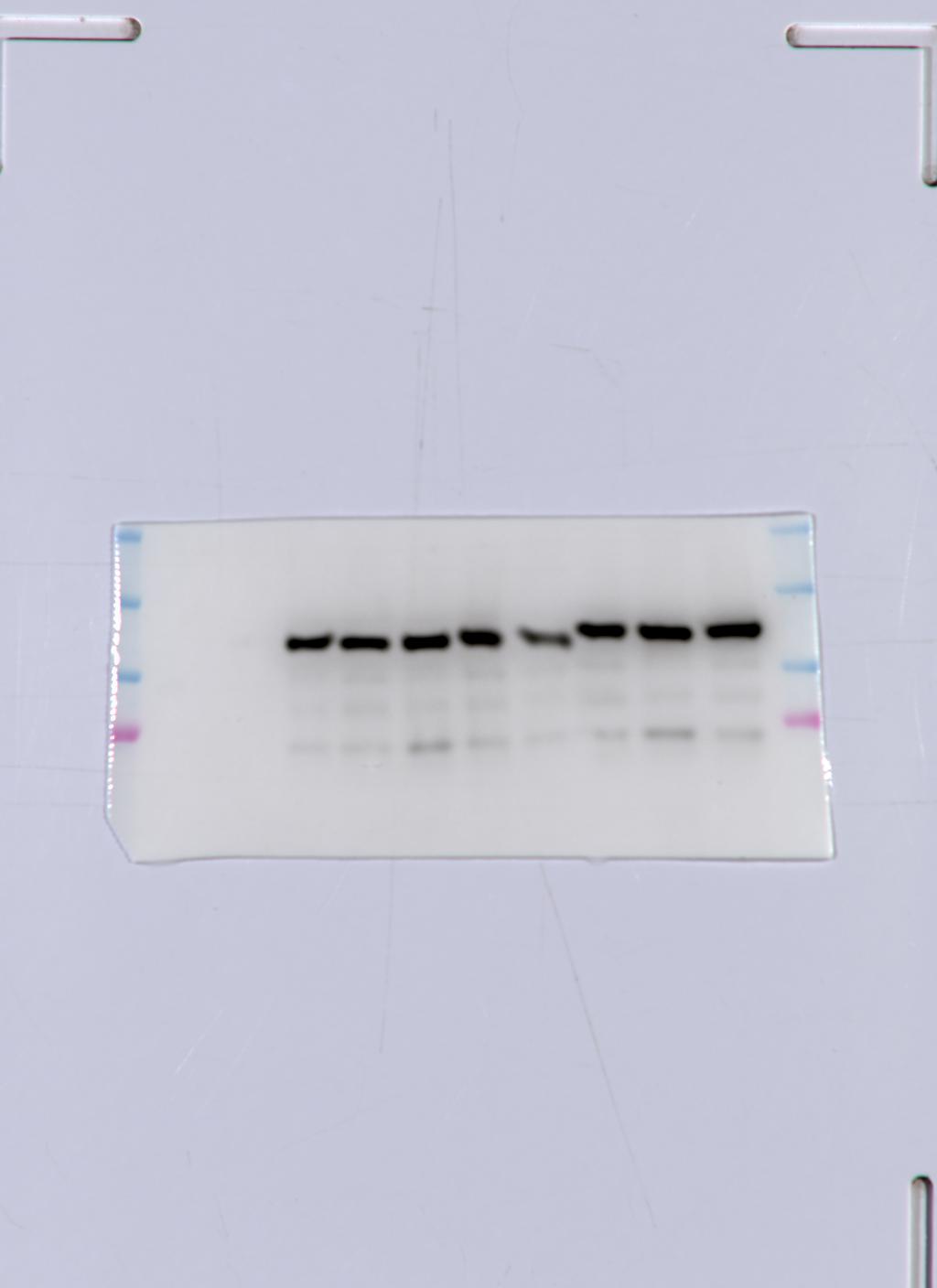

Supplement: Figure 2—figure supplement 3—source data 1. [file elife-76183-fig2-figsupp3-data1.zip › Figure 2-figure supplement 3-source data 1/Figure 2 S3C INPUT-GFP.jpg]

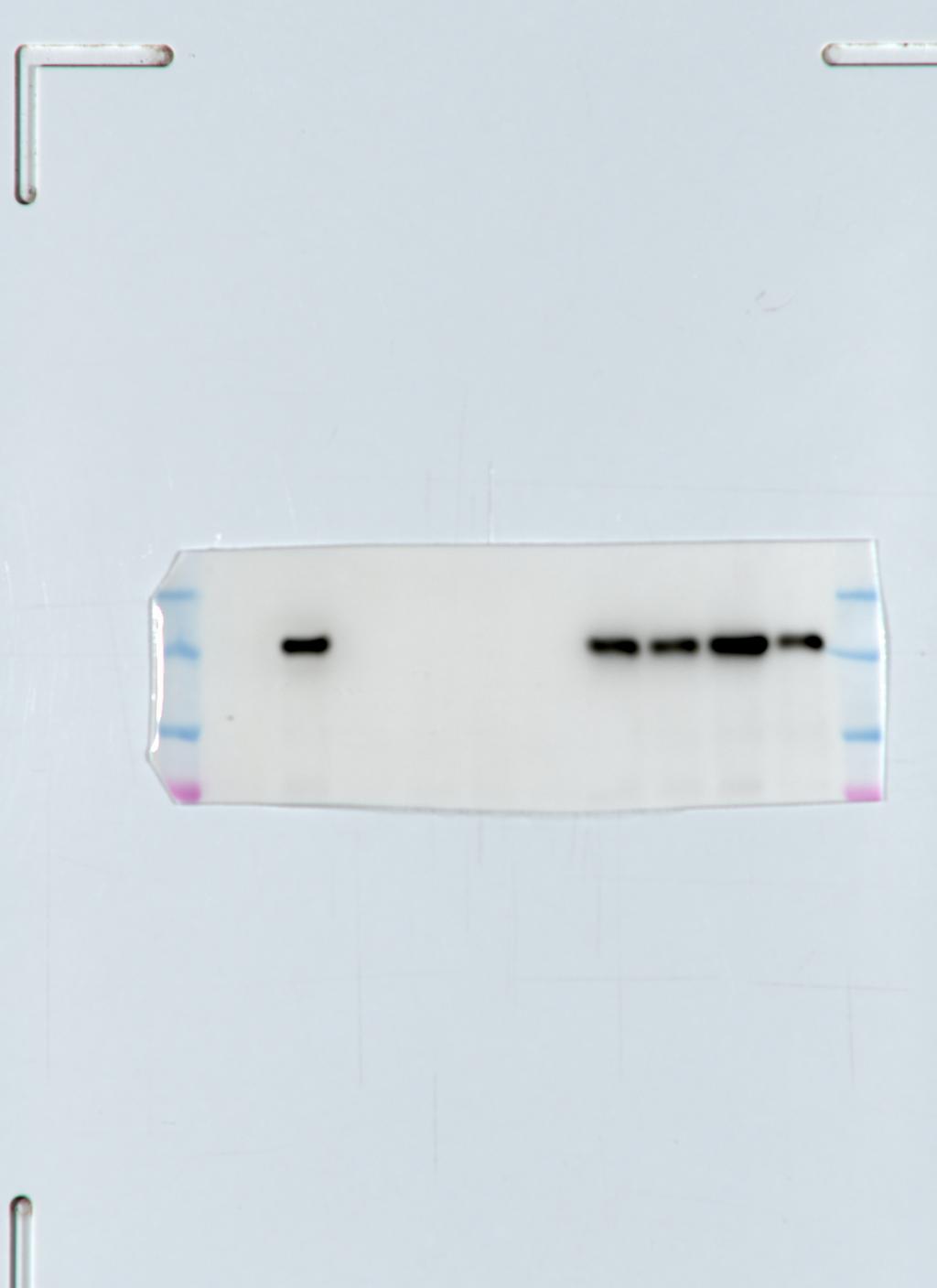

Supplement: Figure 2—figure supplement 3—source data 1. [file elife-76183-fig2-figsupp3-data1.zip › Figure 2-figure supplement 3-source data 1/Figure 2 S3C INPUT-Myc.jpg]

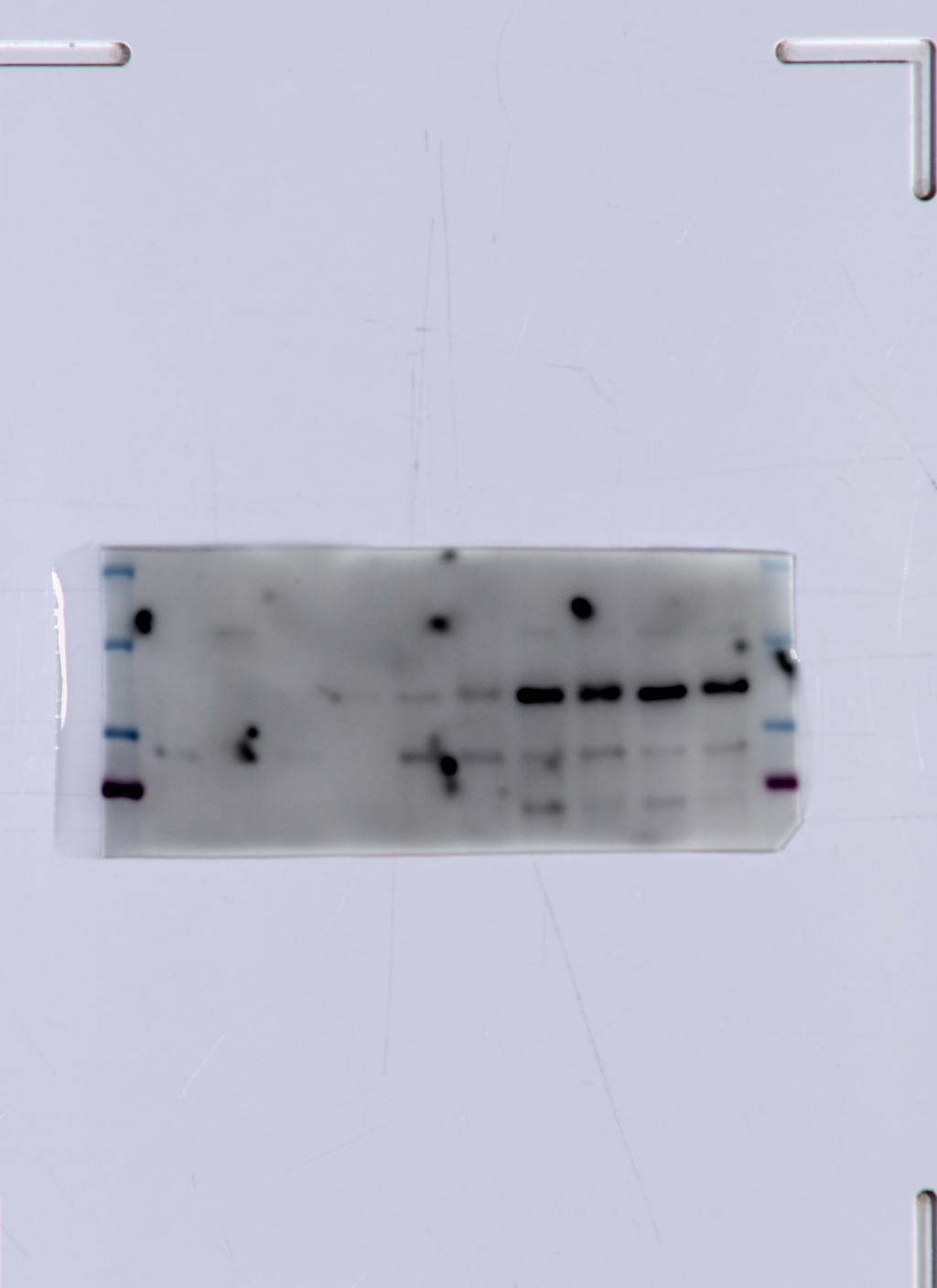

Supplement: Figure 2—figure supplement 3—source data 1. [file elife-76183-fig2-figsupp3-data1.zip › Figure 2-figure supplement 3-source data 1/Figure 2 S3C IP-GFP.jpg]

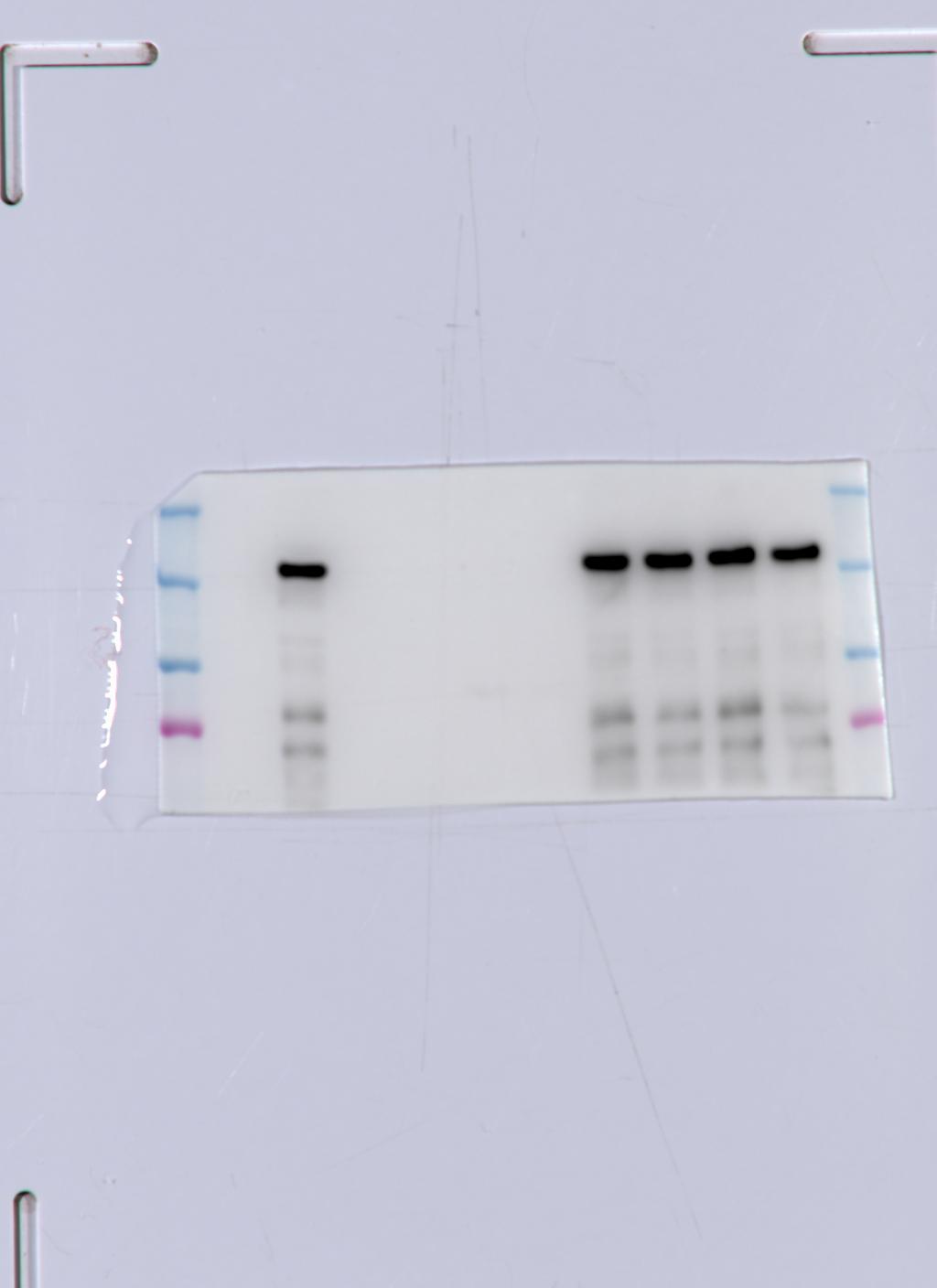

Supplement: Figure 2—figure supplement 3—source data 1. [file elife-76183-fig2-figsupp3-data1.zip › Figure 2-figure supplement 3-source data 1/Figure 2 S3C IP-Myc.jpg]

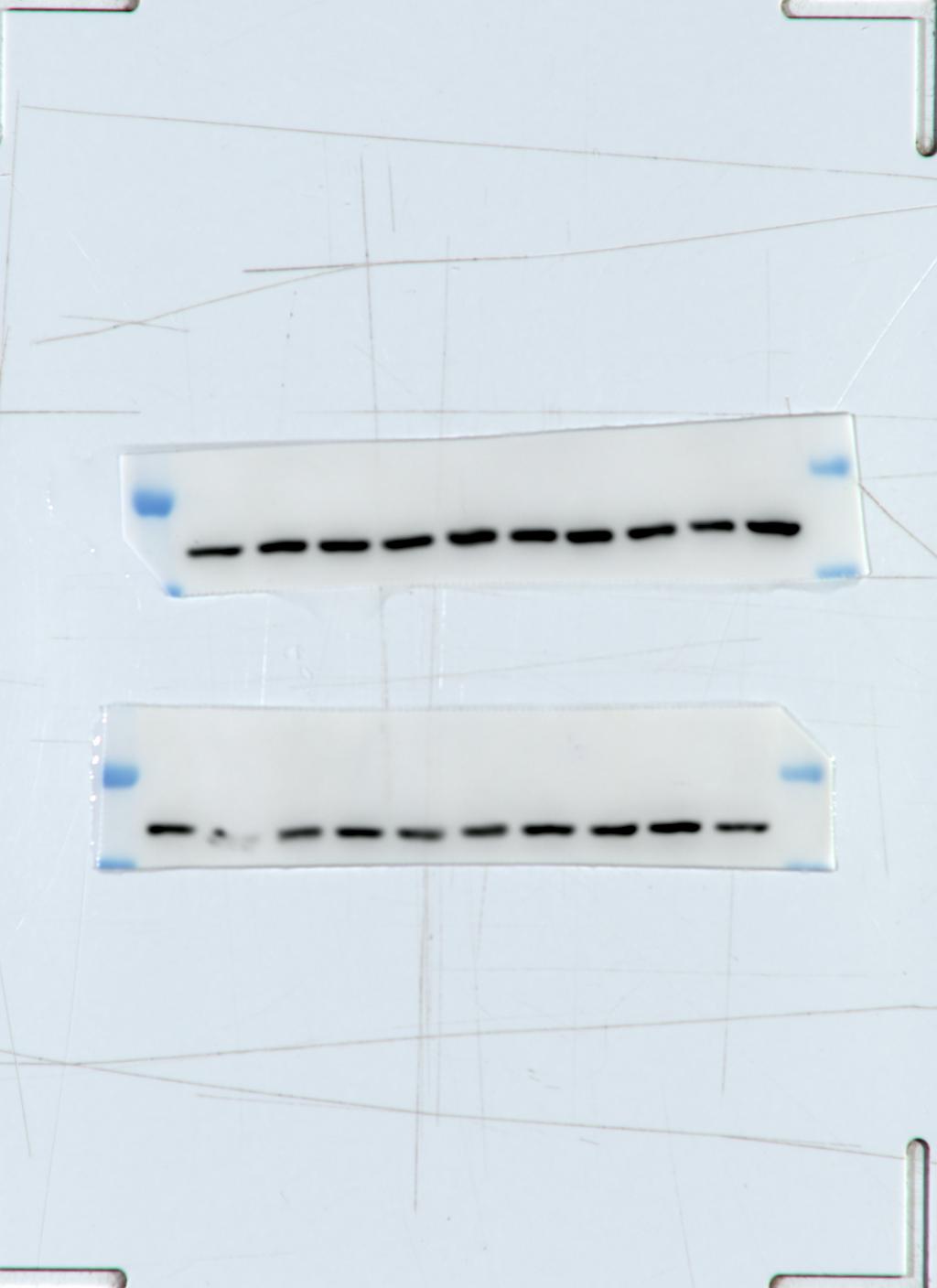

Supplement: Figure 2—figure supplement 3—source data 2. [file elife-76183-fig2-figsupp3-data2.zip › Figure 2-figure supplement 3-source data 2/Figure 2 S3D INPUT-Actin.jpg]

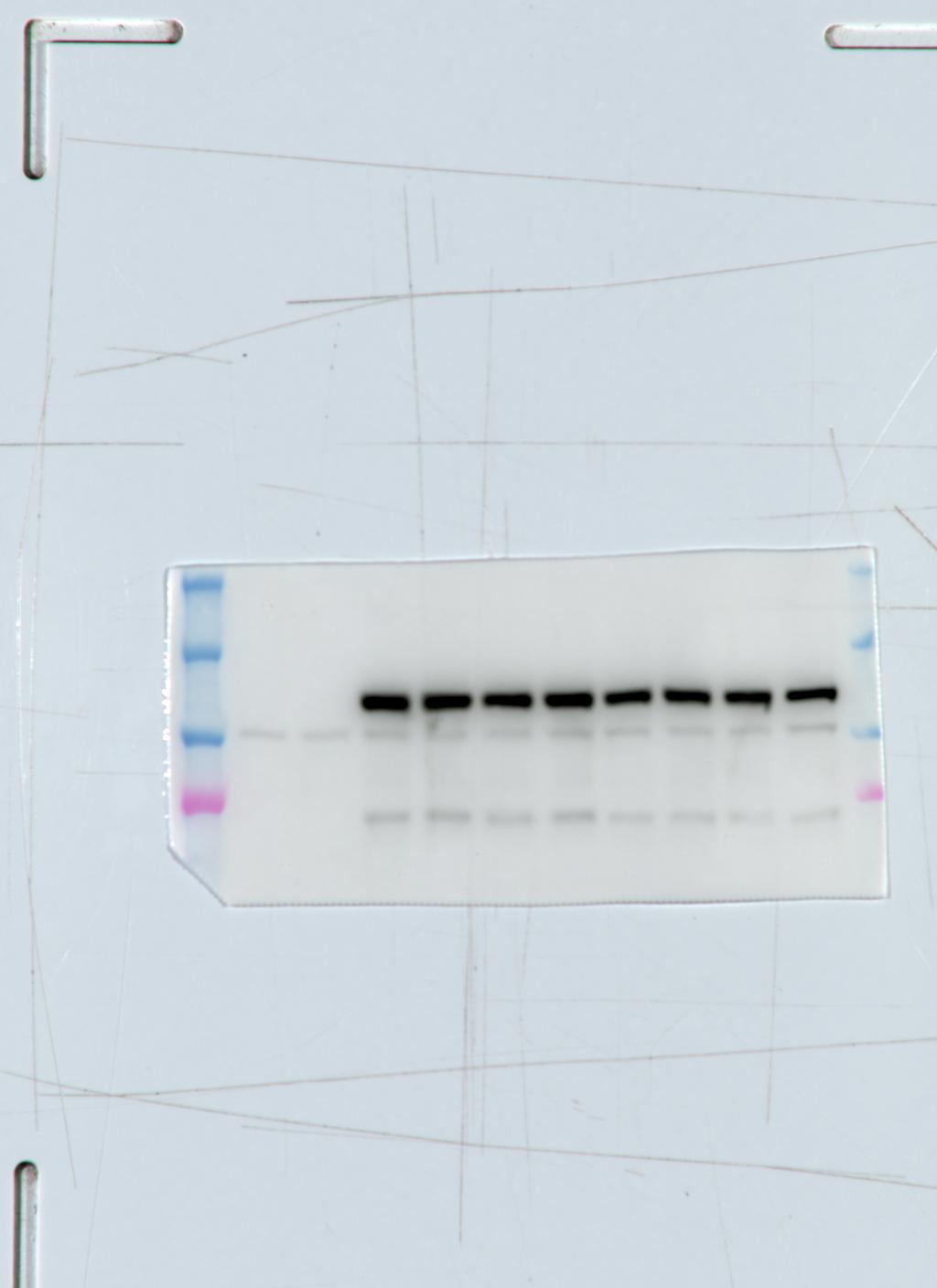

Supplement: Figure 2—figure supplement 3—source data 2. [file elife-76183-fig2-figsupp3-data2.zip › Figure 2-figure supplement 3-source data 2/Figure 2 S3D INPUT-GFP.jpg]

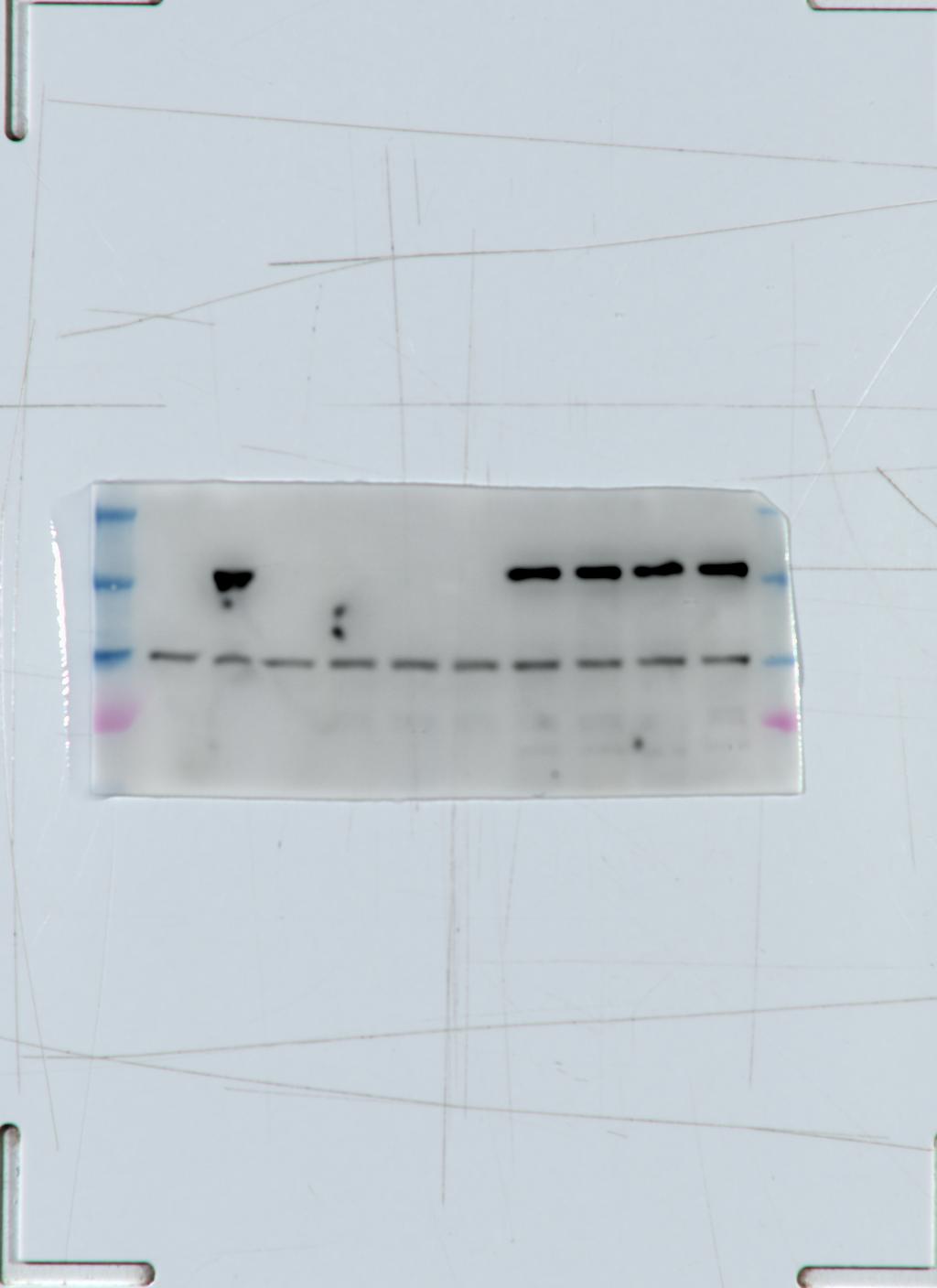

Supplement: Figure 2—figure supplement 3—source data 2. [file elife-76183-fig2-figsupp3-data2.zip › Figure 2-figure supplement 3-source data 2/Figure 2 S3D INPUT-Myc.jpg]

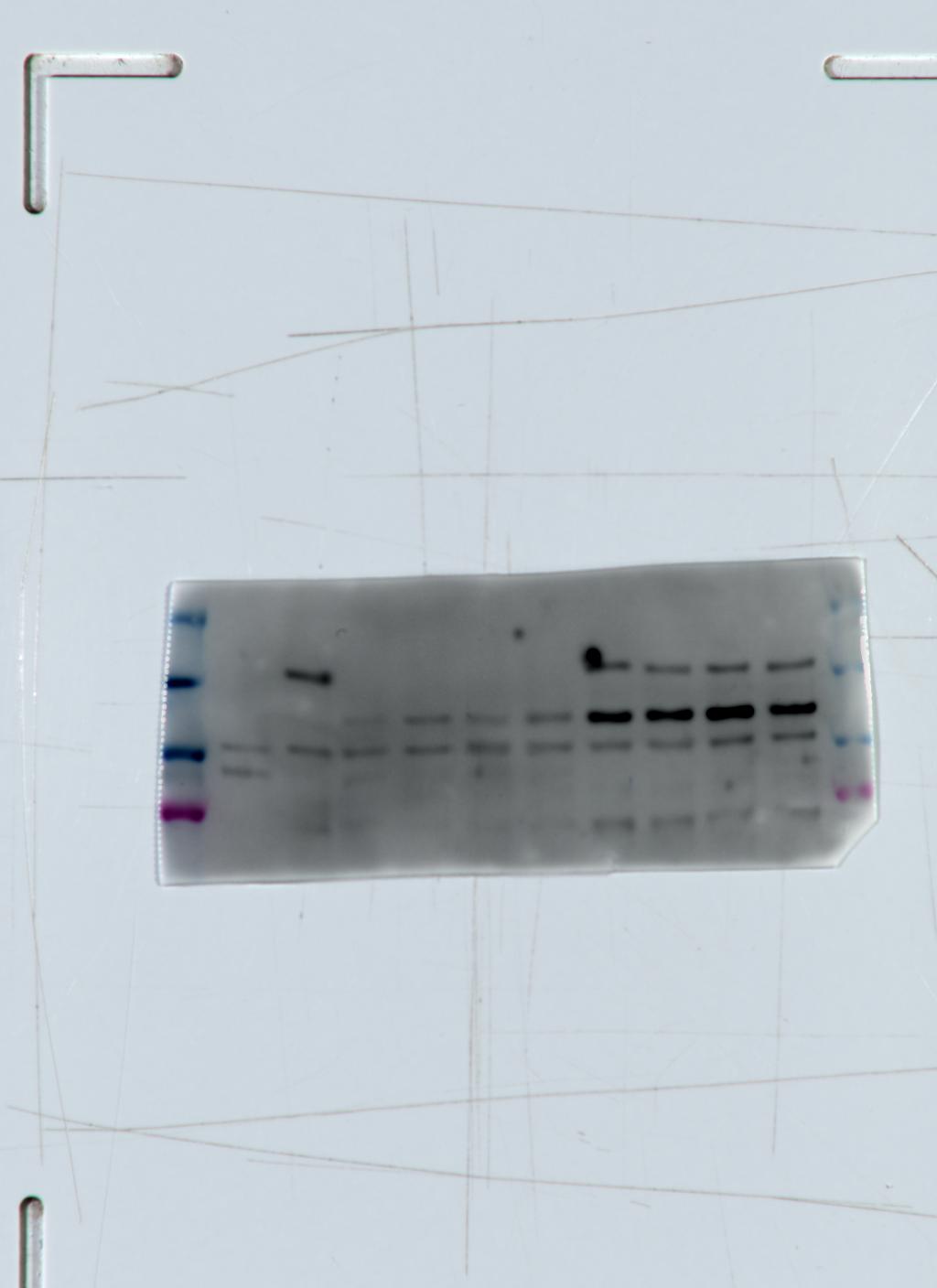

Supplement: Figure 2—figure supplement 3—source data 2. [file elife-76183-fig2-figsupp3-data2.zip › Figure 2-figure supplement 3-source data 2/Figure 2 S3D IP-GFP.jpg]

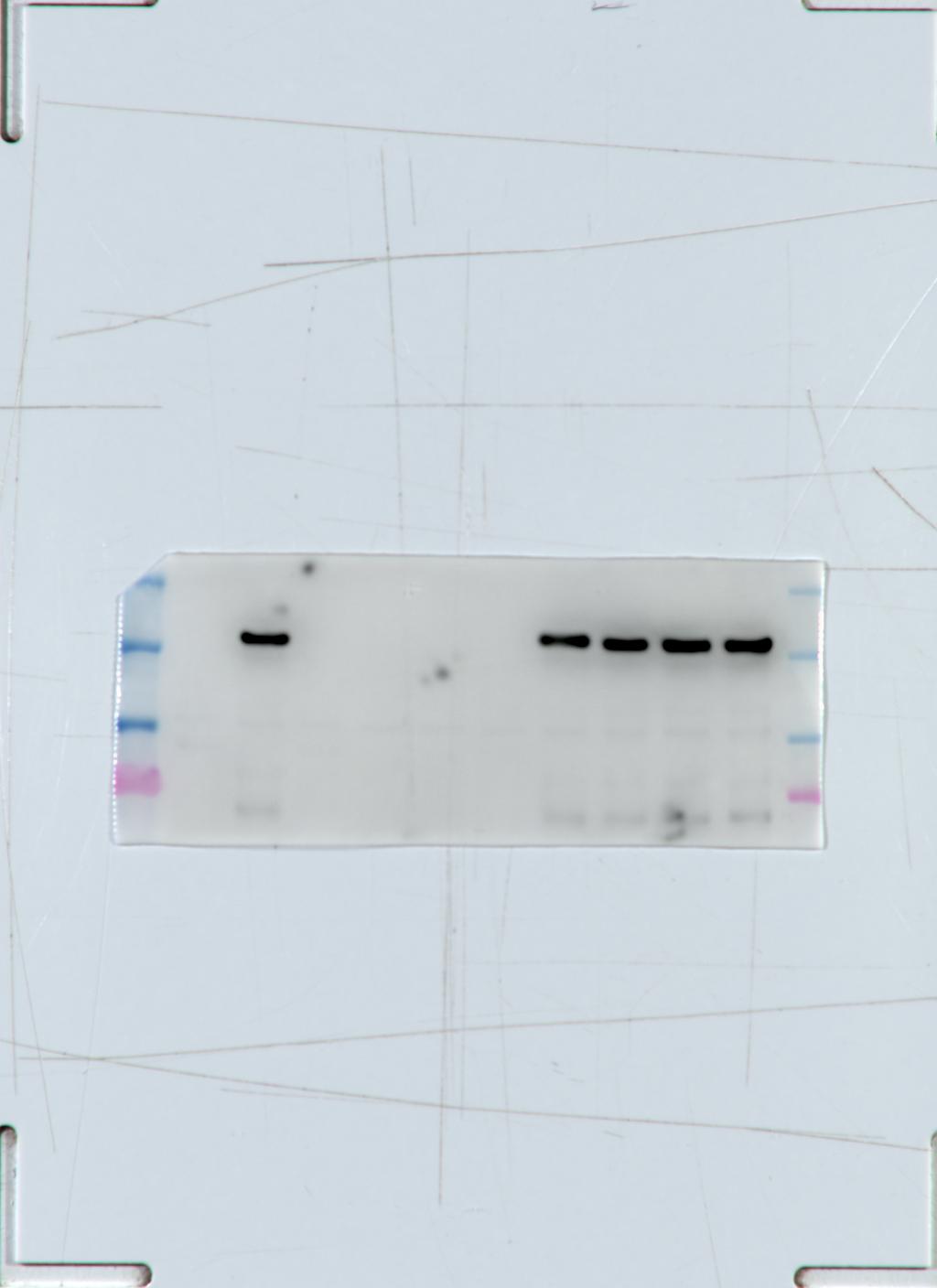

Supplement: Figure 2—figure supplement 3—source data 2. [file elife-76183-fig2-figsupp3-data2.zip › Figure 2-figure supplement 3-source data 2/Figure 2 S3D IP-Myc.jpg]

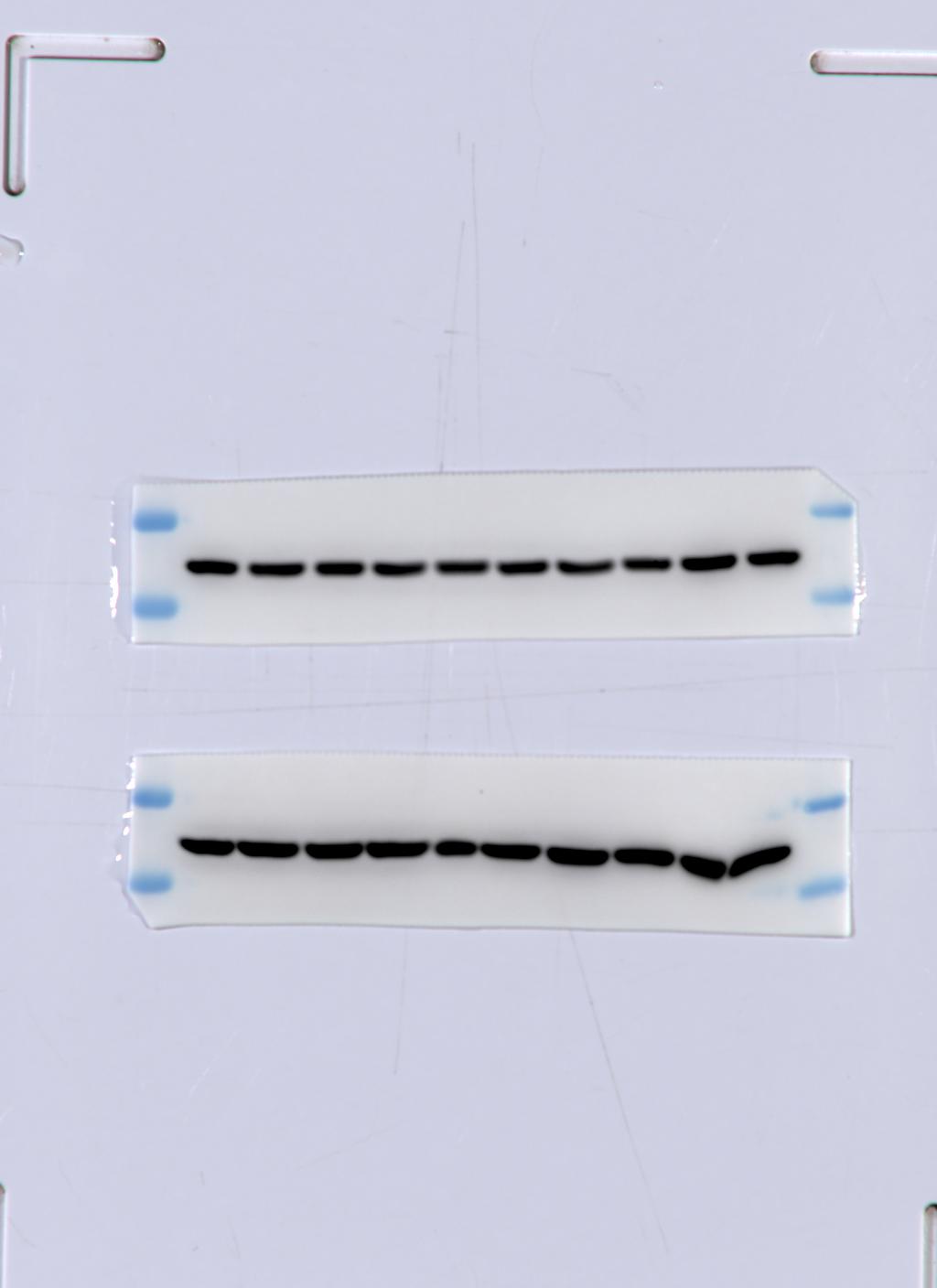

Supplement: Figure 2—figure supplement 3—source data 3. [file elife-76183-fig2-figsupp3-data3.zip › Figure 2-figure supplement 3-source data 3/Figure 2 S3E INPUT-Actin.jpg]

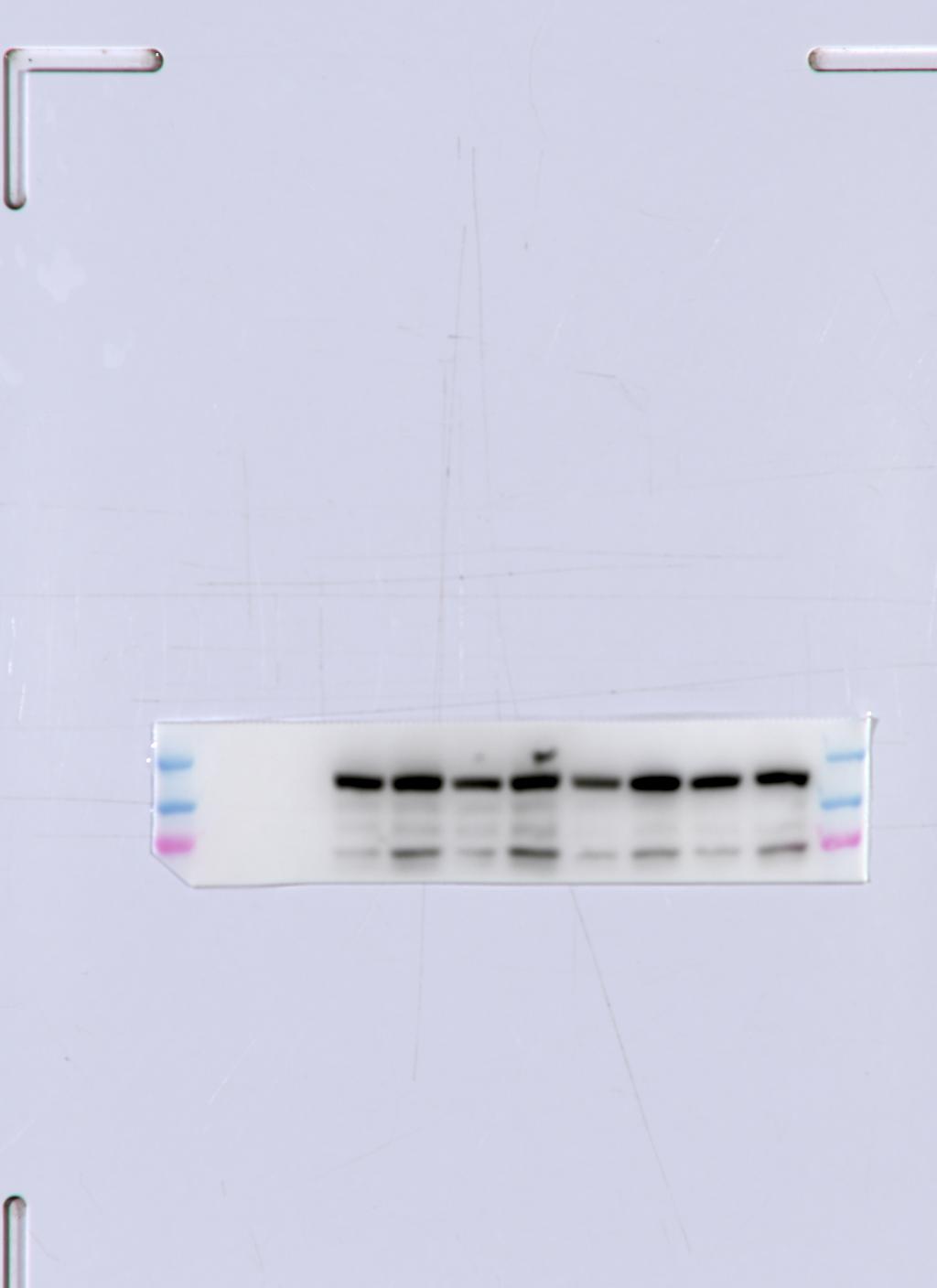

Supplement: Figure 2—figure supplement 3—source data 3. [file elife-76183-fig2-figsupp3-data3.zip › Figure 2-figure supplement 3-source data 3/Figure 2 S3E INPUT-GFP.jpg]

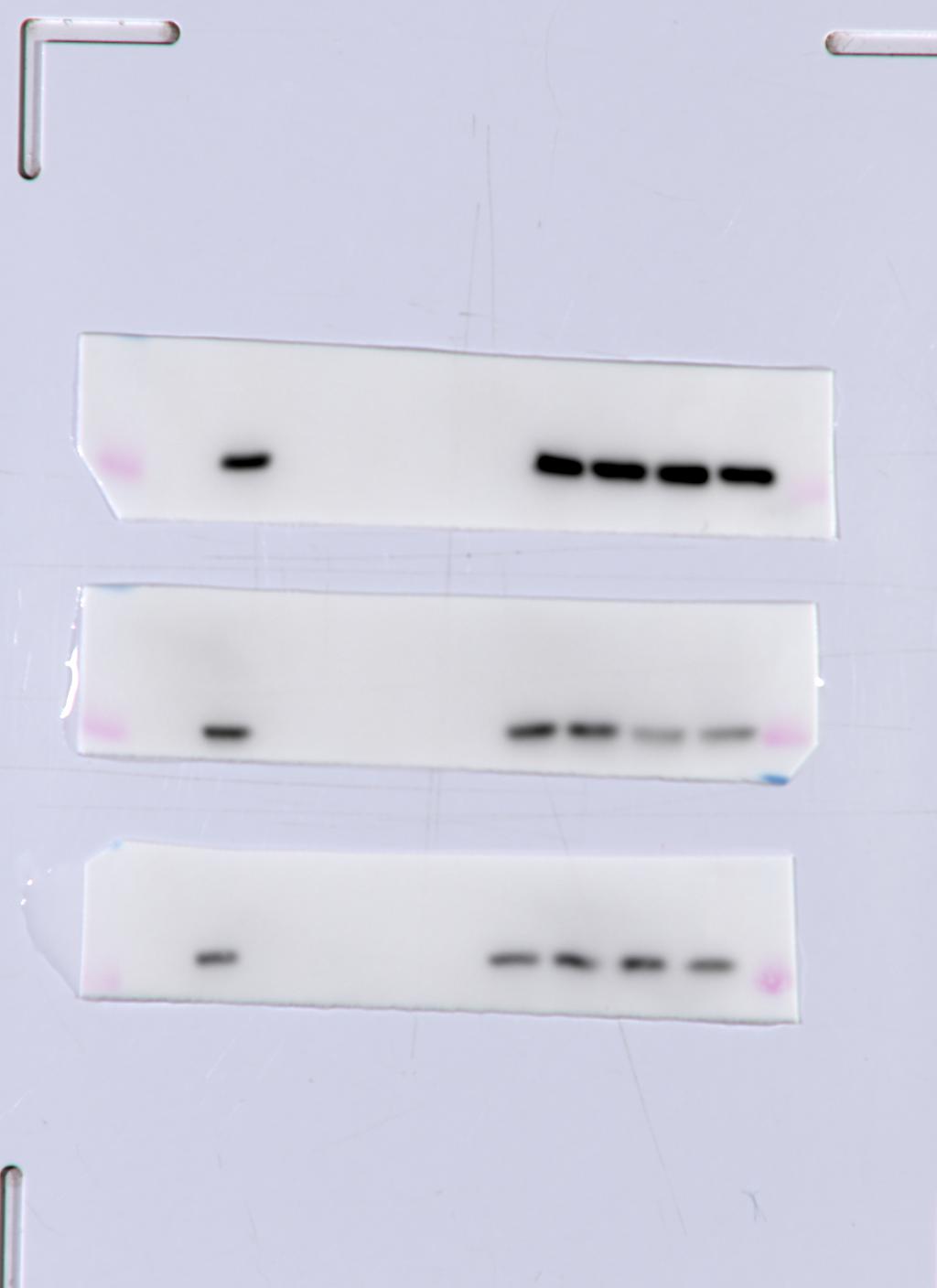

Supplement: Figure 2—figure supplement 3—source data 3. [file elife-76183-fig2-figsupp3-data3.zip › Figure 2-figure supplement 3-source data 3/Figure 2 S3E INPUT-Myc.jpg]

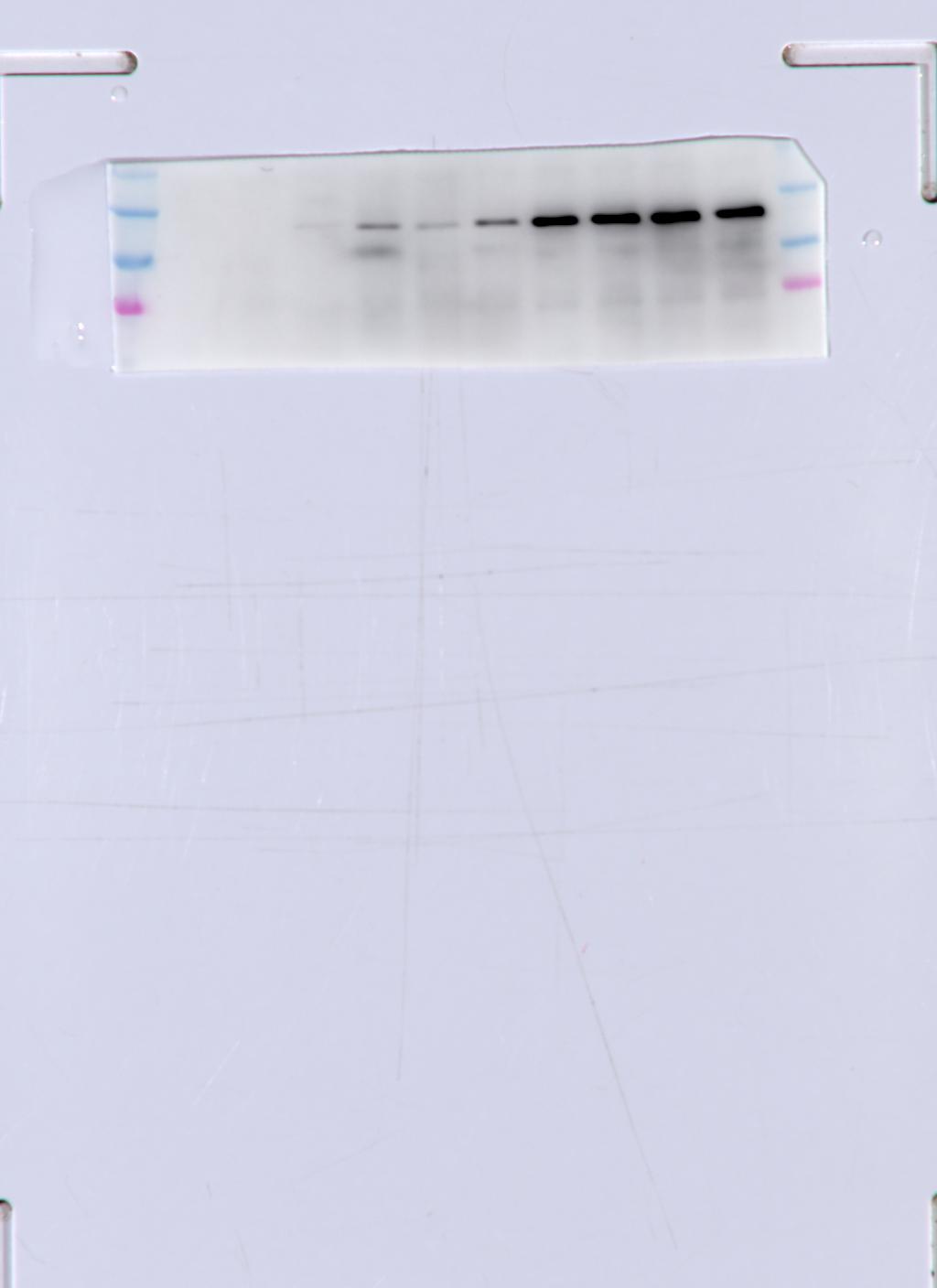

Supplement: Figure 2—figure supplement 3—source data 3. [file elife-76183-fig2-figsupp3-data3.zip › Figure 2-figure supplement 3-source data 3/Figure 2 S3E IP-GFP.jpg]

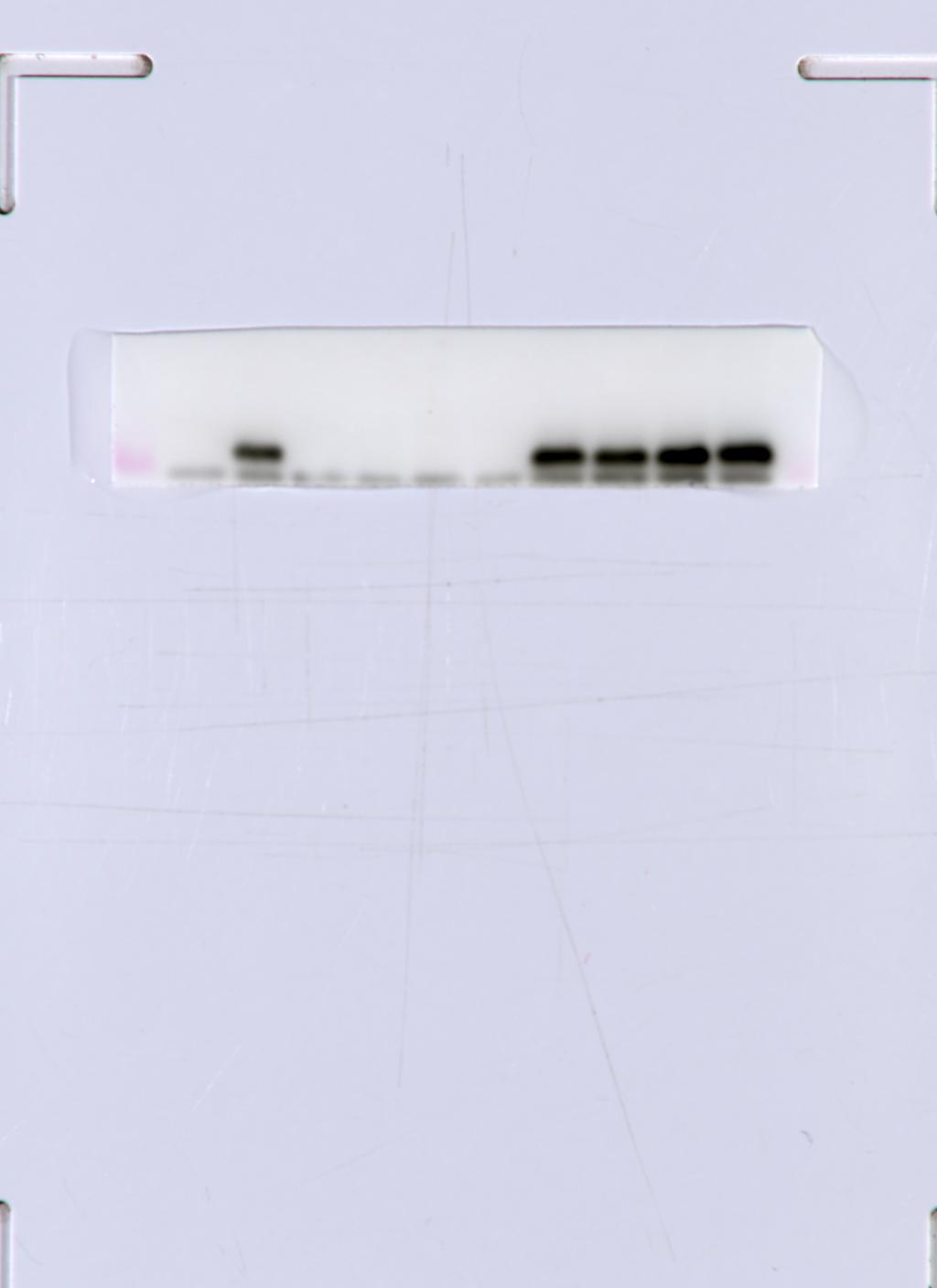

Supplement: Figure 2—figure supplement 3—source data 3. [file elife-76183-fig2-figsupp3-data3.zip › Figure 2-figure supplement 3-source data 3/Figure 2 S3E IP-Myc.jpg]

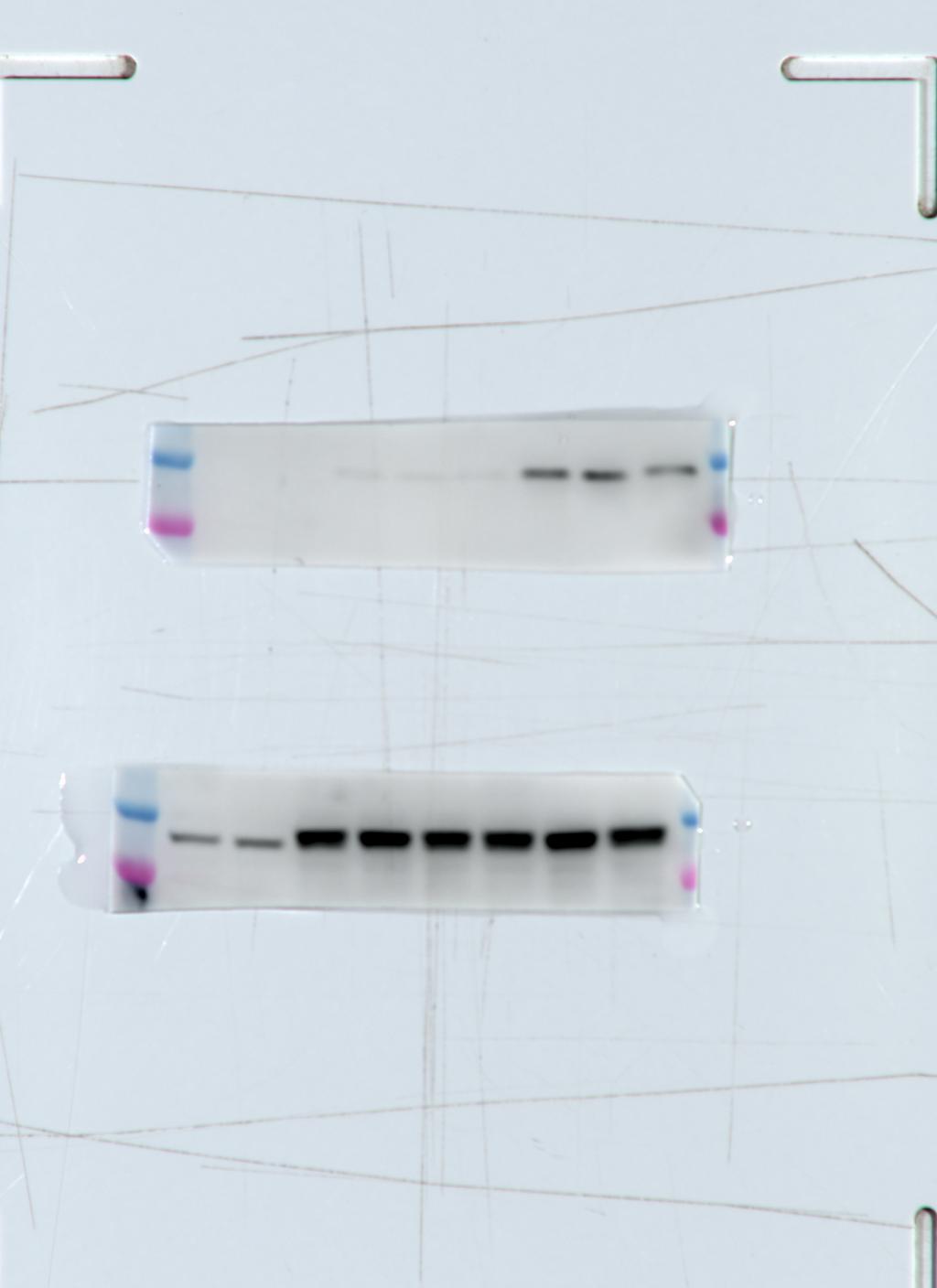

Supplement: Figure 2—figure supplement 3—source data 4. [file elife-76183-fig2-figsupp3-data4.zip › Figure 2-figure supplement 3-source data 4/Figure 2 S3F INPUT-FER.jpg]

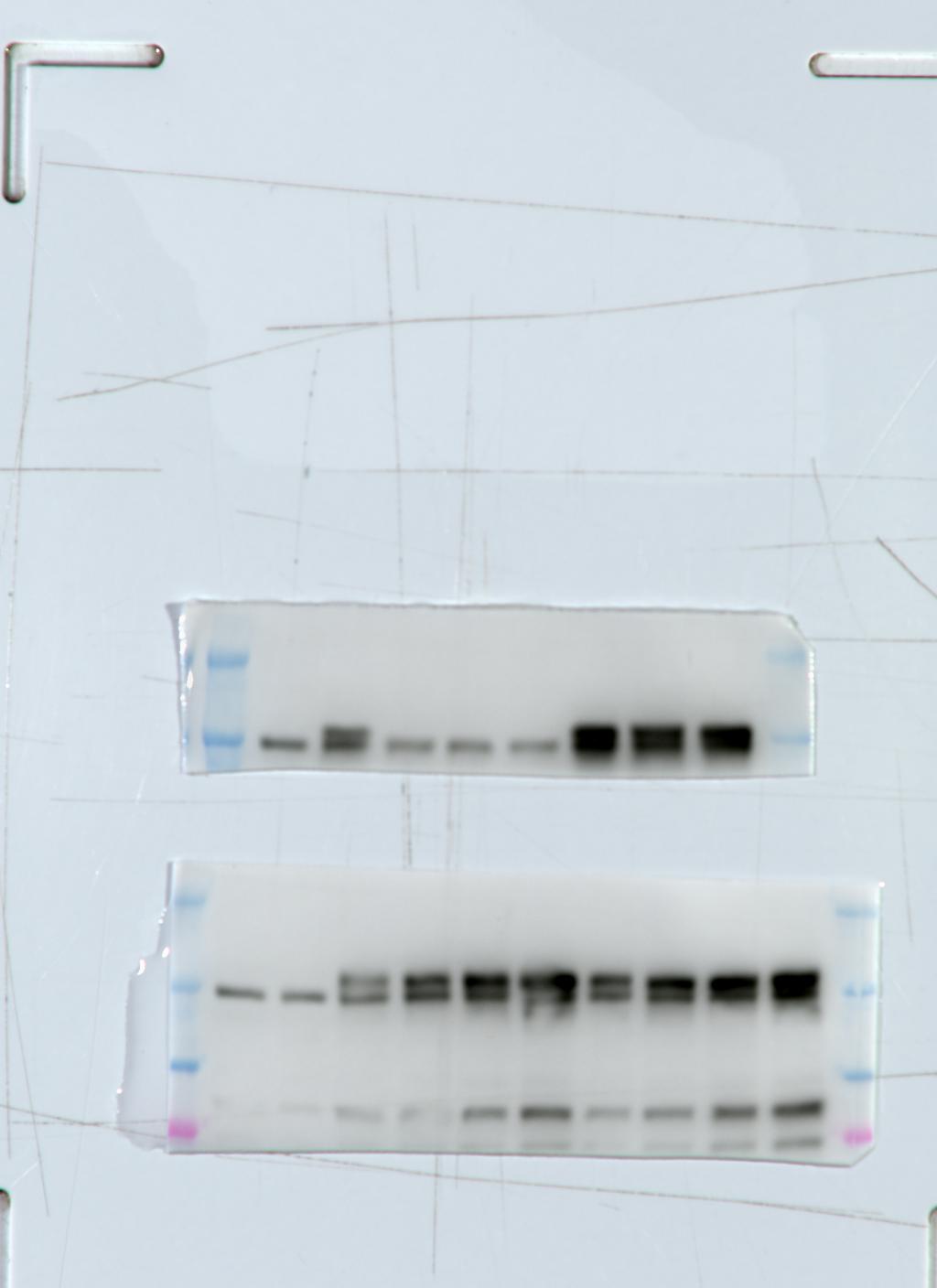

Supplement: Figure 2—figure supplement 3—source data 4. [file elife-76183-fig2-figsupp3-data4.zip › Figure 2-figure supplement 3-source data 4/Figure 2 S3F INPUT-IRS4.jpg]

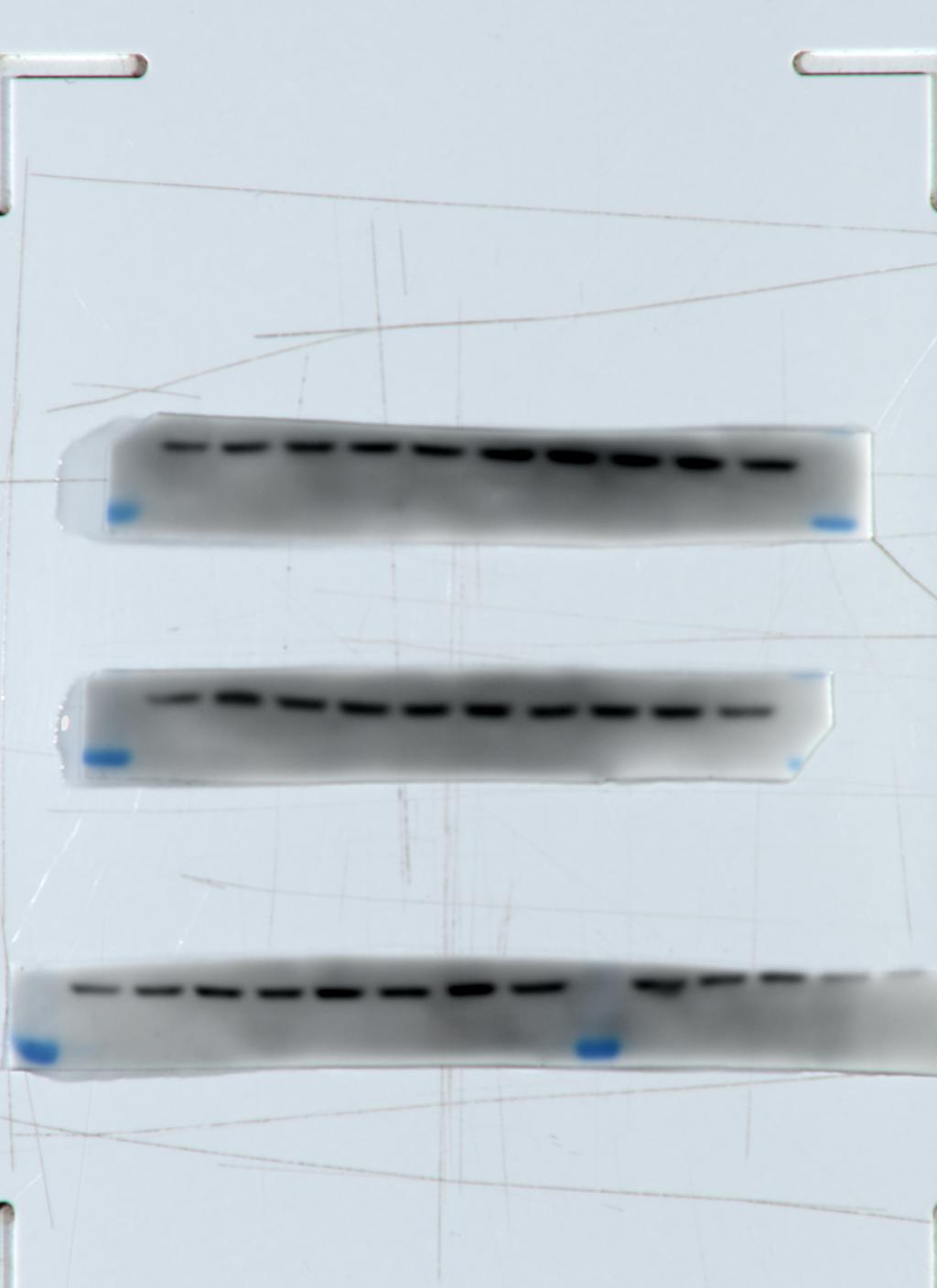

Supplement: Figure 2—figure supplement 3—source data 4. [file elife-76183-fig2-figsupp3-data4.zip › Figure 2-figure supplement 3-source data 4/Figure 2 S3F INPUT-Tubulin.jpg]

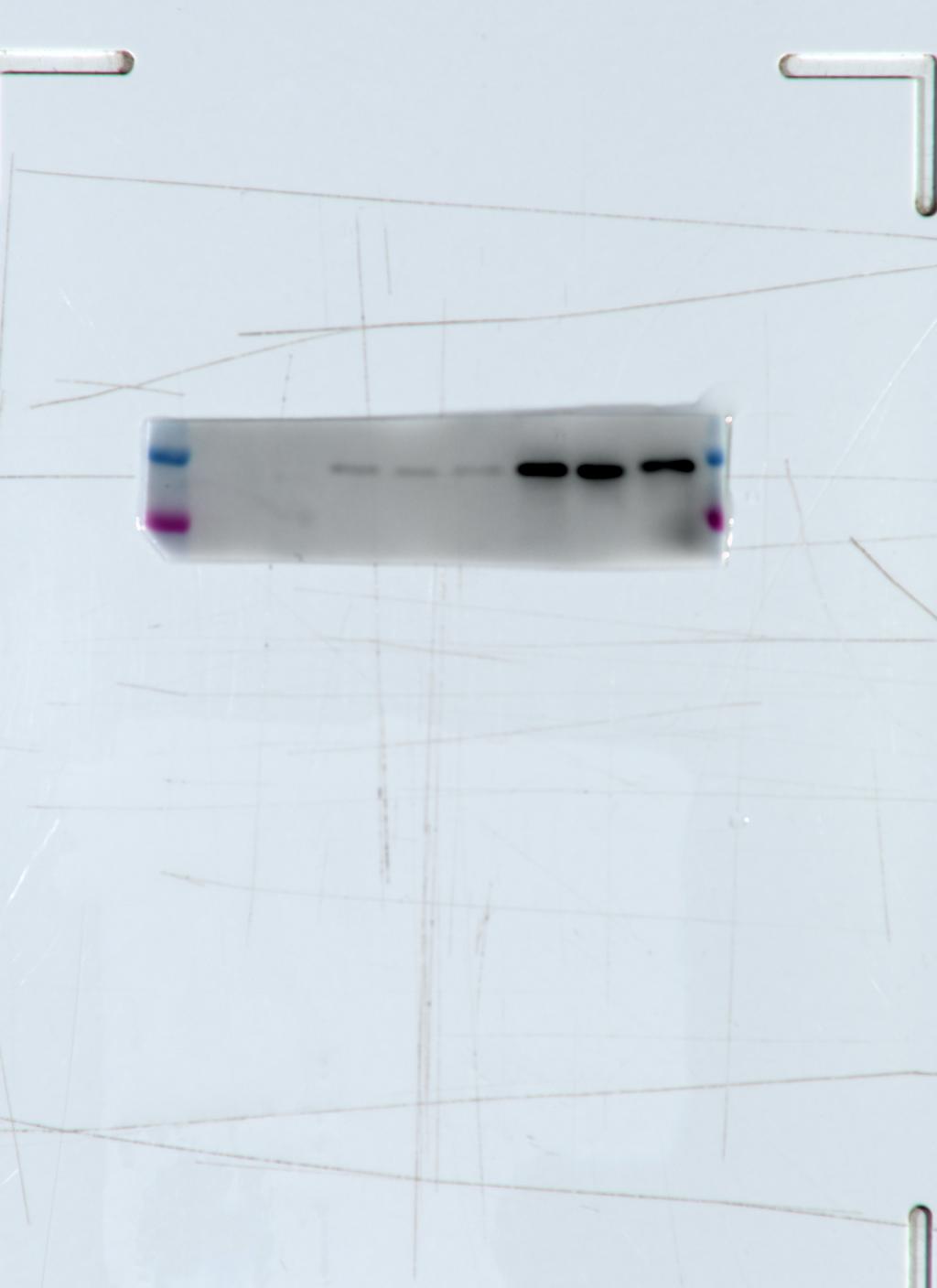

Supplement: Figure 2—figure supplement 3—source data 4. [file elife-76183-fig2-figsupp3-data4.zip › Figure 2-figure supplement 3-source data 4/Figure 2 S3F IP-FER.jpg]

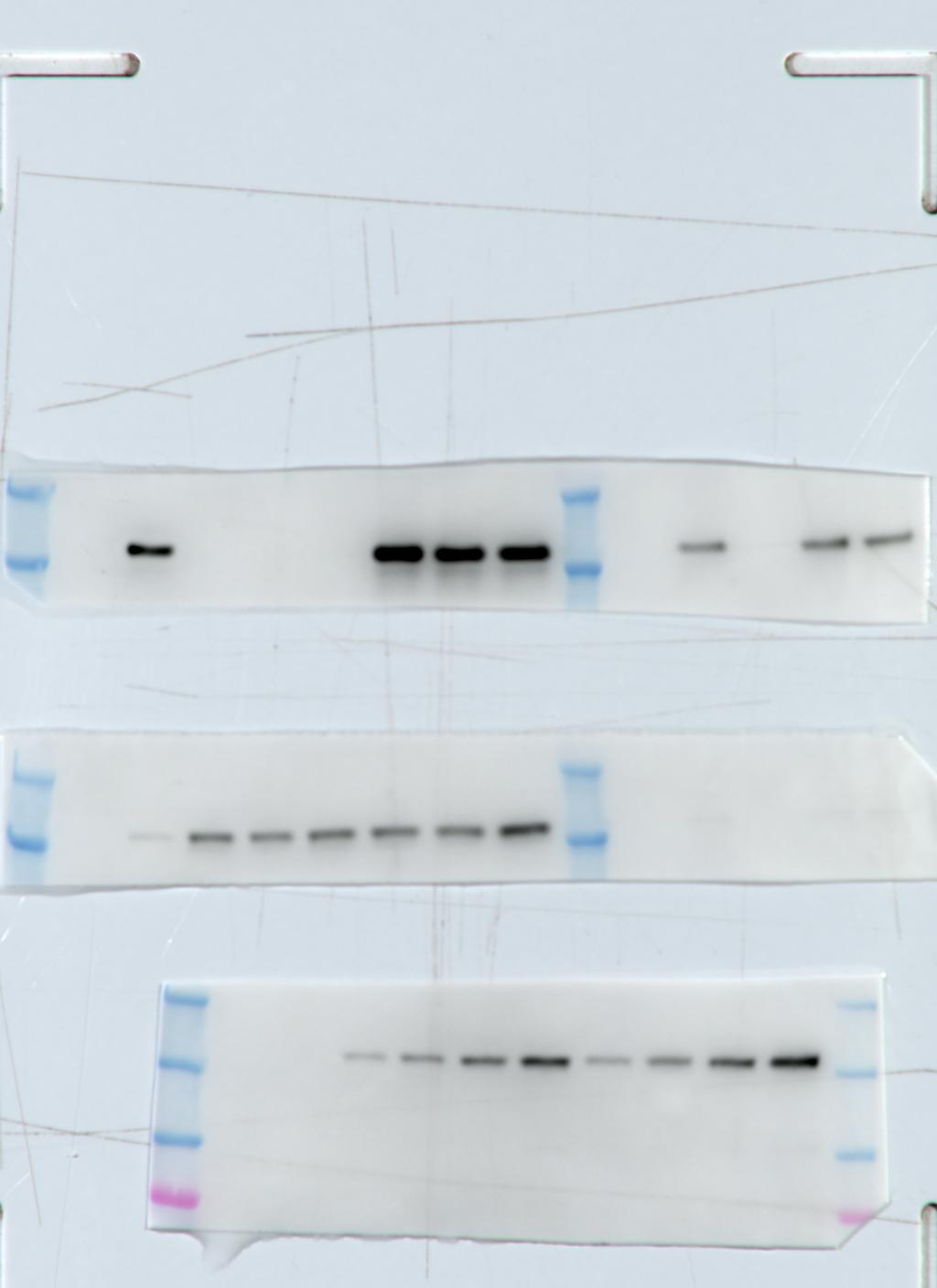

Supplement: Figure 2—figure supplement 3—source data 4. [file elife-76183-fig2-figsupp3-data4.zip › Figure 2-figure supplement 3-source data 4/Figure 2 S3F IP-Myc.jpg]

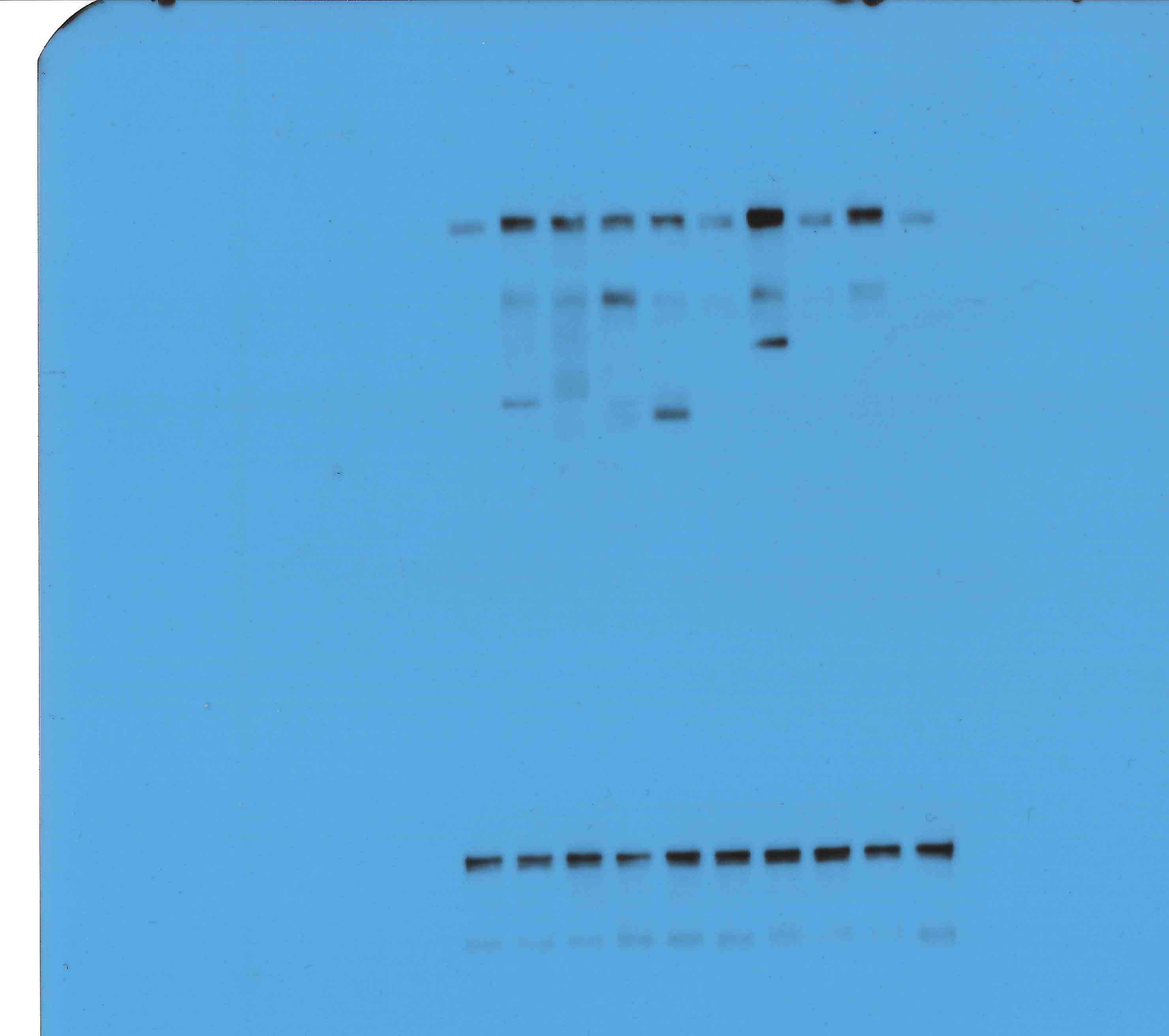

Supplement: Figure 3—source data 1. [file elife-76183-fig3-data1.zip › Figure 3-source data 1/Figure 3A IP-4G10.jpg]

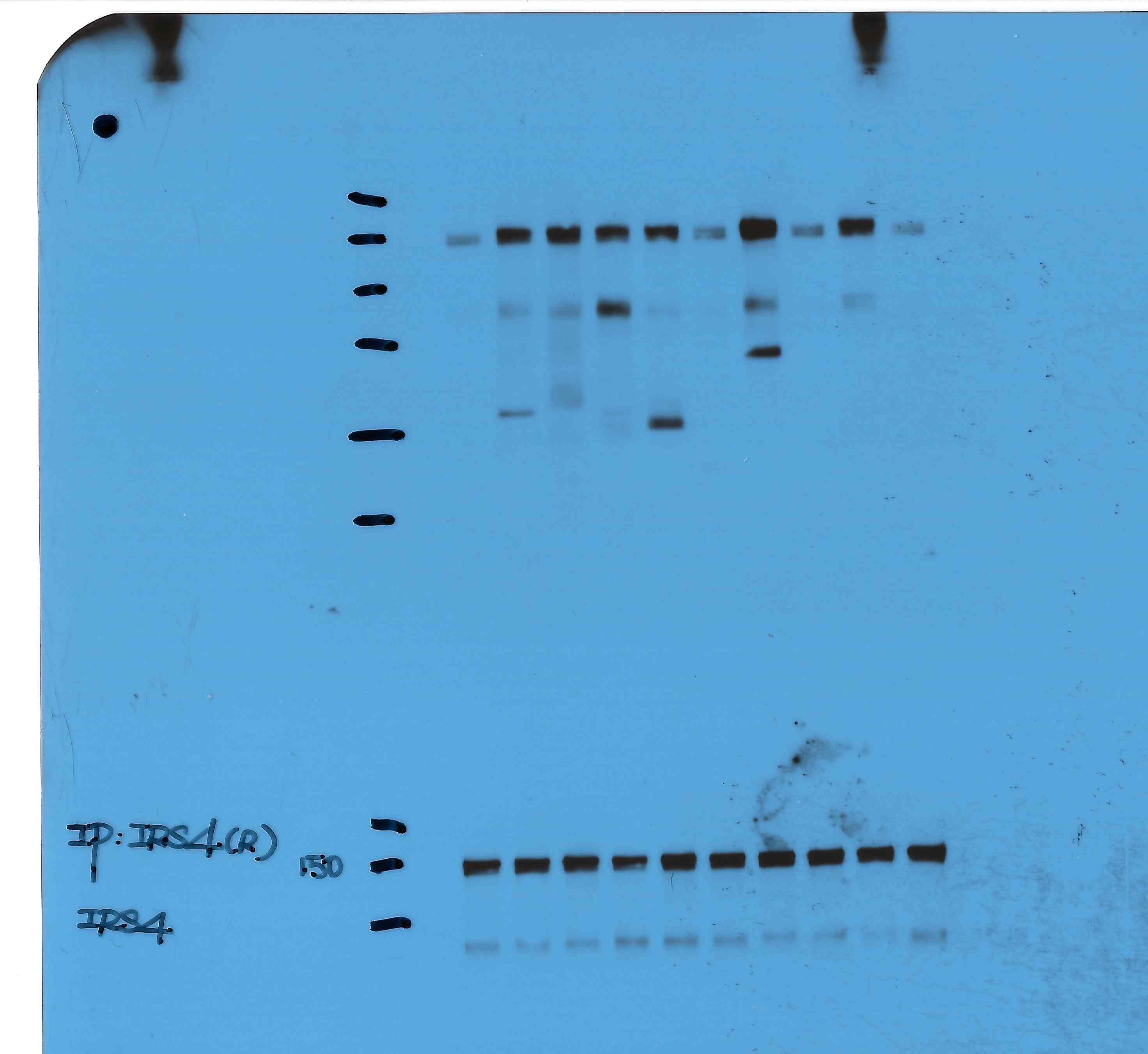

Supplement: Figure 3—source data 1. [file elife-76183-fig3-data1.zip › Figure 3-source data 1/Figure 3A IP-IRS4.jpg]

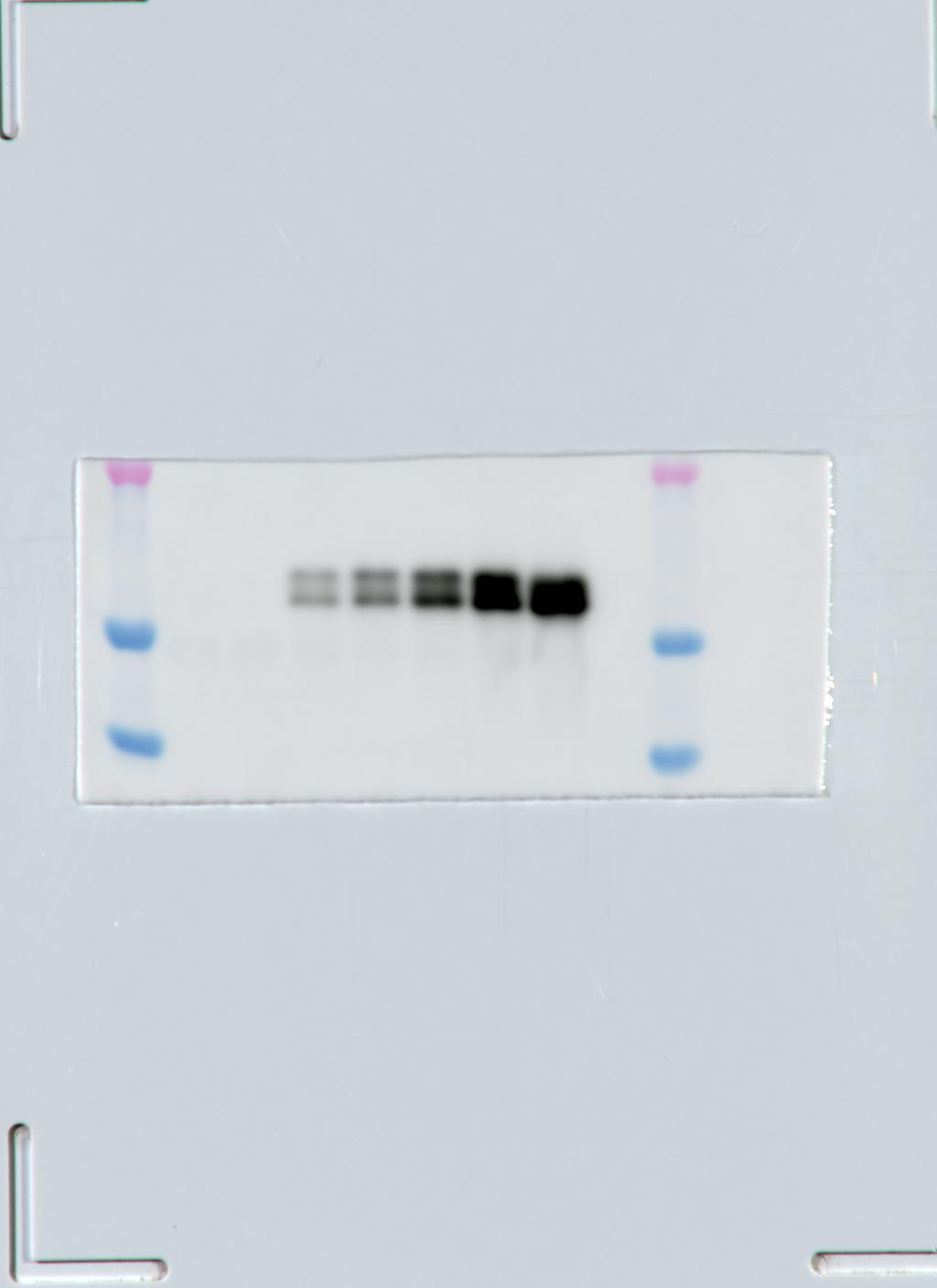

Supplement: Figure 3—source data 2. [file elife-76183-fig3-data2.zip › Figure 3-source data 2/Figure 3B IP Myc WB GST.jpg]

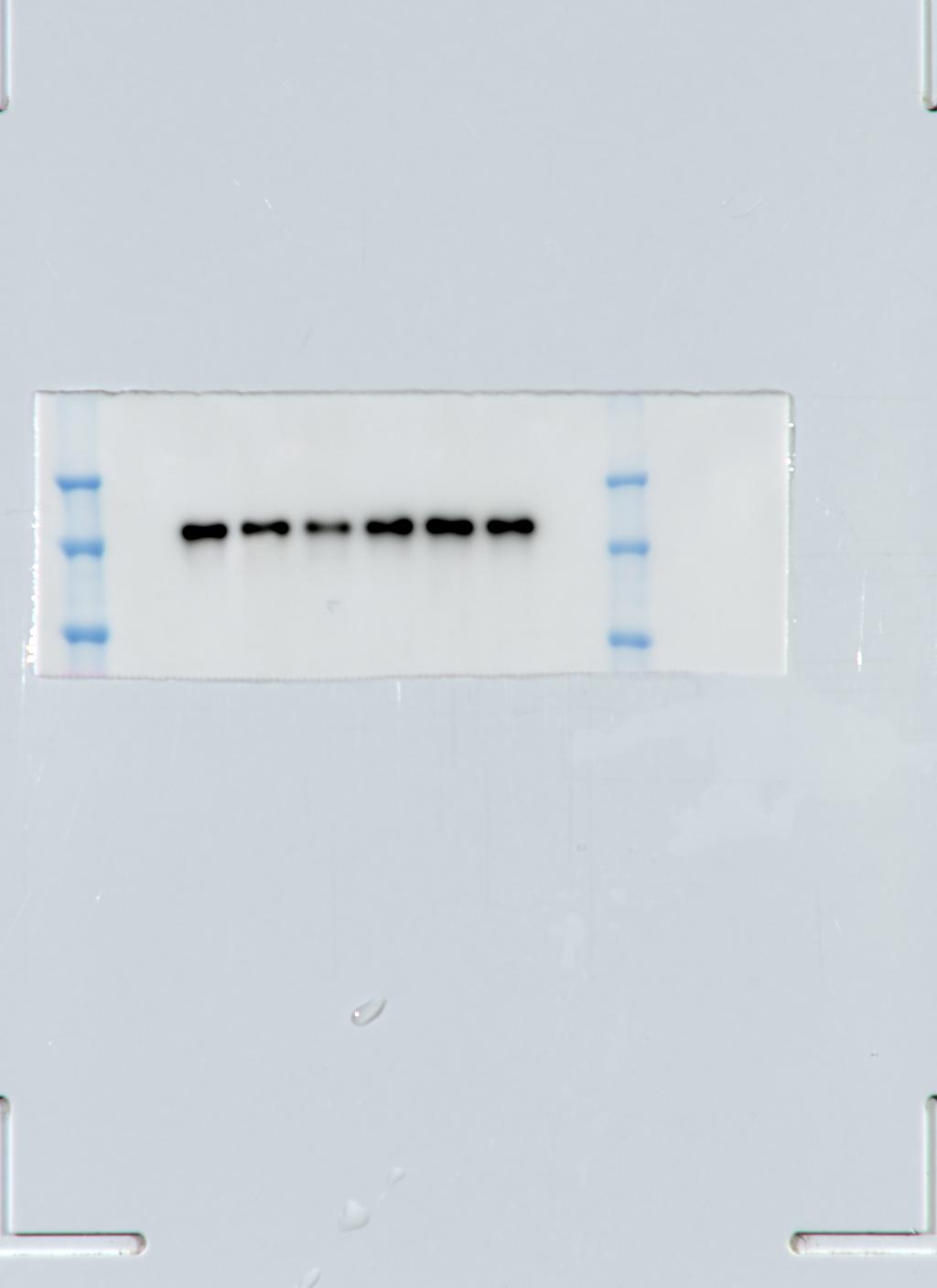

Supplement: Figure 3—source data 2. [file elife-76183-fig3-data2.zip › Figure 3-source data 2/Figure 3B IP Myc WB IRS4.jpg]

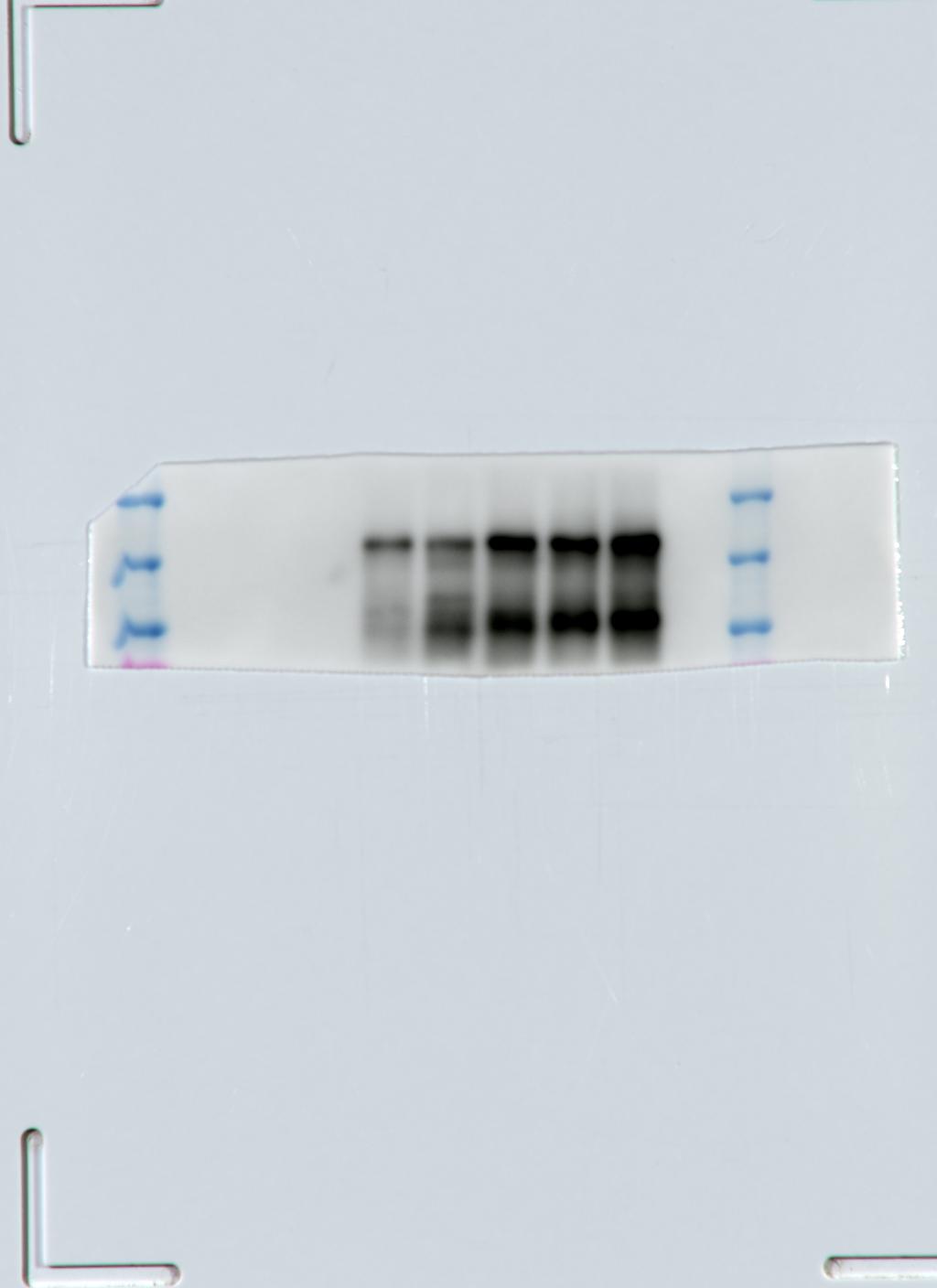

Supplement: Figure 3—source data 2. [file elife-76183-fig3-data2.zip › Figure 3-source data 2/Figure 3B IP Myc WB pTyr.jpg]

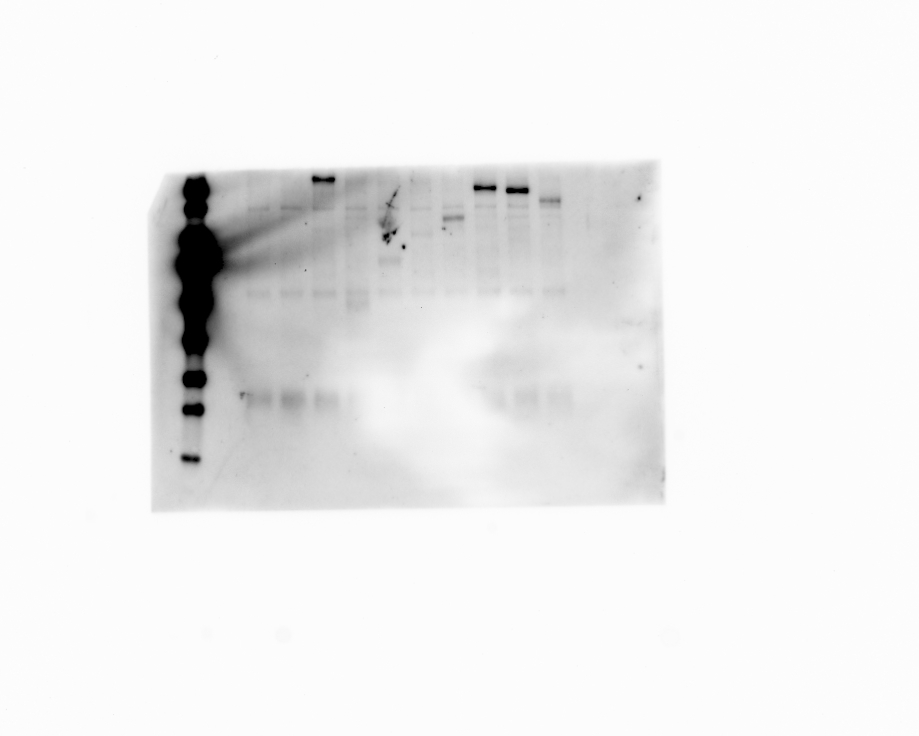

Supplement: Figure 3—source data 3. [file elife-76183-fig3-data3.zip › Figure 3-source data 3/Figure 3C IP-4G10.tif]

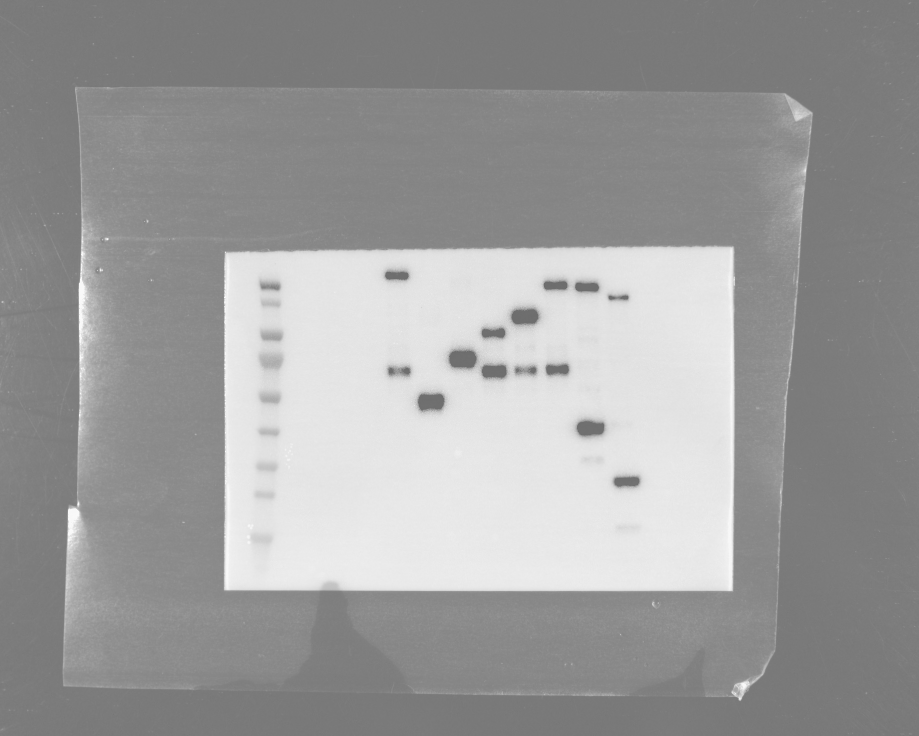

Supplement: Figure 3—source data 3. [file elife-76183-fig3-data3.zip › Figure 3-source data 3/Figure 3C IP-Myc.tif]

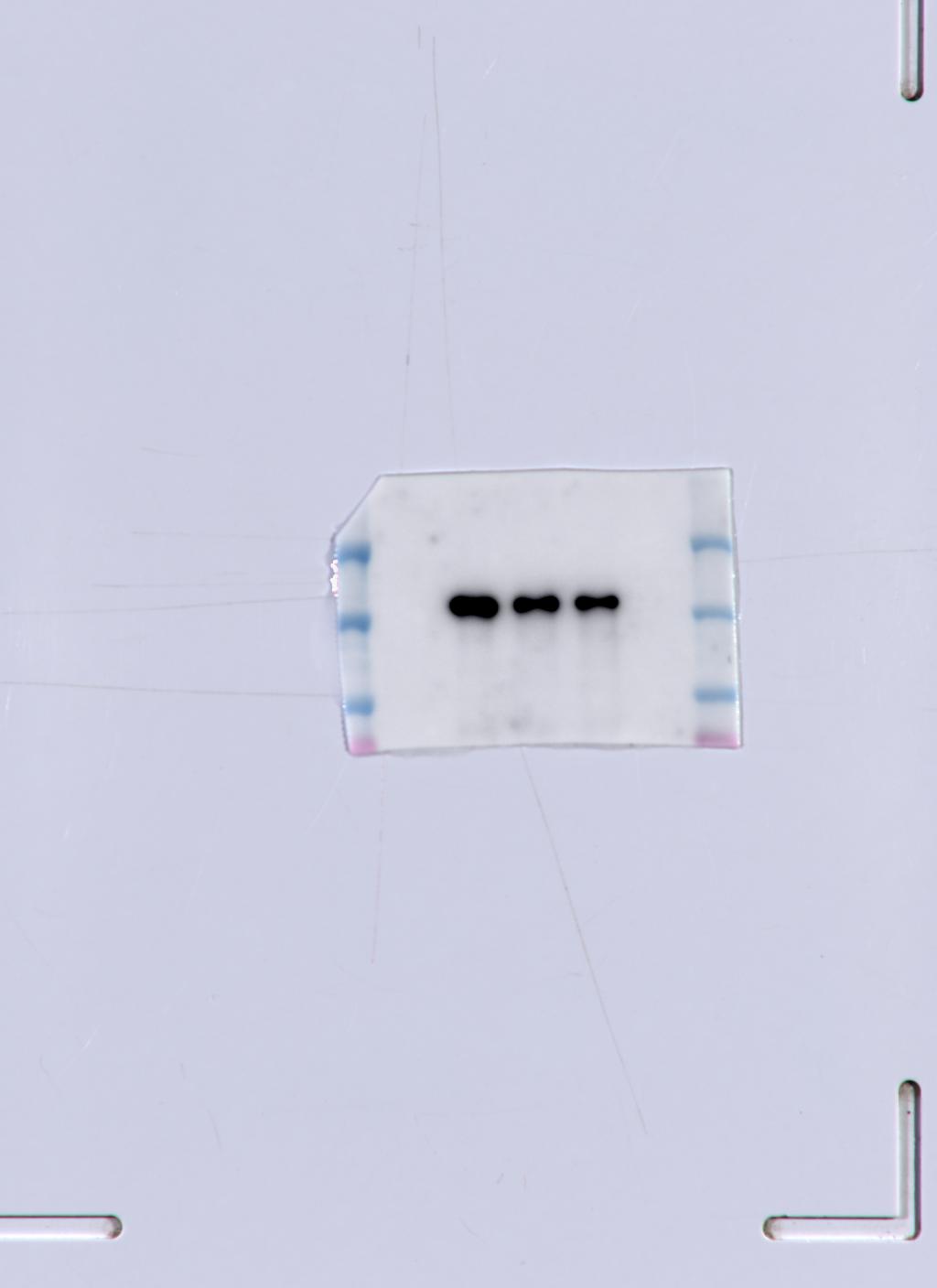

Supplement: Figure 3—source data 4. [file elife-76183-fig3-data4.zip › Figure 3-source data 4/Figure 3E IP Myc WB IRS4 Y921F.jpg]

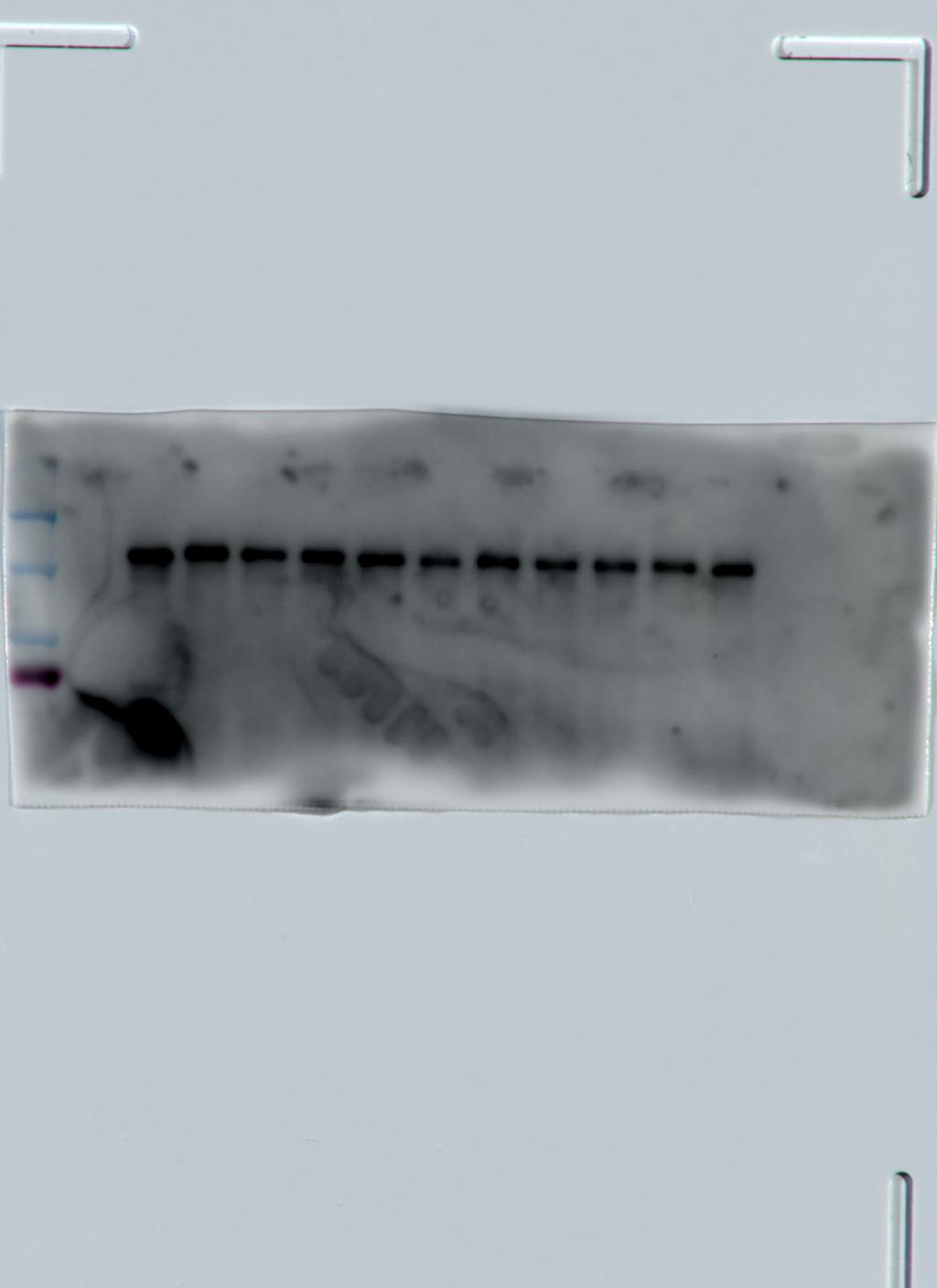

Supplement: Figure 3—source data 4. [file elife-76183-fig3-data4.zip › Figure 3-source data 4/Figure 3E IP Myc WB IRS4.jpg]

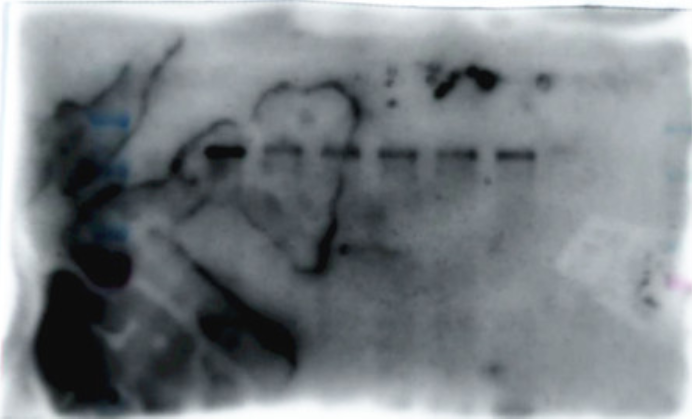

Supplement: Figure 3—source data 4. [file elife-76183-fig3-data4.zip › Figure 3-source data 4/Figure 3E IP Myc WB IRS4.png]

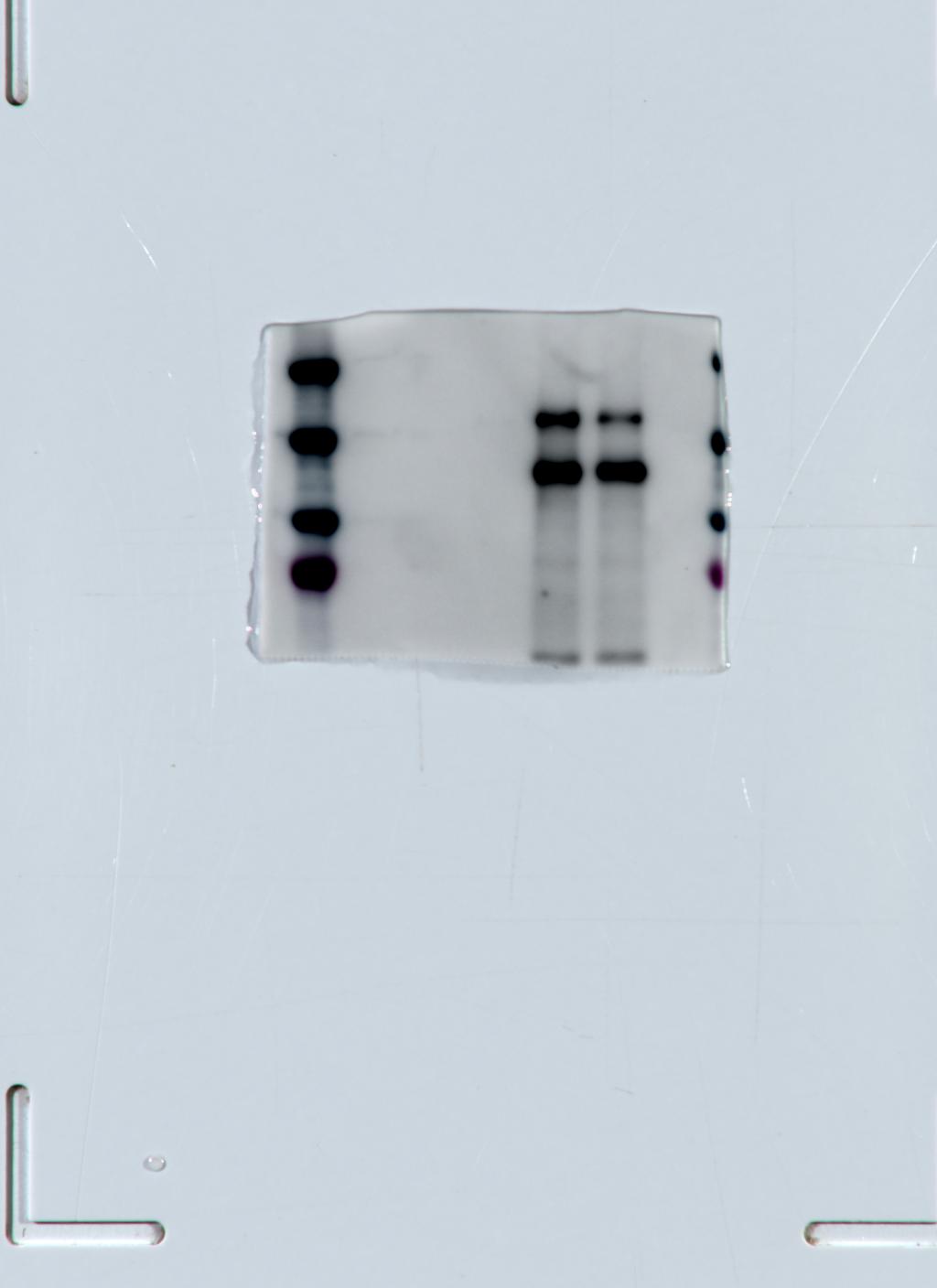

Supplement: Figure 3—source data 4. [file elife-76183-fig3-data4.zip › Figure 3-source data 4/Figure 3E IP Myc WB pTyr Y921F.jpg]

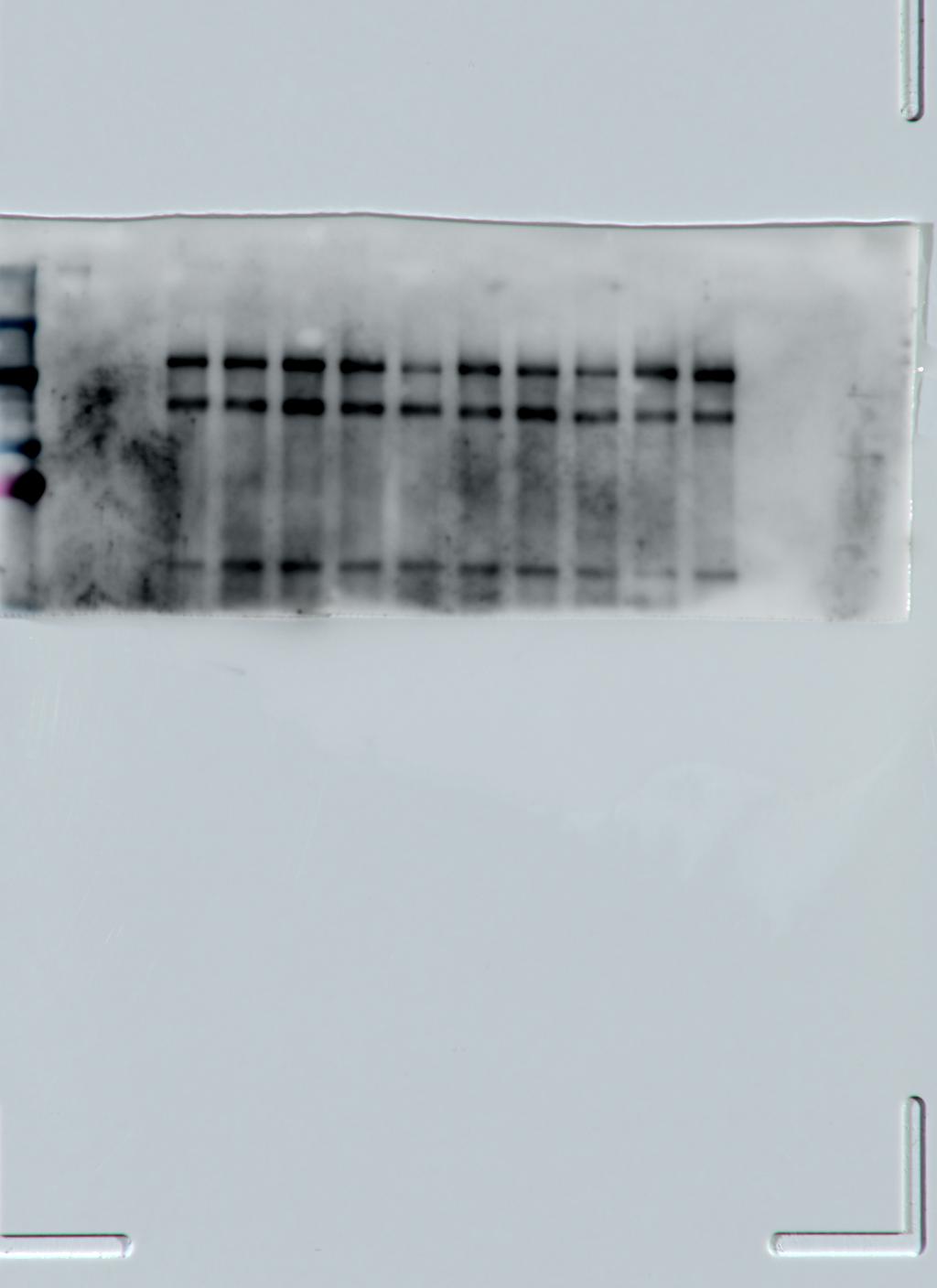

Supplement: Figure 3—source data 4. [file elife-76183-fig3-data4.zip › Figure 3-source data 4/Figure 3E IP Myc WB pTyr.jpg]

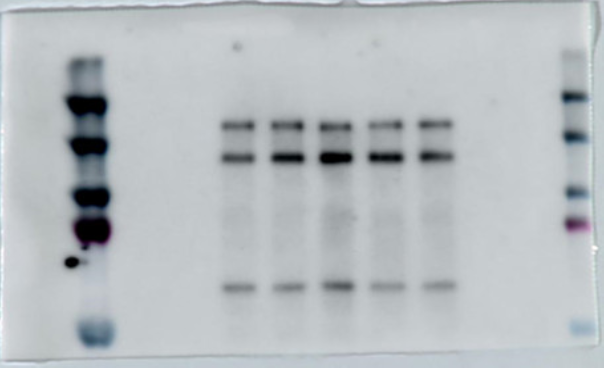

Supplement: Figure 3—source data 4. [file elife-76183-fig3-data4.zip › Figure 3-source data 4/Figure 3E IP Myc WB pTyr.png]

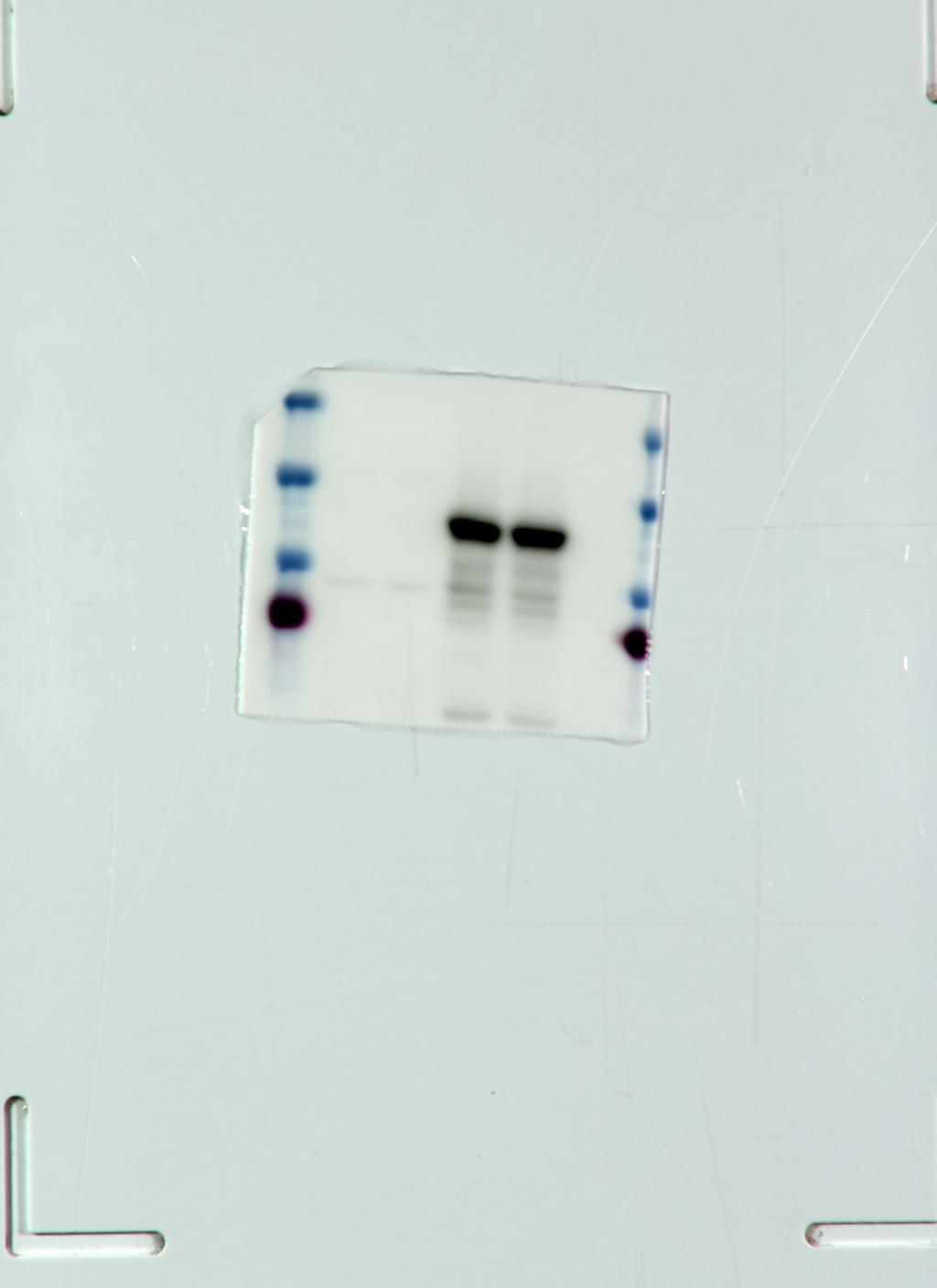

Supplement: Figure 3—source data 5. [file elife-76183-fig3-data5.zip › Figure 3-source data 5/Figure 3F Input WB FER.jpg]

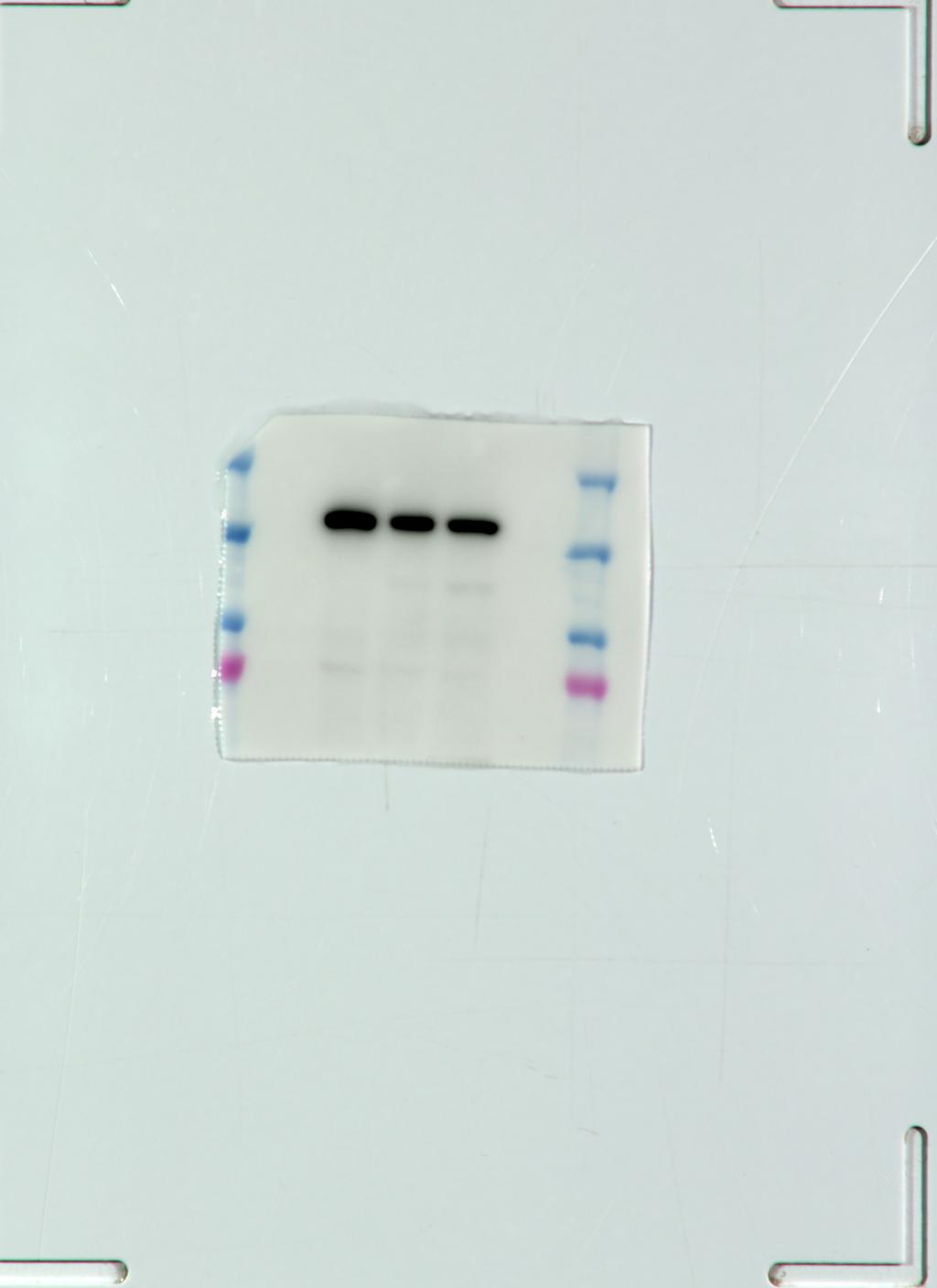

Supplement: Figure 3—source data 5. [file elife-76183-fig3-data5.zip › Figure 3-source data 5/Figure 3F Input WB Myc.jpg]

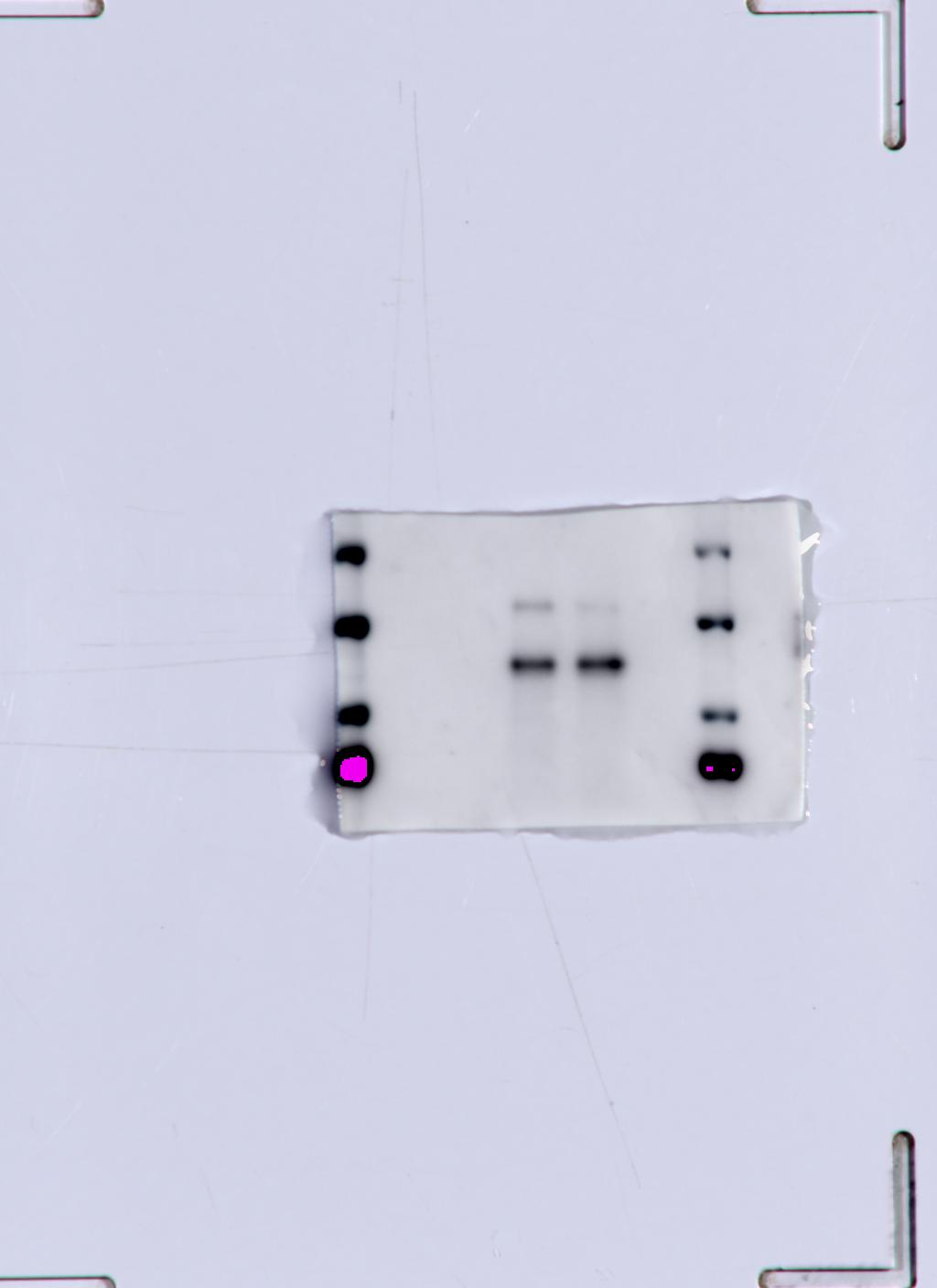

Supplement: Figure 3—source data 5. [file elife-76183-fig3-data5.zip › Figure 3-source data 5/Figure 3F IP Myc WB FER.jpg]

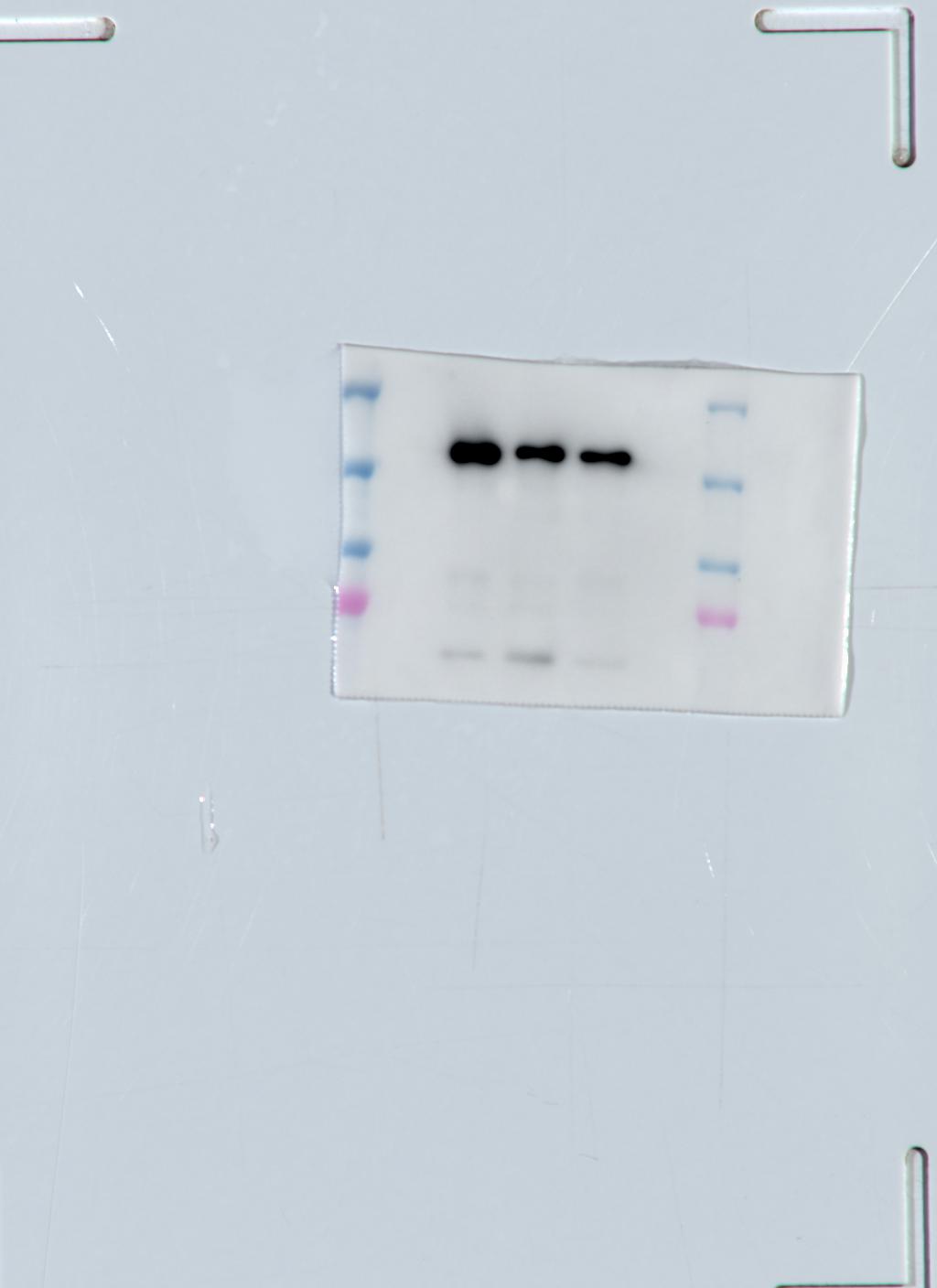

Supplement: Figure 3—source data 5. [file elife-76183-fig3-data5.zip › Figure 3-source data 5/Figure 3F IP Myc WB IRS4.jpg]

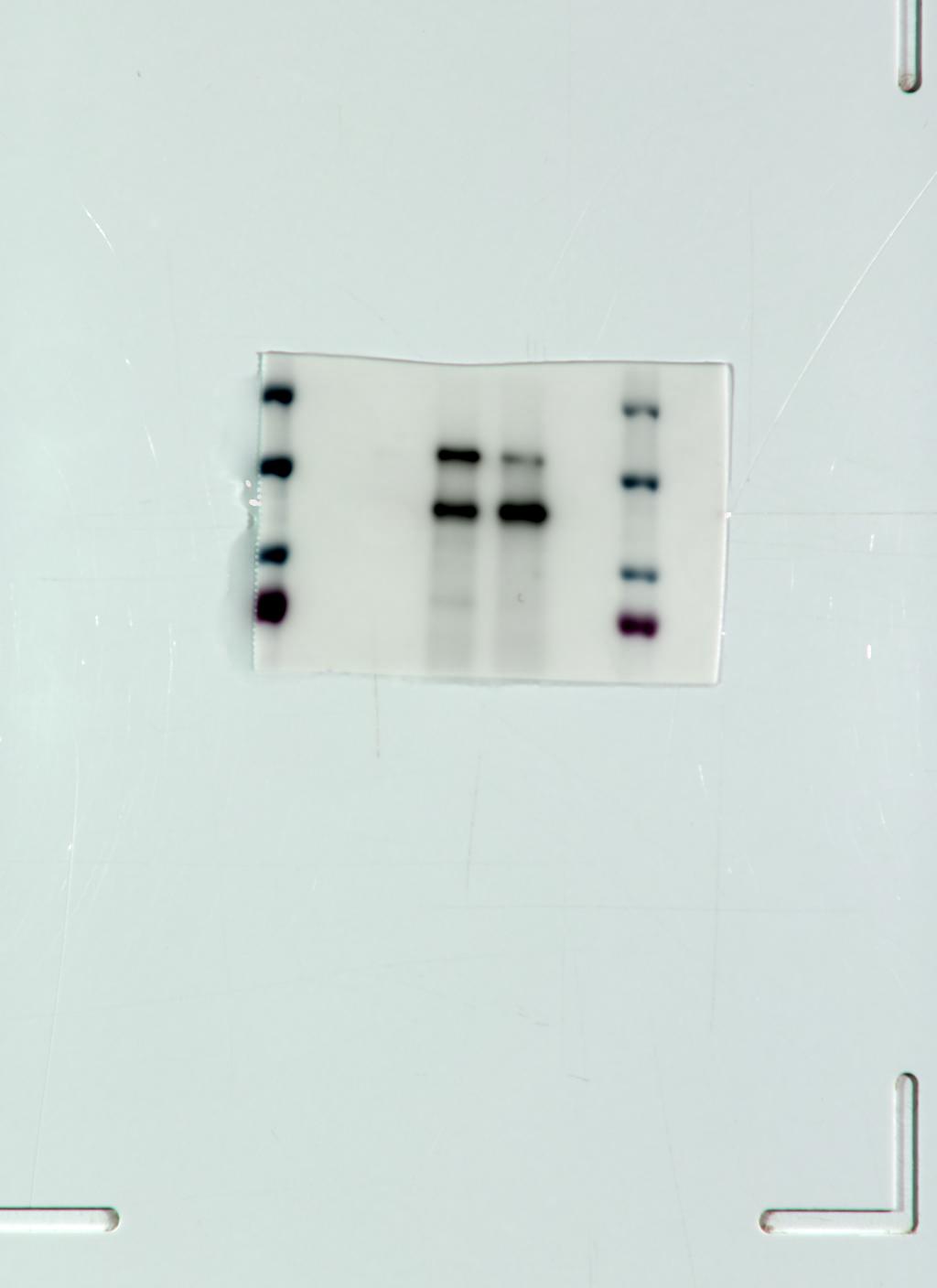

Supplement: Figure 3—source data 5. [file elife-76183-fig3-data5.zip › Figure 3-source data 5/Figure 3F IP Myc WB pTyr.jpg]
