# Supplementary figures and images for: FER-mediated phosphorylation and PIK3R2 recruitment on IRS4 promotes AKT activation and tumorigenesis in ovarian cancer cells (part 2 of 3)
Source: eLife. 2022 May 12;11:e76183. doi: 10.7554/eLife.76183 (PMC9098222; doi:10.7554/eLife.76183)

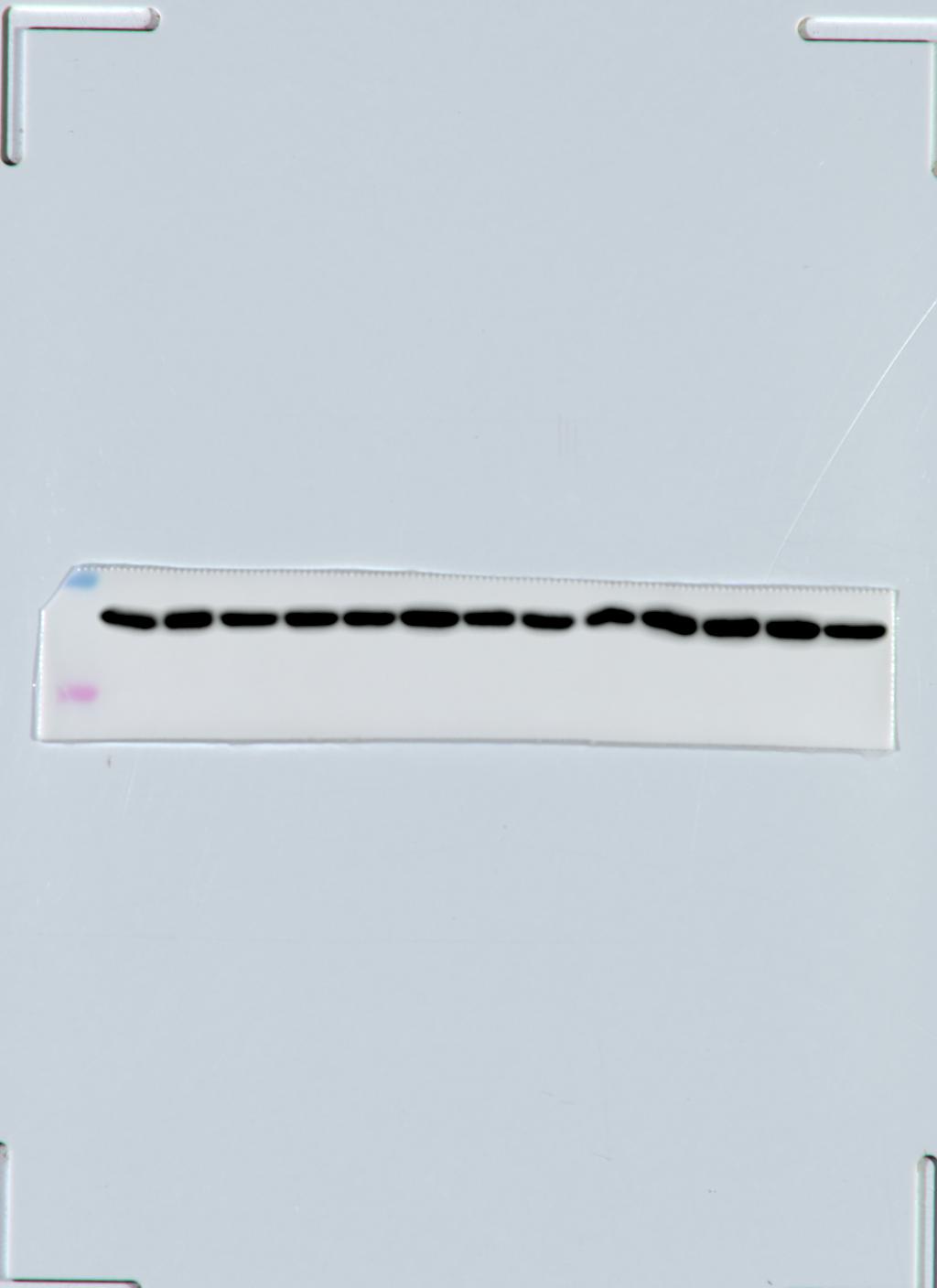

Supplement: Figure 4—source data 1. [file elife-76183-fig4-data1.zip › Figure 4-source data 1/Figure 4A left GAPDH.jpg]

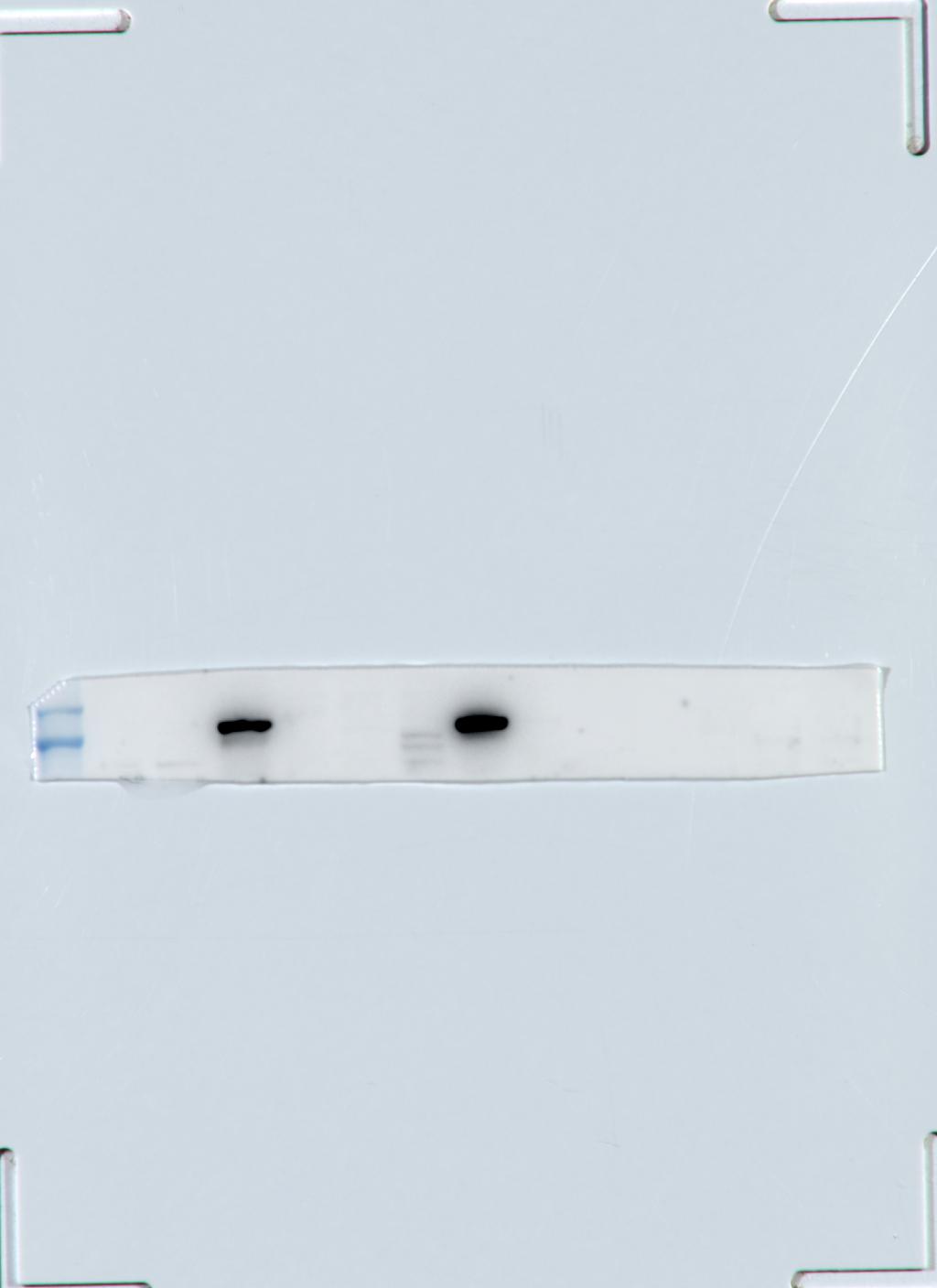

Supplement: Figure 4—source data 1. [file elife-76183-fig4-data1.zip › Figure 4-source data 1/Figure 4A left IRS4.jpg]

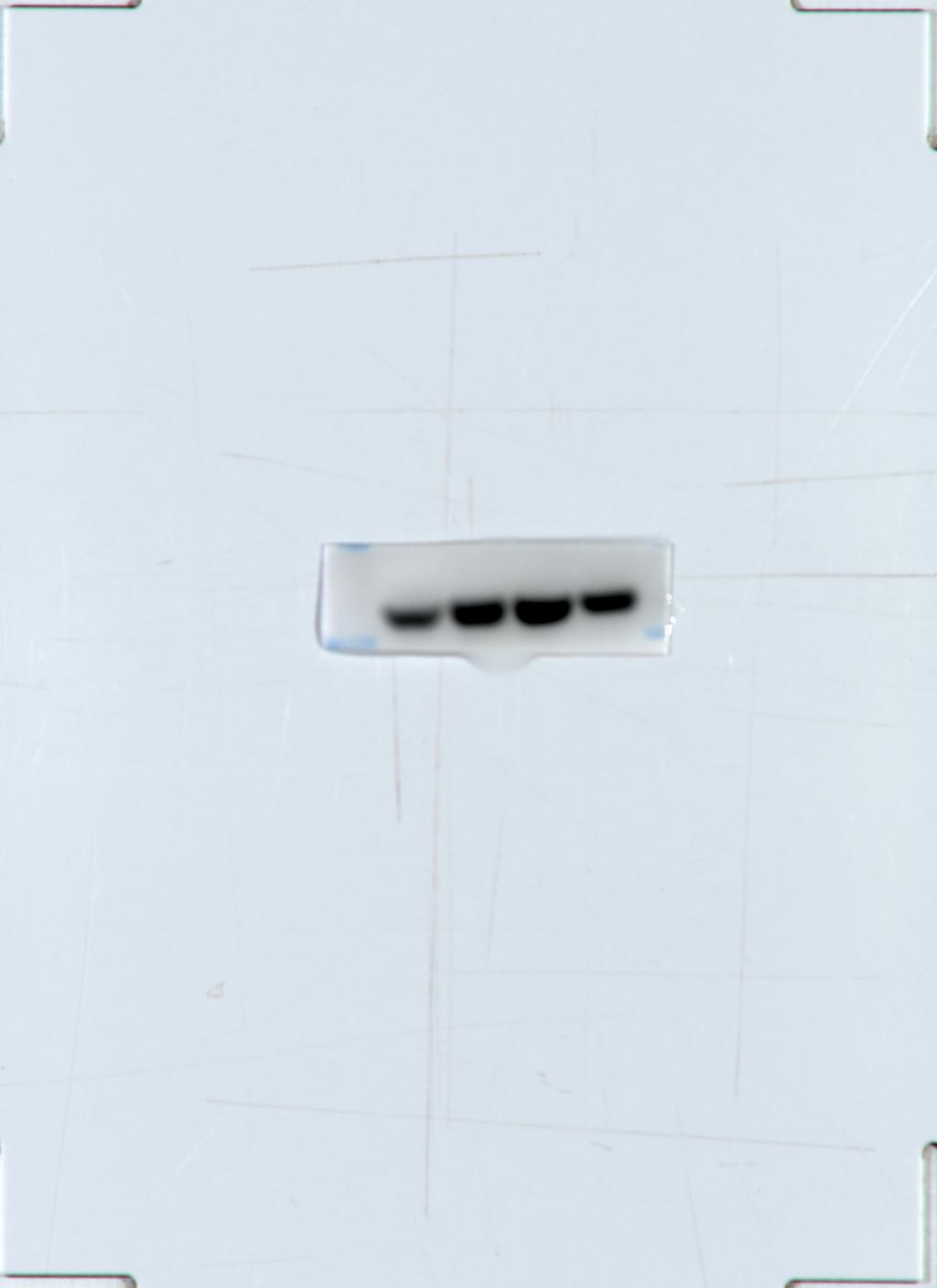

Supplement: Figure 4—source data 1. [file elife-76183-fig4-data1.zip › Figure 4-source data 1/Figure 4A right Actin.jpg]

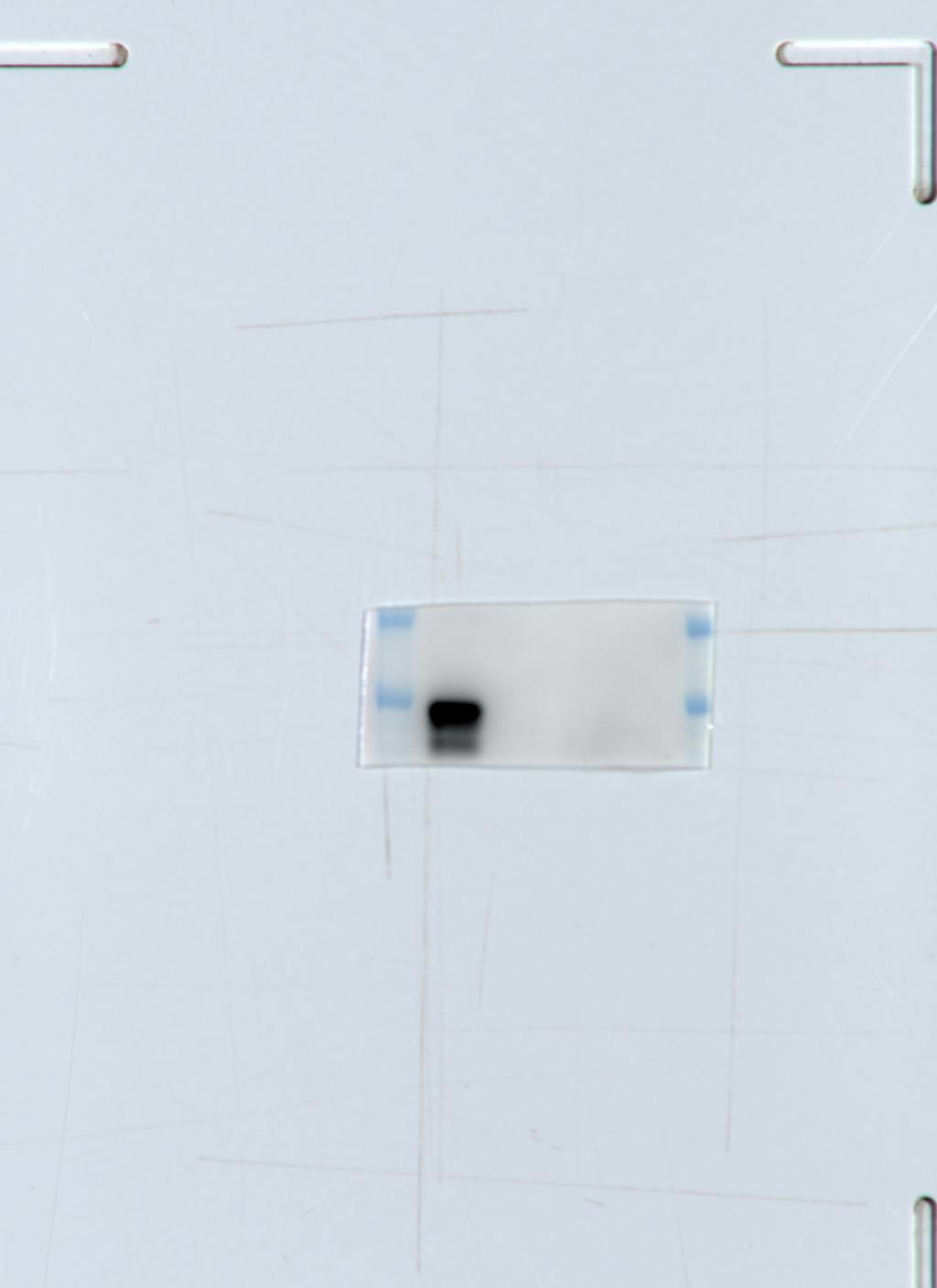

Supplement: Figure 4—source data 1. [file elife-76183-fig4-data1.zip › Figure 4-source data 1/Figure 4A right IRS4.jpg]

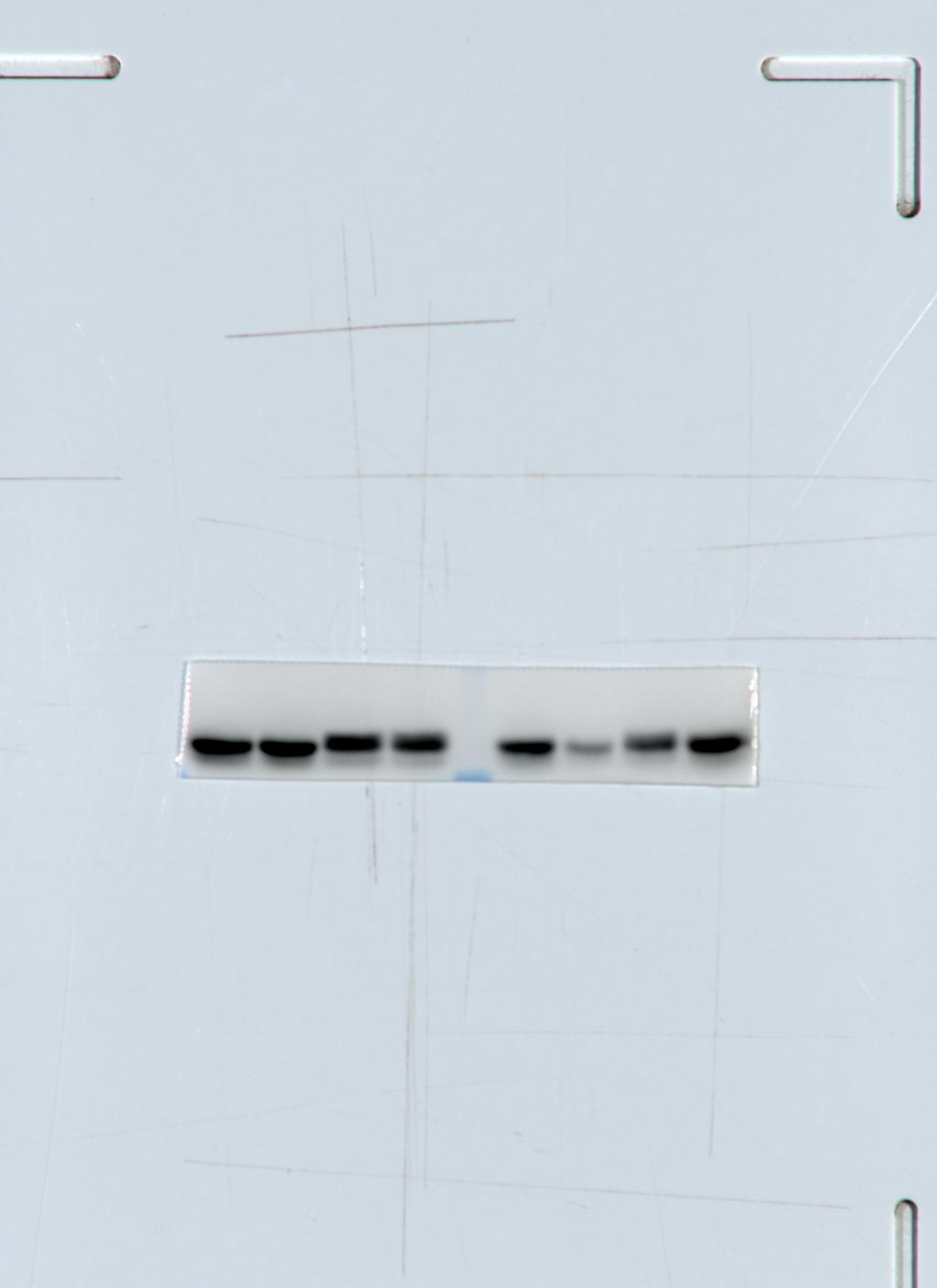

Supplement: Figure 4—source data 2. [file elife-76183-fig4-data2.zip › Figure 4-source data 2/Figure 4B AKT.jpg]

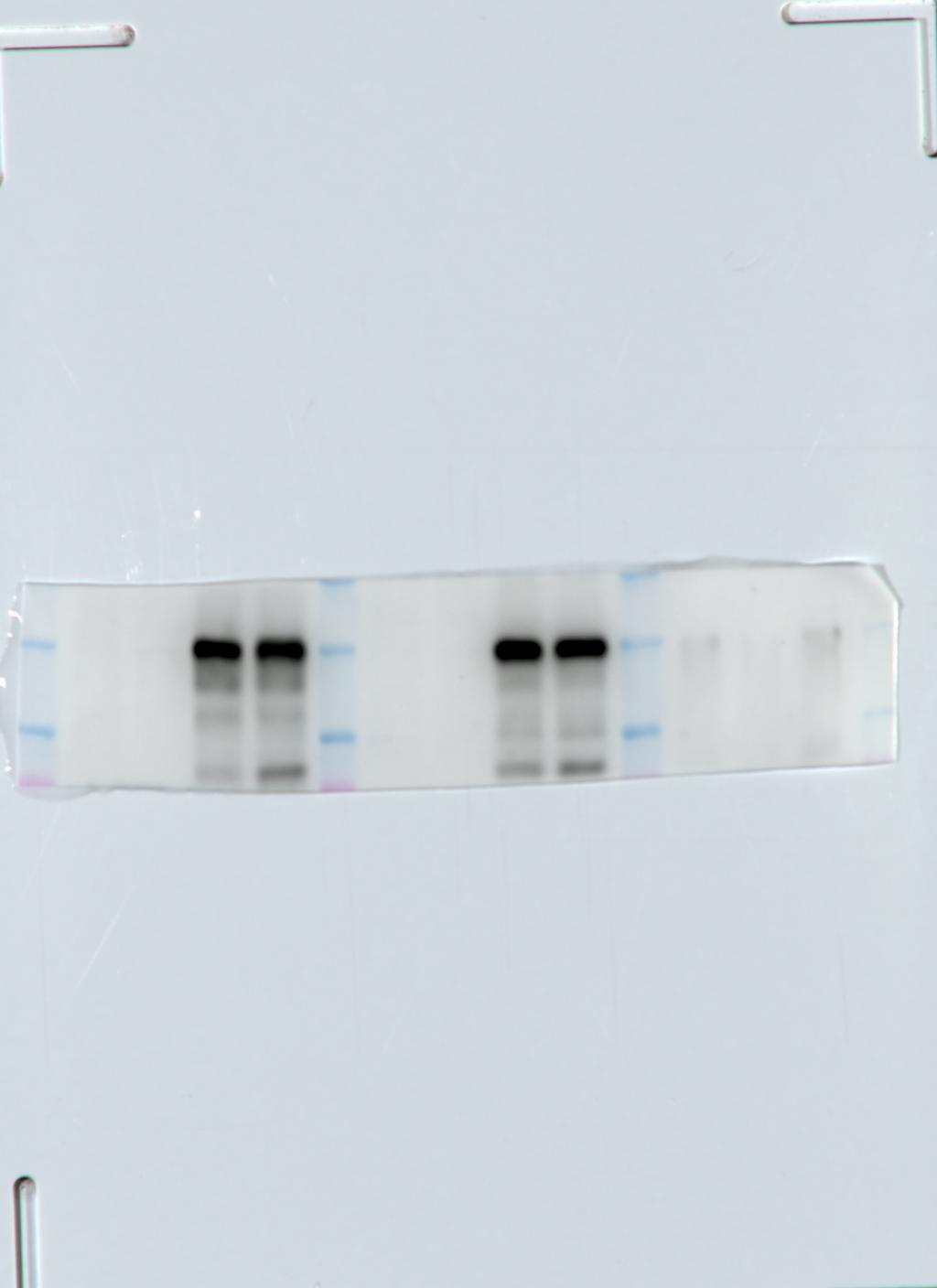

Supplement: Figure 4—source data 2. [file elife-76183-fig4-data2.zip › Figure 4-source data 2/Figure 4B IRS4.jpg]

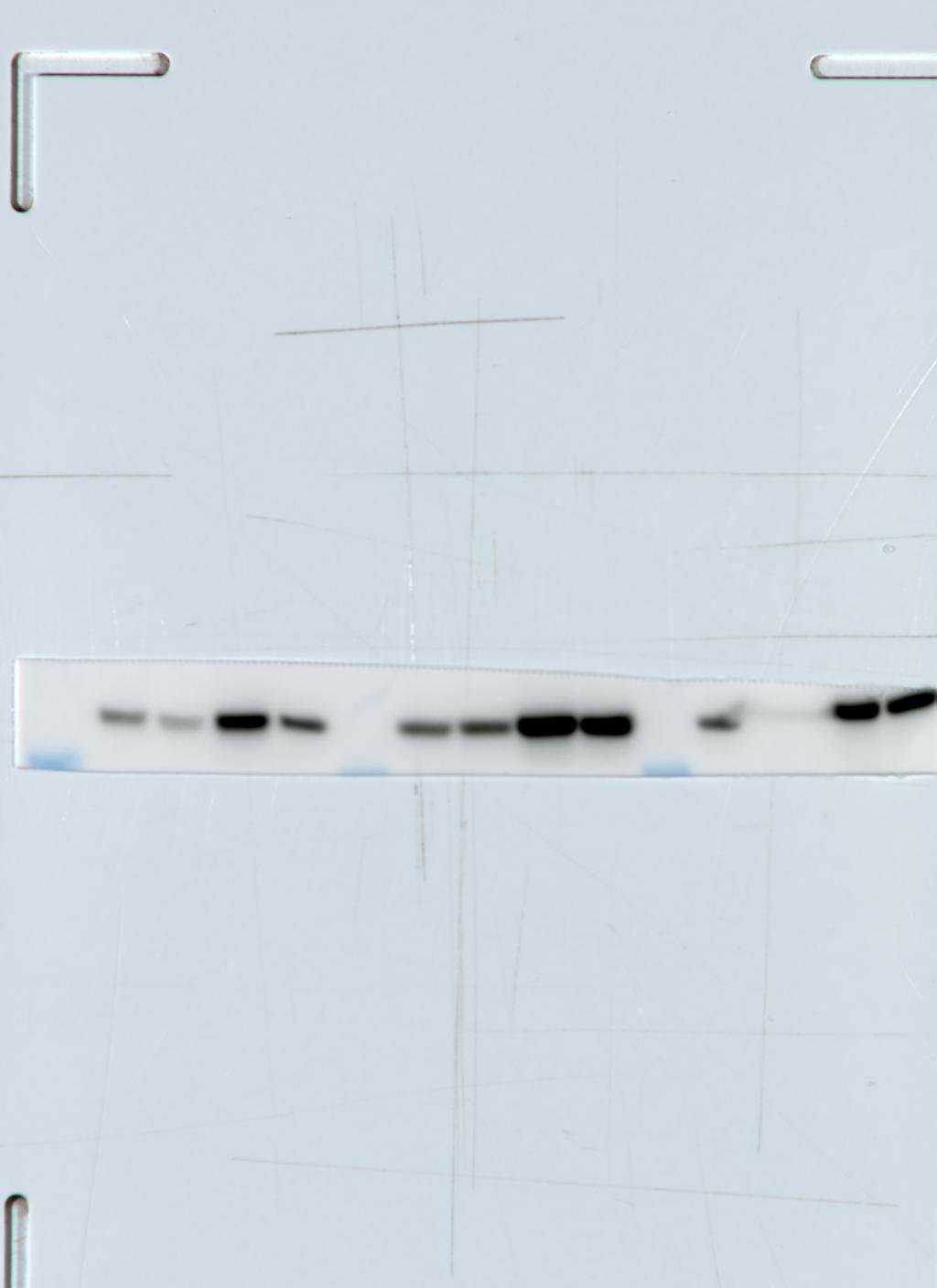

Supplement: Figure 4—source data 2. [file elife-76183-fig4-data2.zip › Figure 4-source data 2/Figure 4B pS473 AKT.jpg]

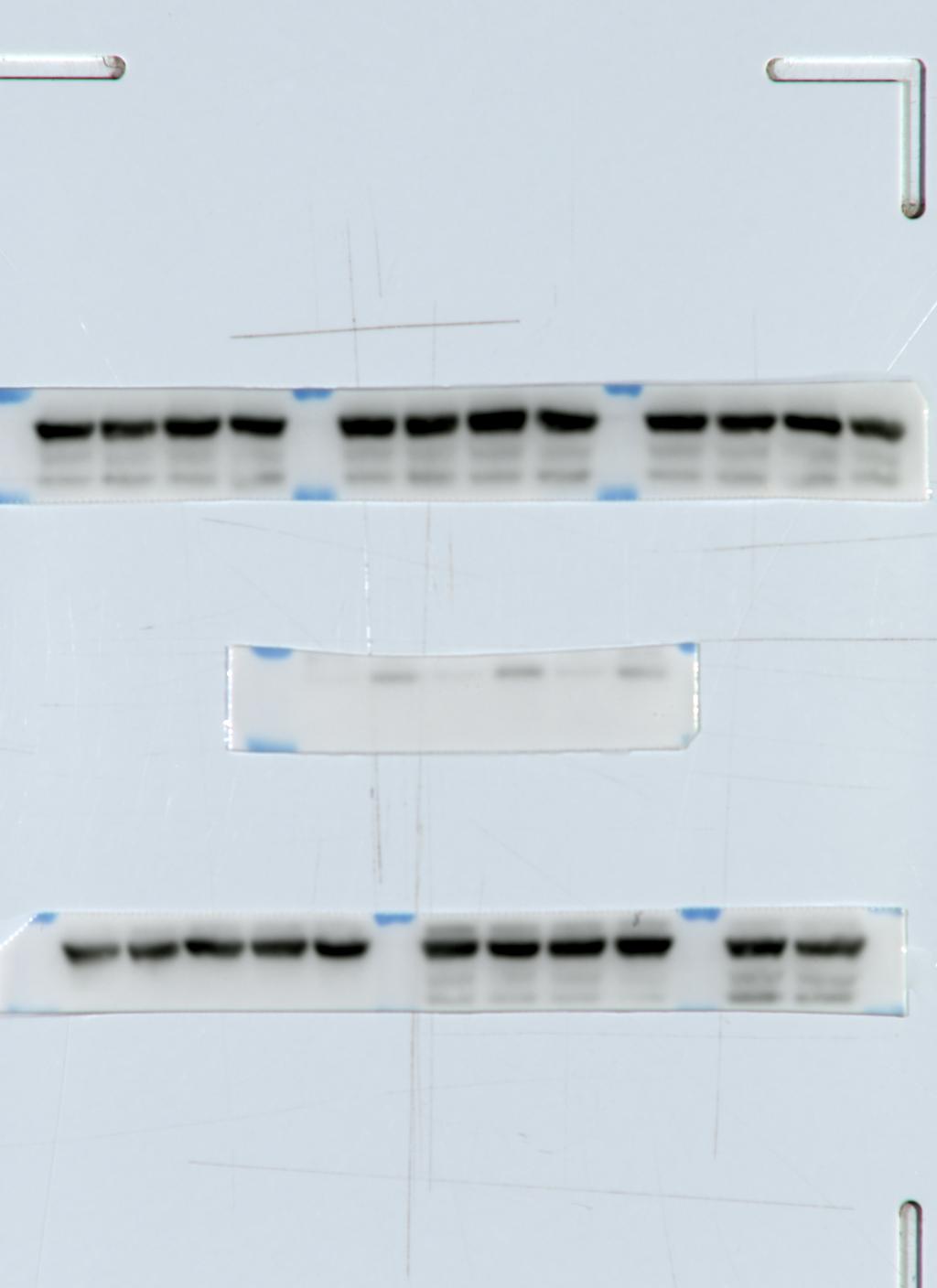

Supplement: Figure 4—source data 2. [file elife-76183-fig4-data2.zip › Figure 4-source data 2/Figure 4B Tubulin.jpg]

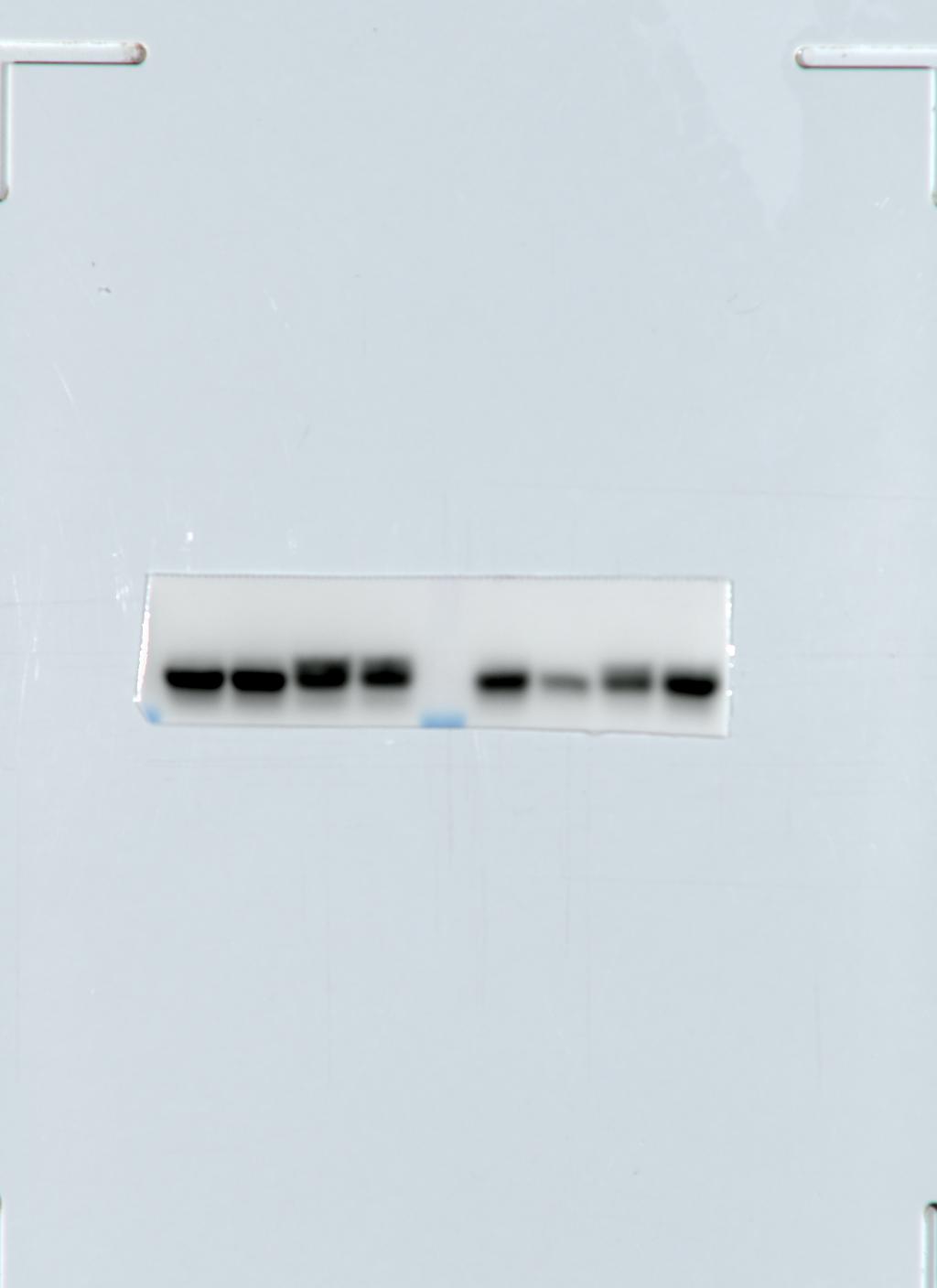

Supplement: Figure 4—source data 3. [file elife-76183-fig4-data3.zip › Figure 4-source data 3/Figue 4D AKT.jpg]

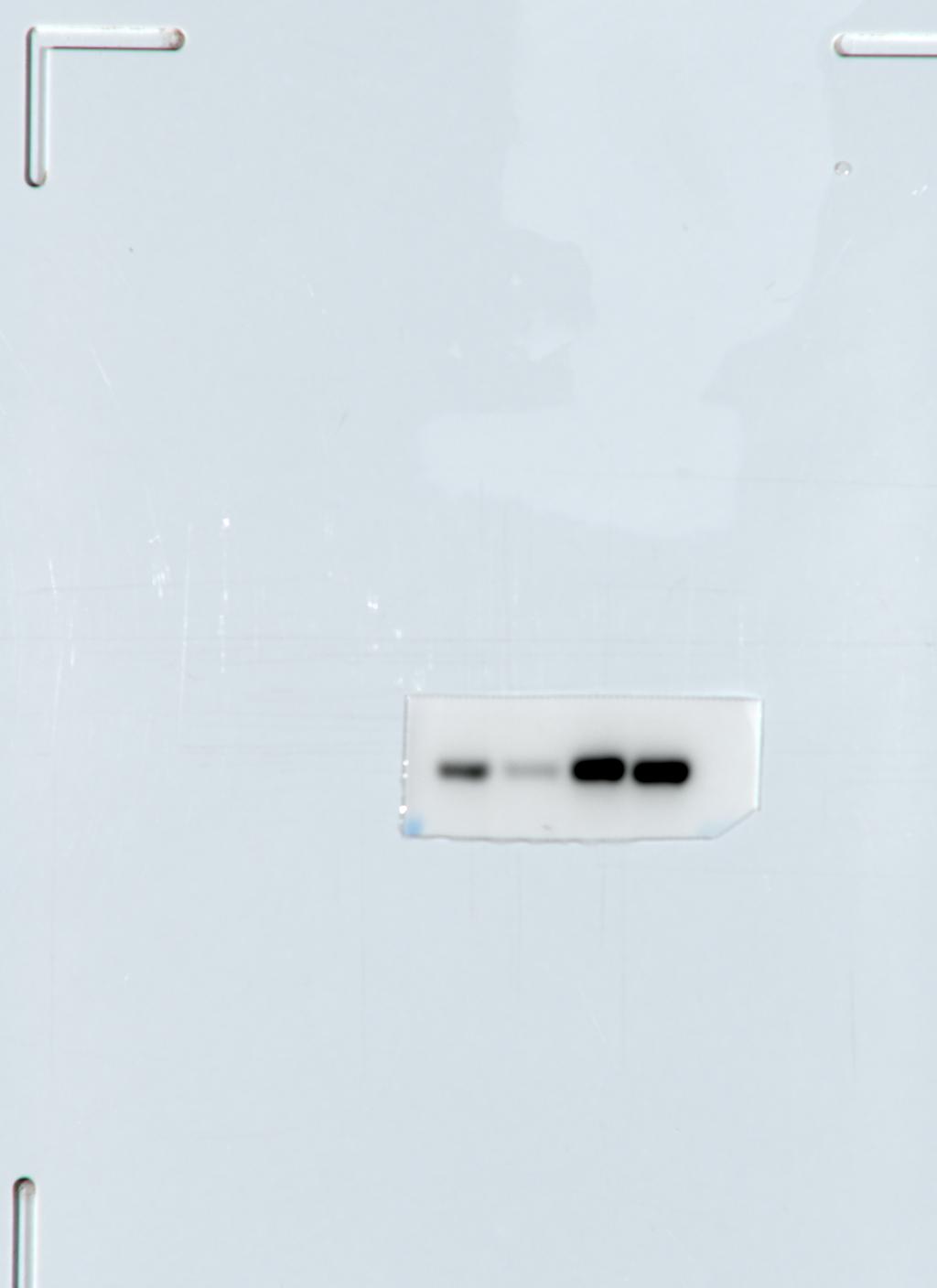

Supplement: Figure 4—source data 3. [file elife-76183-fig4-data3.zip › Figure 4-source data 3/Figue 4D pS473 AKT.jpg]

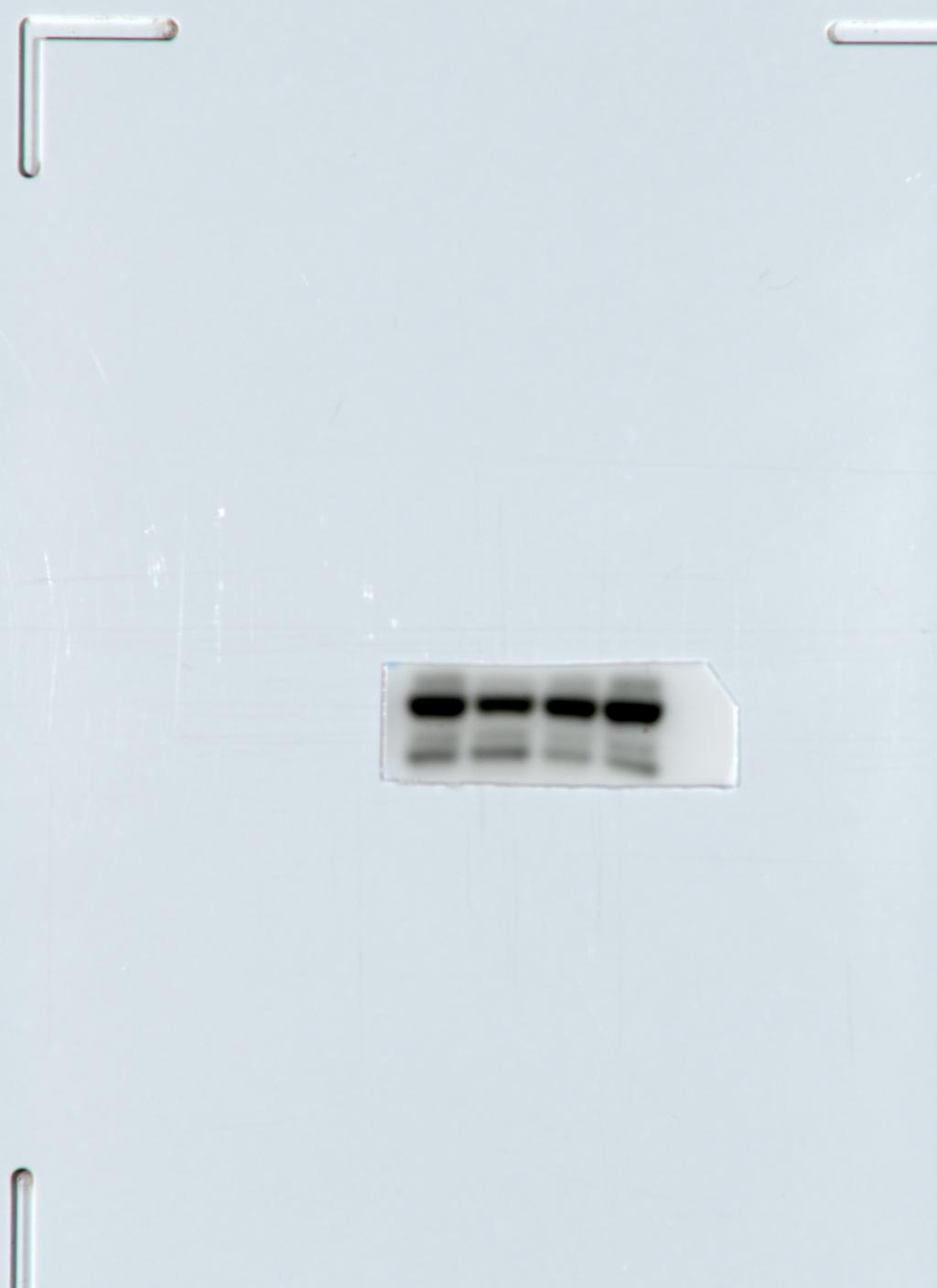

Supplement: Figure 4—source data 3. [file elife-76183-fig4-data3.zip › Figure 4-source data 3/Figue 4D Tubulin.jpg]

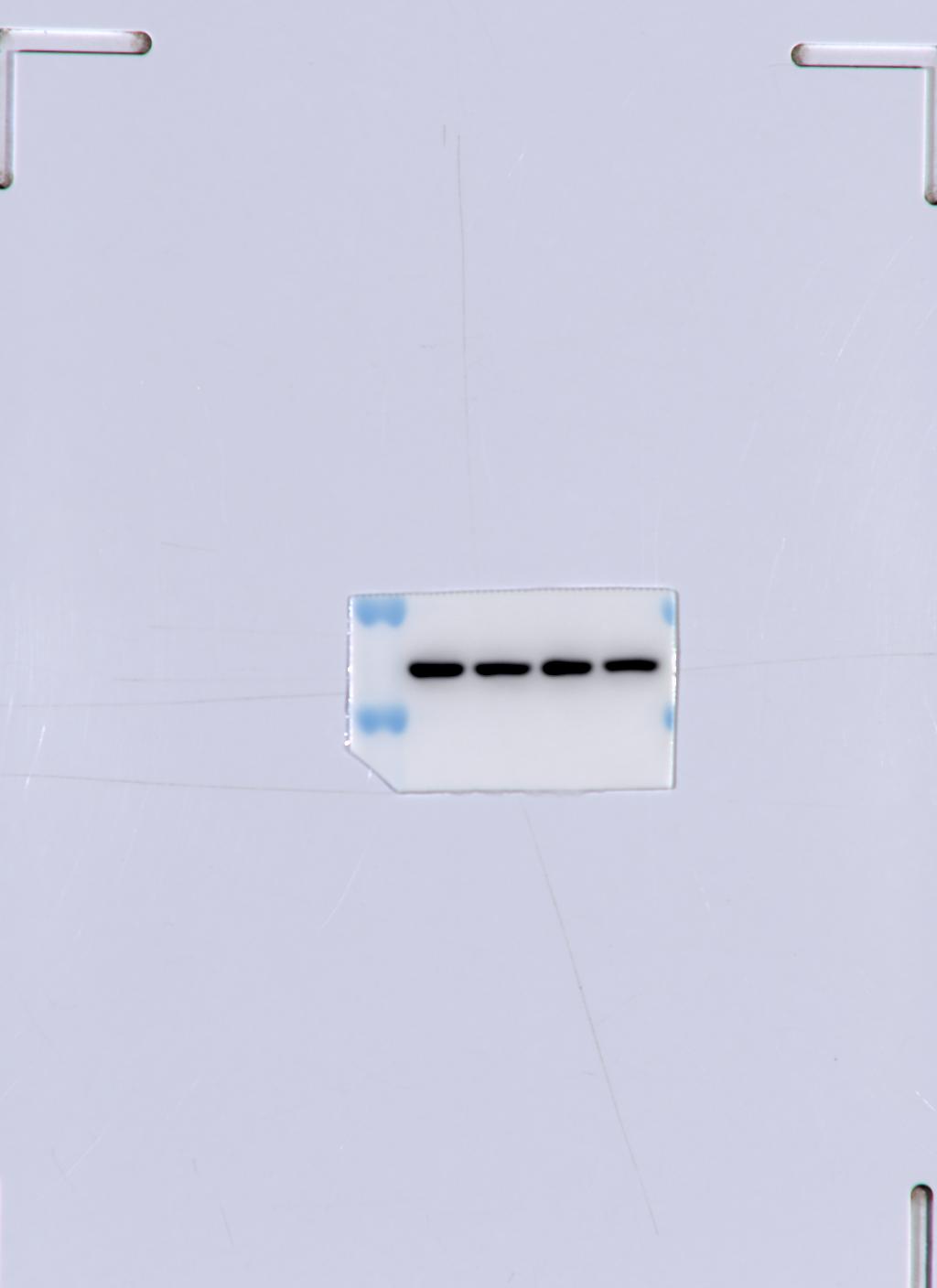

Supplement: Figure 4—source data 4. [file elife-76183-fig4-data4.zip › Figure 4-source data 4/Figure 6D INPUT-Actin.jpg]

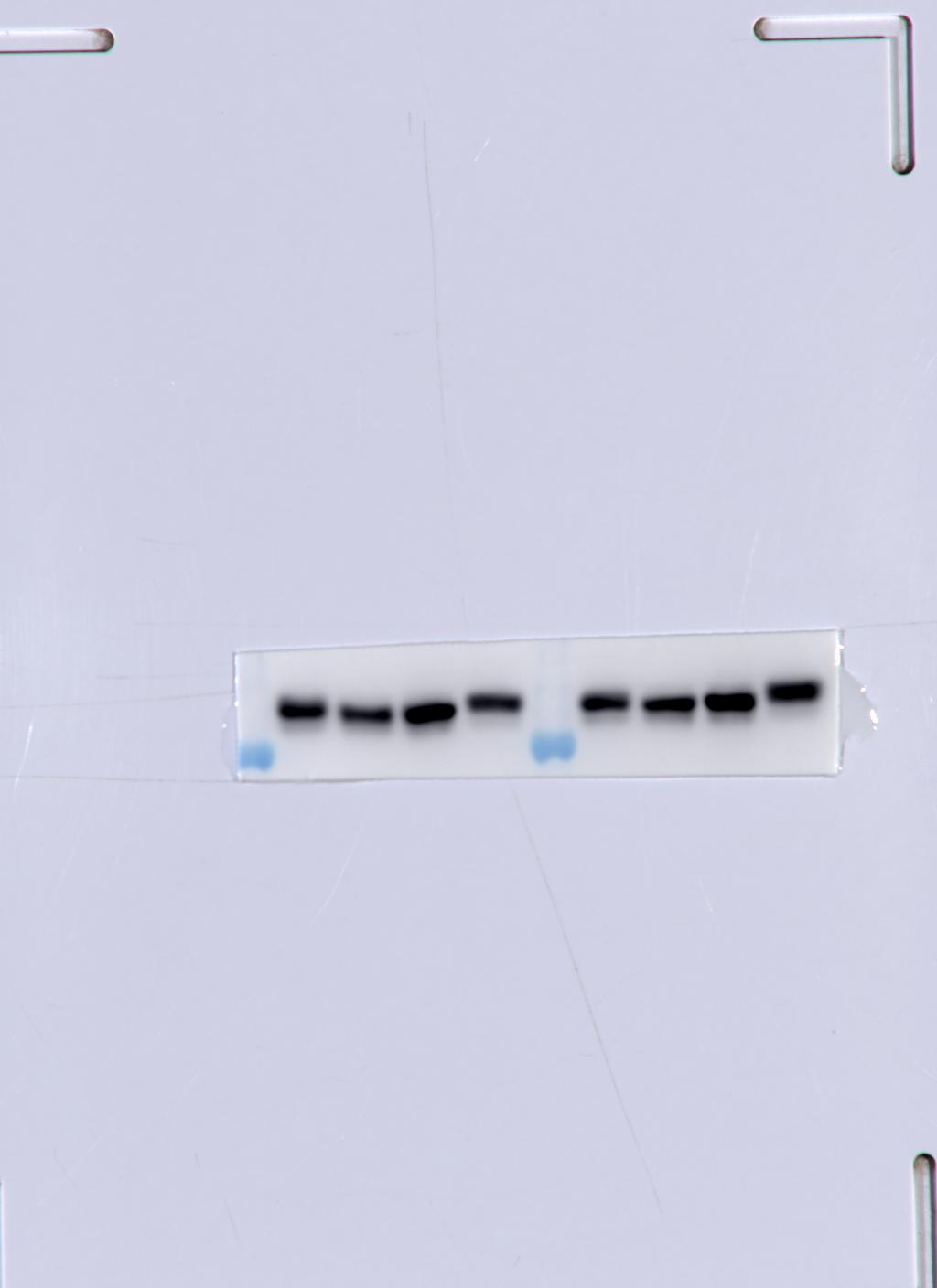

Supplement: Figure 4—source data 4. [file elife-76183-fig4-data4.zip › Figure 4-source data 4/Figure 6D INPUT-AKT.jpg]

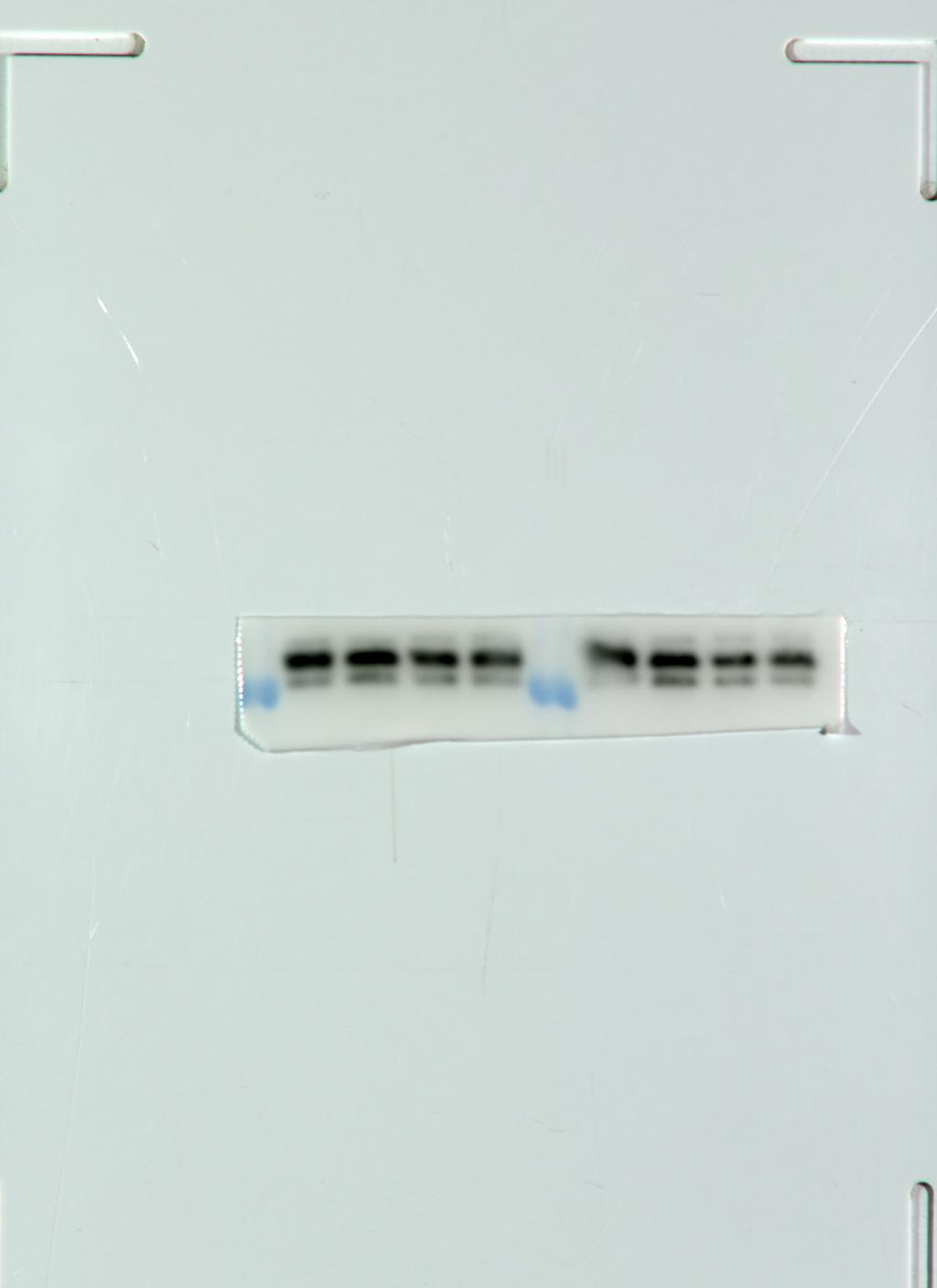

Supplement: Figure 4—source data 4. [file elife-76183-fig4-data4.zip › Figure 4-source data 4/Figure 6D INPUT-ERK1-2.jpg]

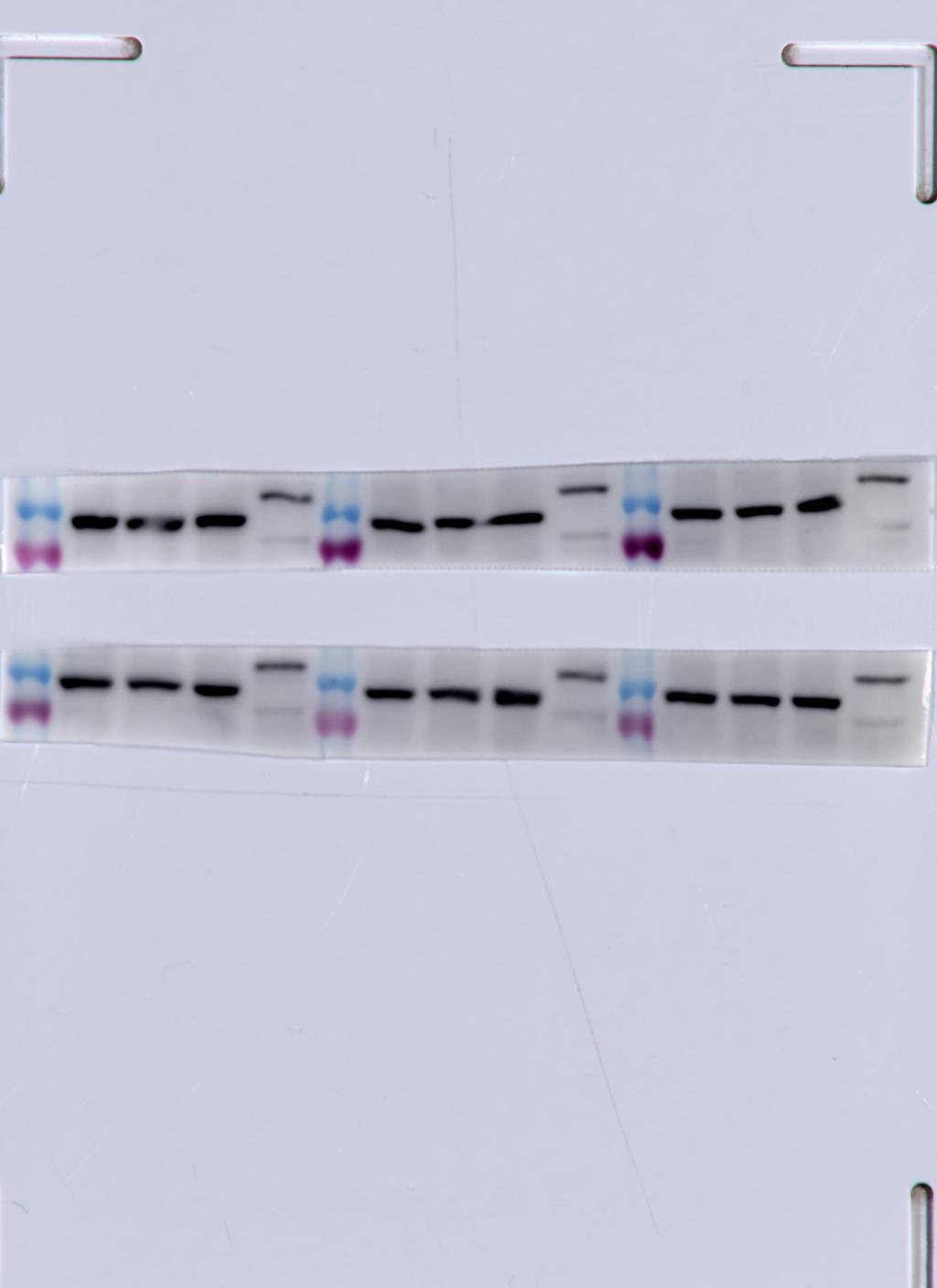

Supplement: Figure 4—source data 4. [file elife-76183-fig4-data4.zip › Figure 4-source data 4/Figure 6D INPUT-FER.jpg]

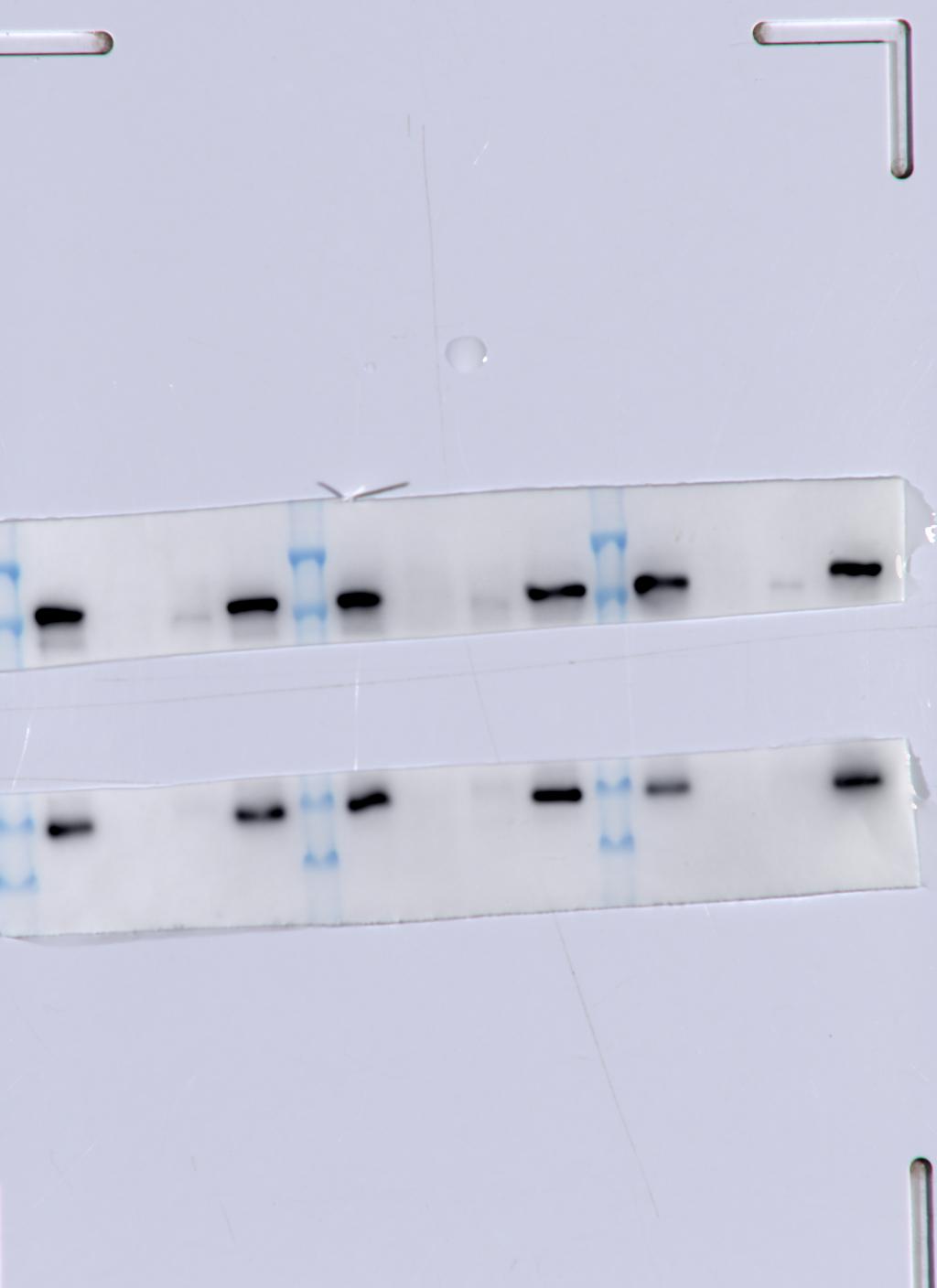

Supplement: Figure 4—source data 4. [file elife-76183-fig4-data4.zip › Figure 4-source data 4/Figure 6D INPUT-IRS4.jpg]

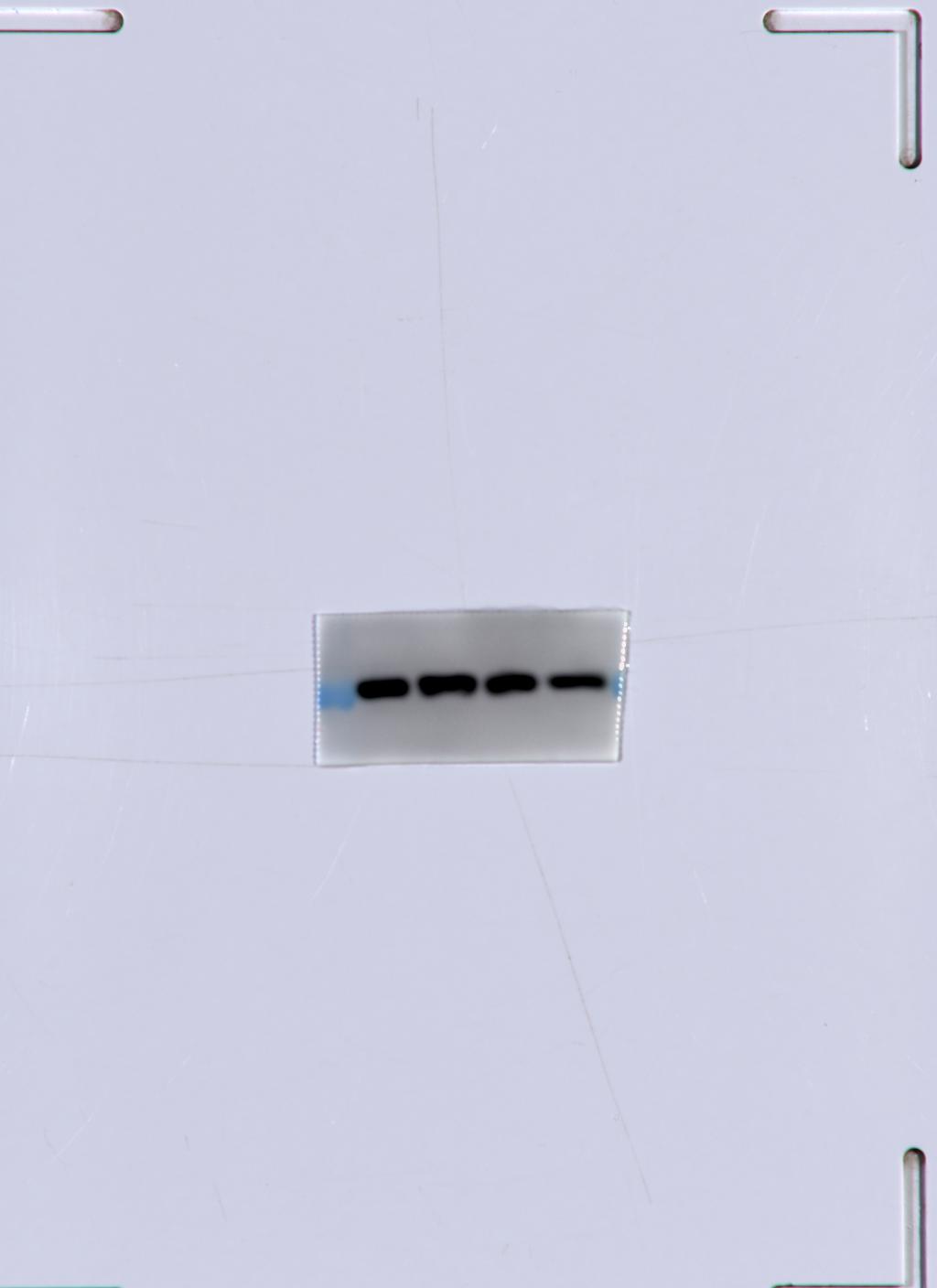

Supplement: Figure 4—source data 4. [file elife-76183-fig4-data4.zip › Figure 4-source data 4/Figure 6D INPUT-p38.jpg]

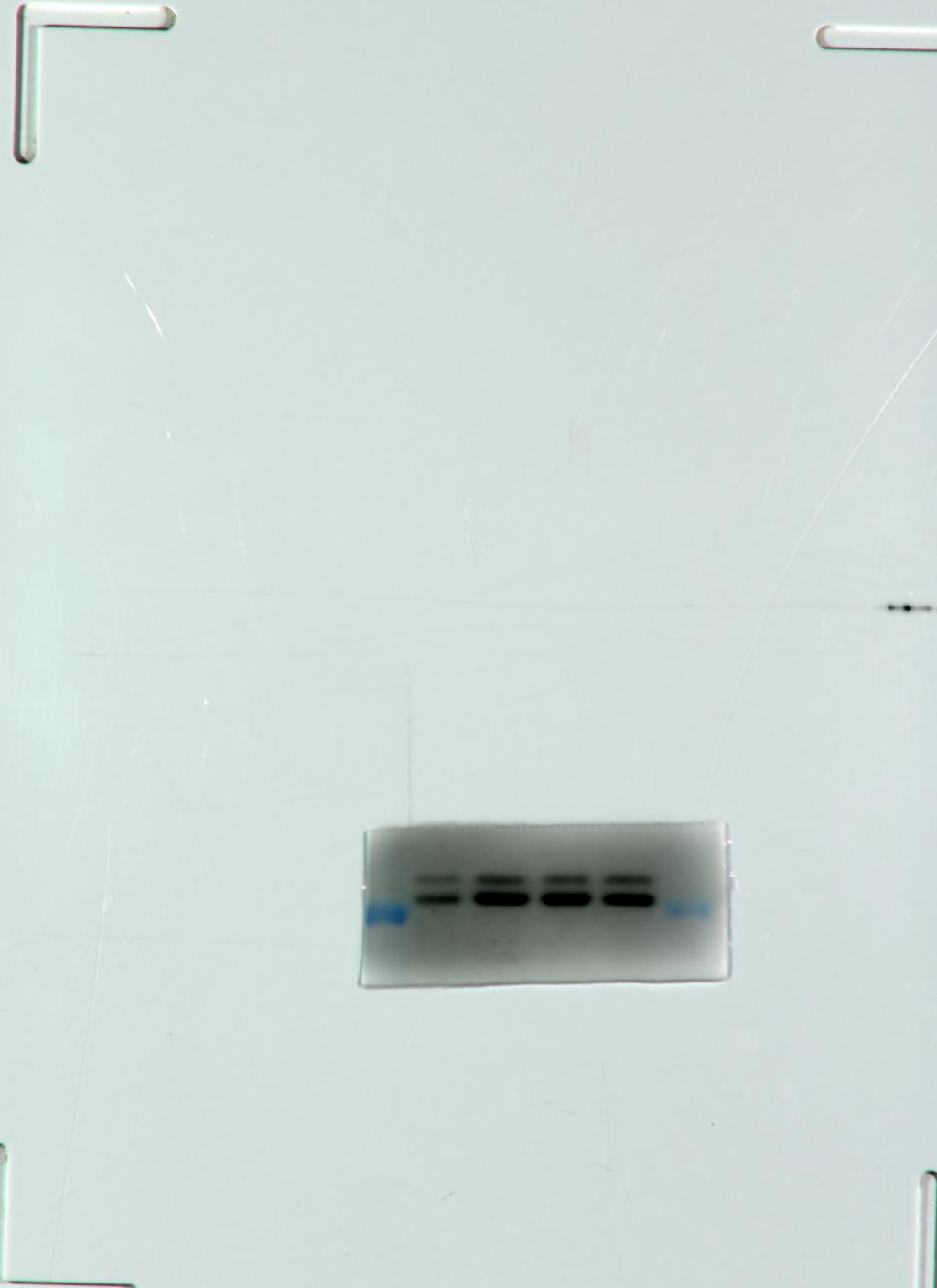

Supplement: Figure 4—source data 4. [file elife-76183-fig4-data4.zip › Figure 4-source data 4/Figure 6D INPUT-pERK1-2.jpg]

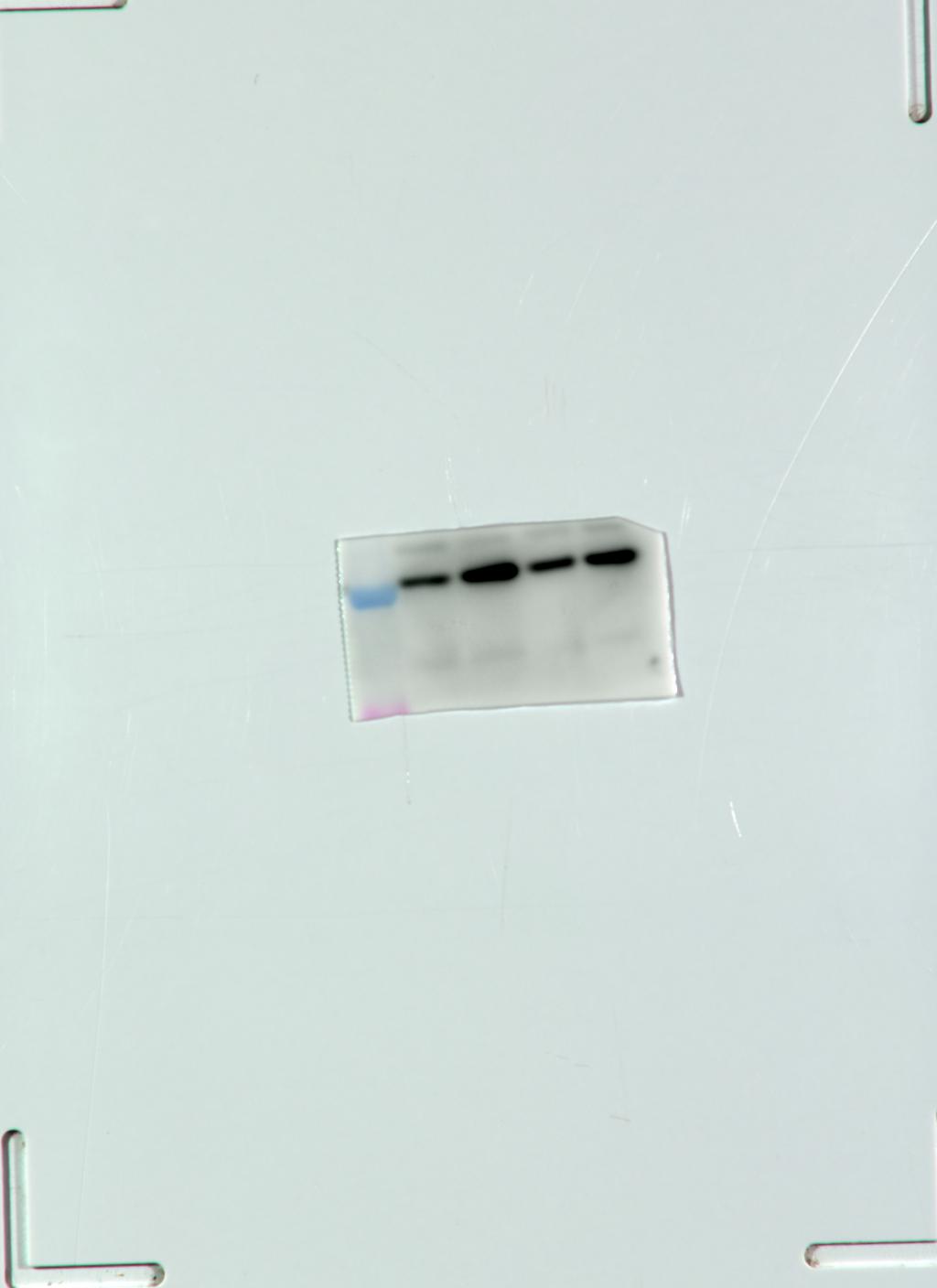

Supplement: Figure 4—source data 4. [file elife-76183-fig4-data4.zip › Figure 4-source data 4/Figure 6D INPUT-pp38.jpg]

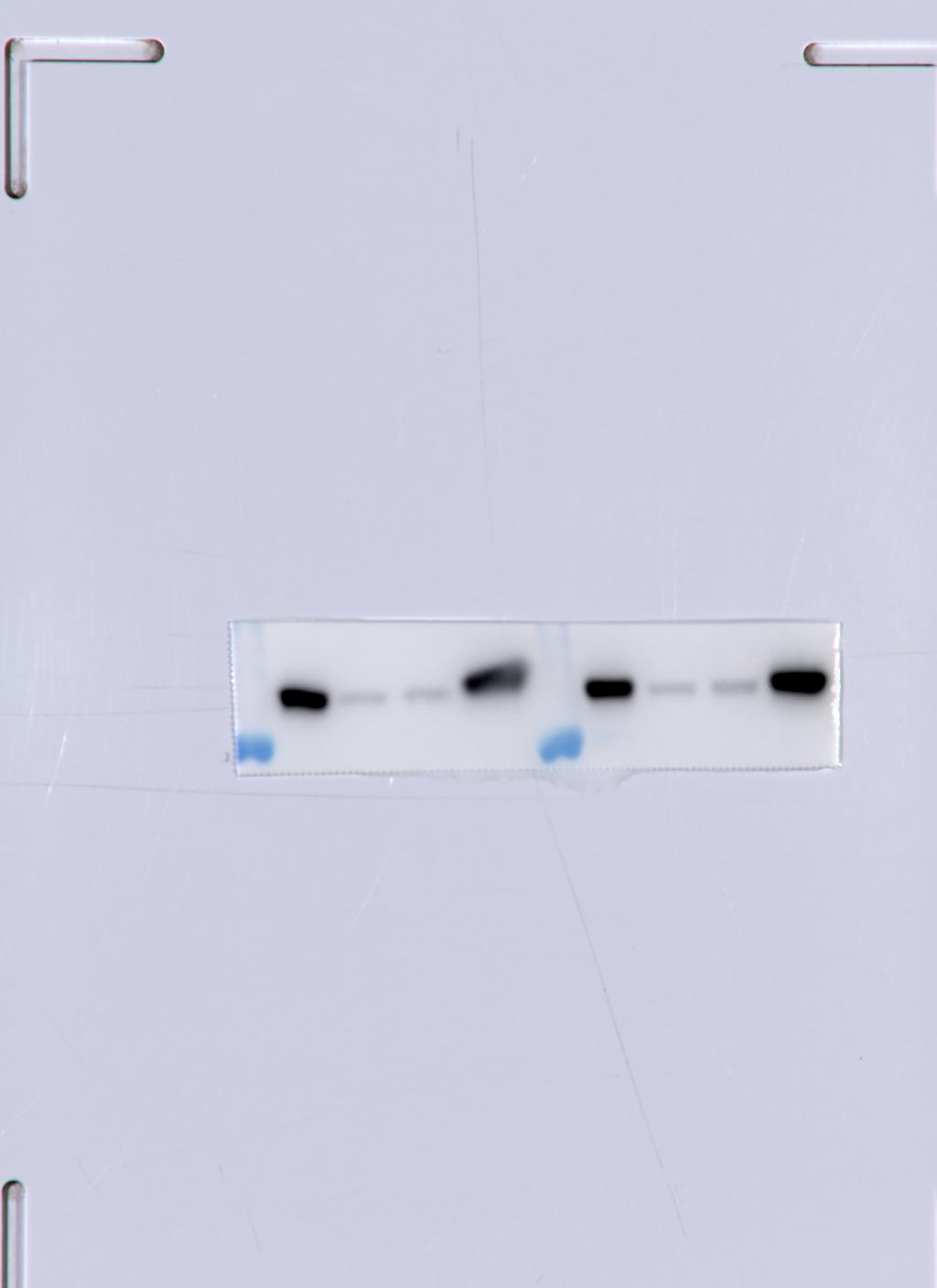

Supplement: Figure 4—source data 4. [file elife-76183-fig4-data4.zip › Figure 4-source data 4/Figure 6D INPUT-pS473 AKT.jpg]

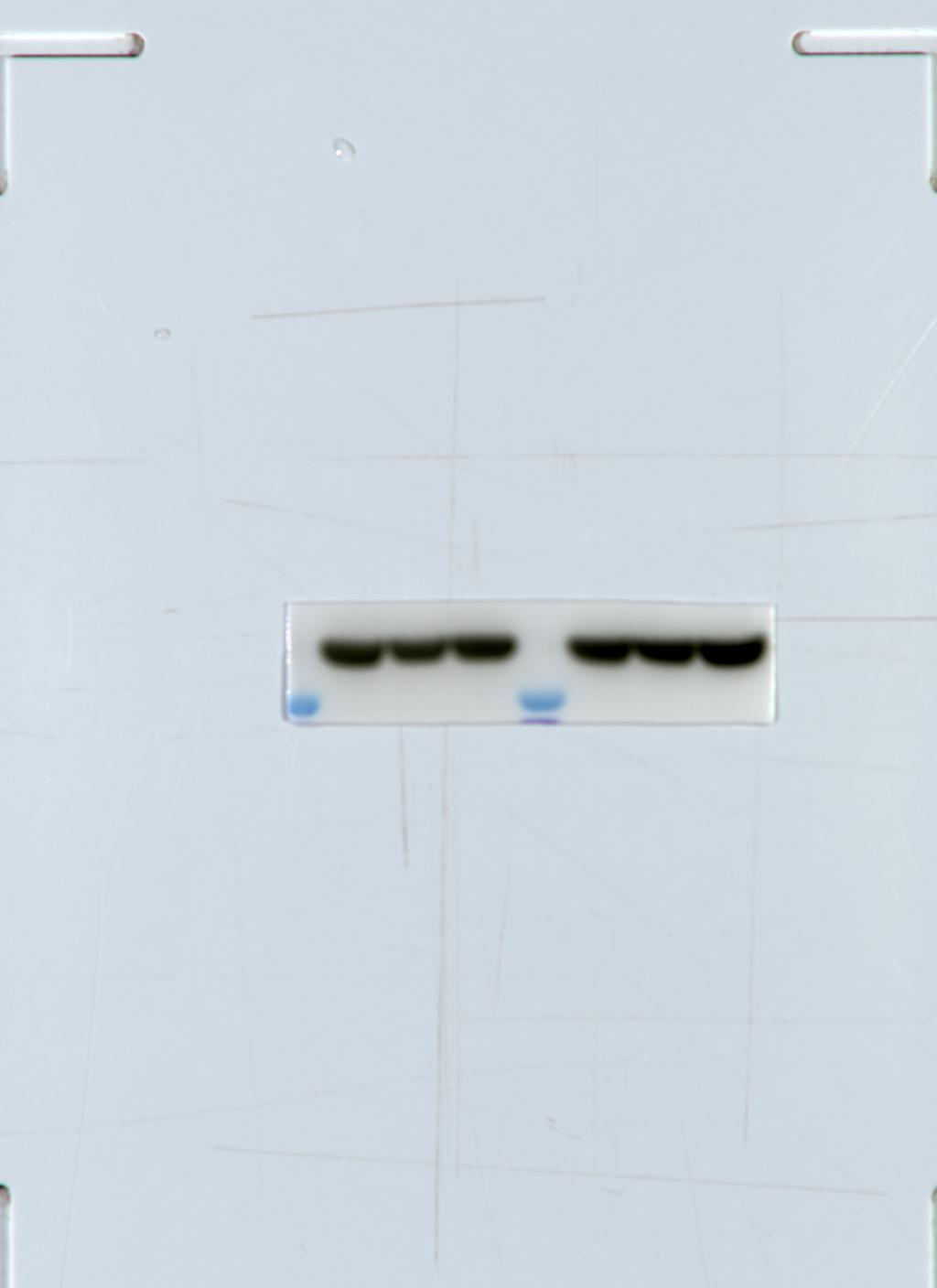

Supplement: Figure 4—source data 5. [file elife-76183-fig4-data5.zip › Figure 4-source data 5/Figure 4I Actin.jpg]

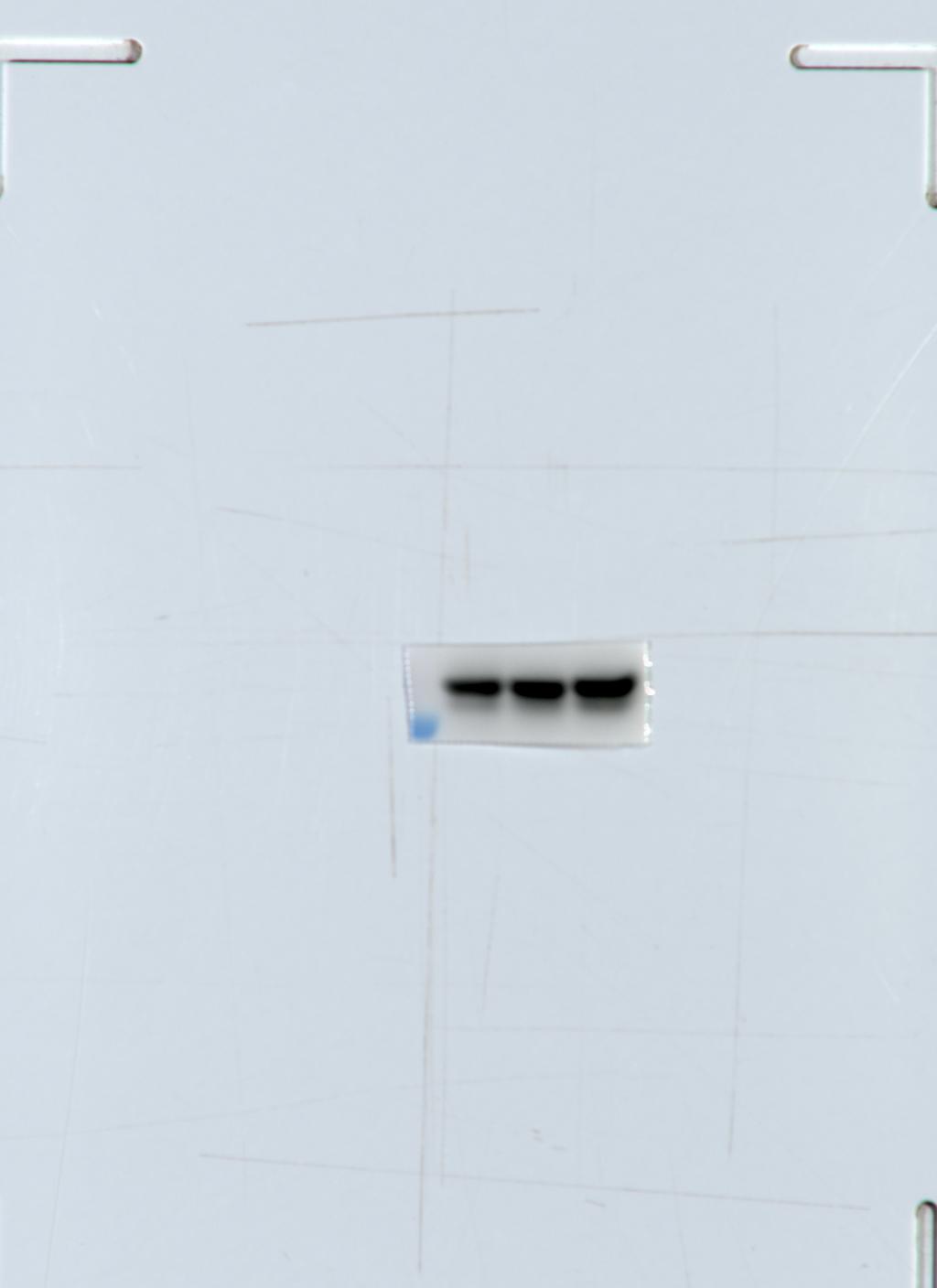

Supplement: Figure 4—source data 5. [file elife-76183-fig4-data5.zip › Figure 4-source data 5/Figure 4I AKT.jpg]

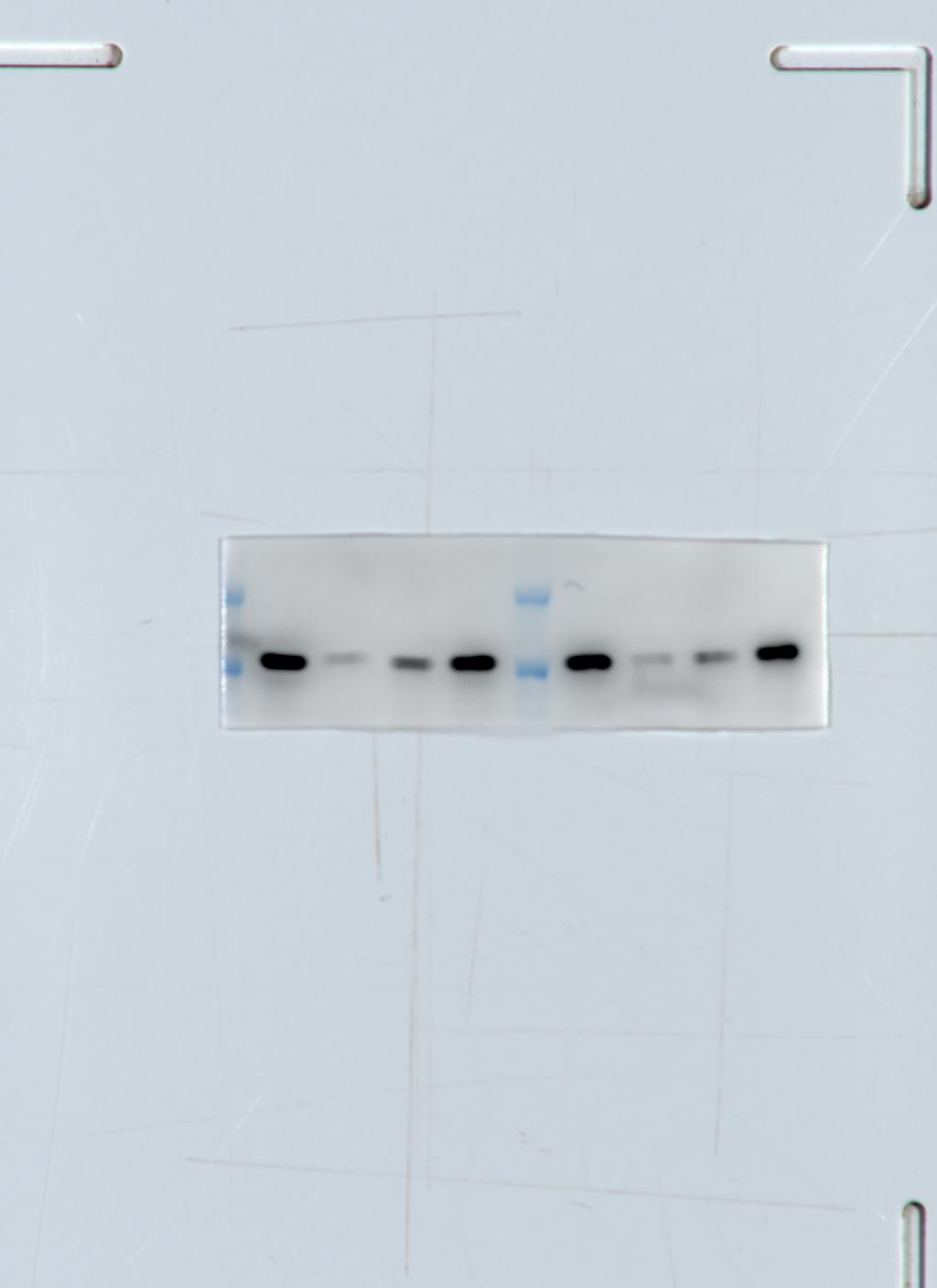

Supplement: Figure 4—source data 5. [file elife-76183-fig4-data5.zip › Figure 4-source data 5/Figure 4I IRS4.jpg]

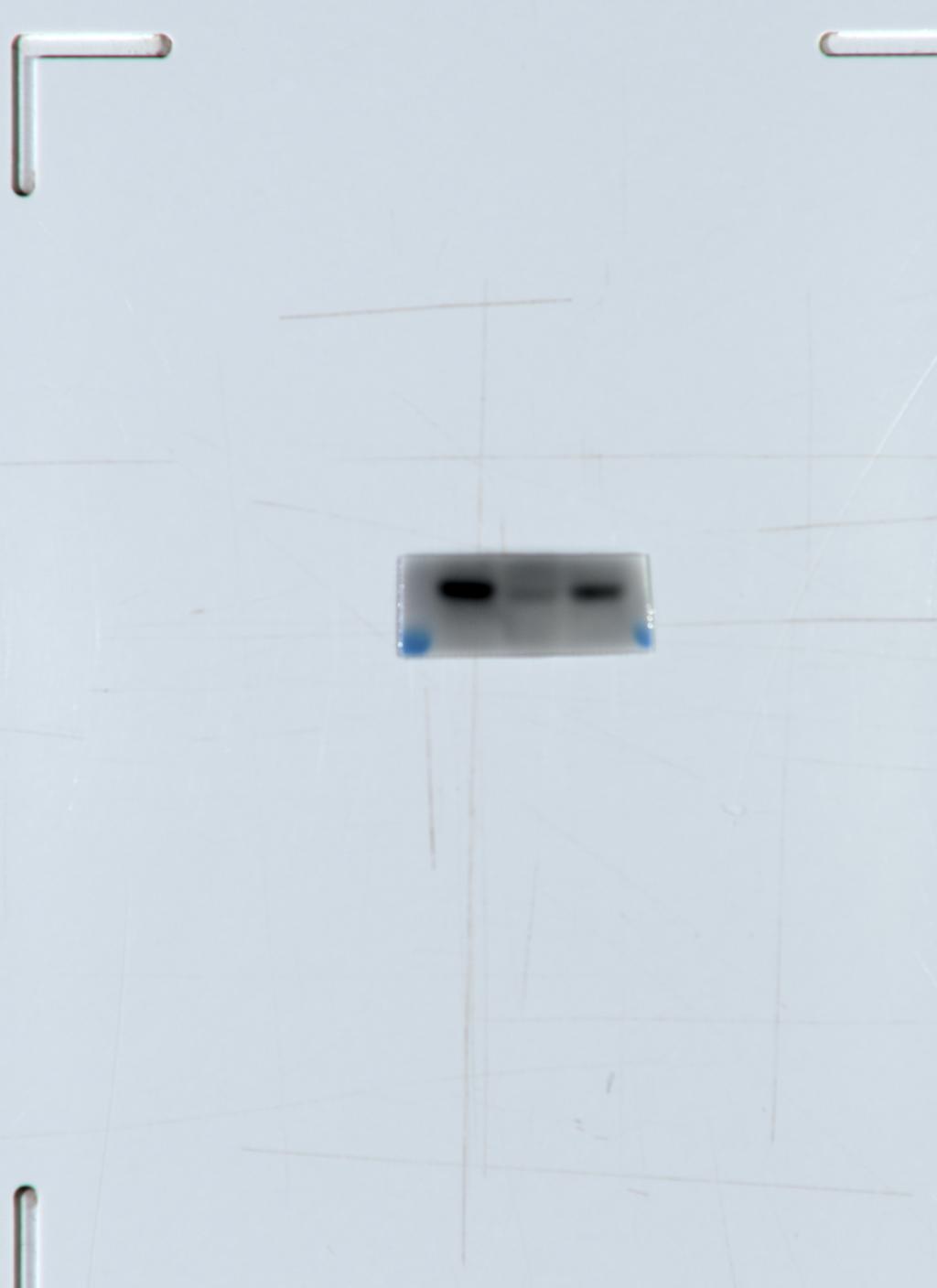

Supplement: Figure 4—source data 5. [file elife-76183-fig4-data5.zip › Figure 4-source data 5/Figure 4I pS473 AKT.jpg]

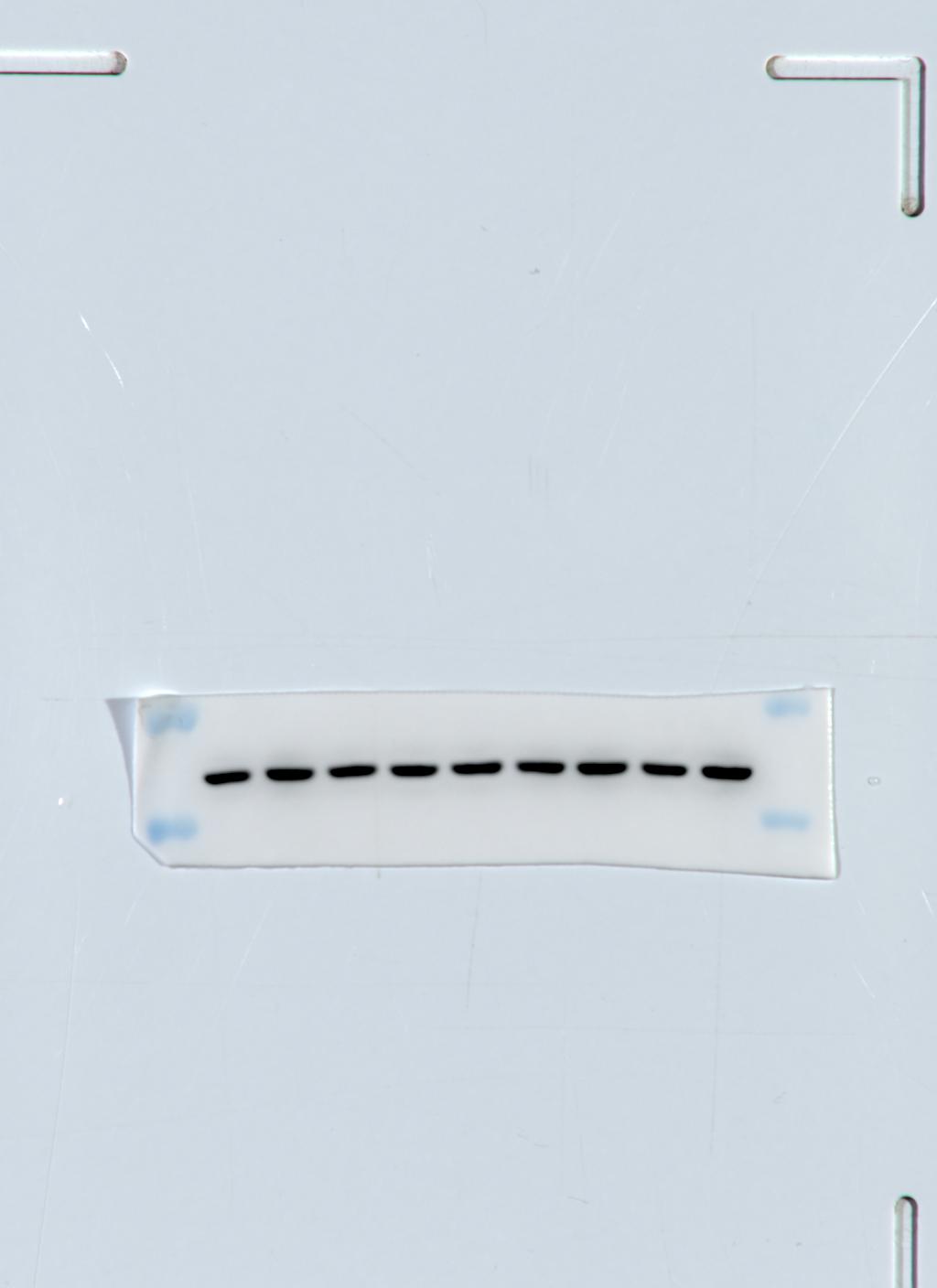

Supplement: Figure 4—source data 6. [file elife-76183-fig4-data6.zip › Figure 4-source data 6/Figure 4K Actin.jpg]

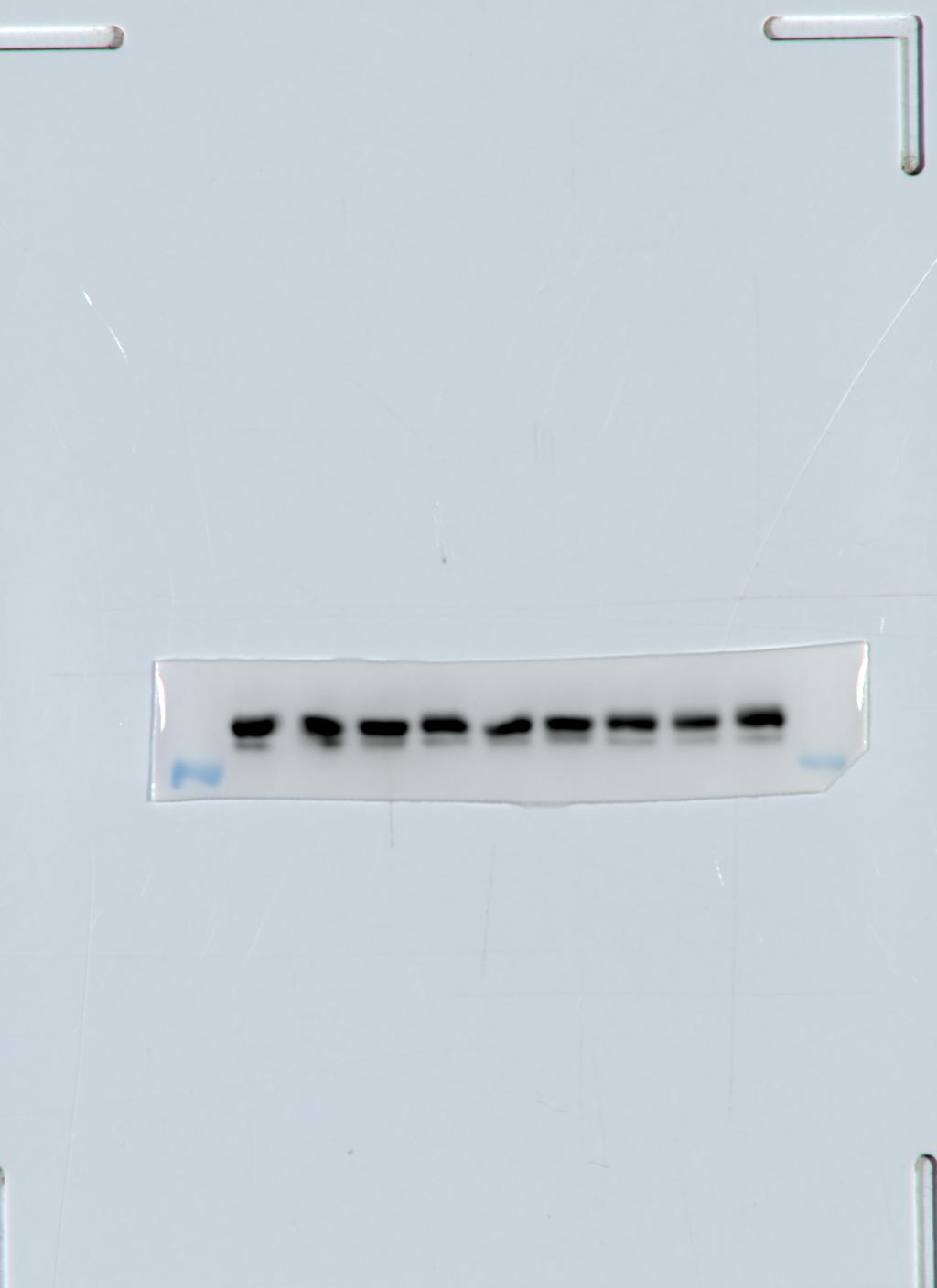

Supplement: Figure 4—source data 6. [file elife-76183-fig4-data6.zip › Figure 4-source data 6/Figure 4K AKT.jpg]

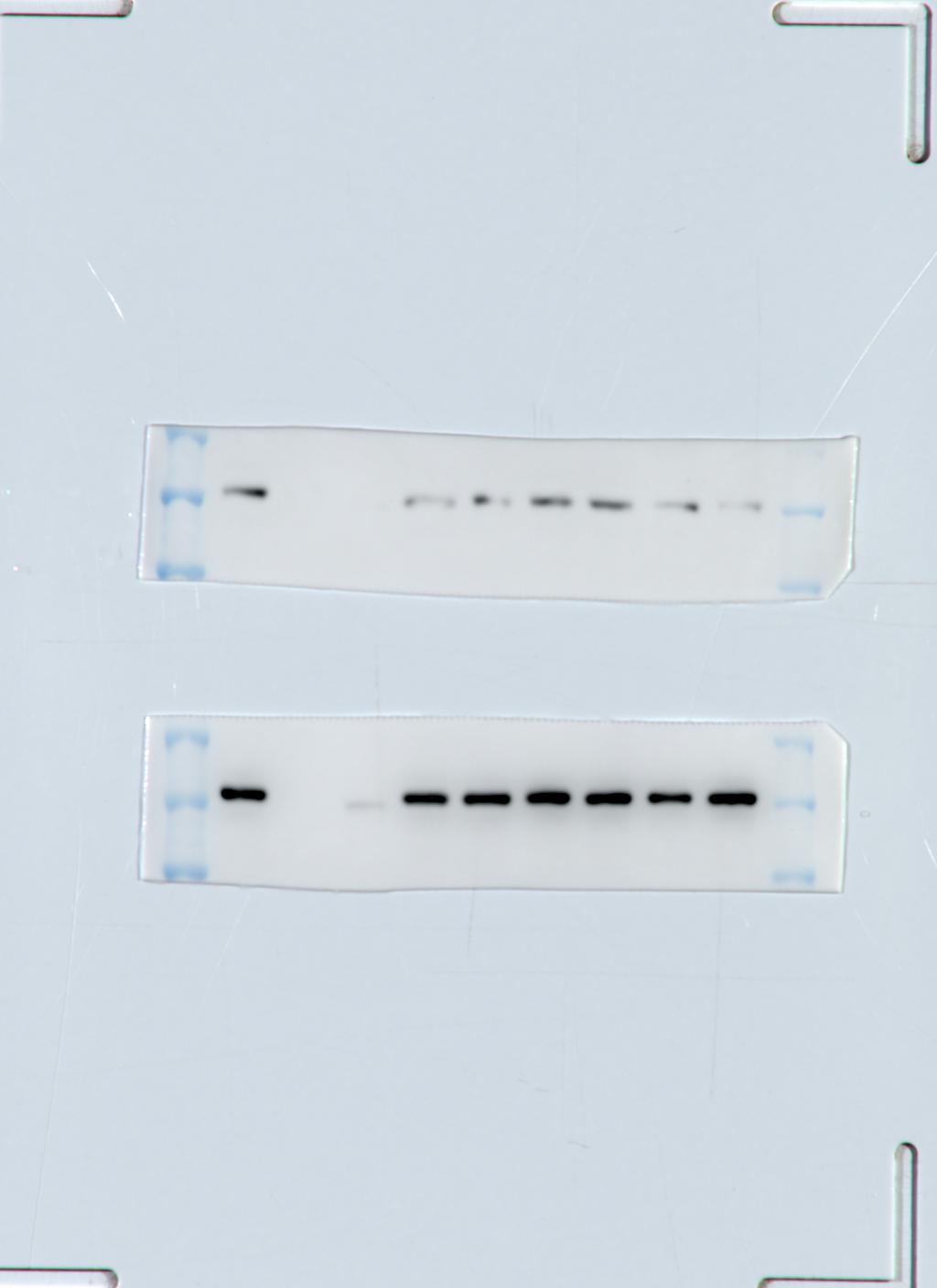

Supplement: Figure 4—source data 6. [file elife-76183-fig4-data6.zip › Figure 4-source data 6/Figure 4K IRS4.jpg]

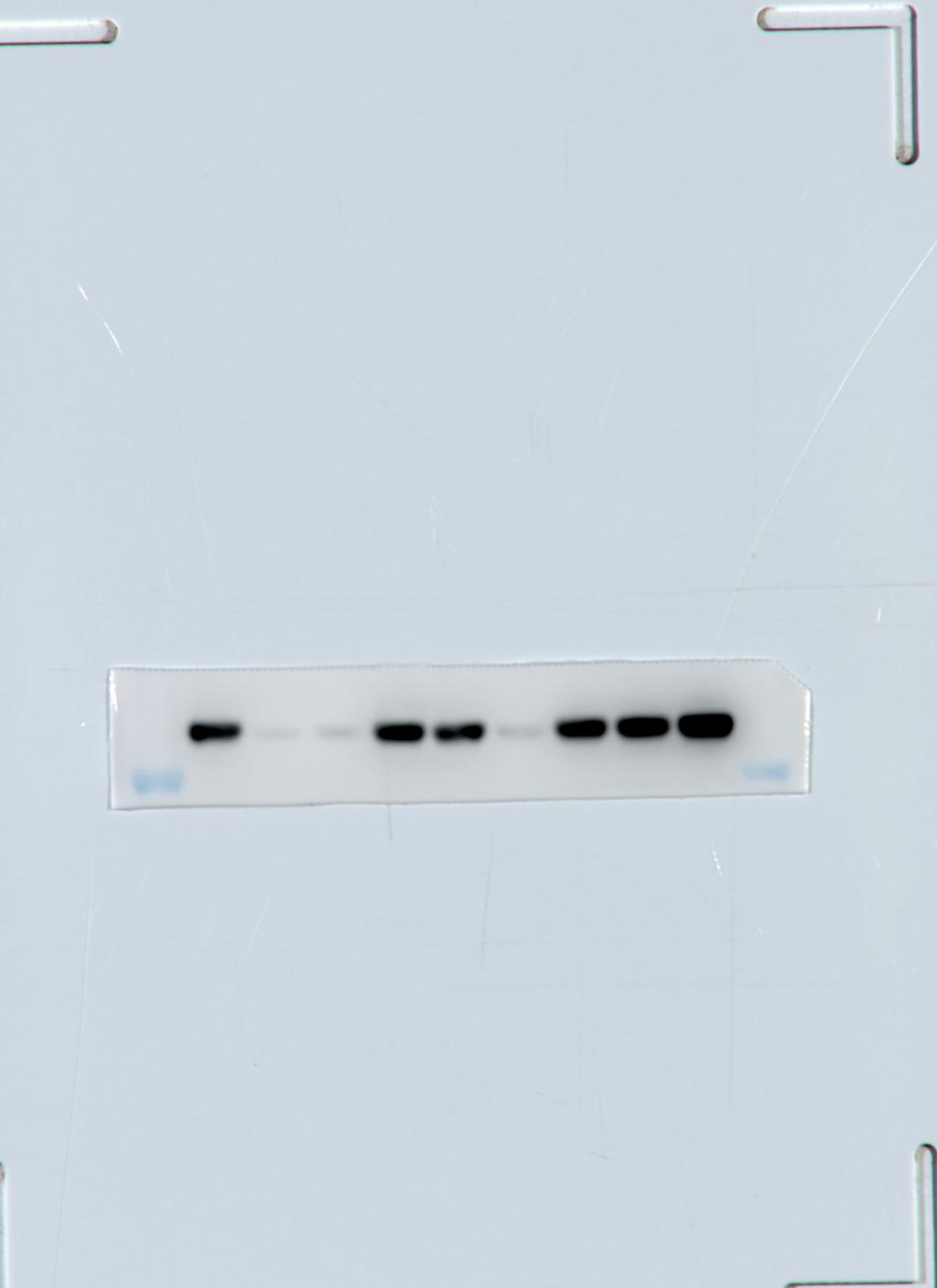

Supplement: Figure 4—source data 6. [file elife-76183-fig4-data6.zip › Figure 4-source data 6/Figure 4K pS473 AKT.jpg]

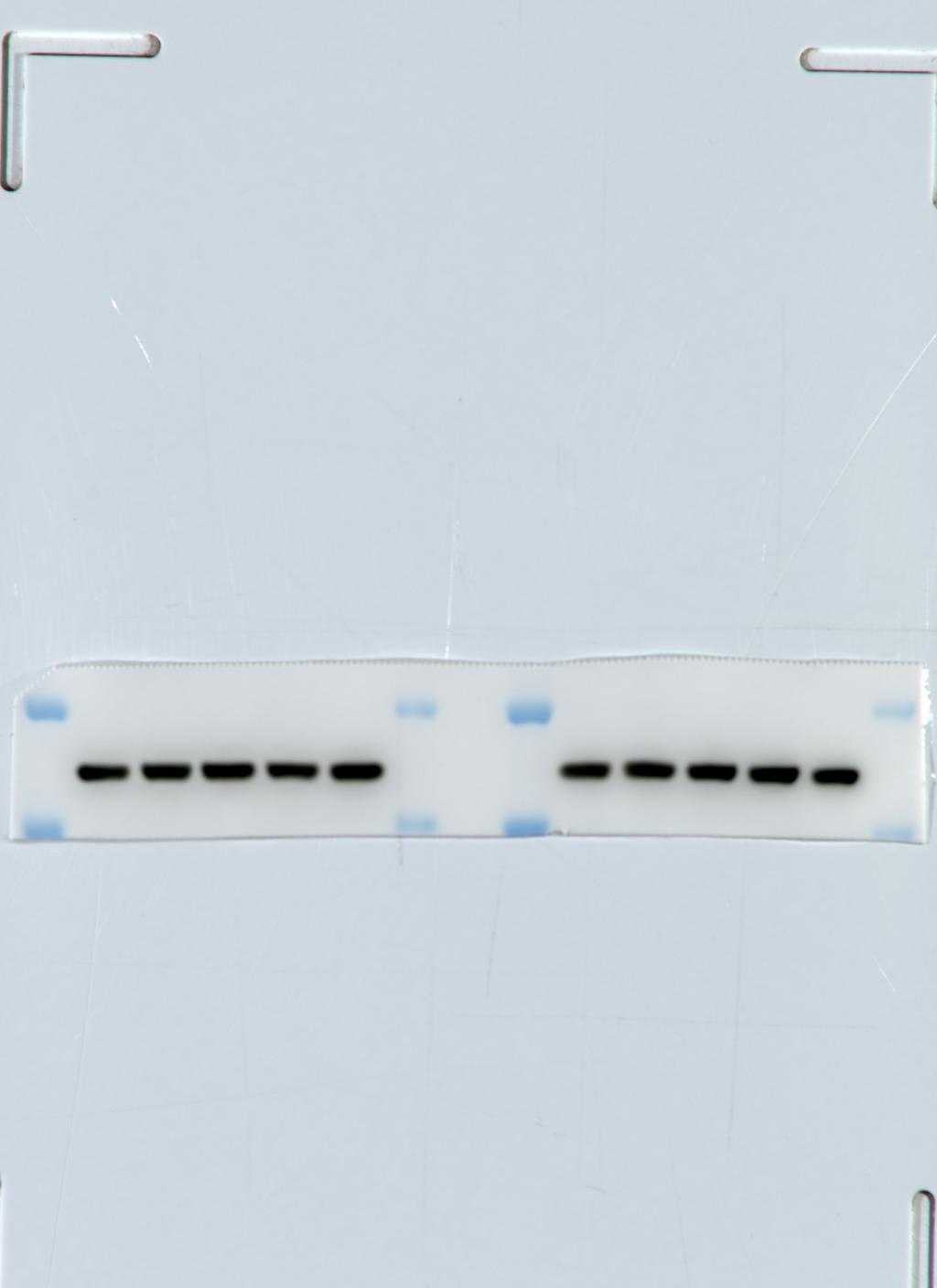

Supplement: Figure 5—source data 1. [file elife-76183-fig5-data1.zip › Figure 5-source data 1/Figfure 5H INPUT-Actin.jpg]

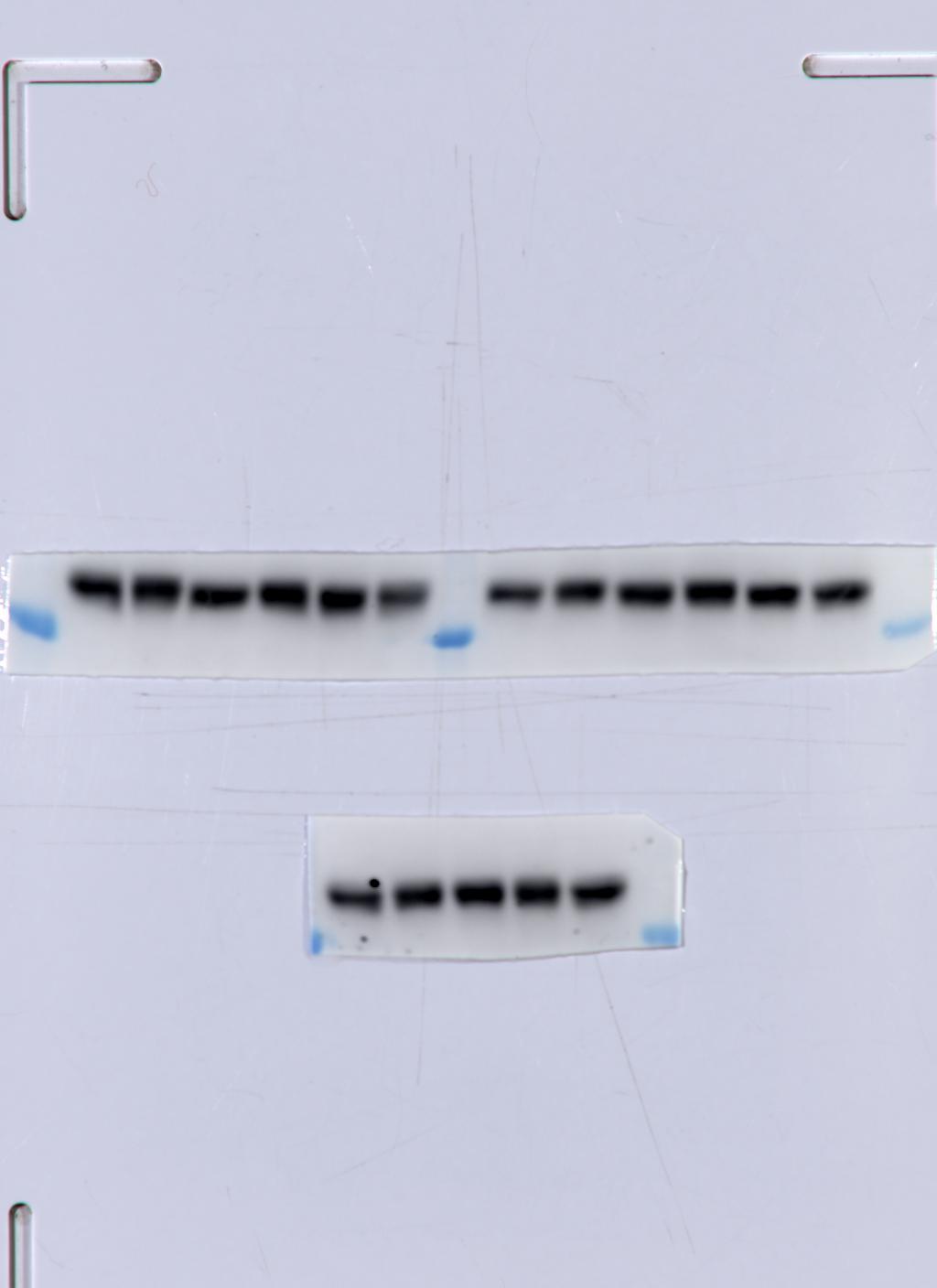

Supplement: Figure 5—source data 1. [file elife-76183-fig5-data1.zip › Figure 5-source data 1/Figfure 5H INPUT-AKT.jpg]

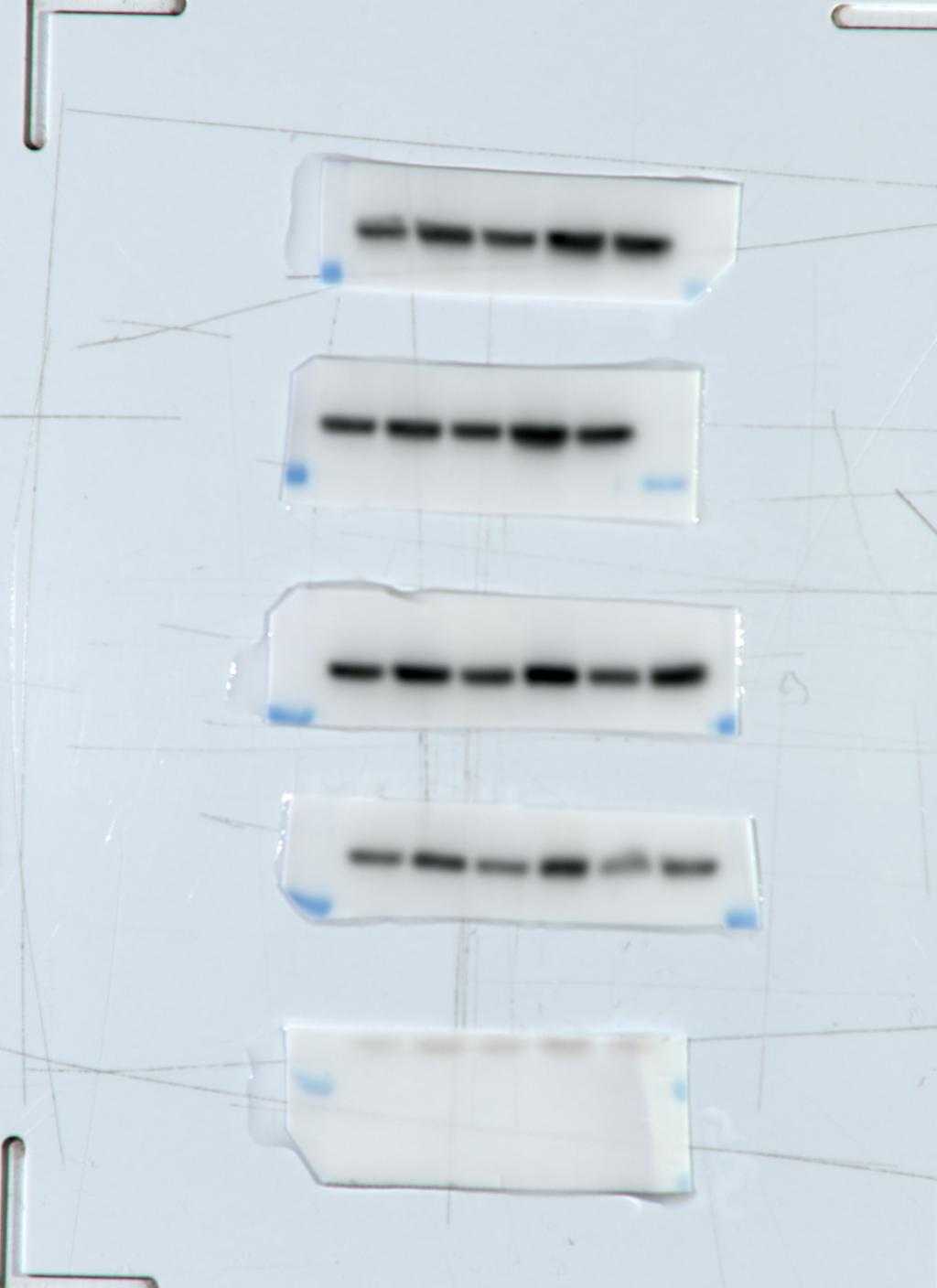

Supplement: Figure 5—source data 1. [file elife-76183-fig5-data1.zip › Figure 5-source data 1/Figfure 5H INPUT-pS473 AKT.jpg]

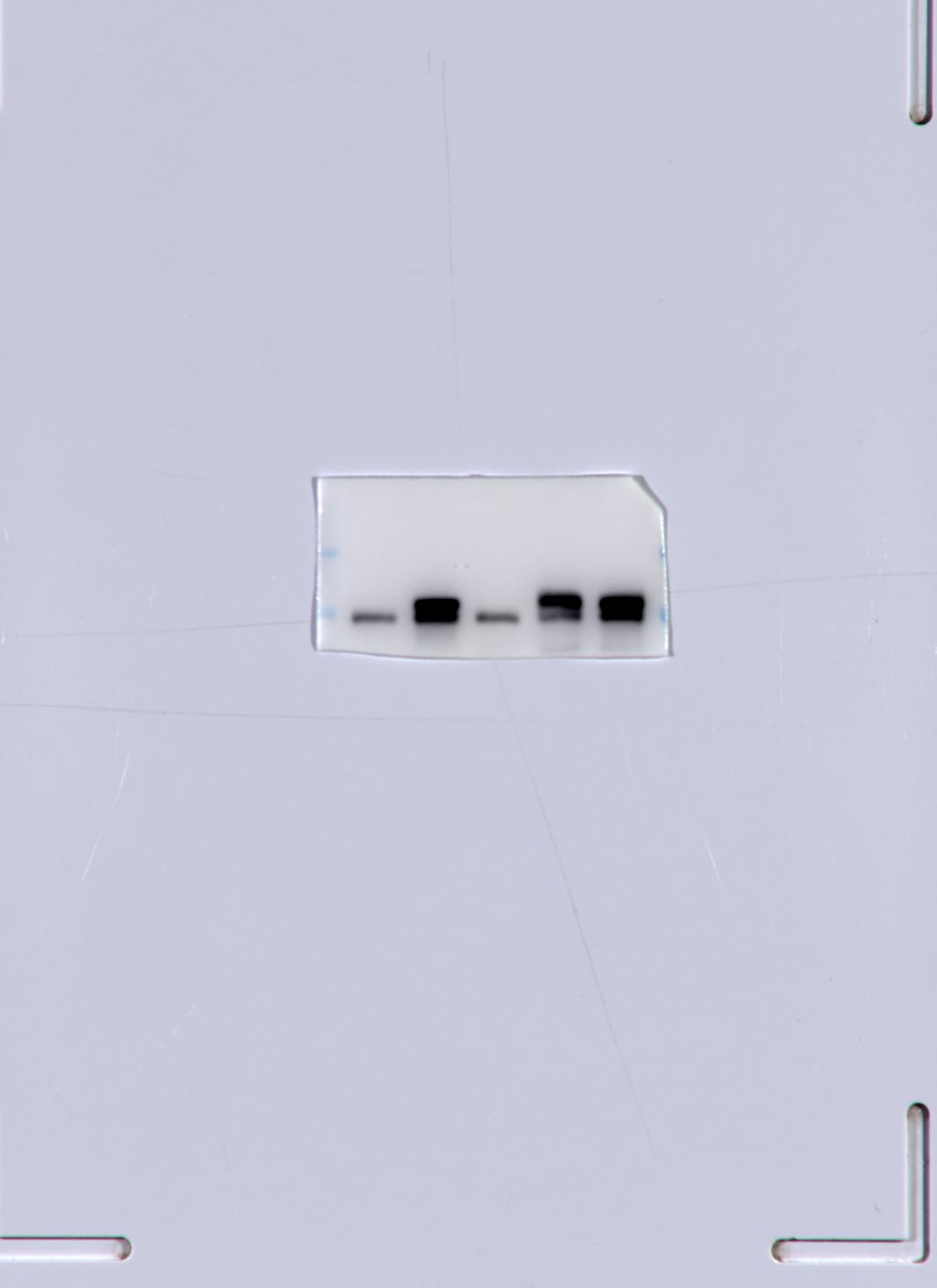

Supplement: Figure 5—source data 1. [file elife-76183-fig5-data1.zip › Figure 5-source data 1/Figure 5H INPUT-IRS4.jpg]

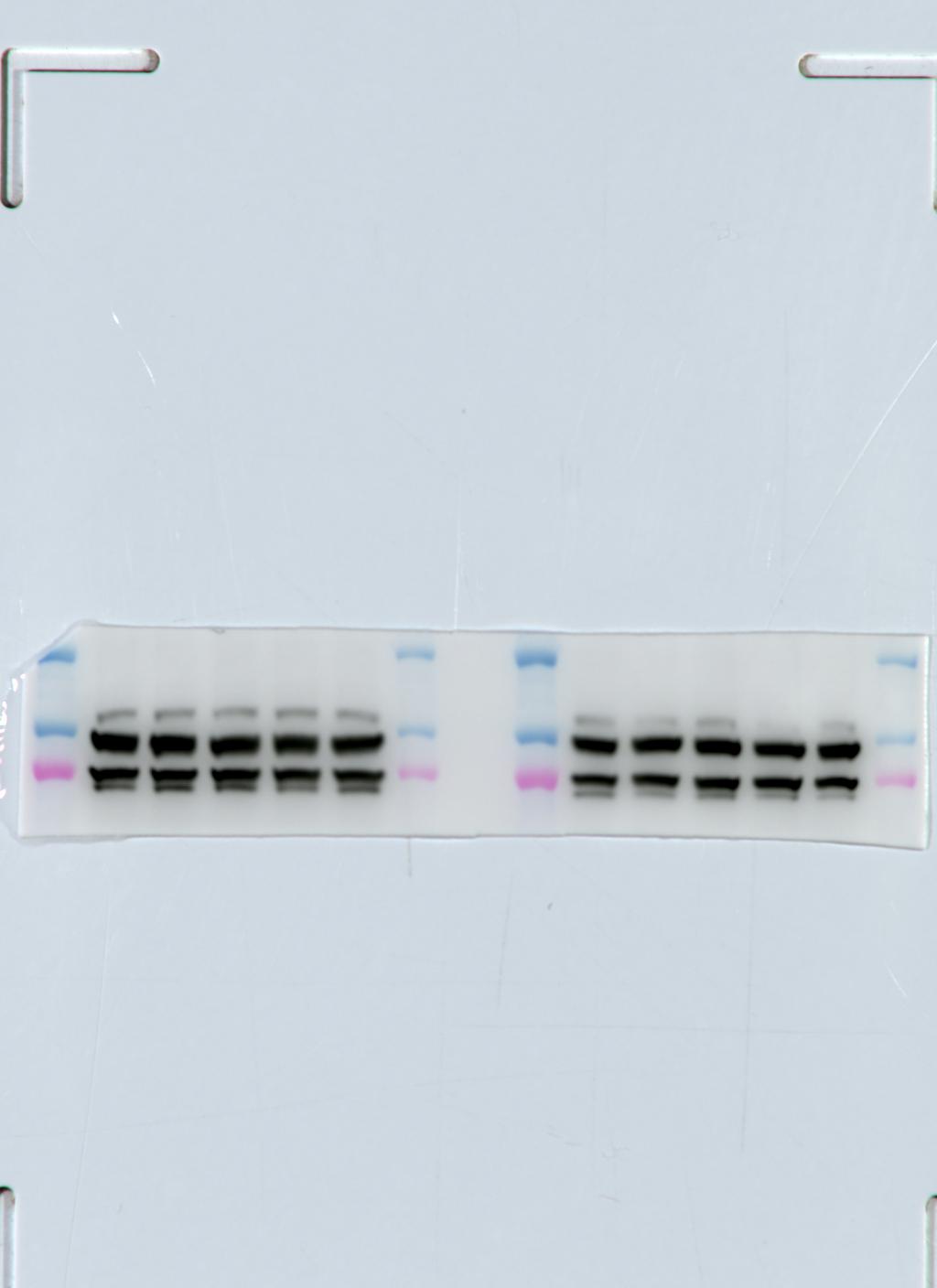

Supplement: Figure 5—source data 1. [file elife-76183-fig5-data1.zip › Figure 5-source data 1/Figure 5H INPUT-PIK3R2.jpg]

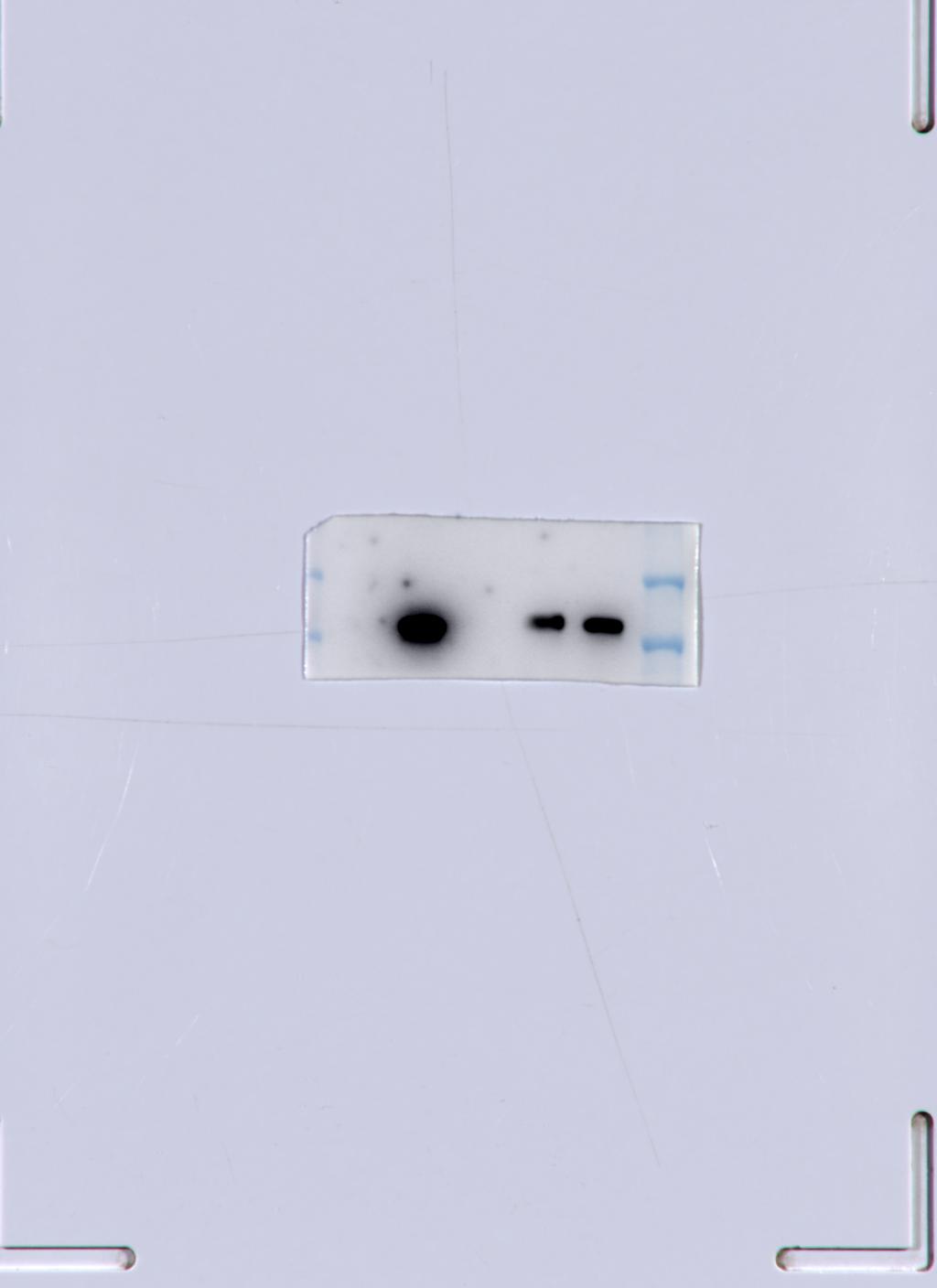

Supplement: Figure 5—source data 1. [file elife-76183-fig5-data1.zip › Figure 5-source data 1/Figure 5H IP-IRS4.jpg]

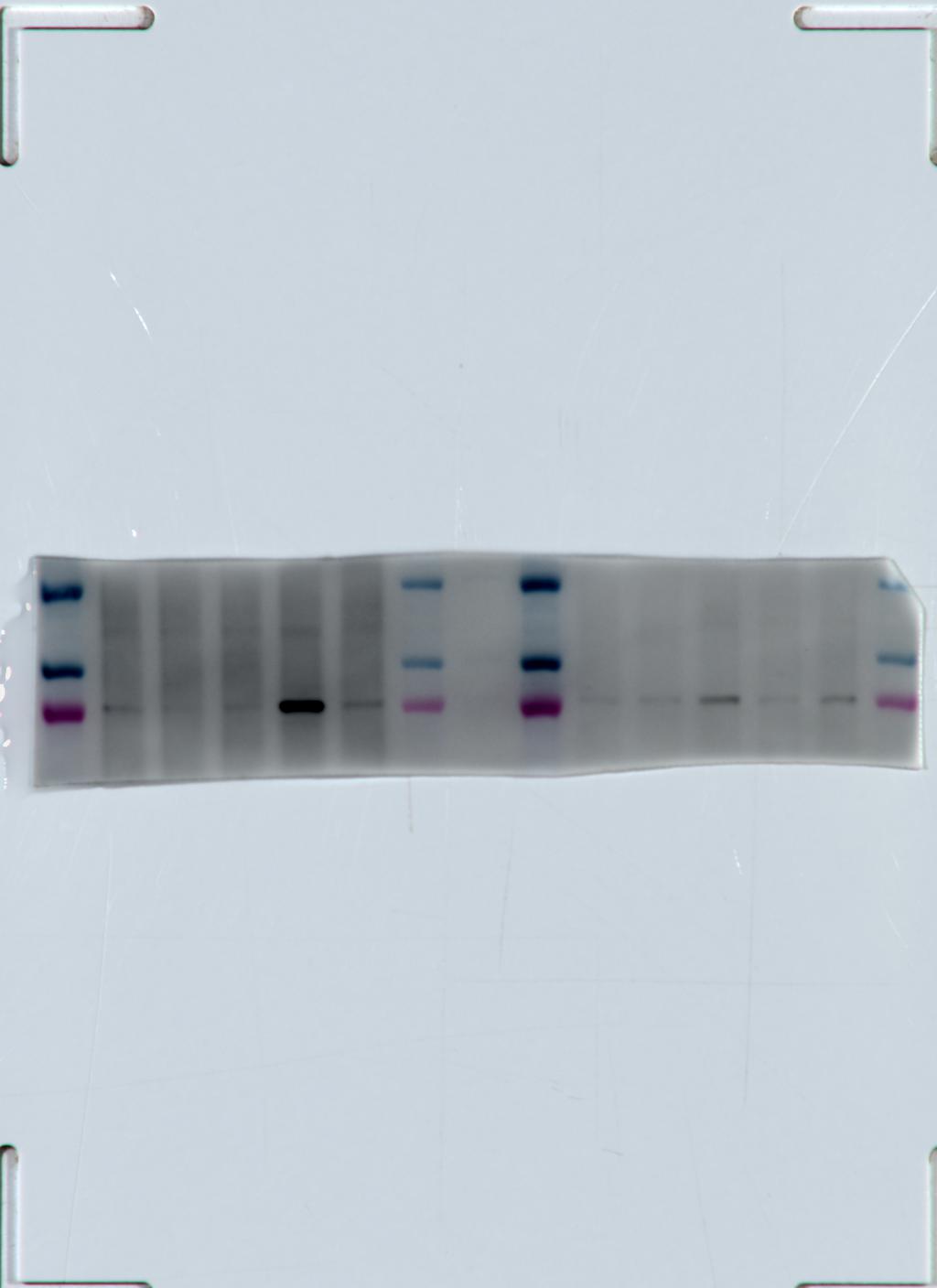

Supplement: Figure 5—source data 1. [file elife-76183-fig5-data1.zip › Figure 5-source data 1/Figure 5H IP-PIK3R2.jpg]

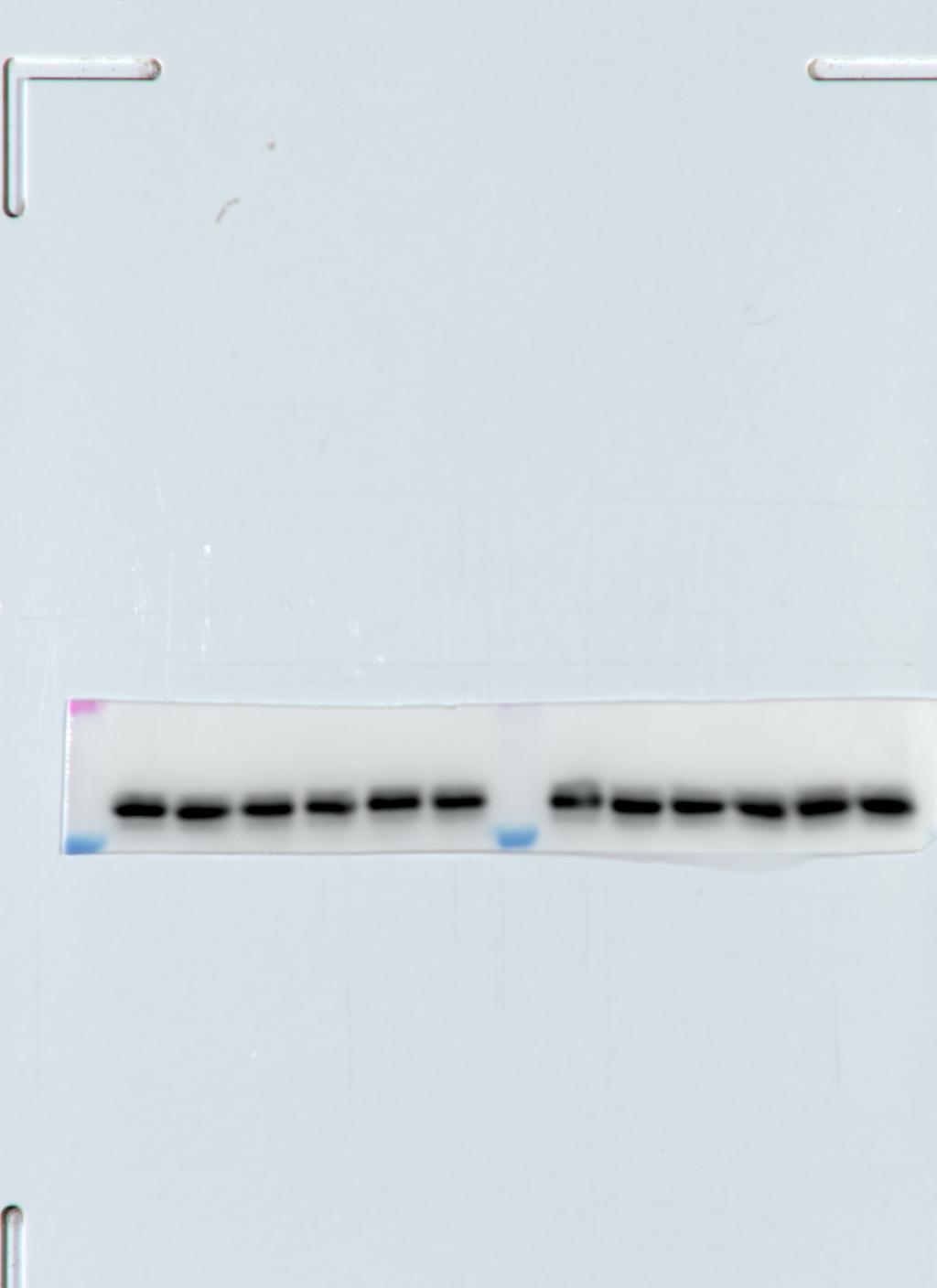

Supplement: Figure 5—figure supplement 1—source data 1. [file elife-76183-fig5-figsupp1-data1.zip › Figure 5-figure supplement 1-source data 1/Figure 5 S1A INPUT-AKT.jpg]

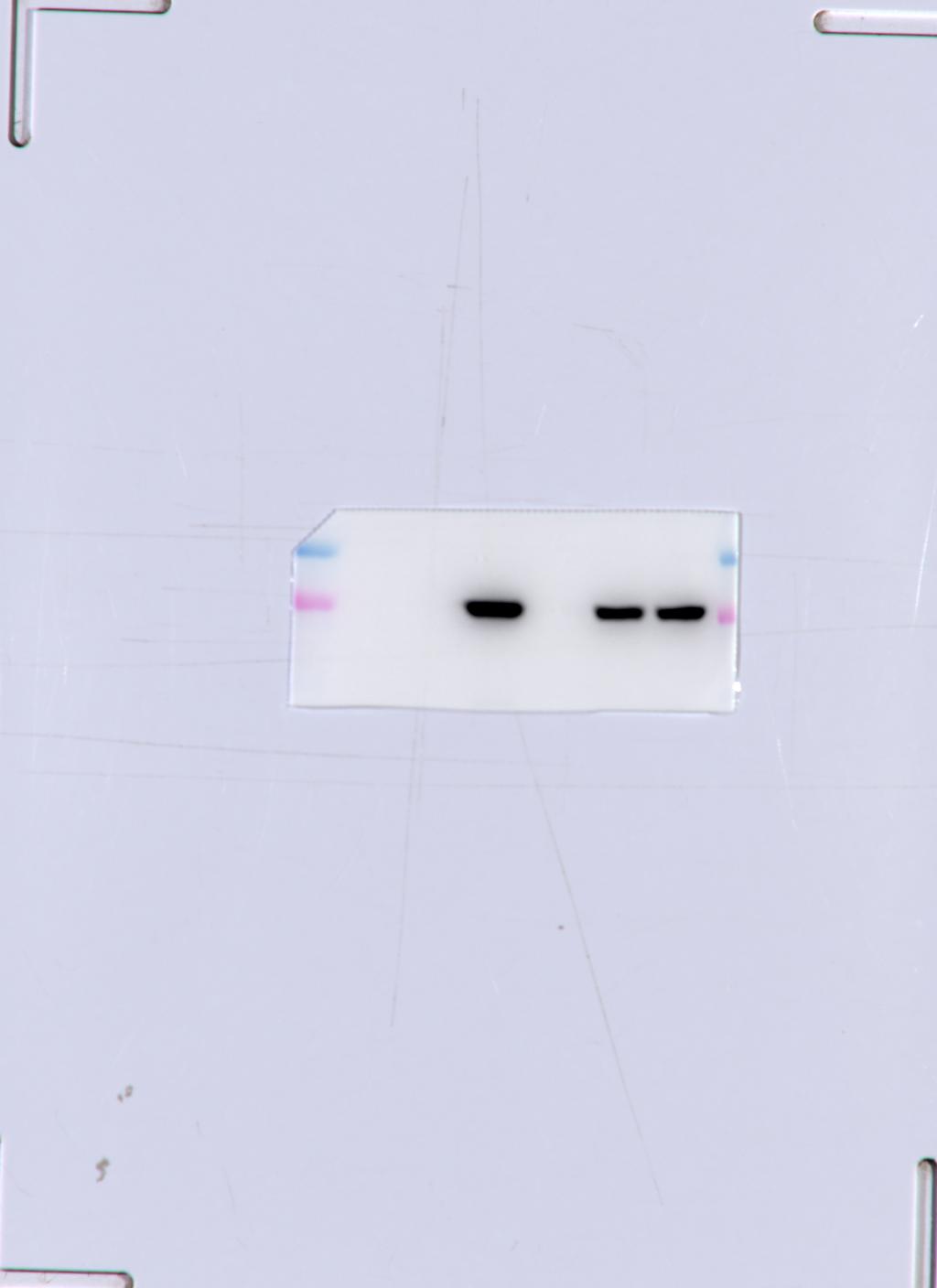

Supplement: Figure 5—figure supplement 1—source data 1. [file elife-76183-fig5-figsupp1-data1.zip › Figure 5-figure supplement 1-source data 1/Figure 5 S1A INPUT-FLAG.jpg]

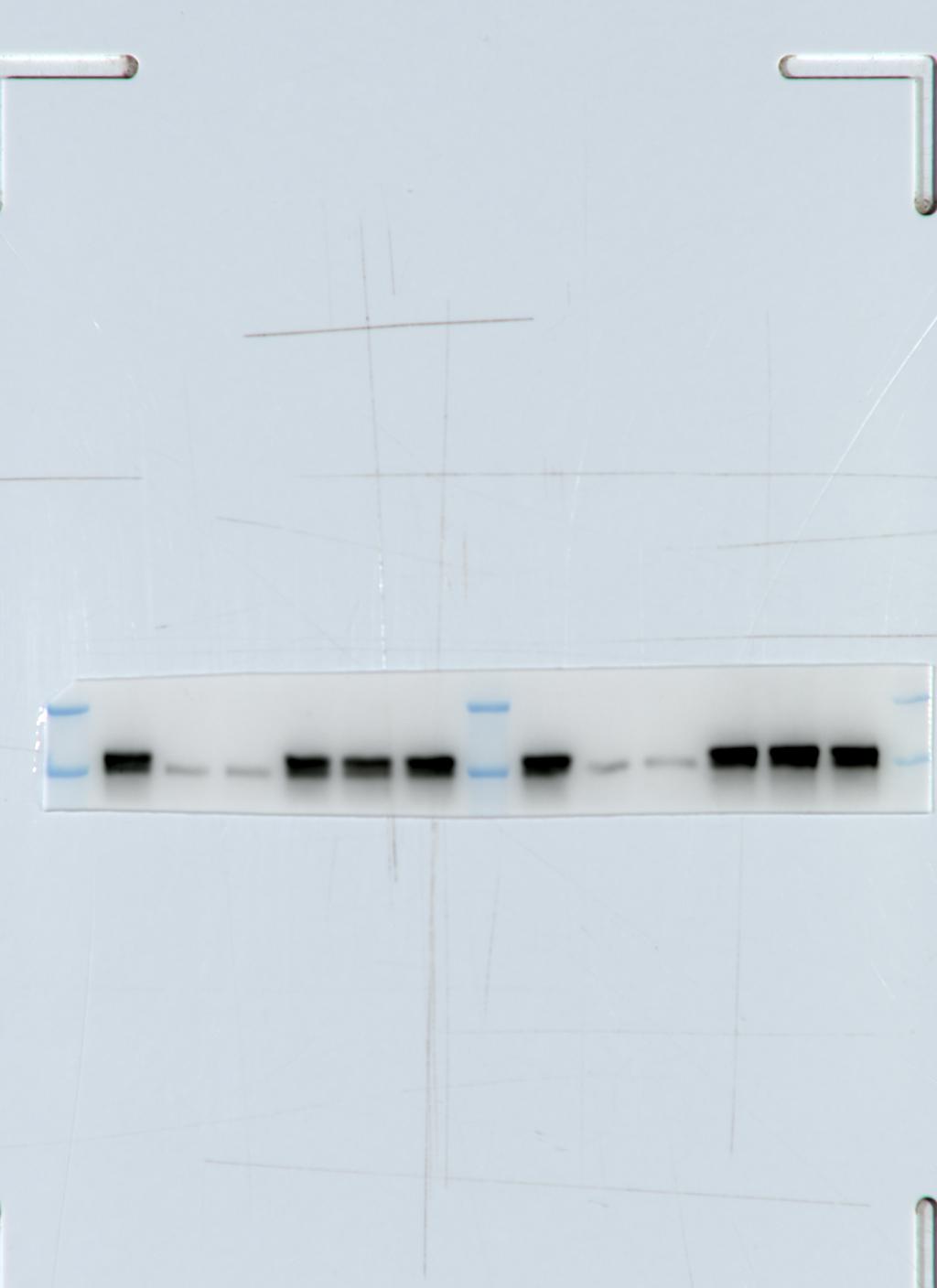

Supplement: Figure 5—figure supplement 1—source data 1. [file elife-76183-fig5-figsupp1-data1.zip › Figure 5-figure supplement 1-source data 1/Figure 5 S1A INPUT-IRS4.jpg]

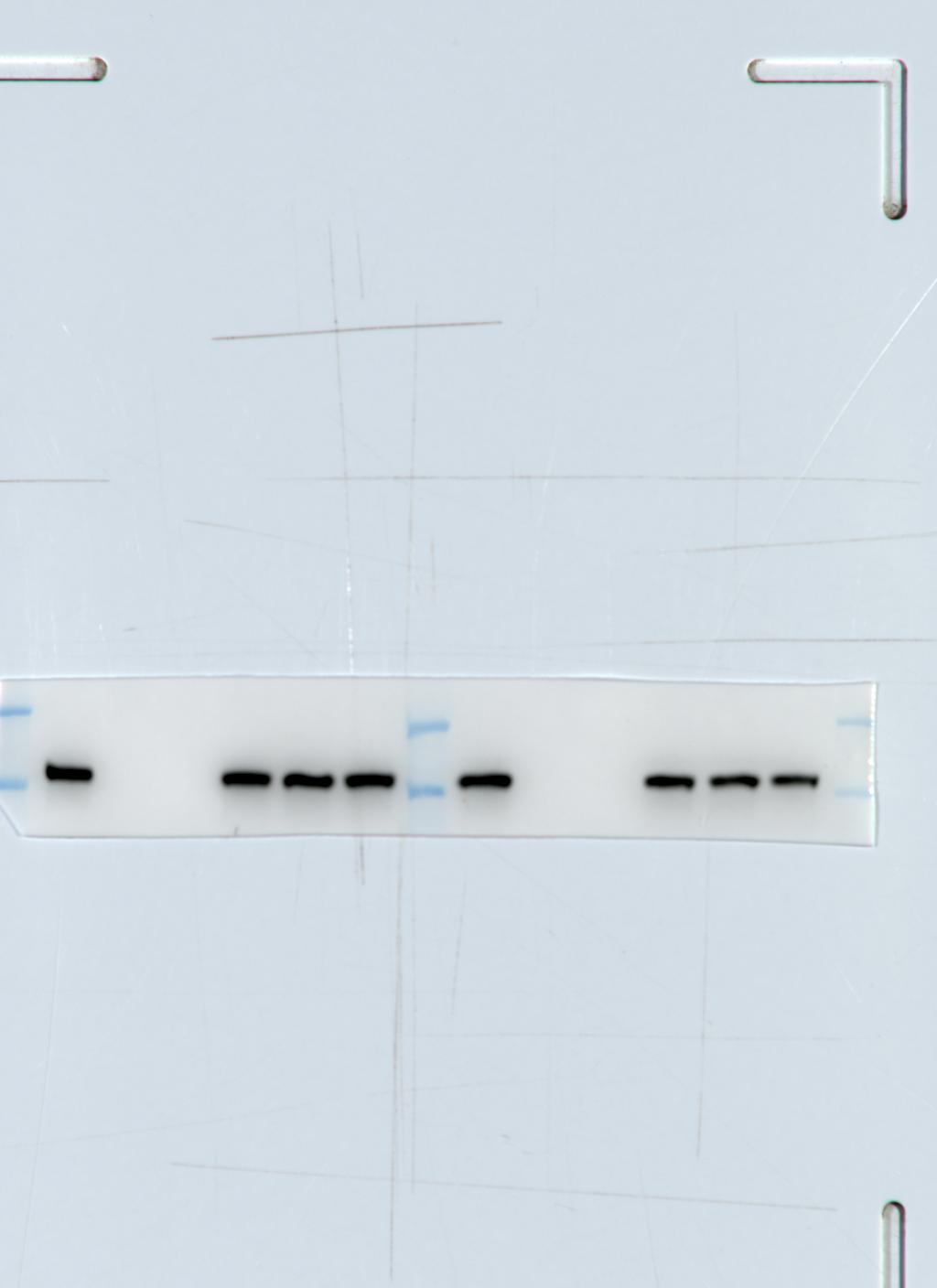

Supplement: Figure 5—figure supplement 1—source data 1. [file elife-76183-fig5-figsupp1-data1.zip › Figure 5-figure supplement 1-source data 1/Figure 5 S1A INPUT-Myc.jpg]

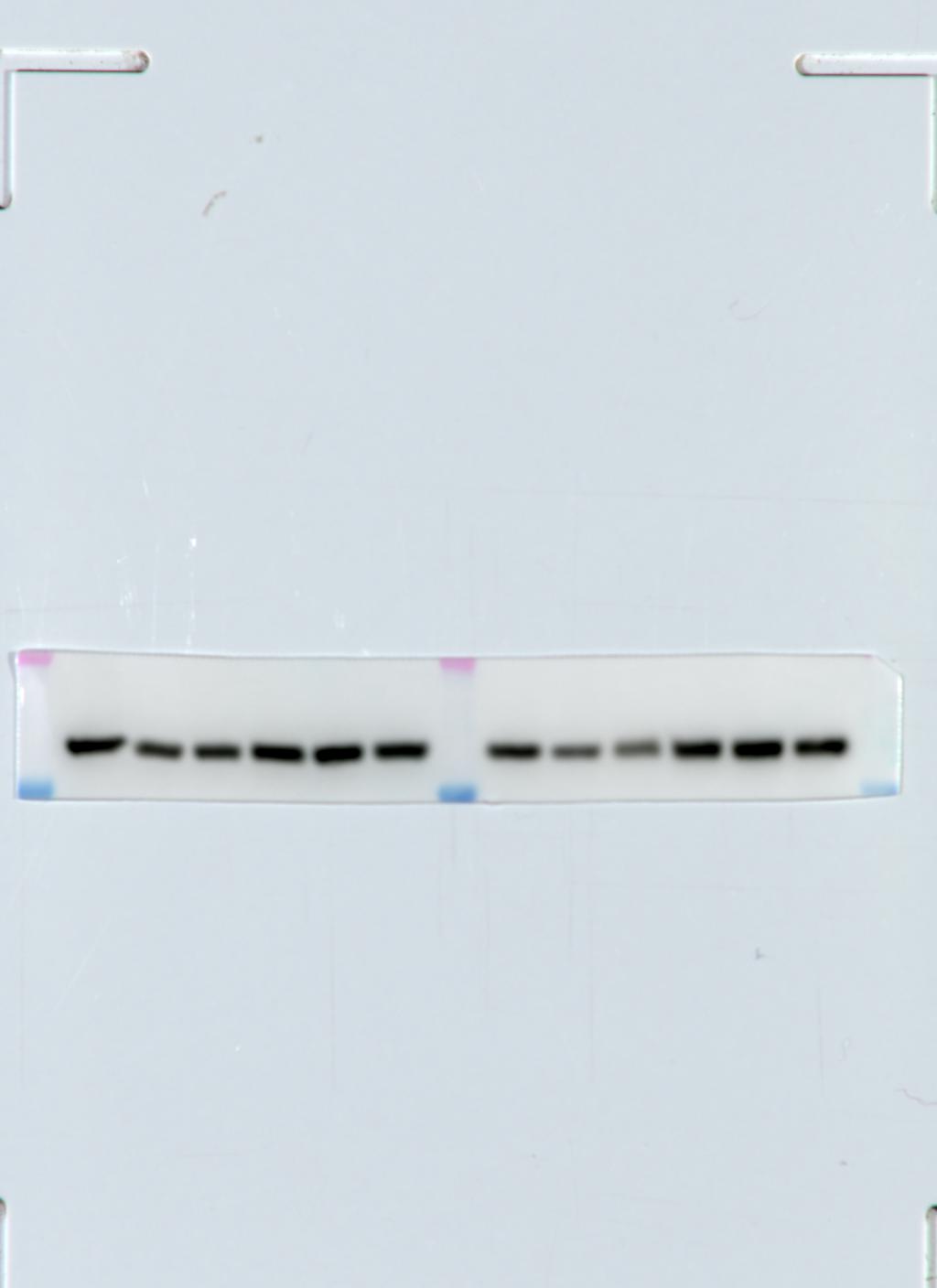

Supplement: Figure 5—figure supplement 1—source data 1. [file elife-76183-fig5-figsupp1-data1.zip › Figure 5-figure supplement 1-source data 1/Figure 5 S1A INPUT-pS473 AKT.jpg]

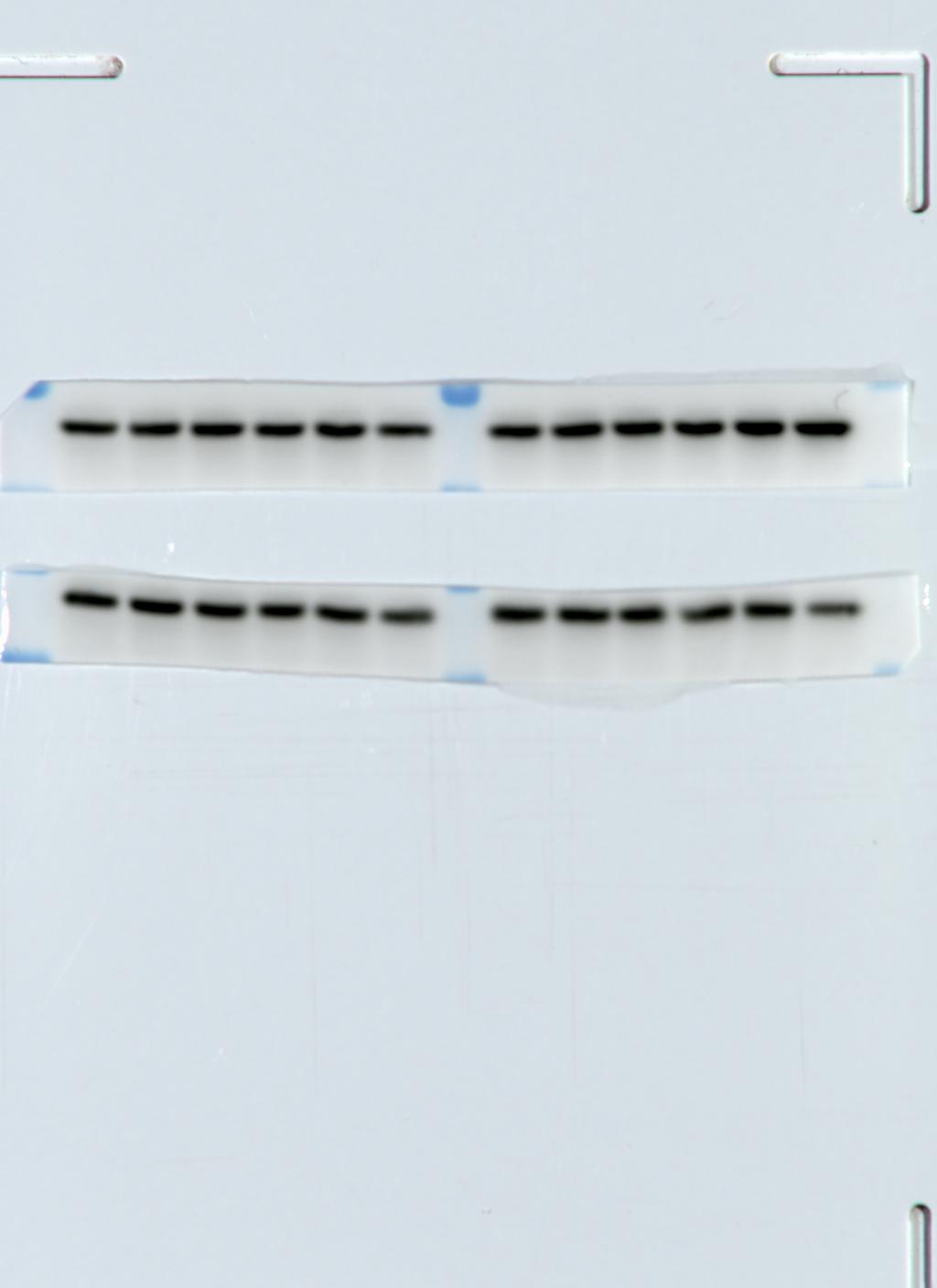

Supplement: Figure 5—figure supplement 1—source data 1. [file elife-76183-fig5-figsupp1-data1.zip › Figure 5-figure supplement 1-source data 1/Figure 5 S1A INPUT-Tubulin.jpg]

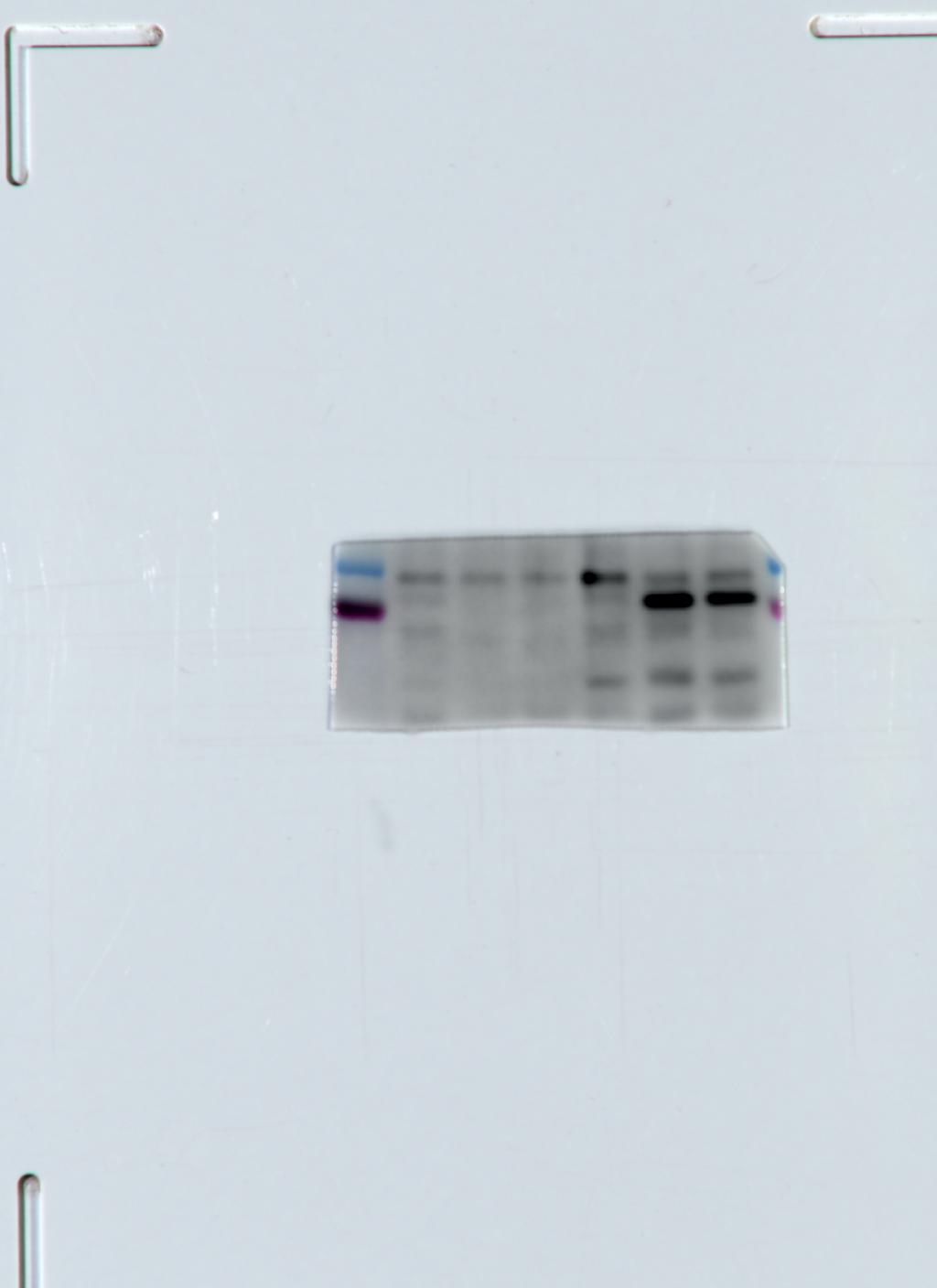

Supplement: Figure 5—figure supplement 1—source data 1. [file elife-76183-fig5-figsupp1-data1.zip › Figure 5-figure supplement 1-source data 1/Figure 5 S1A IP-FLAG.jpg]

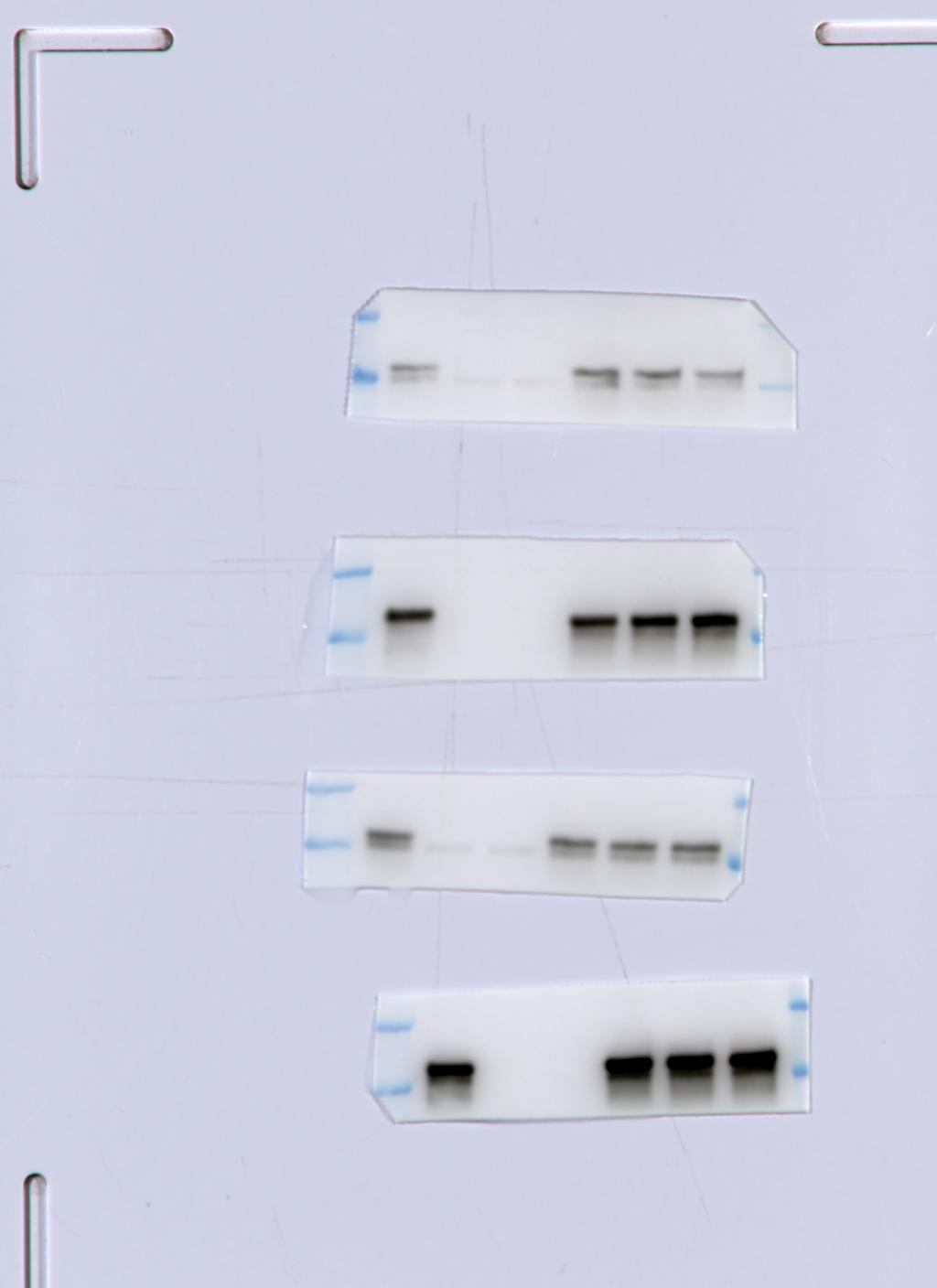

Supplement: Figure 5—figure supplement 1—source data 1. [file elife-76183-fig5-figsupp1-data1.zip › Figure 5-figure supplement 1-source data 1/Figure 5 S1A IP-IRS4.jpg]

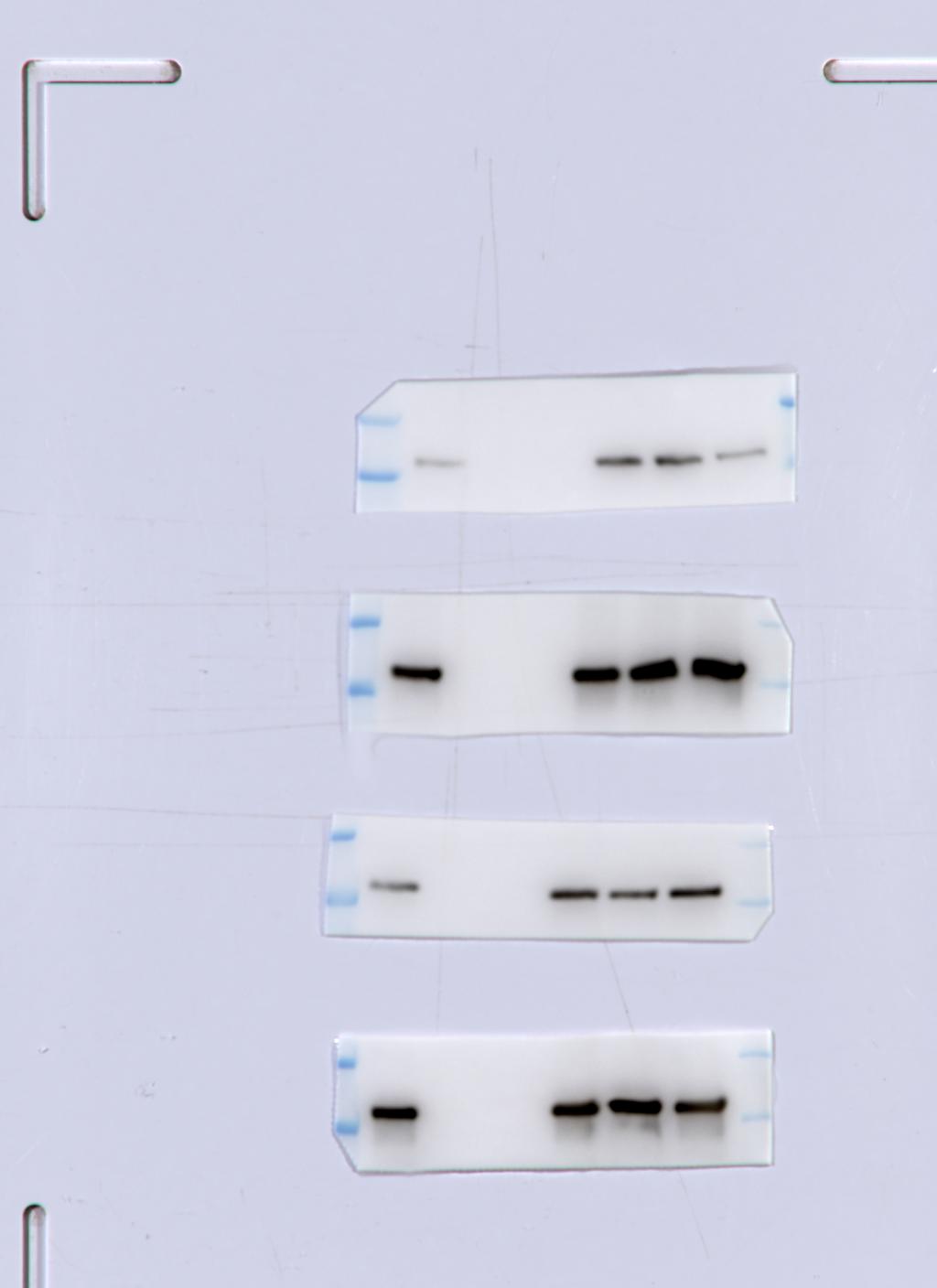

Supplement: Figure 5—figure supplement 1—source data 1. [file elife-76183-fig5-figsupp1-data1.zip › Figure 5-figure supplement 1-source data 1/Figure 5 S1A IP-Myc.jpg]

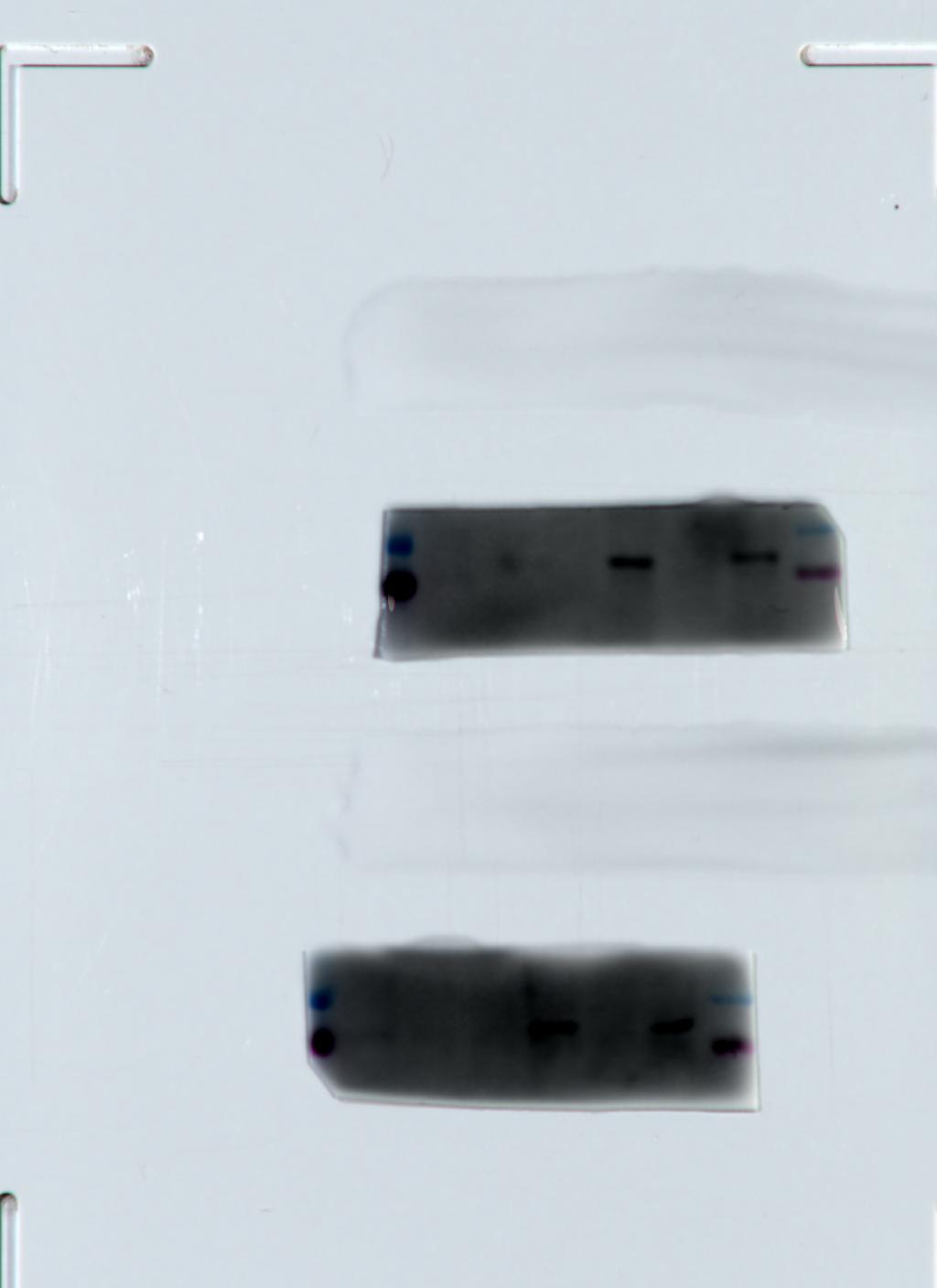

Supplement: Figure 5—figure supplement 1—source data 1. [file elife-76183-fig5-figsupp1-data1.zip › Figure 5-figure supplement 1-source data 1/Figure 5 S1A IP-PIK3R2.jpg]

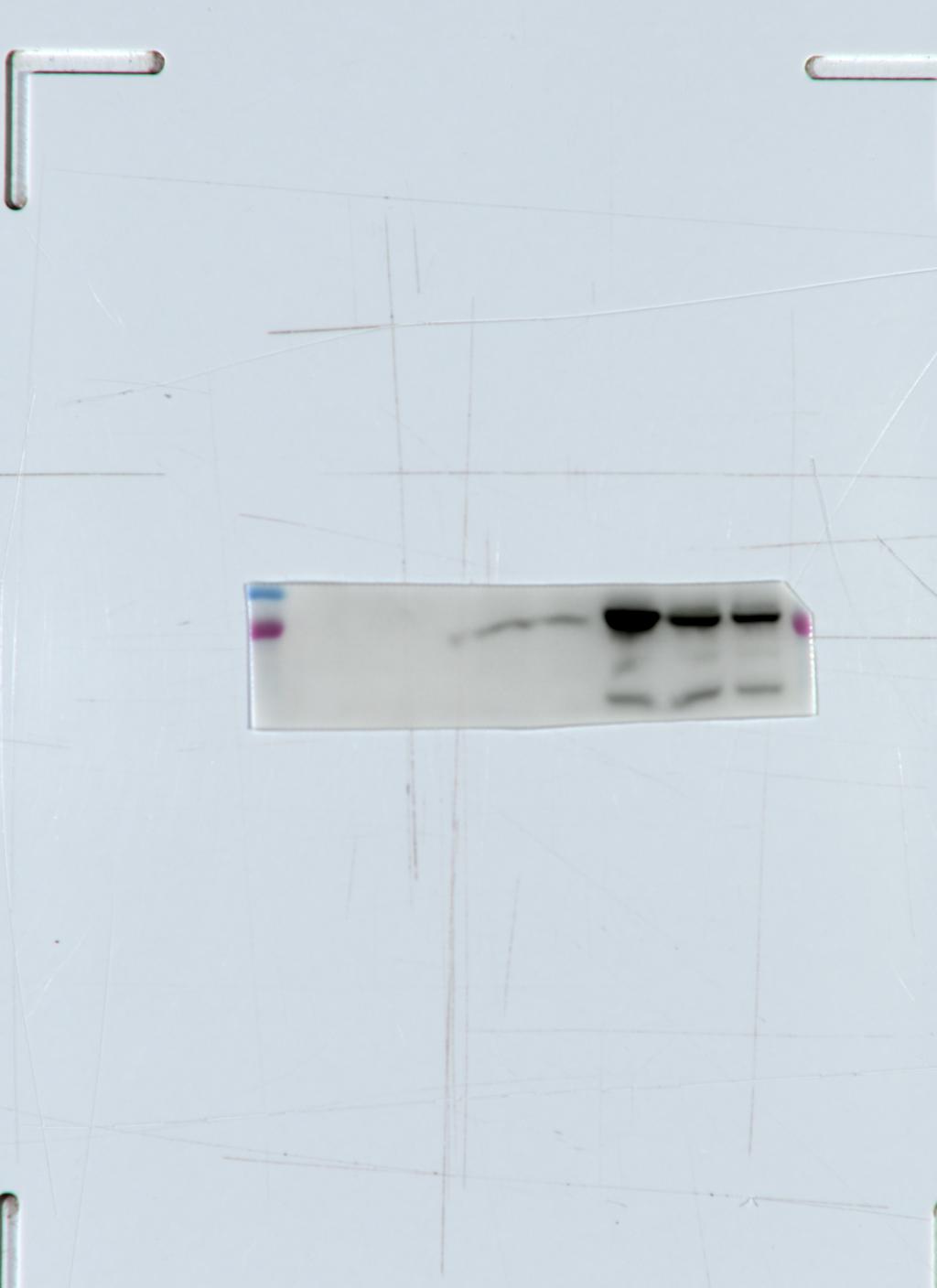

Supplement: Figure 5—figure supplement 1—source data 2. [file elife-76183-fig5-figsupp1-data2.zip › Figure 5-figure supplement 1-source data 2/Figure 5 S1B INPUT-FLAG.jpg]

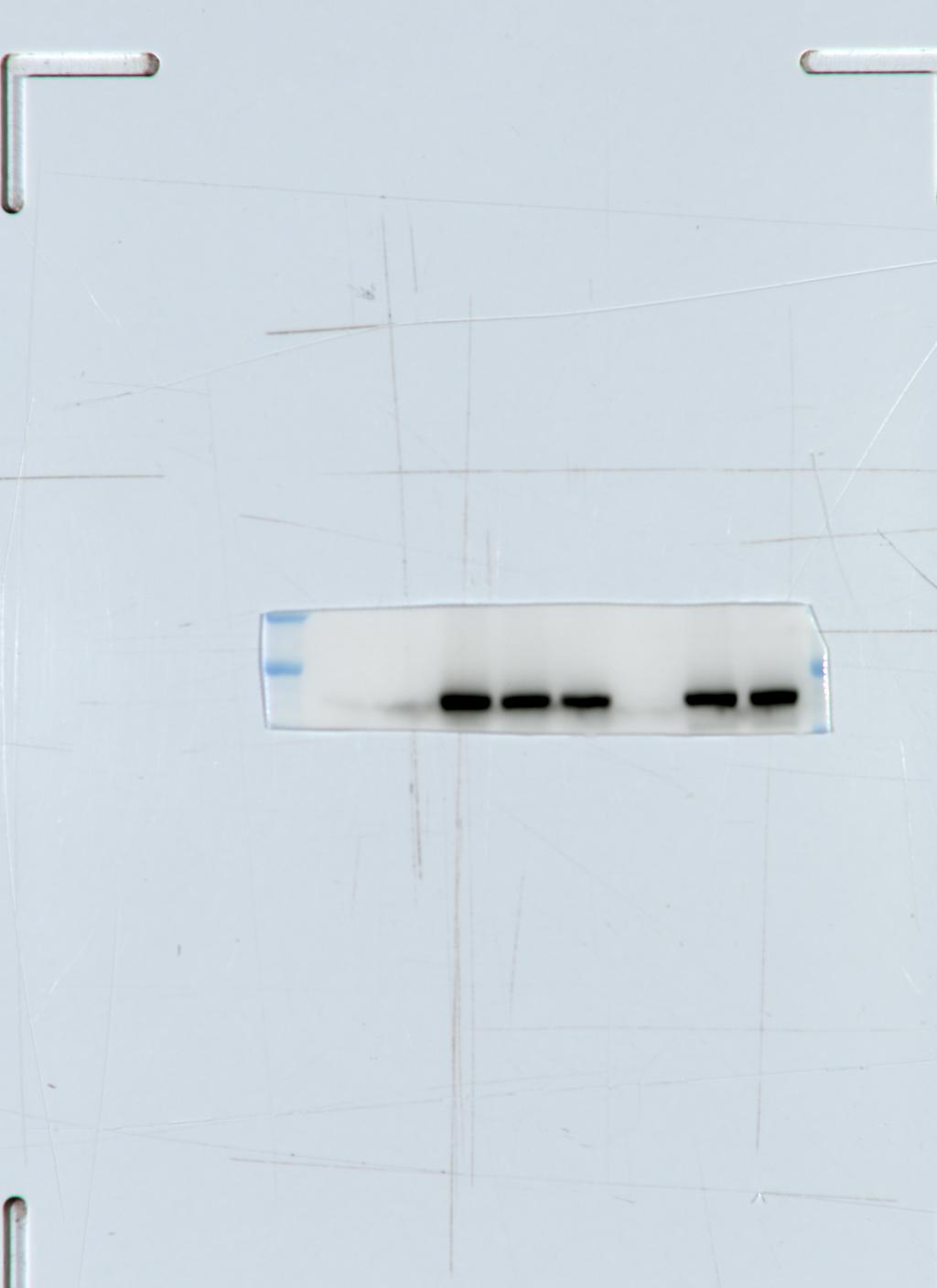

Supplement: Figure 5—figure supplement 1—source data 2. [file elife-76183-fig5-figsupp1-data2.zip › Figure 5-figure supplement 1-source data 2/Figure 5 S1B INPUT-GFP.jpg]

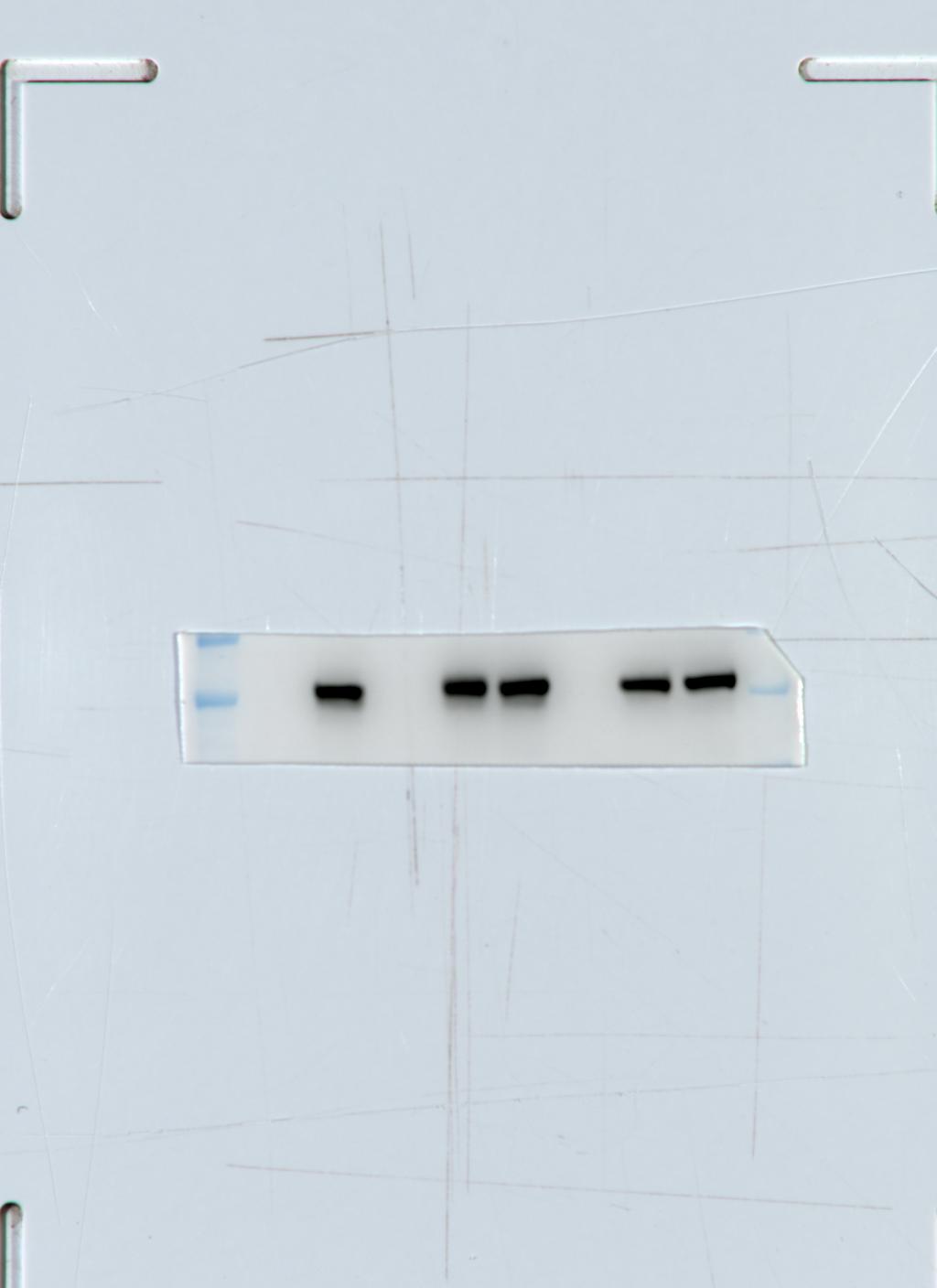

Supplement: Figure 5—figure supplement 1—source data 2. [file elife-76183-fig5-figsupp1-data2.zip › Figure 5-figure supplement 1-source data 2/Figure 5 S1B INPUT-Myc.jpg]

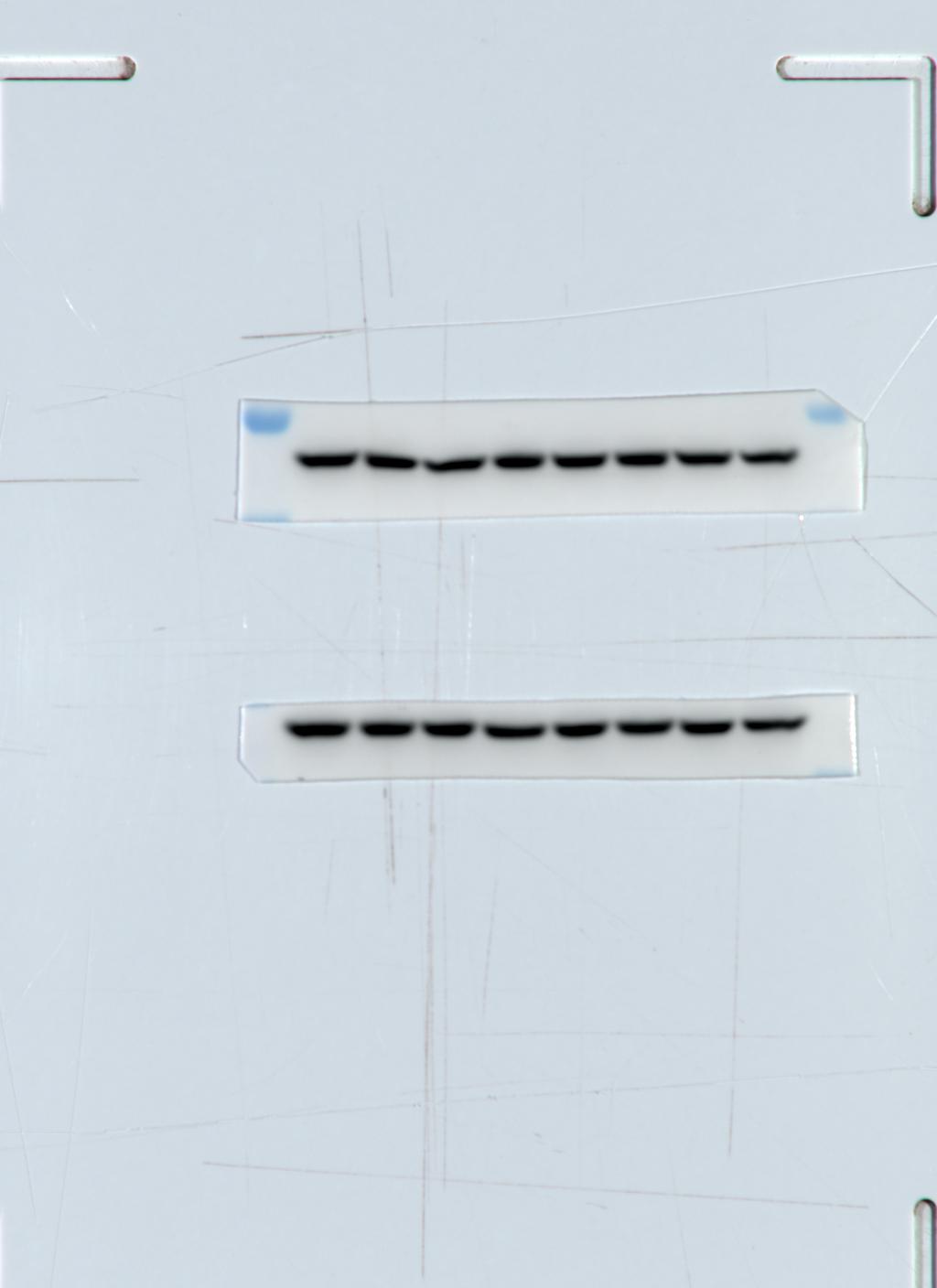

Supplement: Figure 5—figure supplement 1—source data 2. [file elife-76183-fig5-figsupp1-data2.zip › Figure 5-figure supplement 1-source data 2/Figure 5 S1B INPUT-Tubulin.jpg]

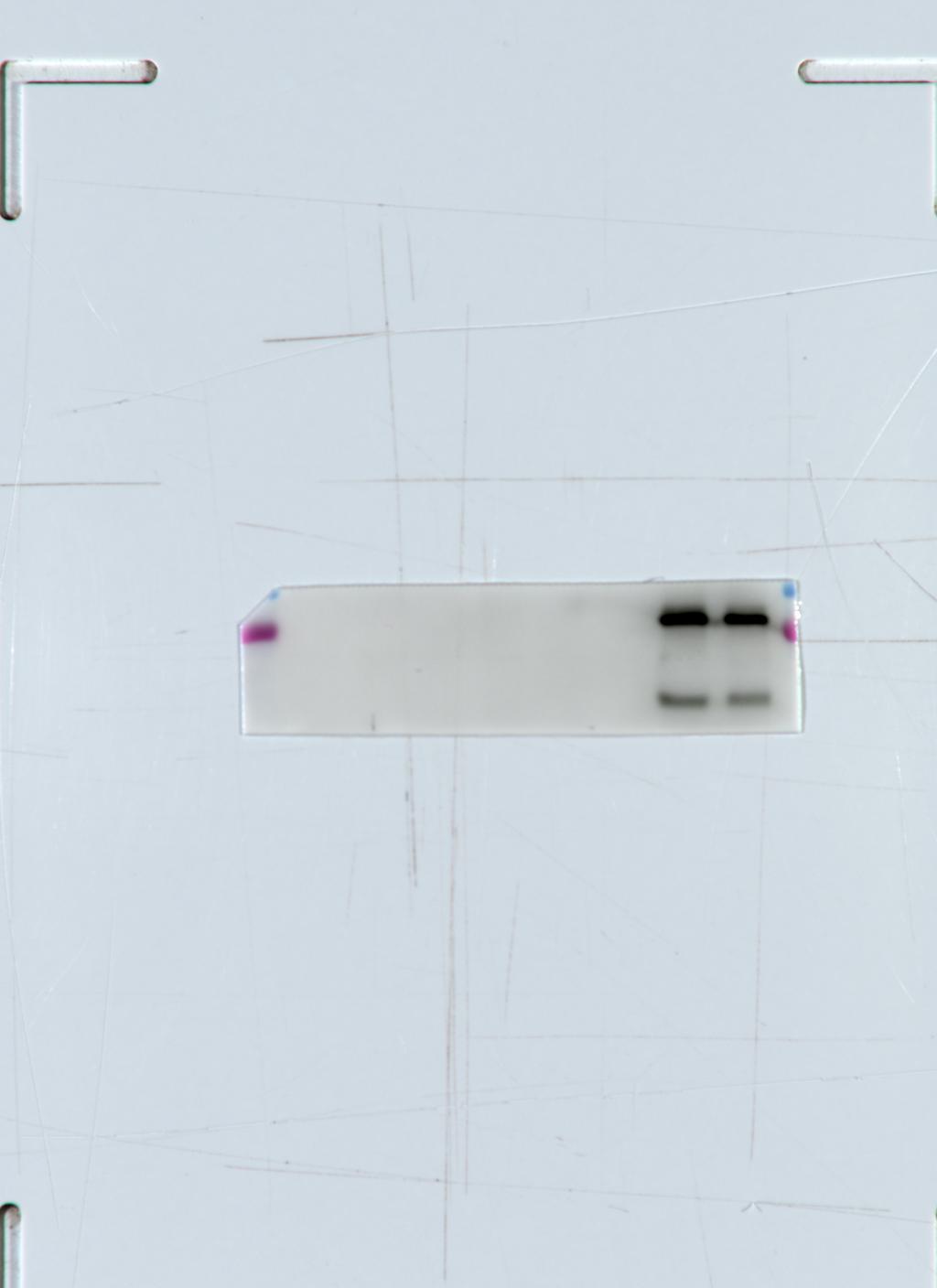

Supplement: Figure 5—figure supplement 1—source data 2. [file elife-76183-fig5-figsupp1-data2.zip › Figure 5-figure supplement 1-source data 2/Figure 5 S1B IP-FLAG.jpg]

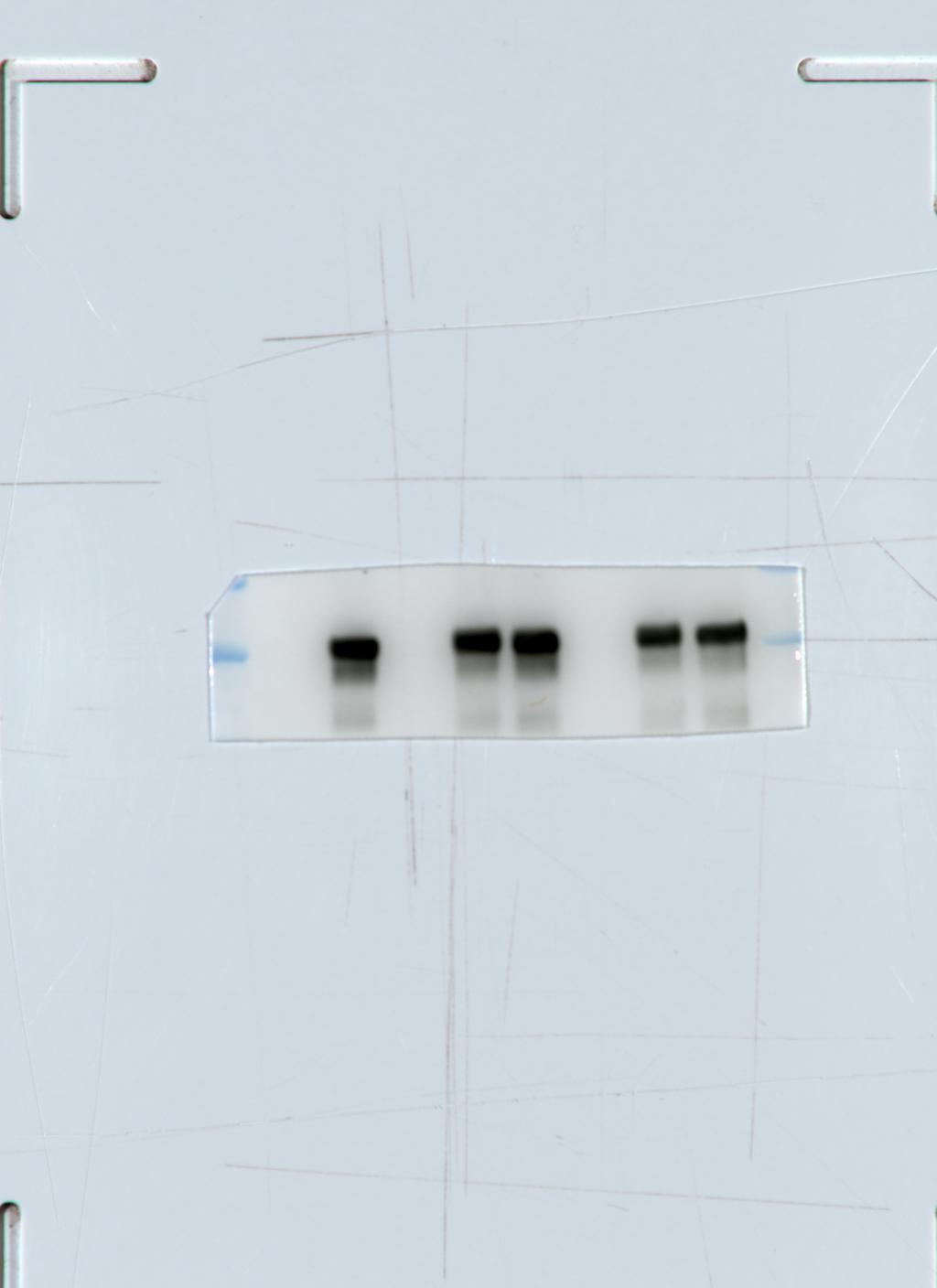

Supplement: Figure 5—figure supplement 1—source data 2. [file elife-76183-fig5-figsupp1-data2.zip › Figure 5-figure supplement 1-source data 2/Figure 5 S1B IP-Myc.jpg]

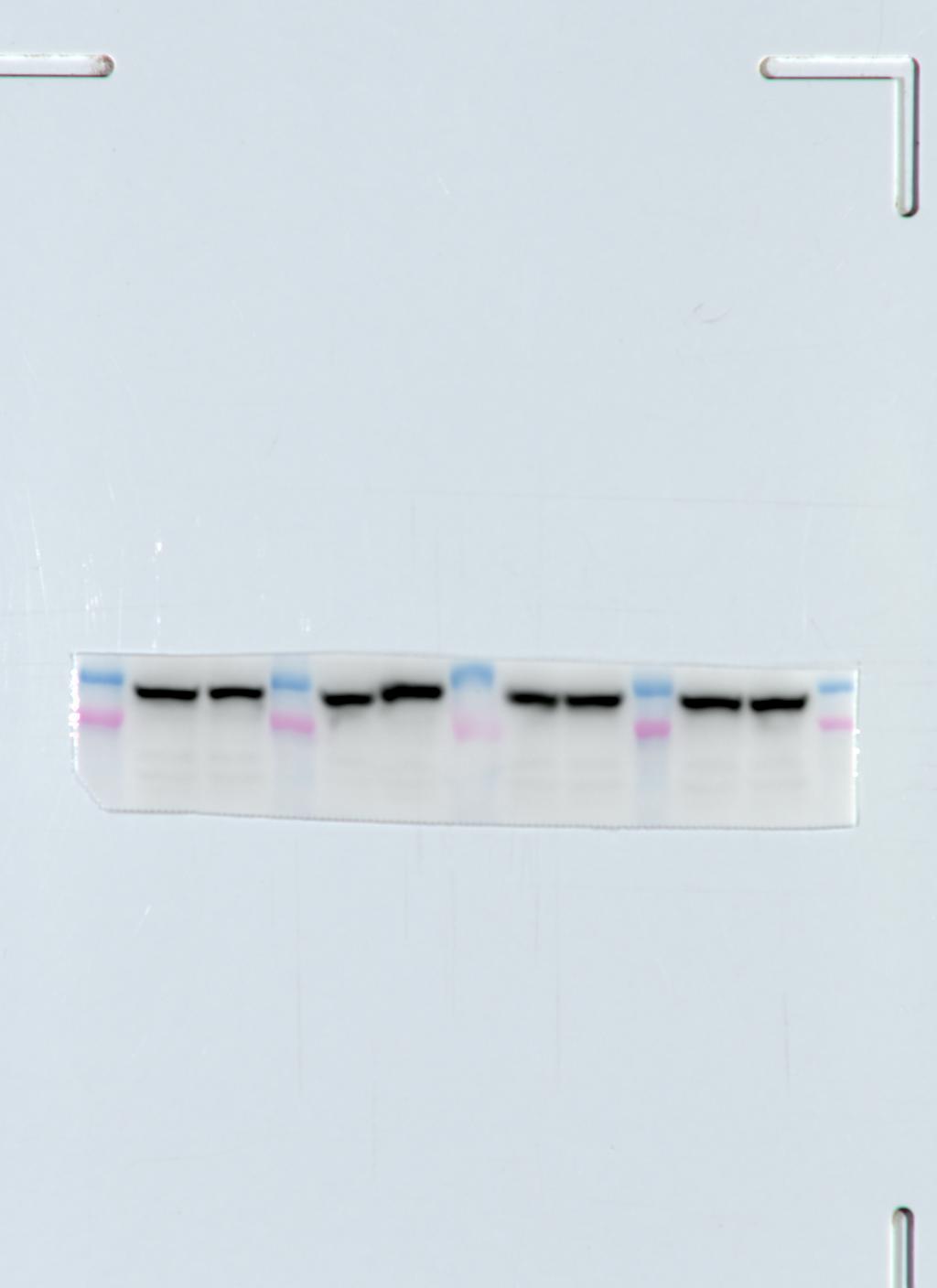

Supplement: Figure 6—source data 1. [file elife-76183-fig6-data1.zip › Figure 6-source data 1/Figure 6A down INPUT-FER.jpg]

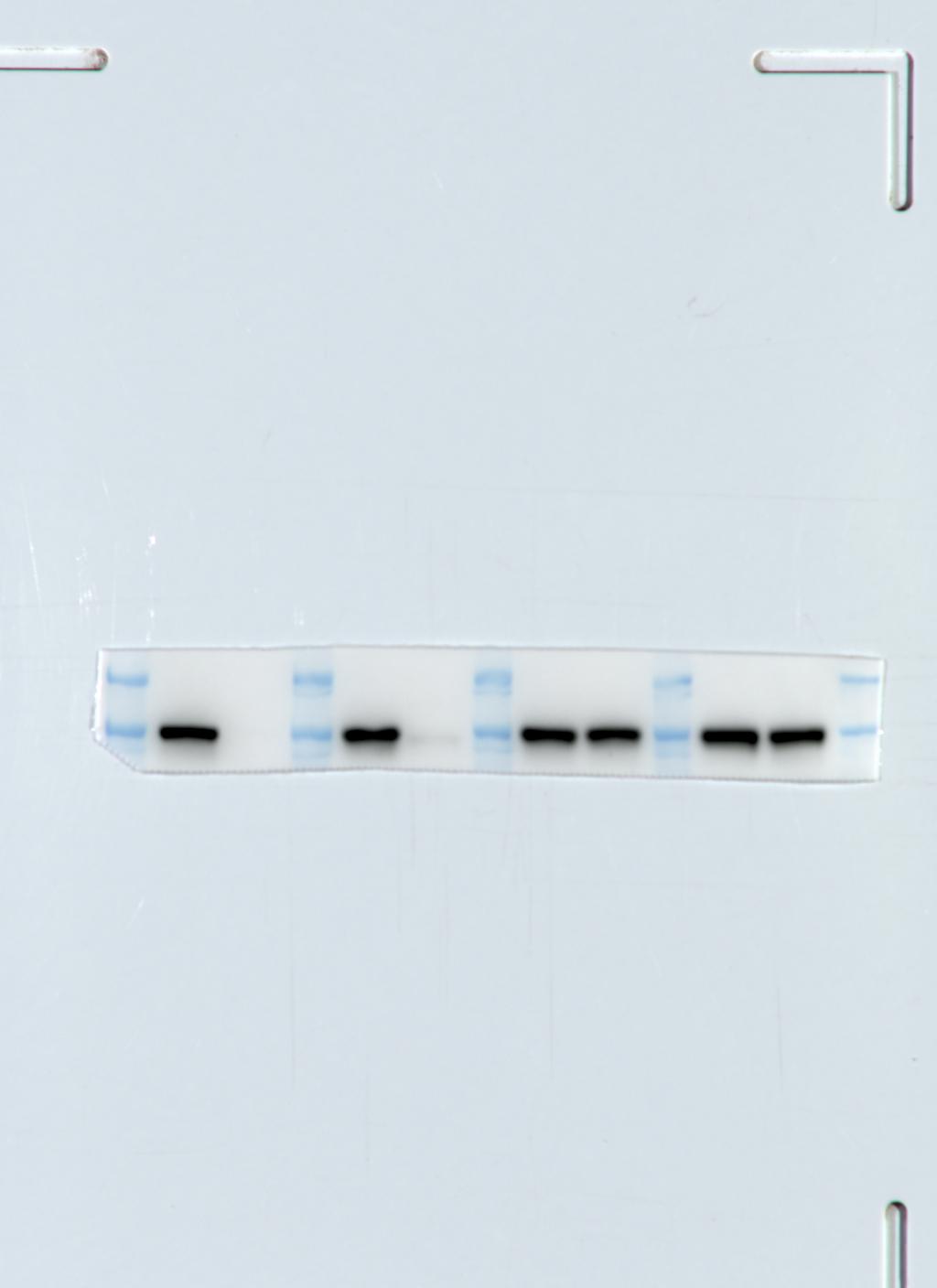

Supplement: Figure 6—source data 1. [file elife-76183-fig6-data1.zip › Figure 6-source data 1/Figure 6A down INPUT-IRS4.jpg]

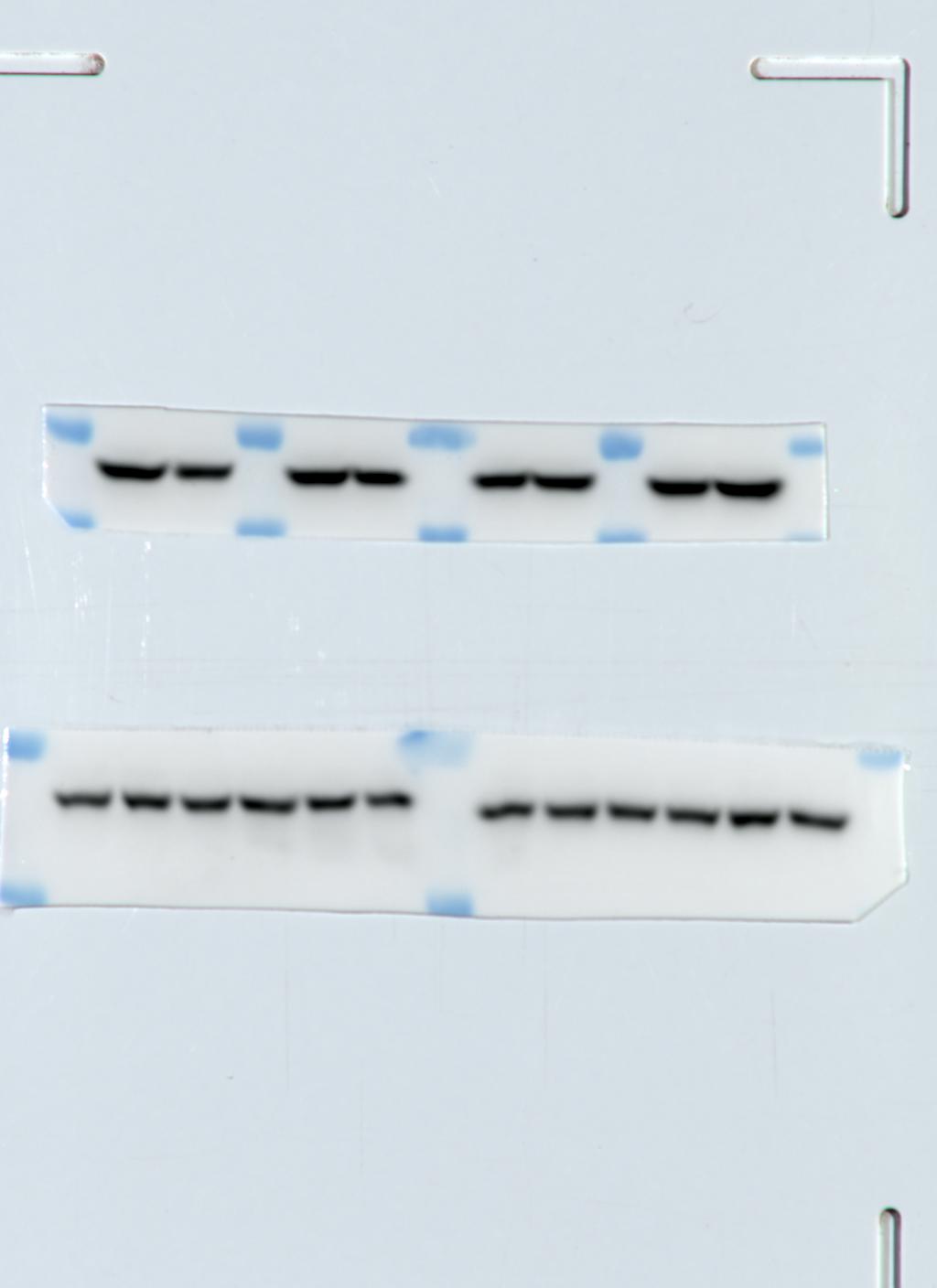

Supplement: Figure 6—source data 1. [file elife-76183-fig6-data1.zip › Figure 6-source data 1/Figure 6A down INPUT-Tubulin.jpg]

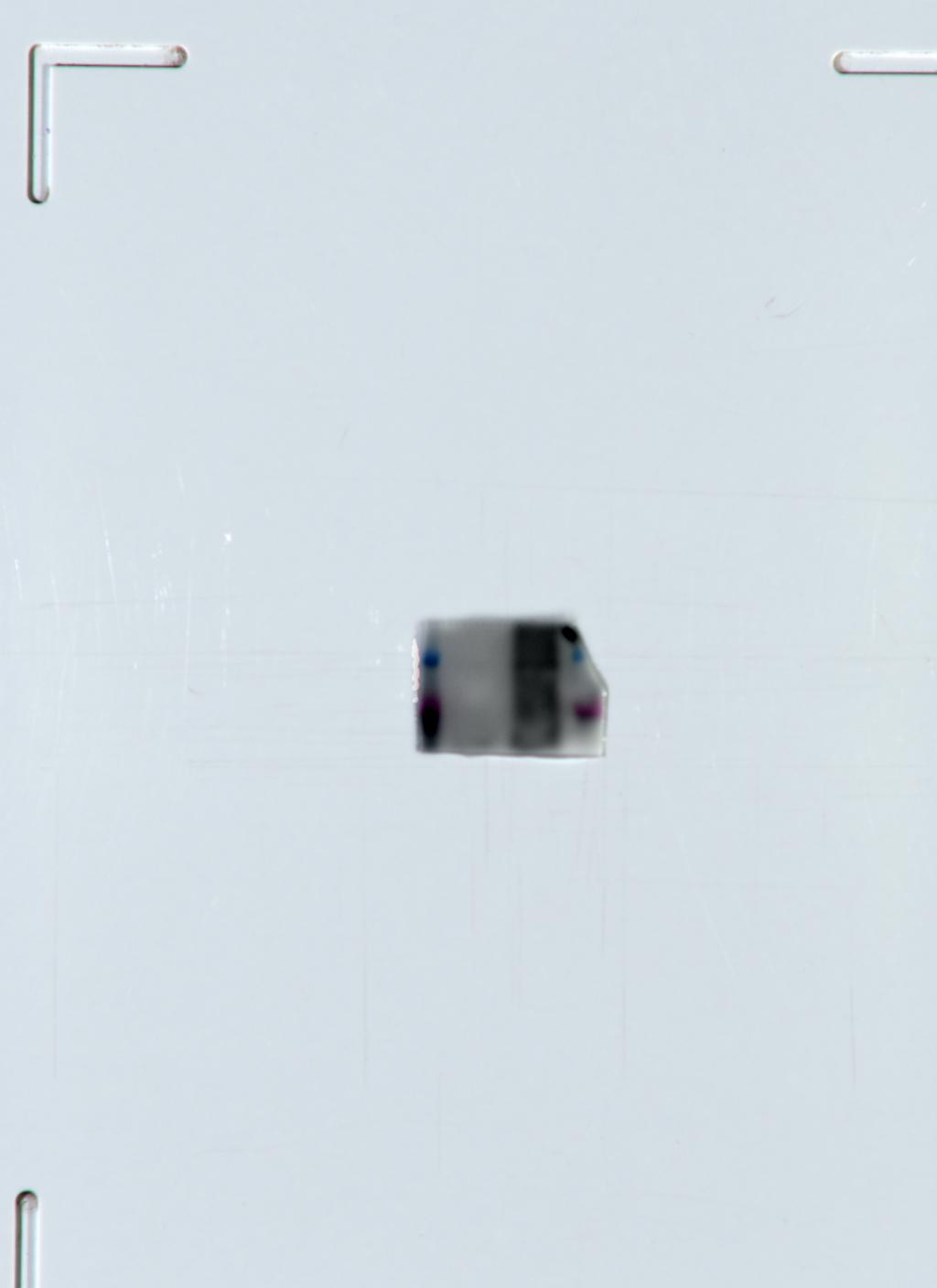

Supplement: Figure 6—source data 1. [file elife-76183-fig6-data1.zip › Figure 6-source data 1/Figure 6A down IP-FER.jpg]

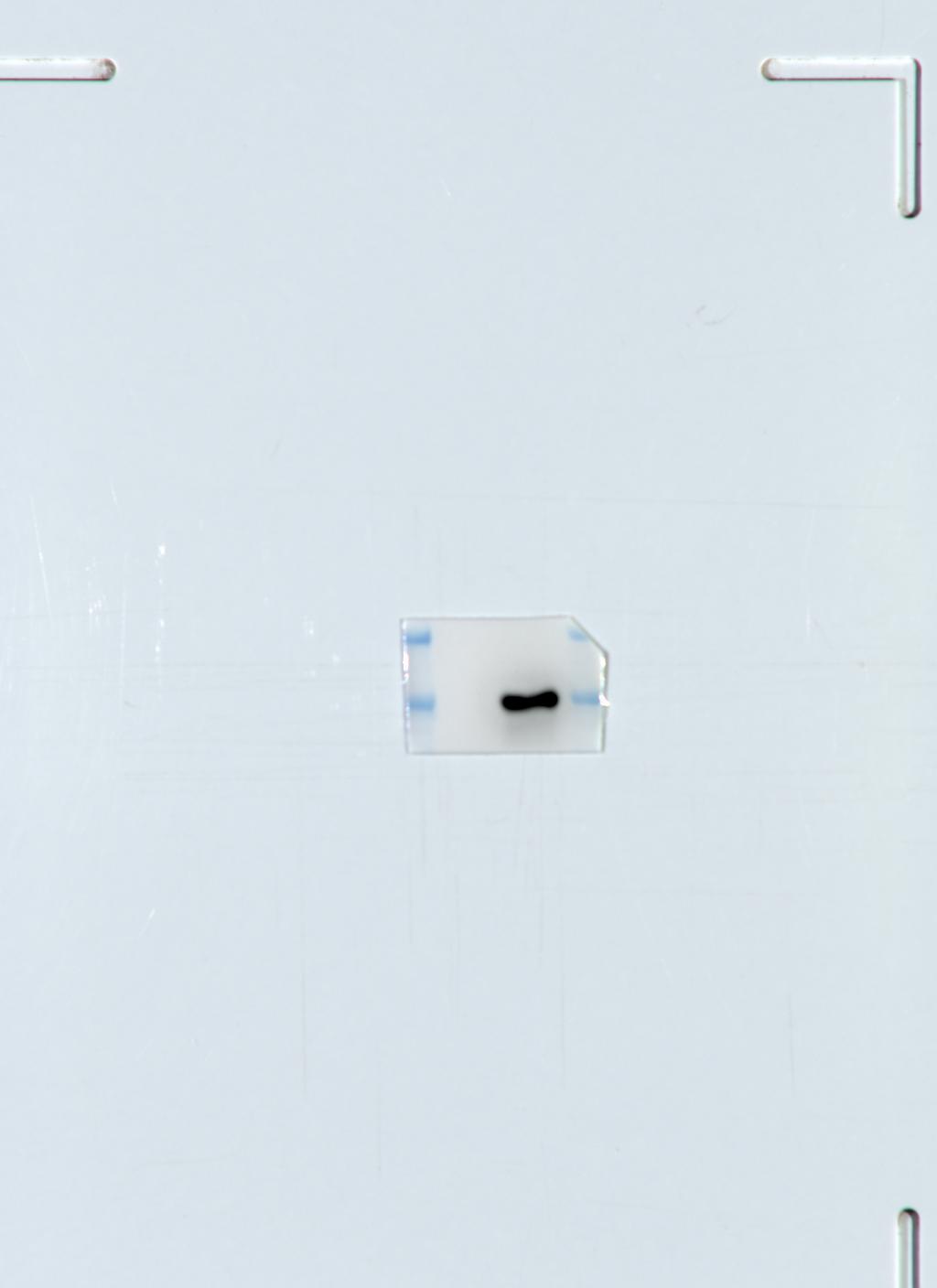

Supplement: Figure 6—source data 1. [file elife-76183-fig6-data1.zip › Figure 6-source data 1/Figure 6A down IP-IRS4.jpg]

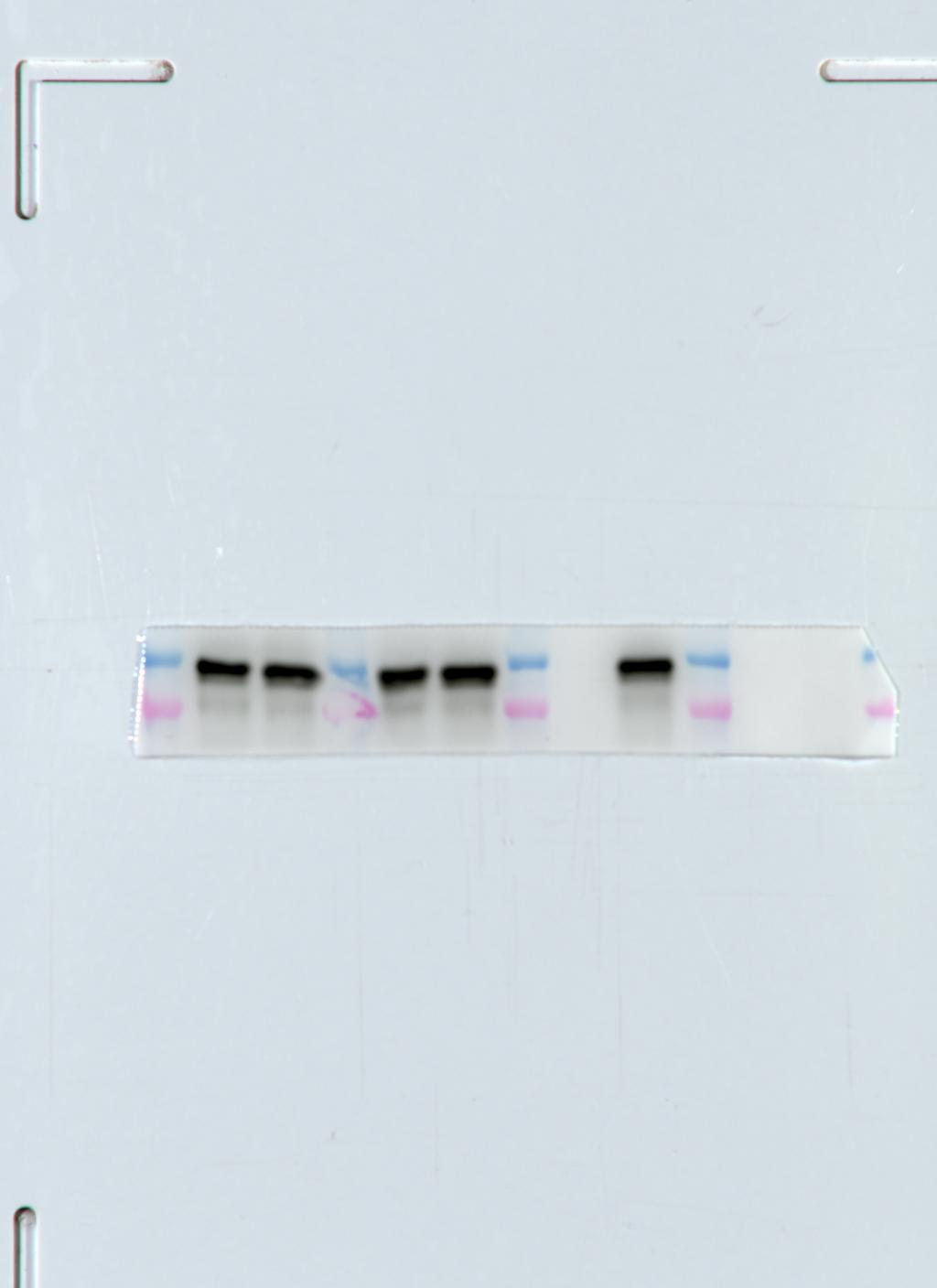

Supplement: Figure 6—source data 1. [file elife-76183-fig6-data1.zip › Figure 6-source data 1/Figure 6A up IP-FER.jpg]

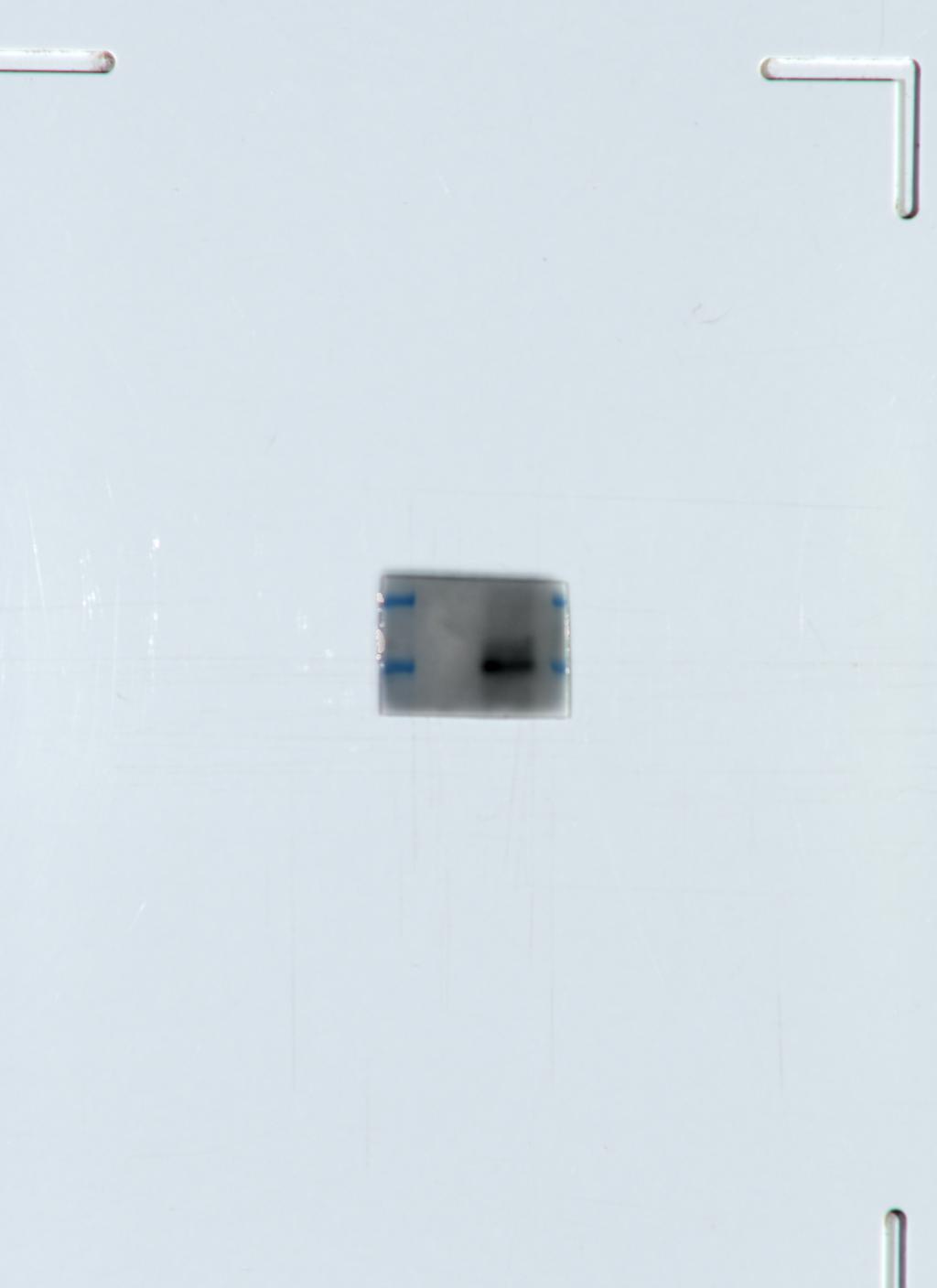

Supplement: Figure 6—source data 1. [file elife-76183-fig6-data1.zip › Figure 6-source data 1/Figure 6A up IP-IRS4.jpg]

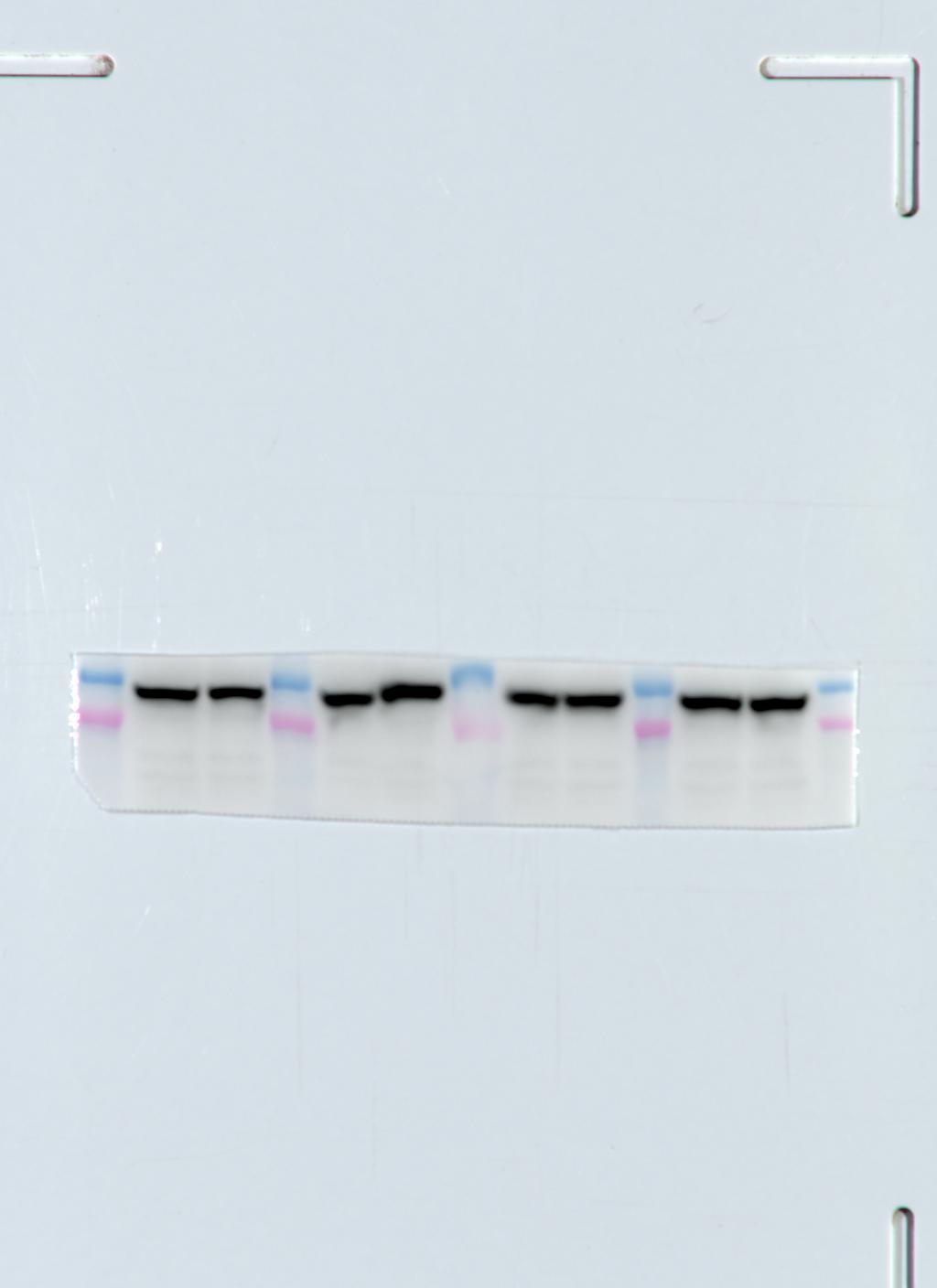

Supplement: Figure 6—source data 2. [file elife-76183-fig6-data2.zip › Figure 6-source data 2/Figure 6B down INPUT-FER.jpg]

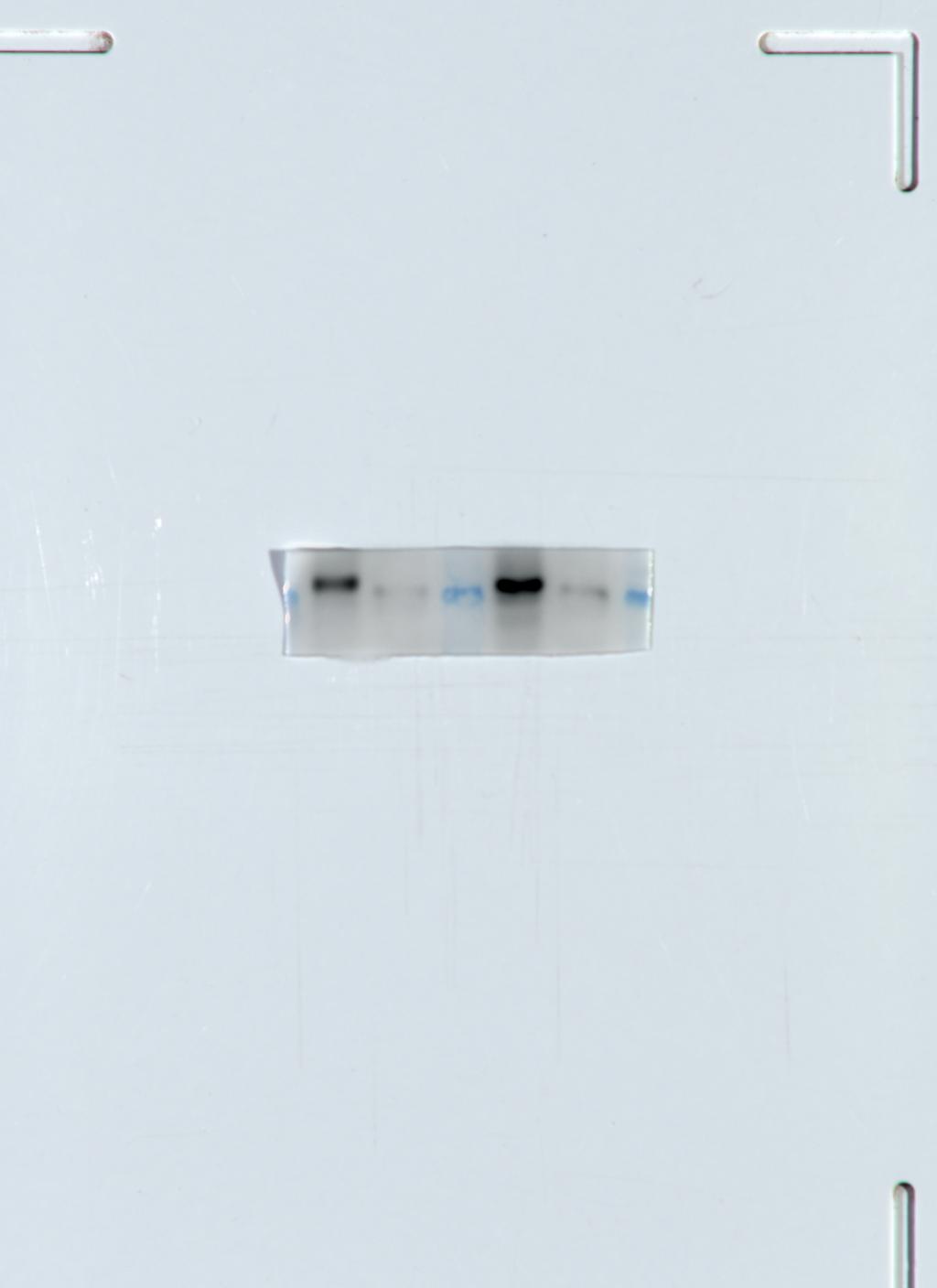

Supplement: Figure 6—source data 2. [file elife-76183-fig6-data2.zip › Figure 6-source data 2/Figure 6B down IP-IRS4.jpg]

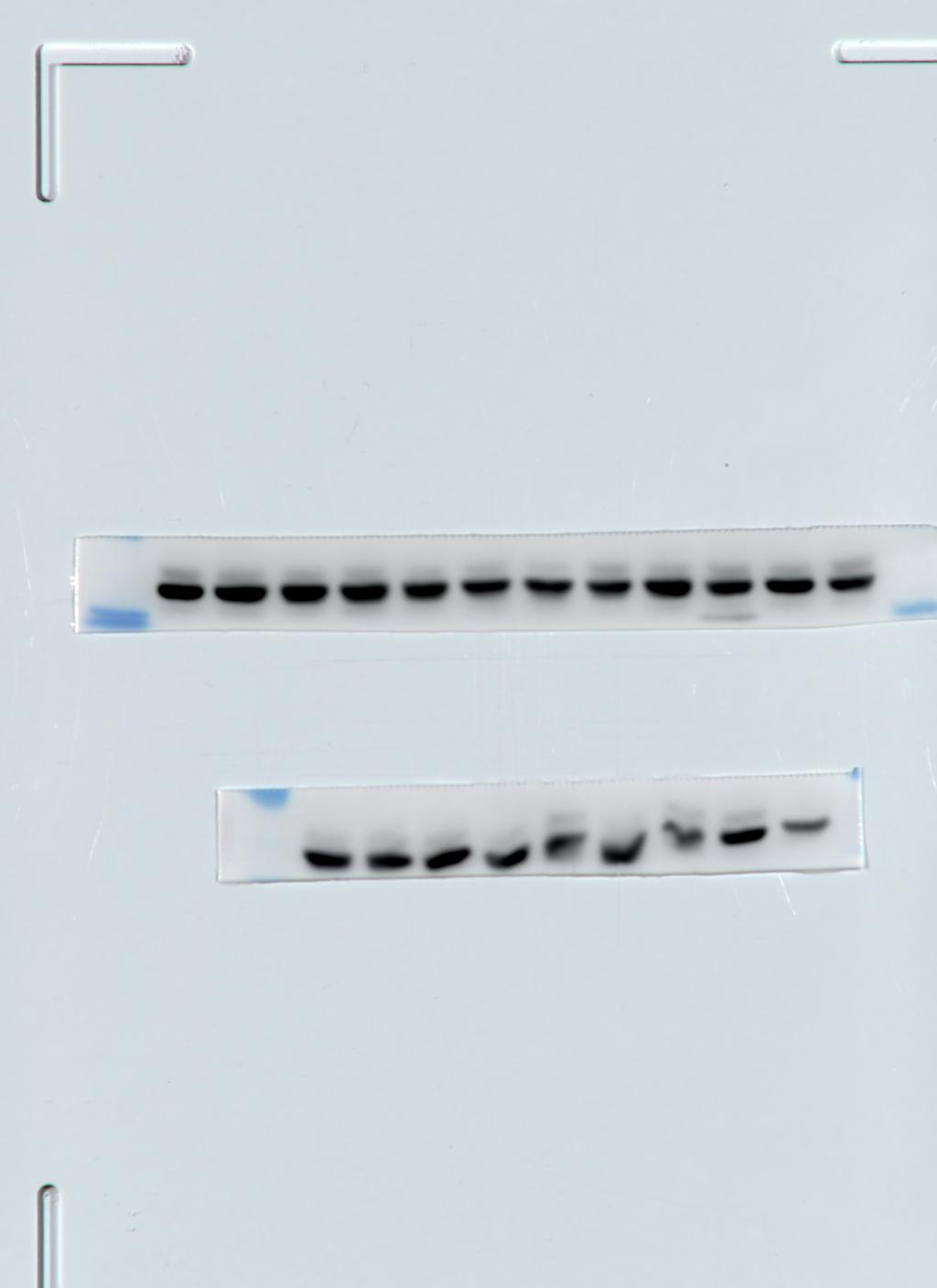

Supplement: Figure 6—source data 2. [file elife-76183-fig6-data2.zip › Figure 6-source data 2/Figure 6B up INPUT-Actin.jpg]

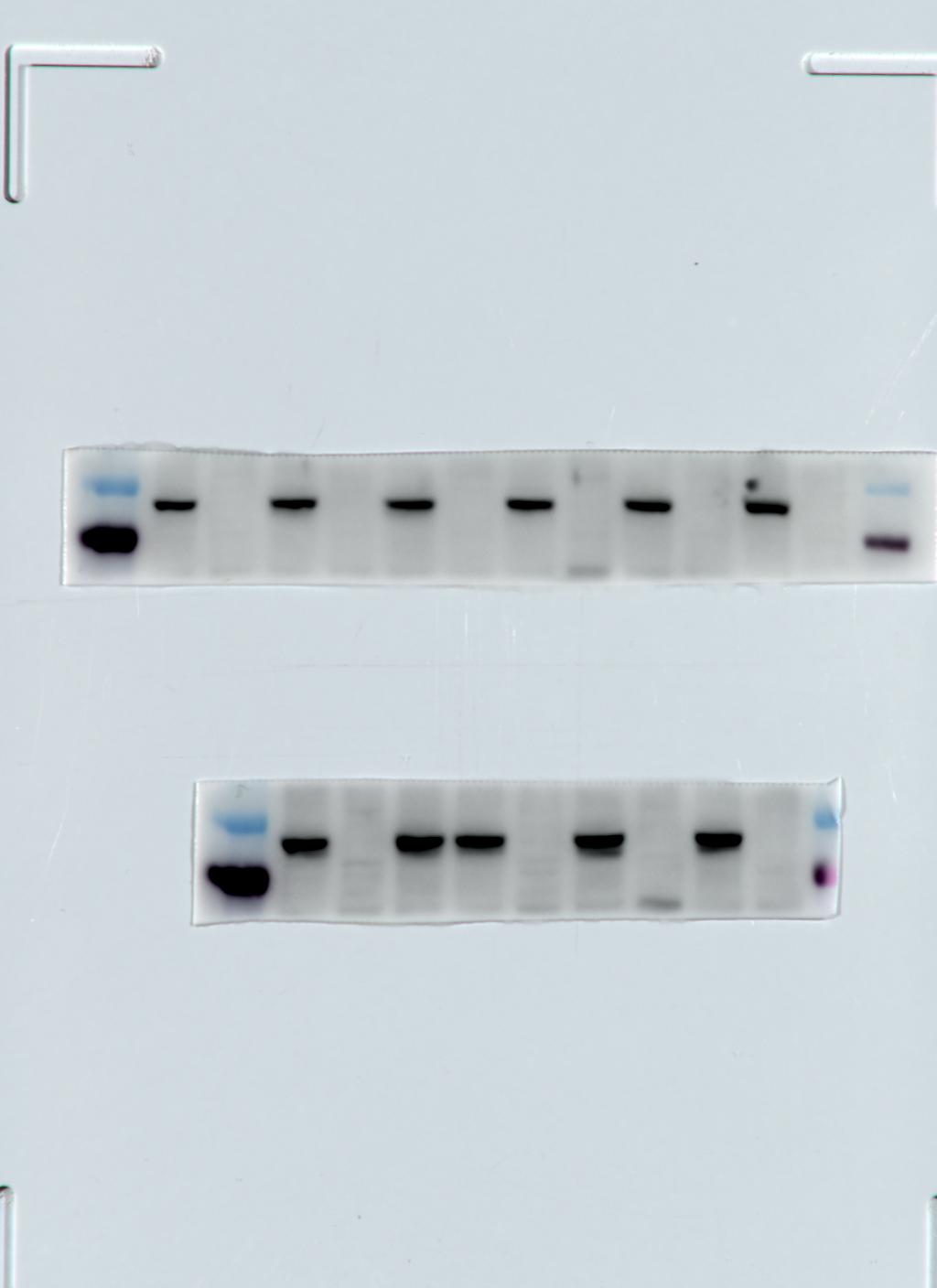

Supplement: Figure 6—source data 2. [file elife-76183-fig6-data2.zip › Figure 6-source data 2/Figure 6B up INPUT-FER.jpg]

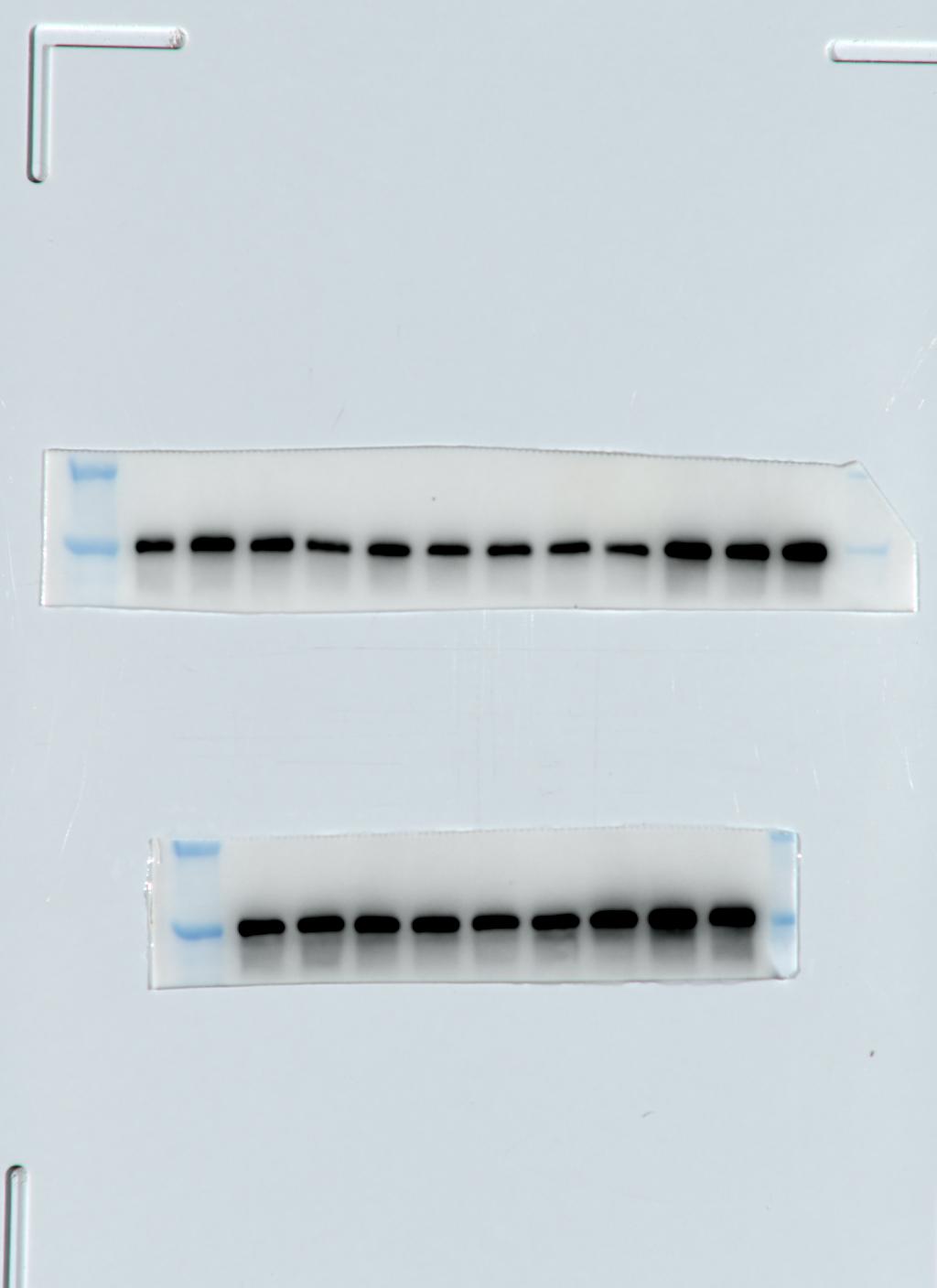

Supplement: Figure 6—source data 2. [file elife-76183-fig6-data2.zip › Figure 6-source data 2/Figure 6B up INPUT-IRS4.jpg]

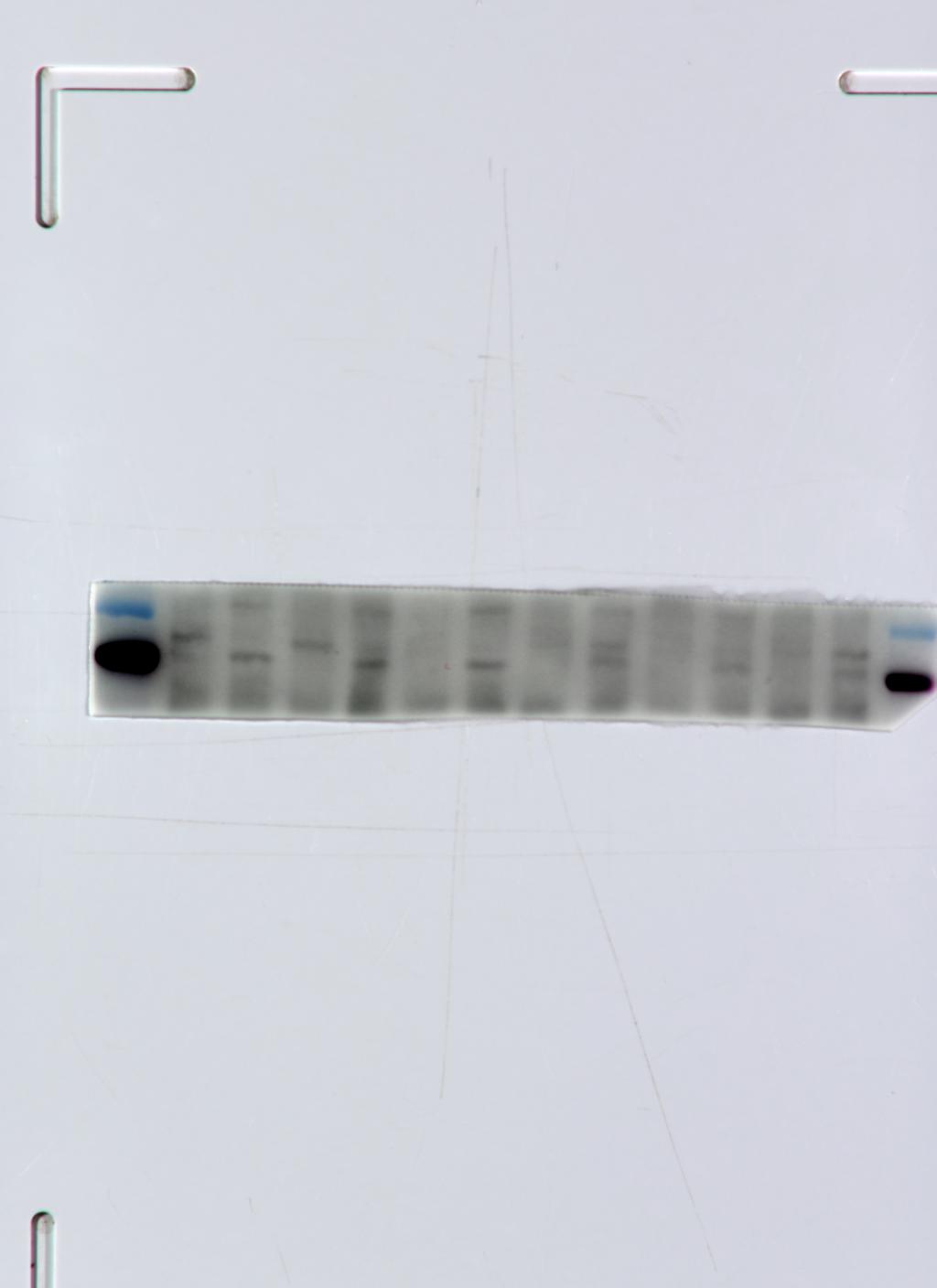

Supplement: Figure 6—source data 2. [file elife-76183-fig6-data2.zip › Figure 6-source data 2/Figure 6B up IP-FER.jpg]

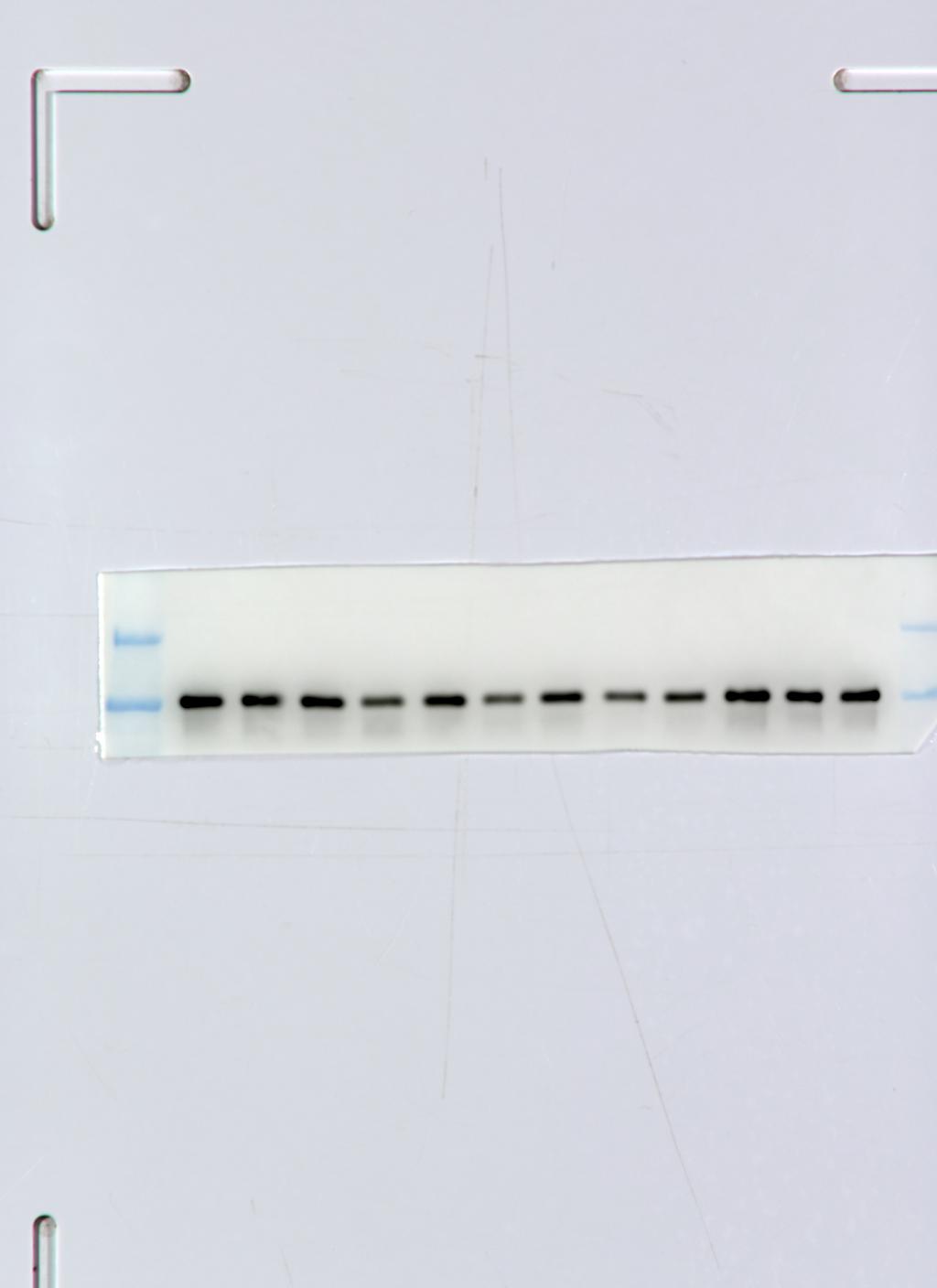

Supplement: Figure 6—source data 2. [file elife-76183-fig6-data2.zip › Figure 6-source data 2/Figure 6B up IP-IRS4.jpg]

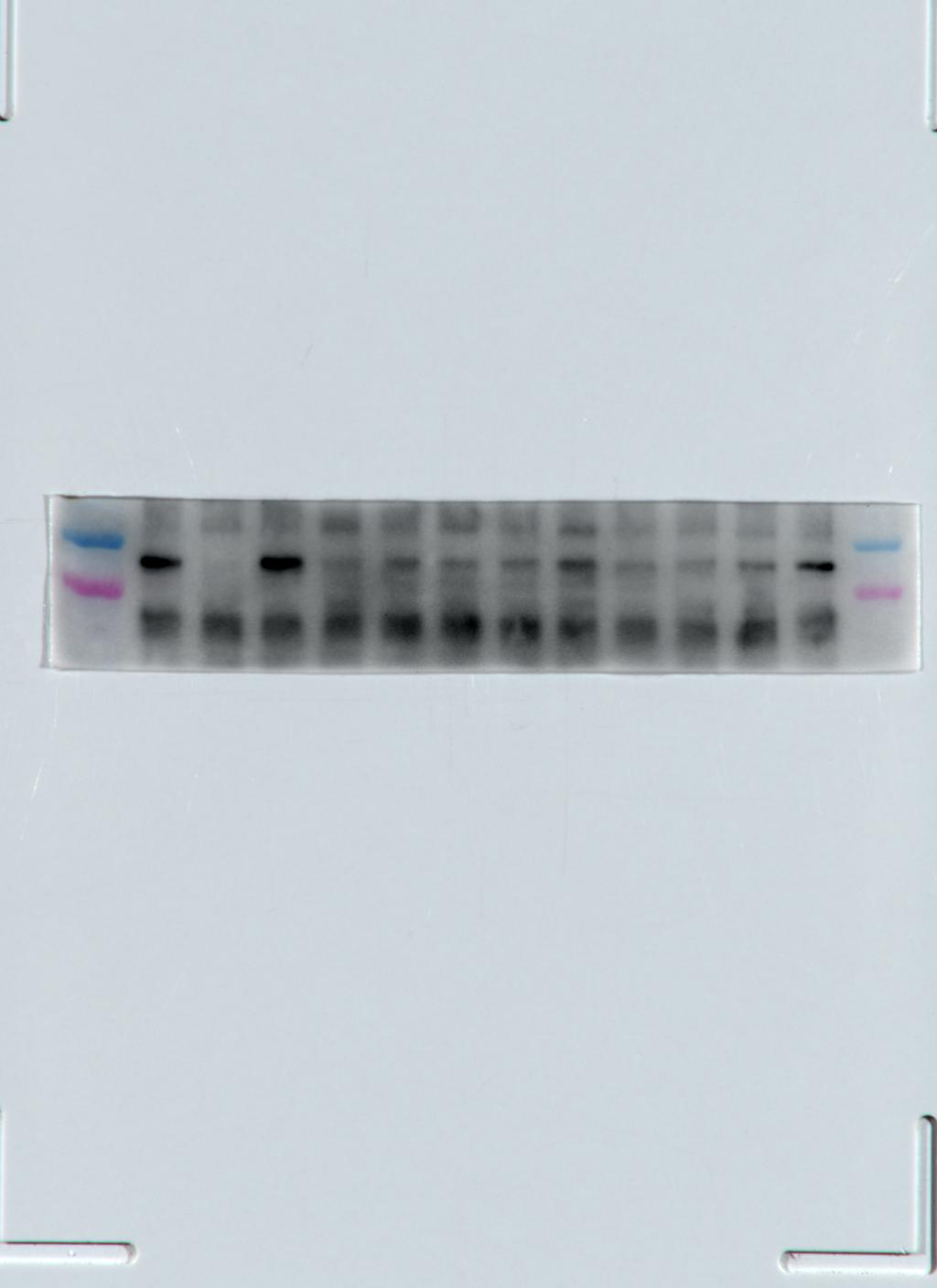

Supplement: Figure 6—source data 2. [file elife-76183-fig6-data2.zip › Figure 6-source data 2/Figure 6B up IP-pY402 FER.jpg]

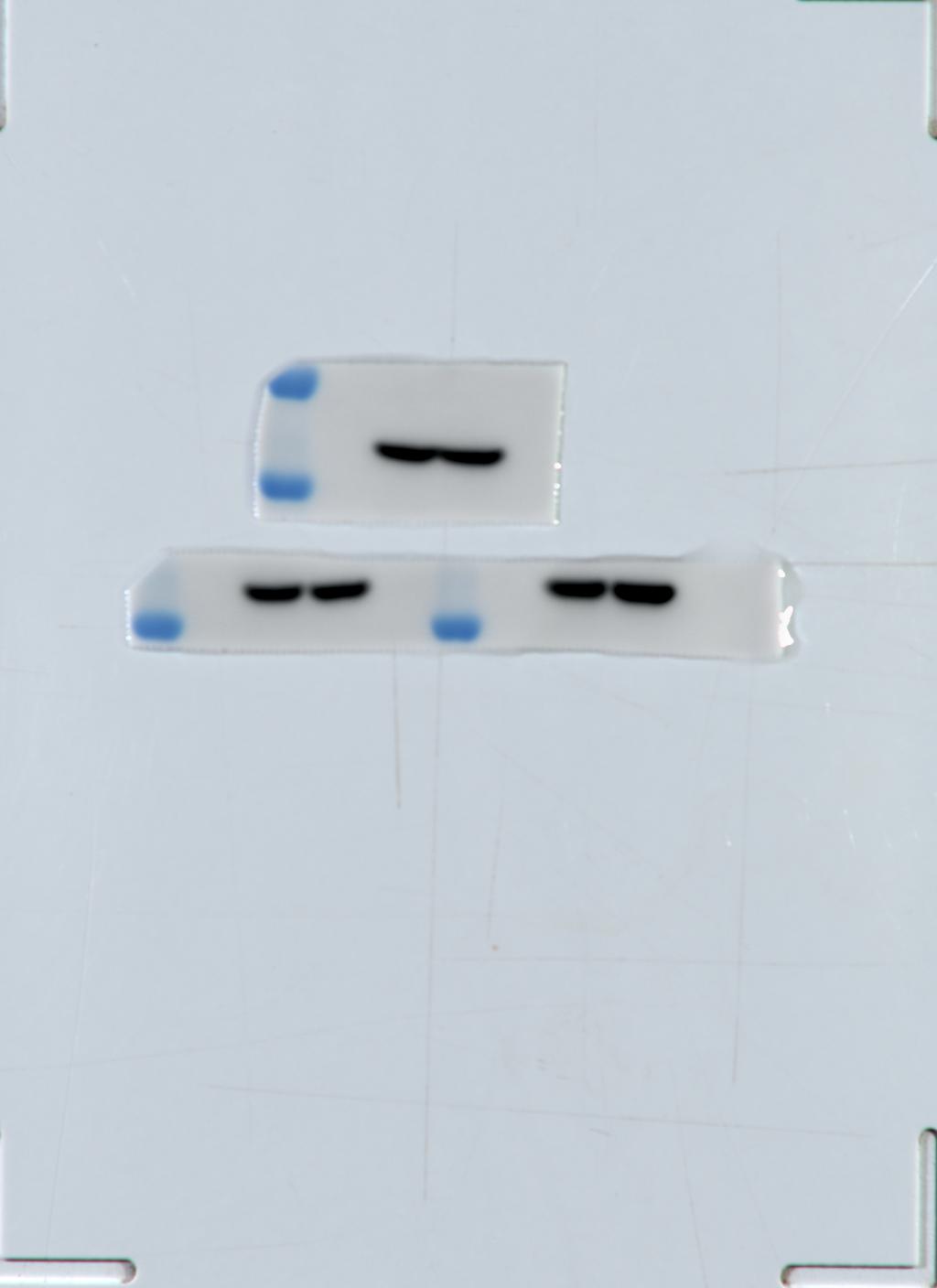

Supplement: Figure 6—source data 3. [file elife-76183-fig6-data3.zip › Figure 6-source data 3/Figure 6C Actin.jpg]

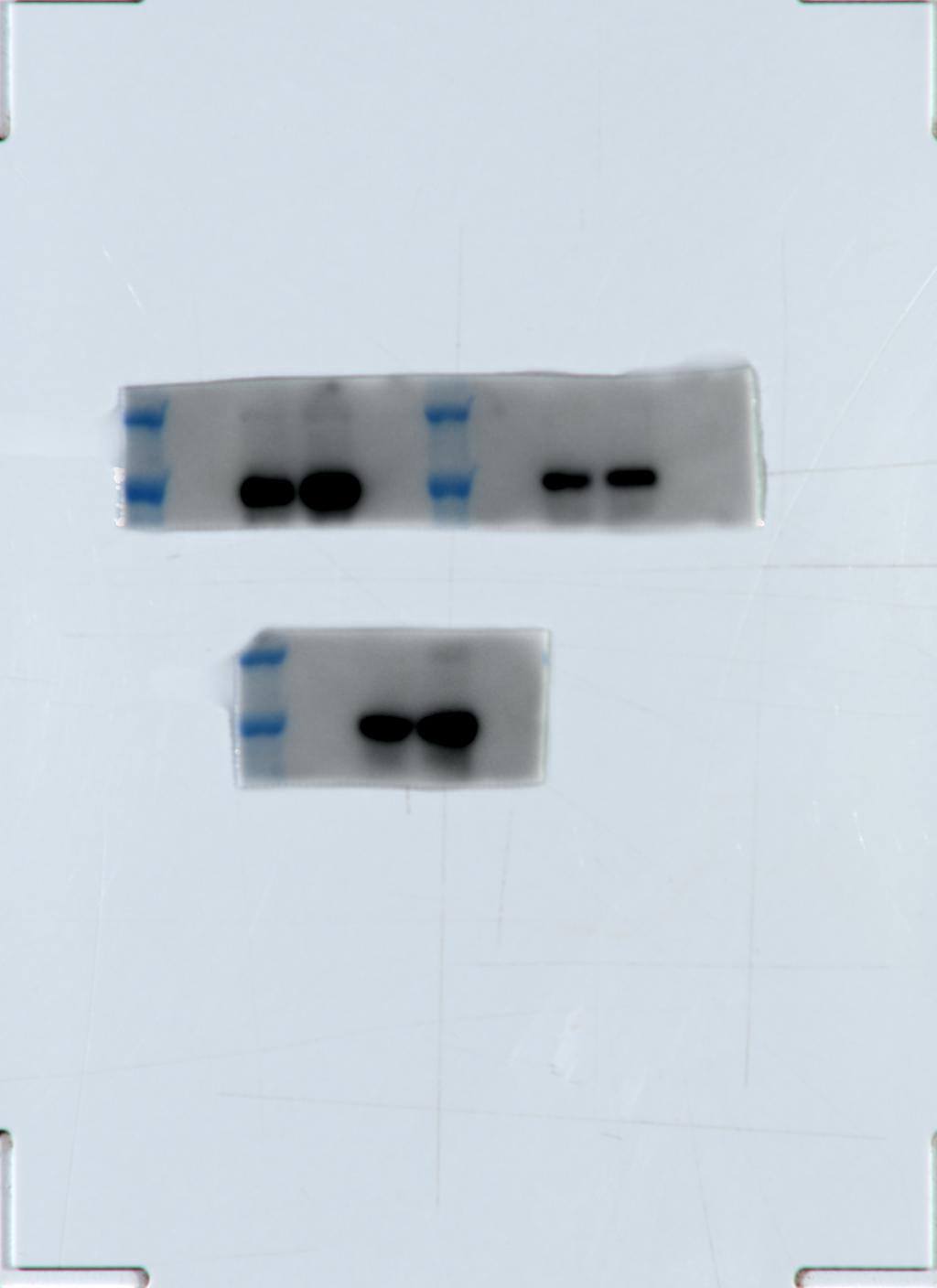

Supplement: Figure 6—source data 3. [file elife-76183-fig6-data3.zip › Figure 6-source data 3/Figure 6C input WB IRS4.jpg]

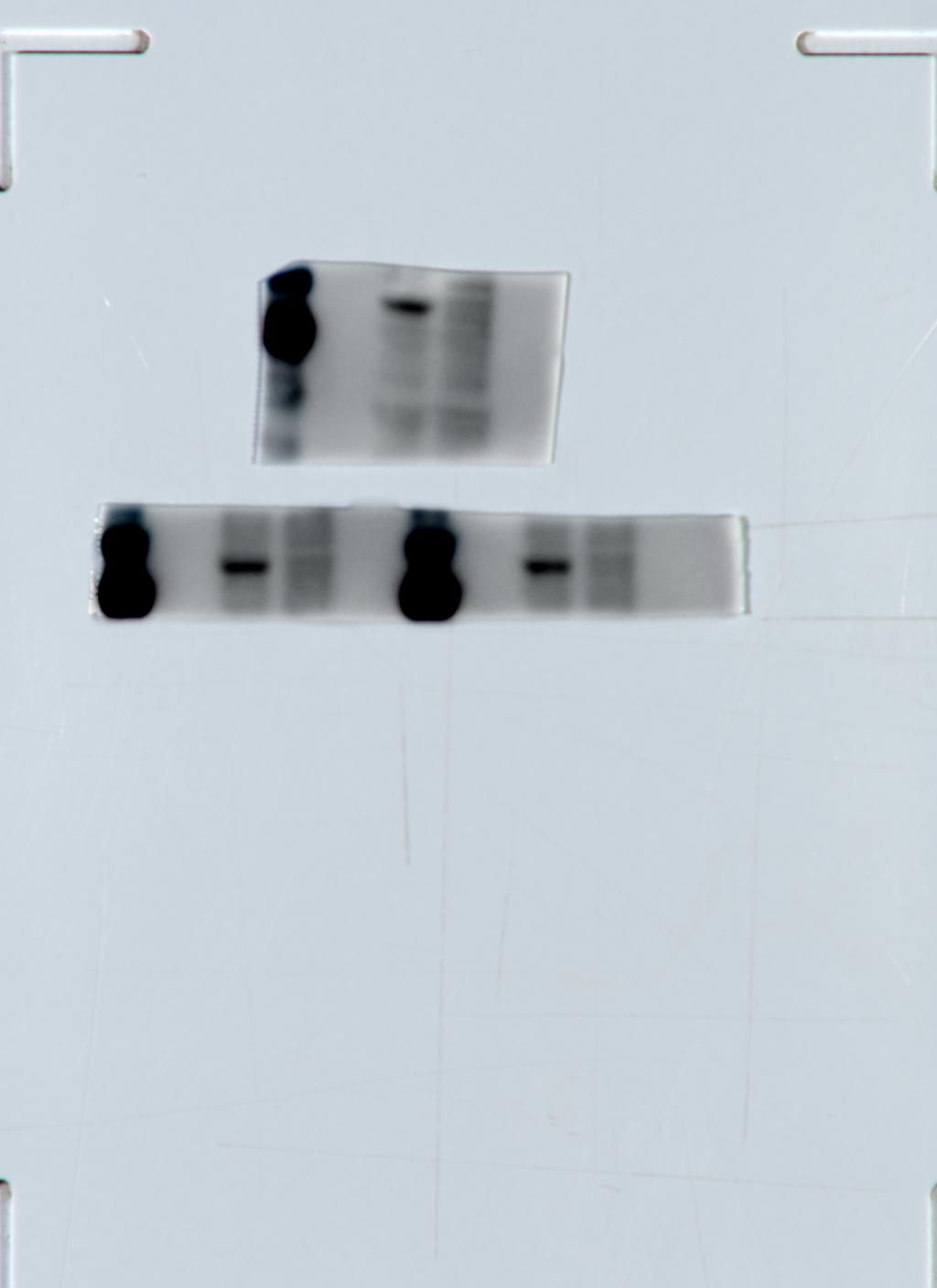

Supplement: Figure 6—source data 3. [file elife-76183-fig6-data3.zip › Figure 6-source data 3/Figure 6C input WB FER.jpg]

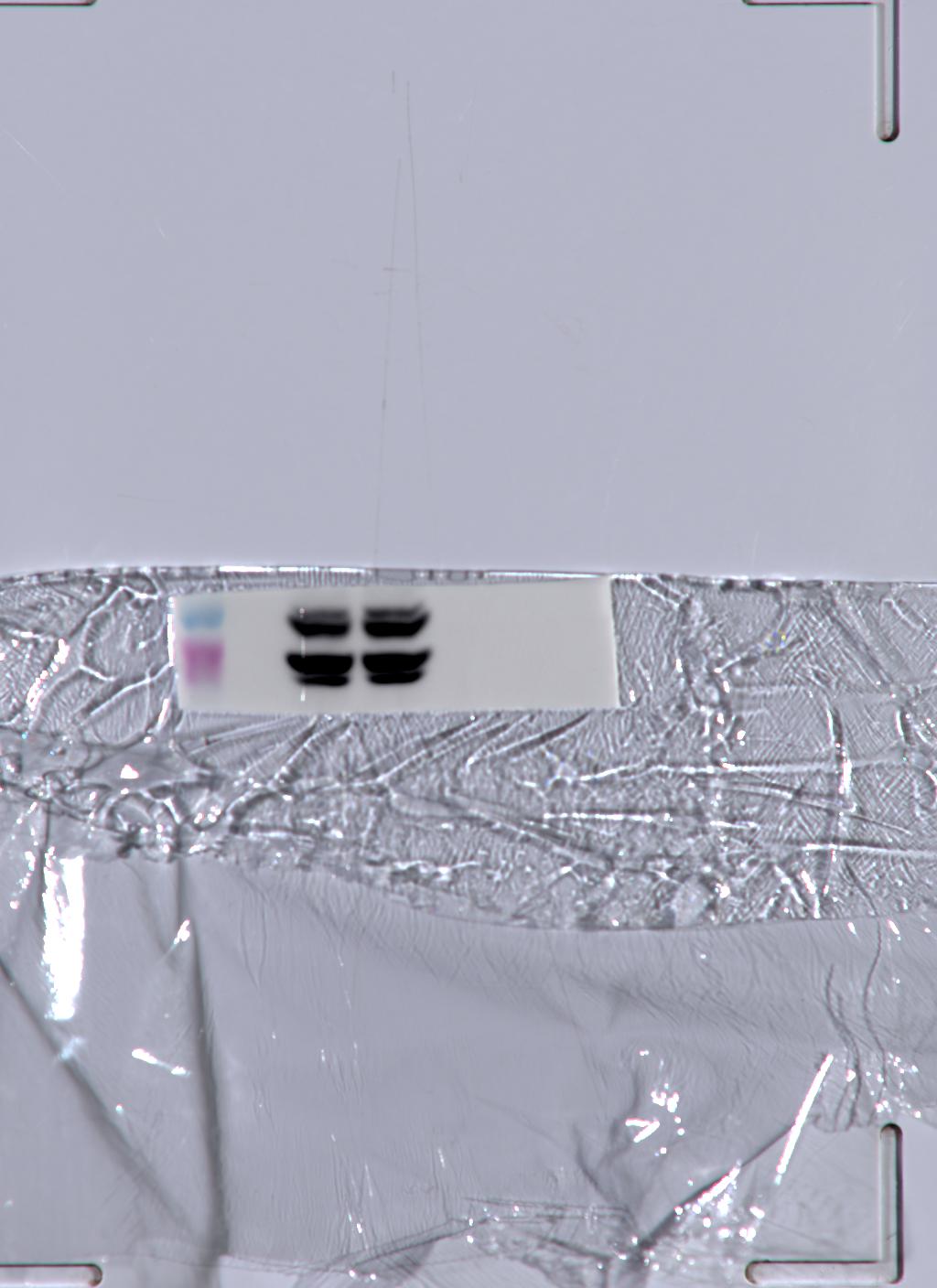

Supplement: Figure 6—source data 3. [file elife-76183-fig6-data3.zip › Figure 6-source data 3/Figure 6C Input WB PIK3R2.jpg]

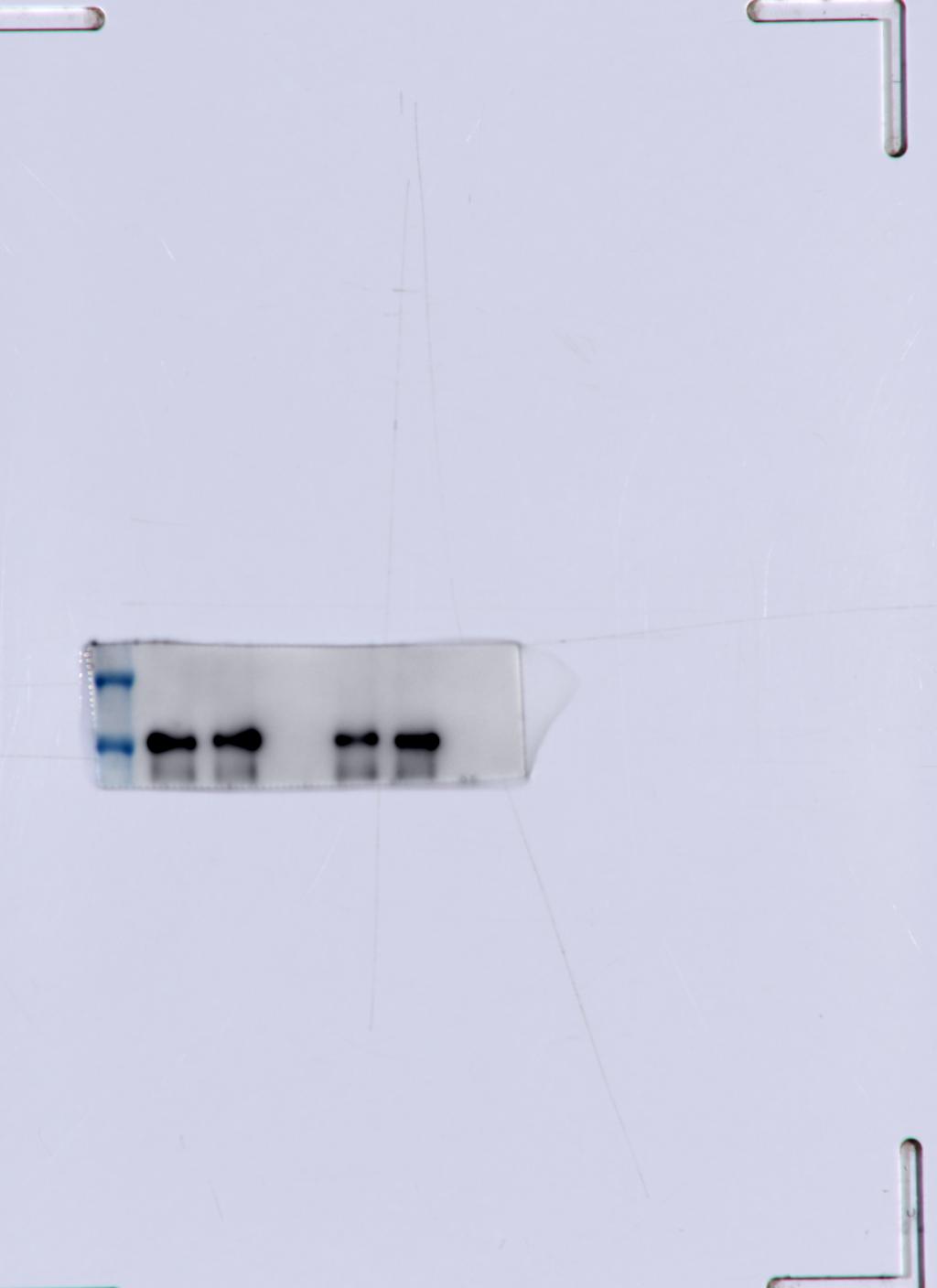

Supplement: Figure 6—source data 3. [file elife-76183-fig6-data3.zip › Figure 6-source data 3/Figure 6C IP IRS4 WB IRS4.jpg]

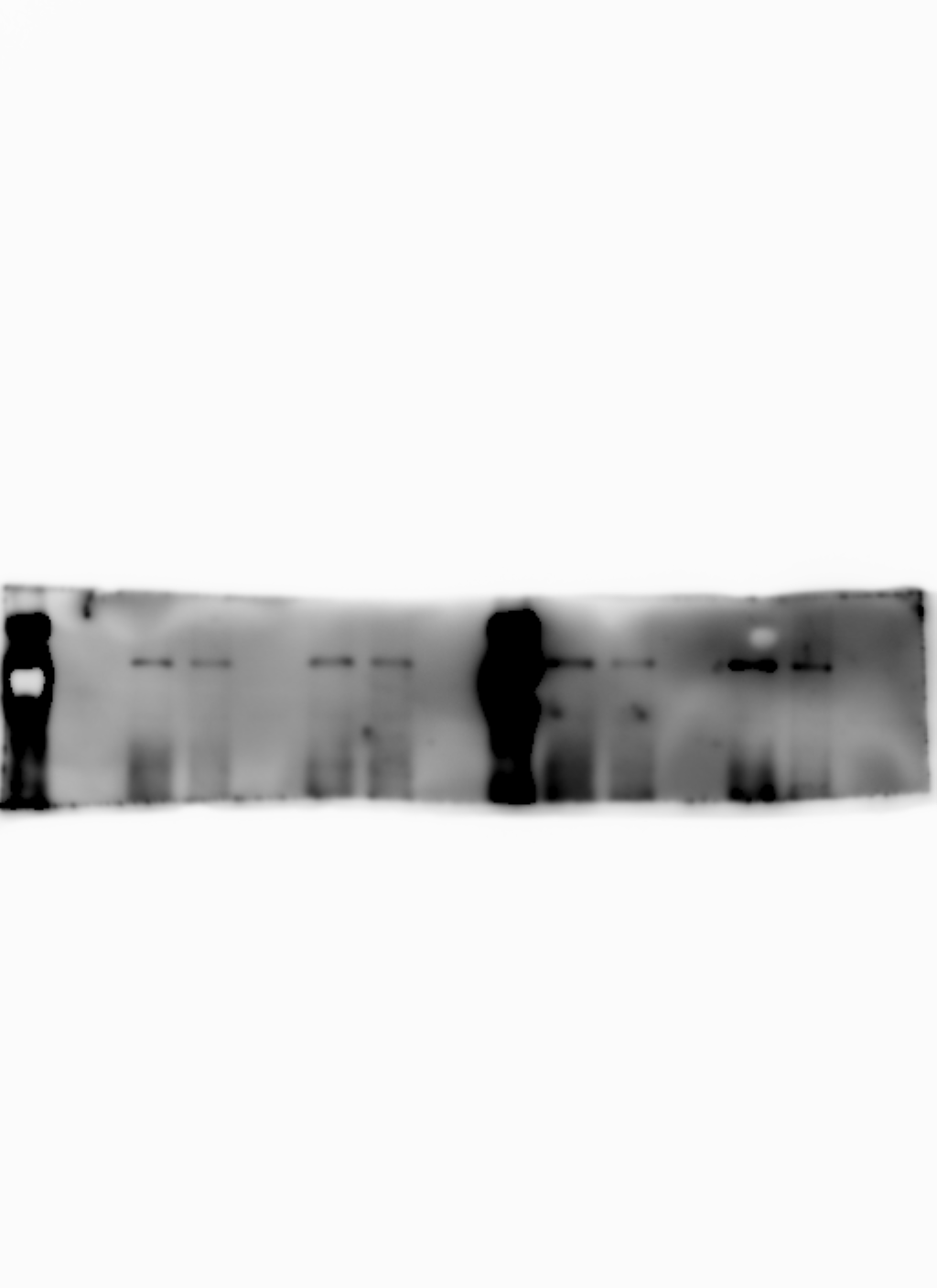

Supplement: Figure 6—source data 3. [file elife-76183-fig6-data3.zip › Figure 6-source data 3/Figure 6C IP IRS4 WB PIK3R2.tif]

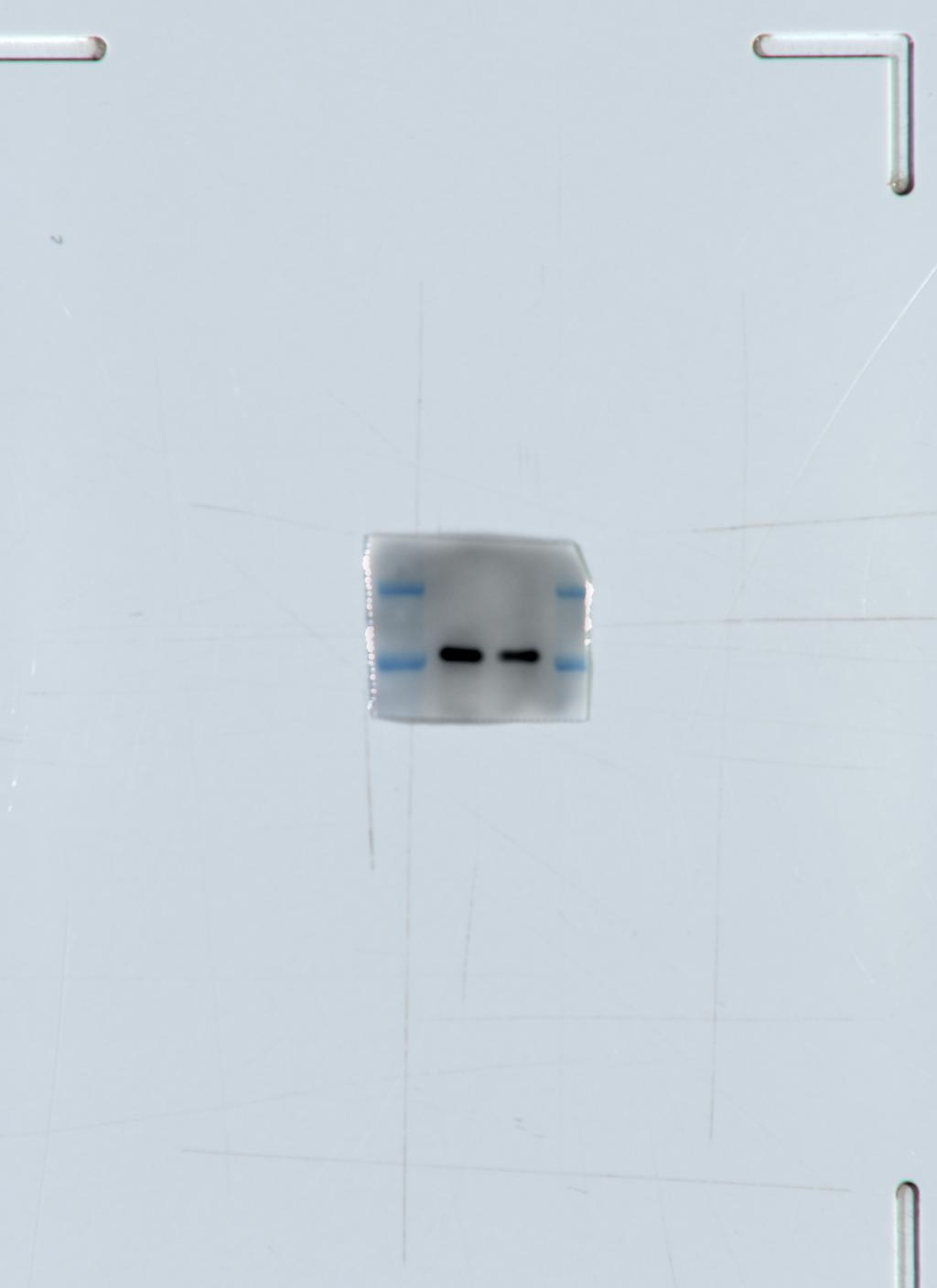

Supplement: Figure 6—source data 3. [file elife-76183-fig6-data3.zip › Figure 6-source data 3/Figure 6C IP-4G10.jpg]

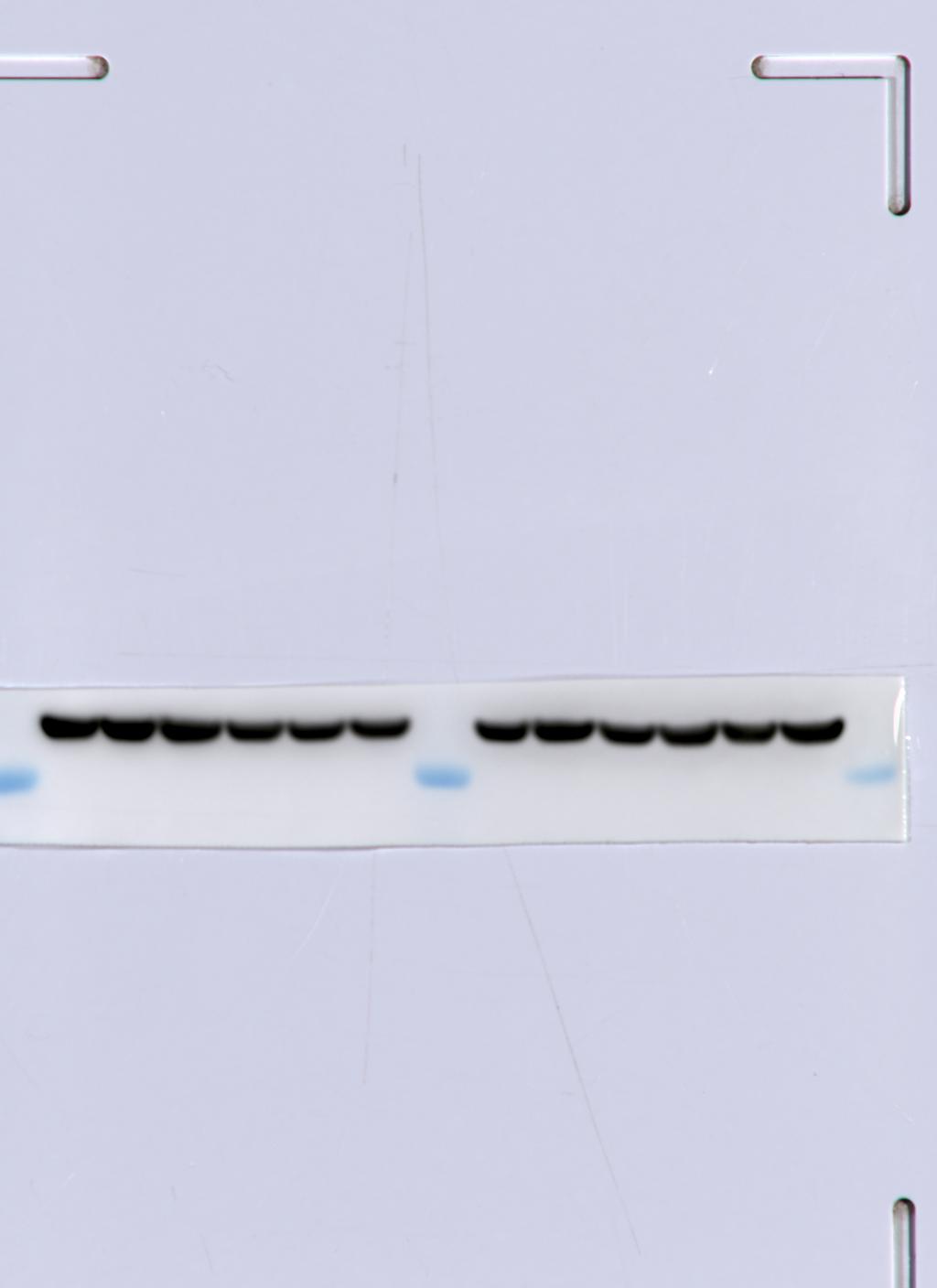

Supplement: Figure 6—source data 4. [file elife-76183-fig6-data4.zip › Figure 6-source data 4/Figure 6D INPUT-Actin.jpg]

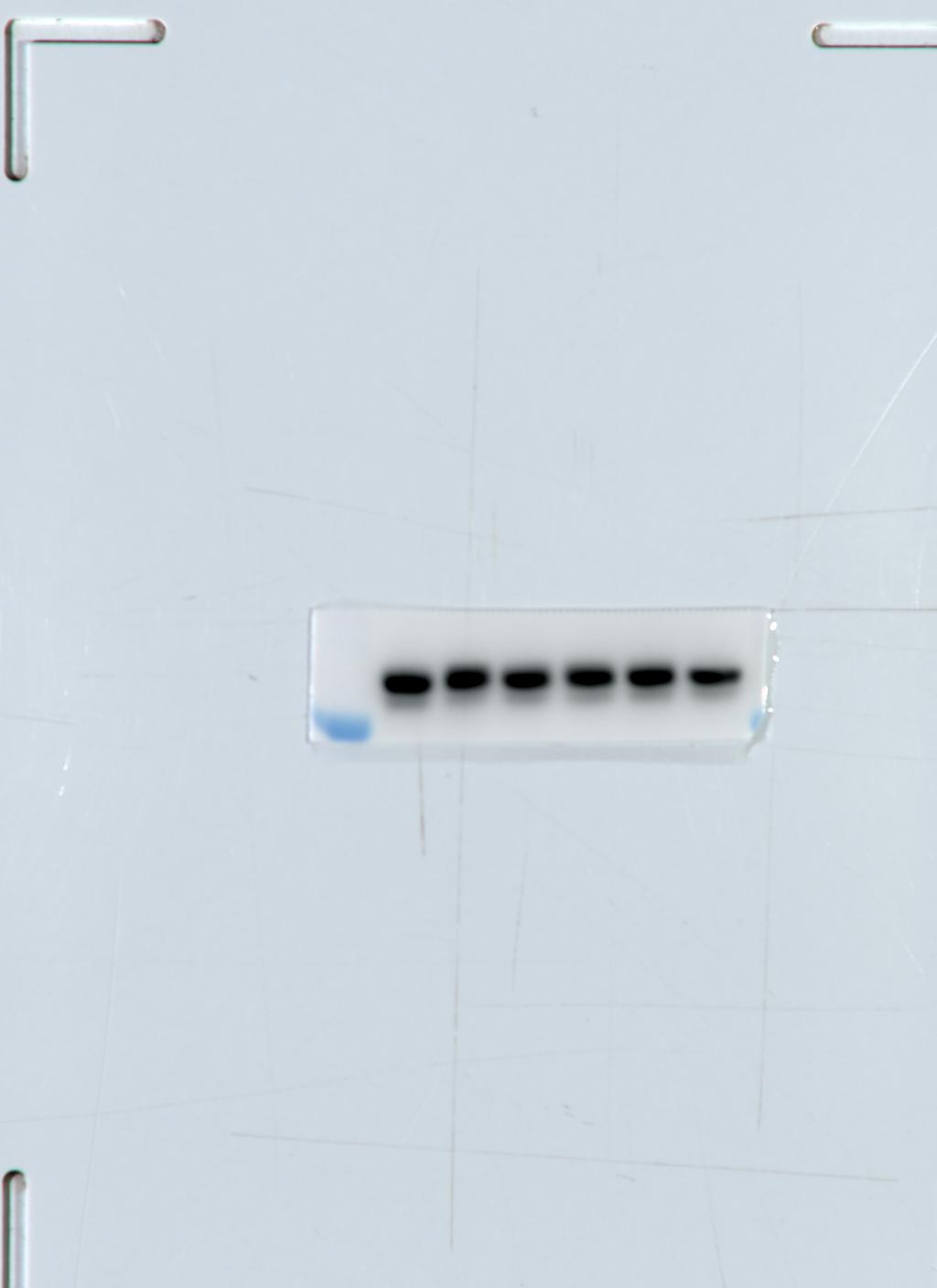

Supplement: Figure 6—source data 4. [file elife-76183-fig6-data4.zip › Figure 6-source data 4/Figure 6D INPUT-AKT.jpg]

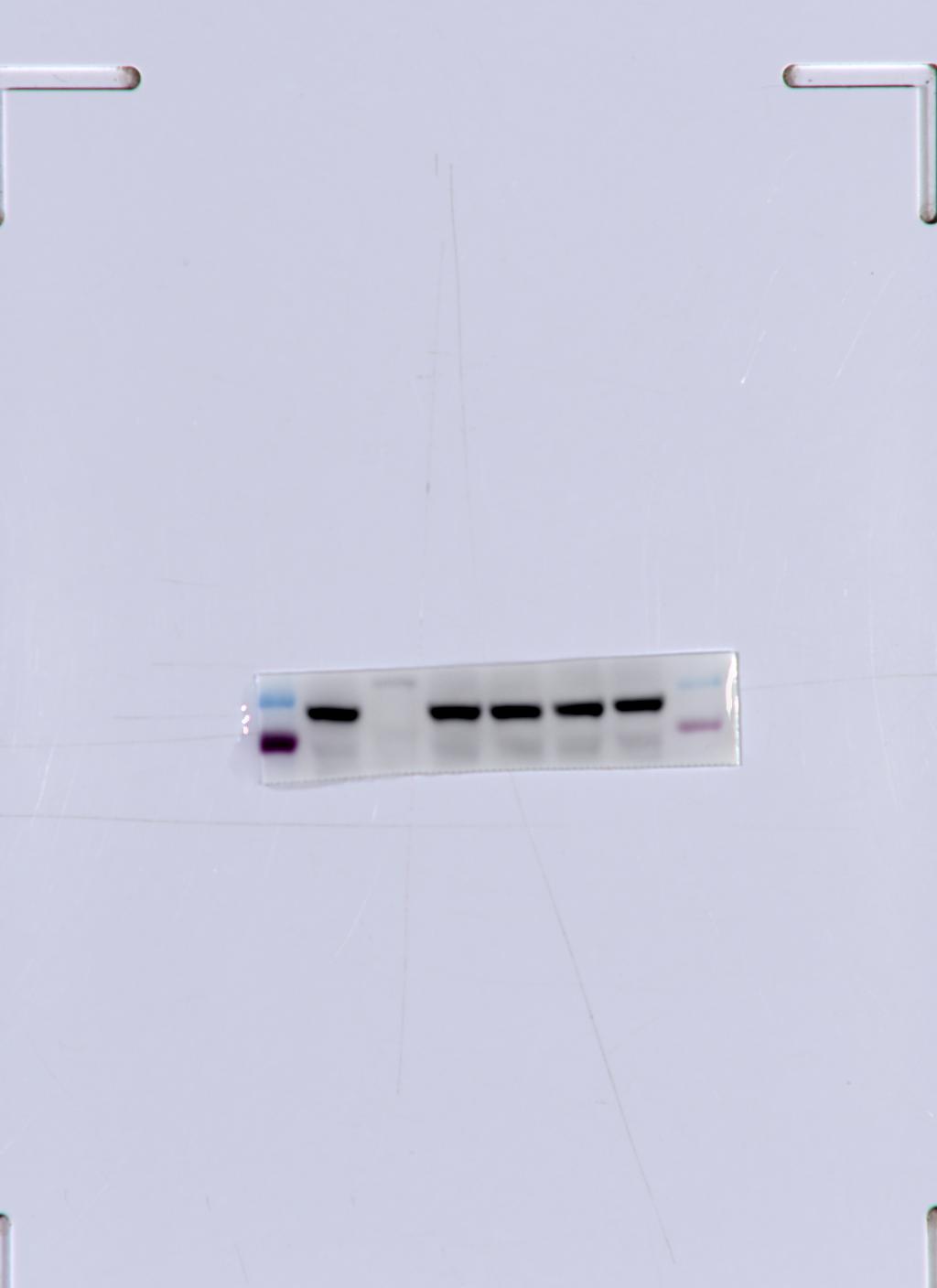

Supplement: Figure 6—source data 4. [file elife-76183-fig6-data4.zip › Figure 6-source data 4/Figure 6D INPUT-FER.jpg]

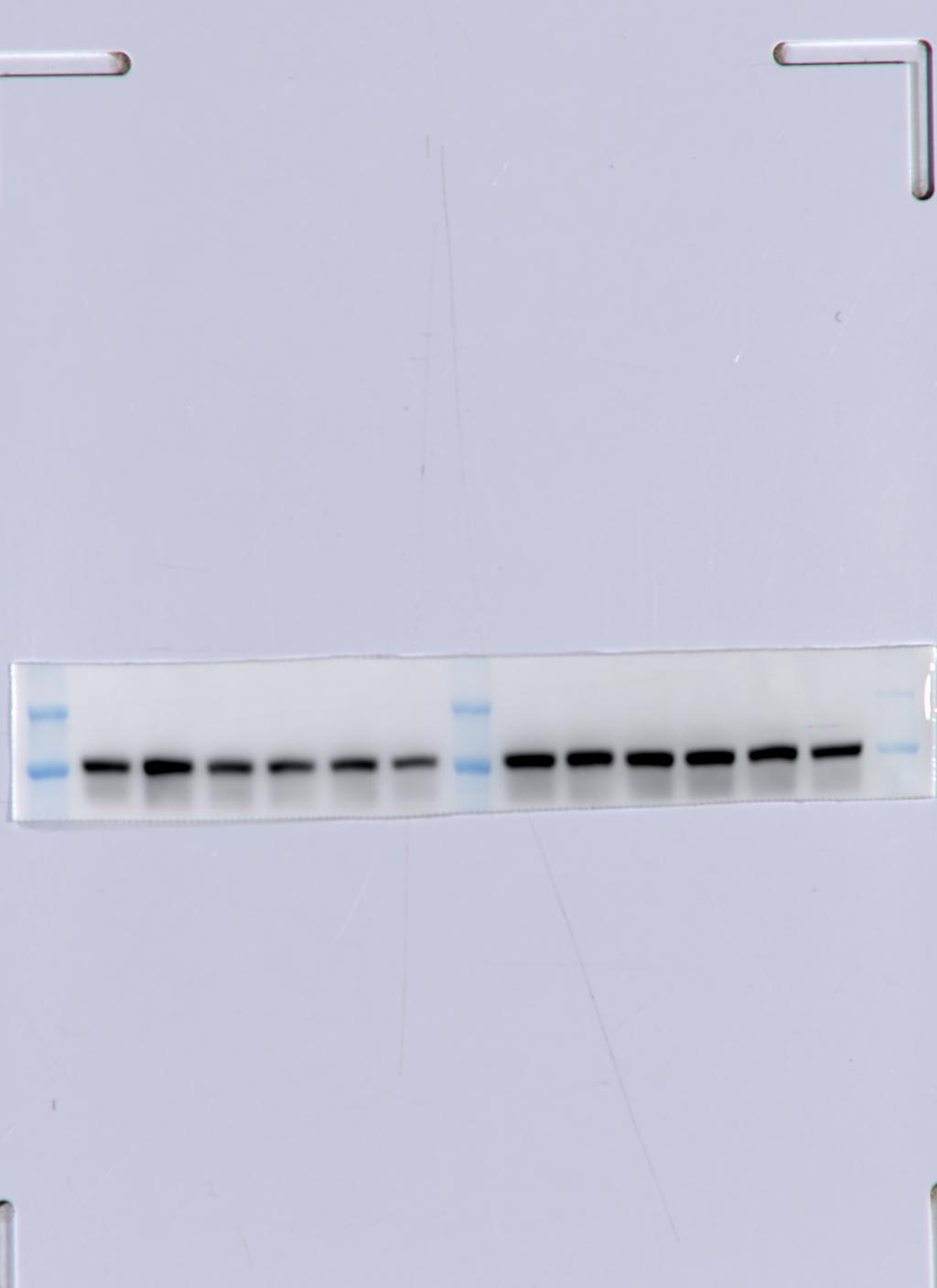

Supplement: Figure 6—source data 4. [file elife-76183-fig6-data4.zip › Figure 6-source data 4/Figure 6D INPUT-IRS4.jpg]

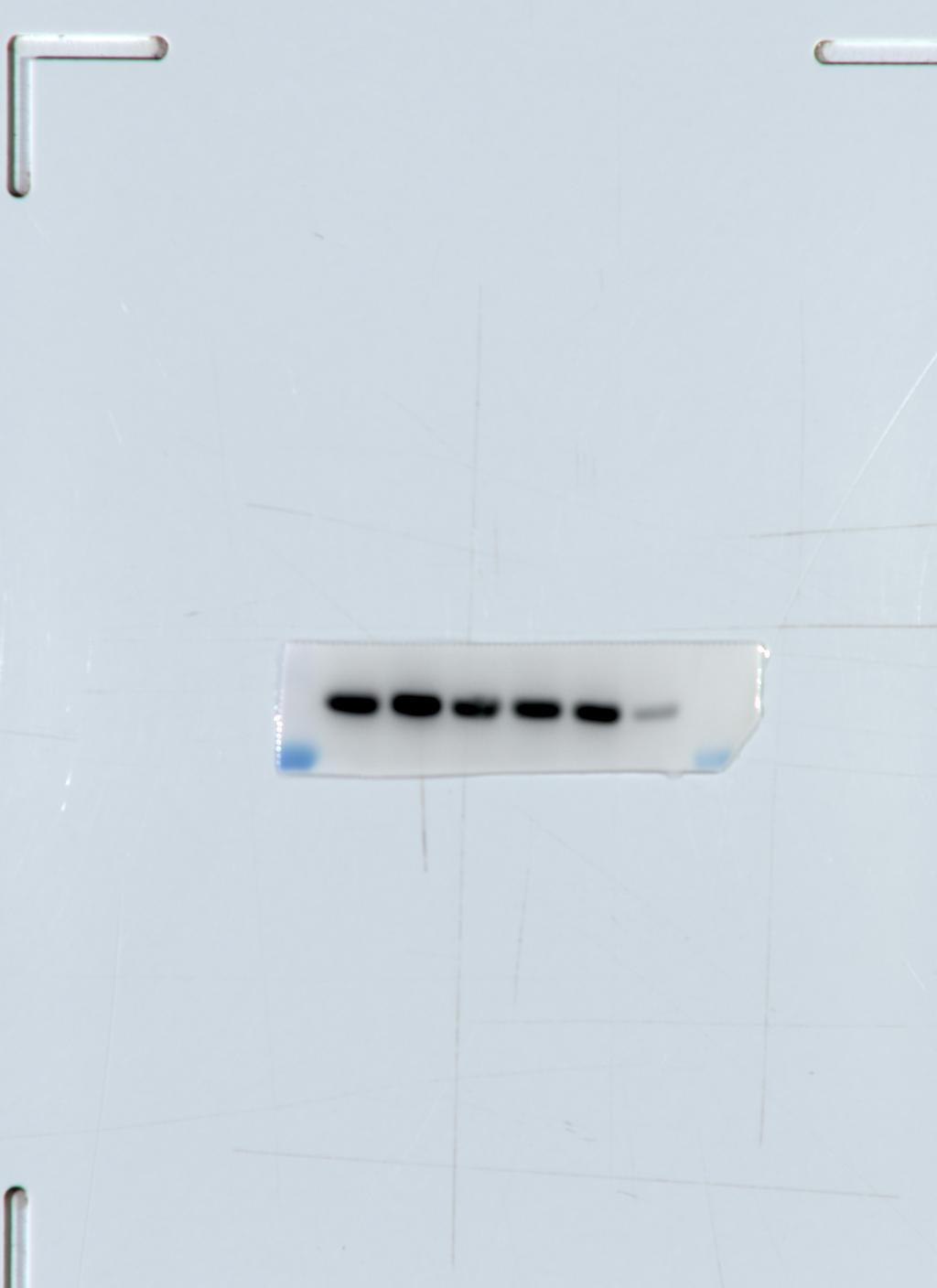

Supplement: Figure 6—source data 4. [file elife-76183-fig6-data4.zip › Figure 6-source data 4/Figure 6D INPUT-pS473 AKT.jpg]

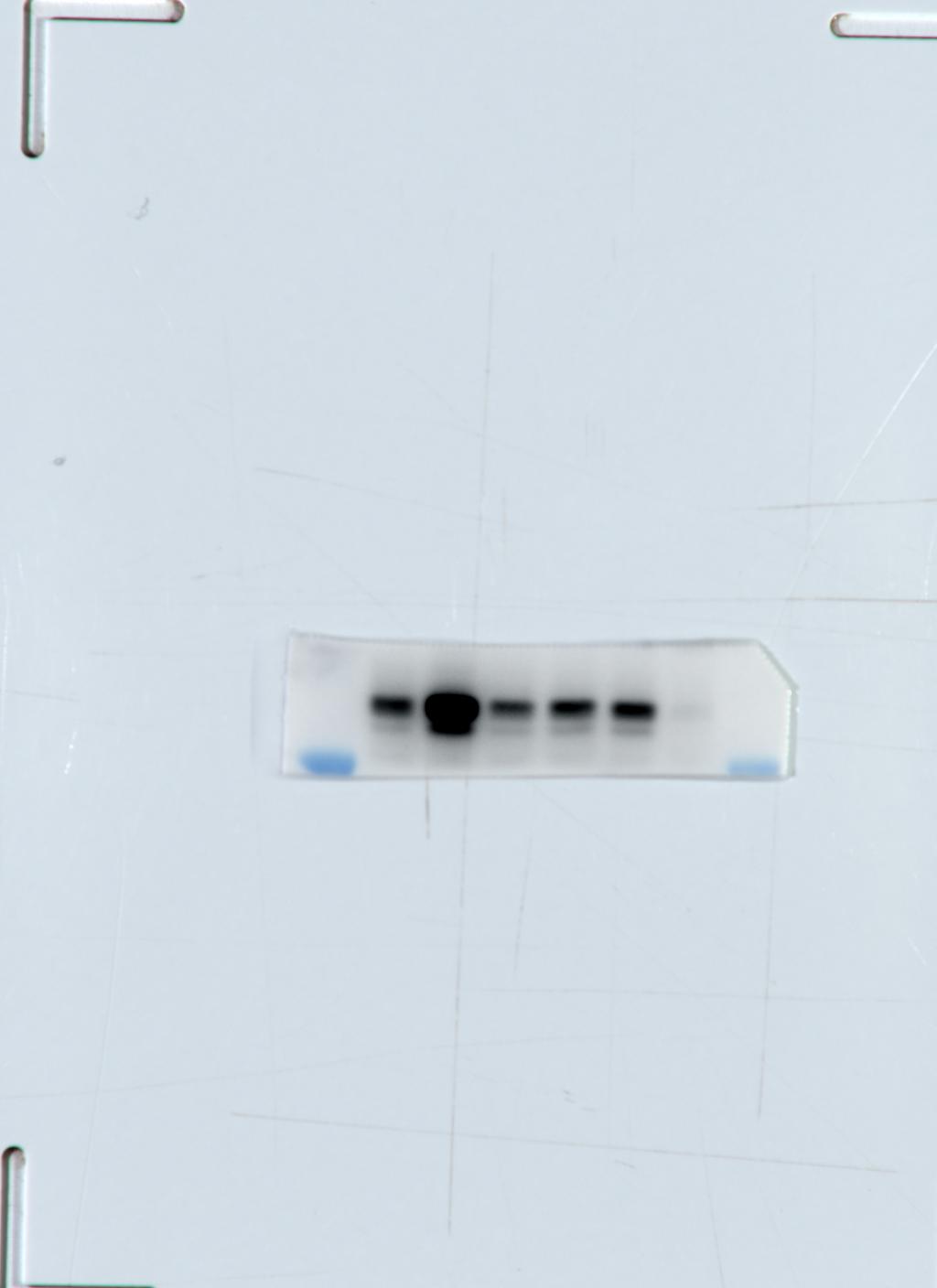

Supplement: Figure 6—source data 4. [file elife-76183-fig6-data4.zip › Figure 6-source data 4/Figure 6D INPUT-pT308 AKT.jpg]

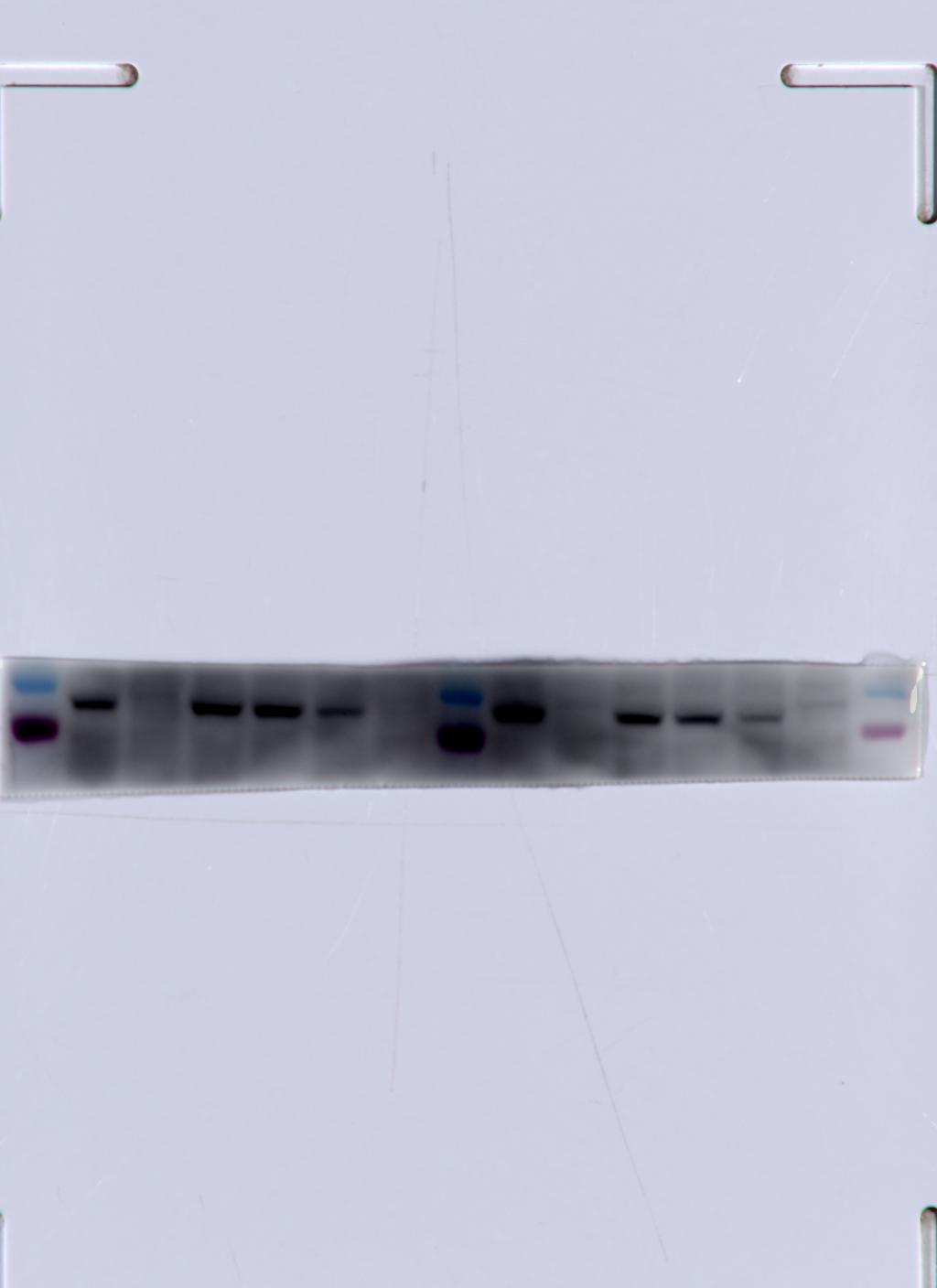

Supplement: Figure 6—source data 4. [file elife-76183-fig6-data4.zip › Figure 6-source data 4/Figure 6D INPUT-pY402 FER.jpg]

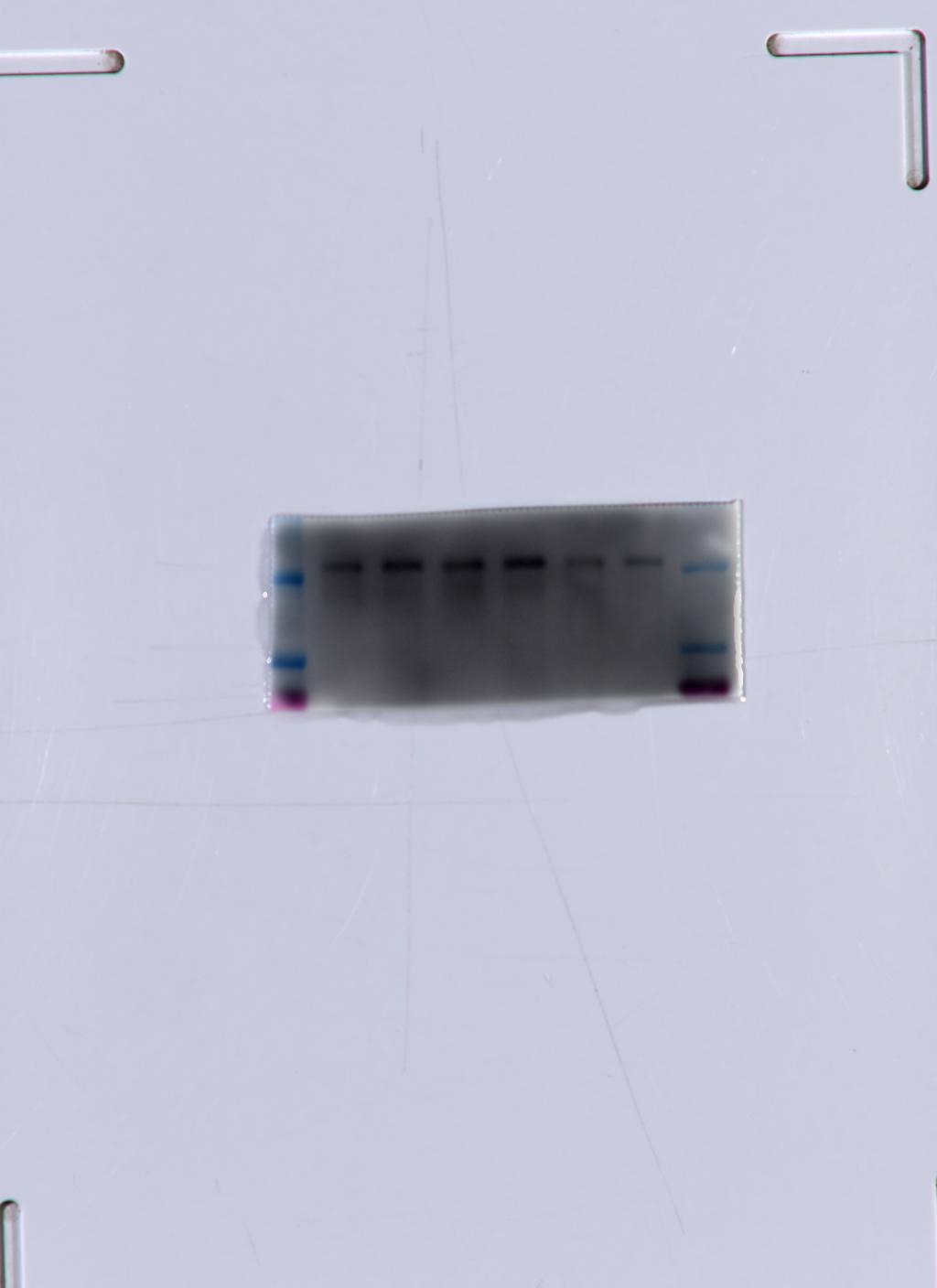

Supplement: Figure 6—source data 4. [file elife-76183-fig6-data4.zip › Figure 6-source data 4/Figure 6D IP-4G10.jpg]

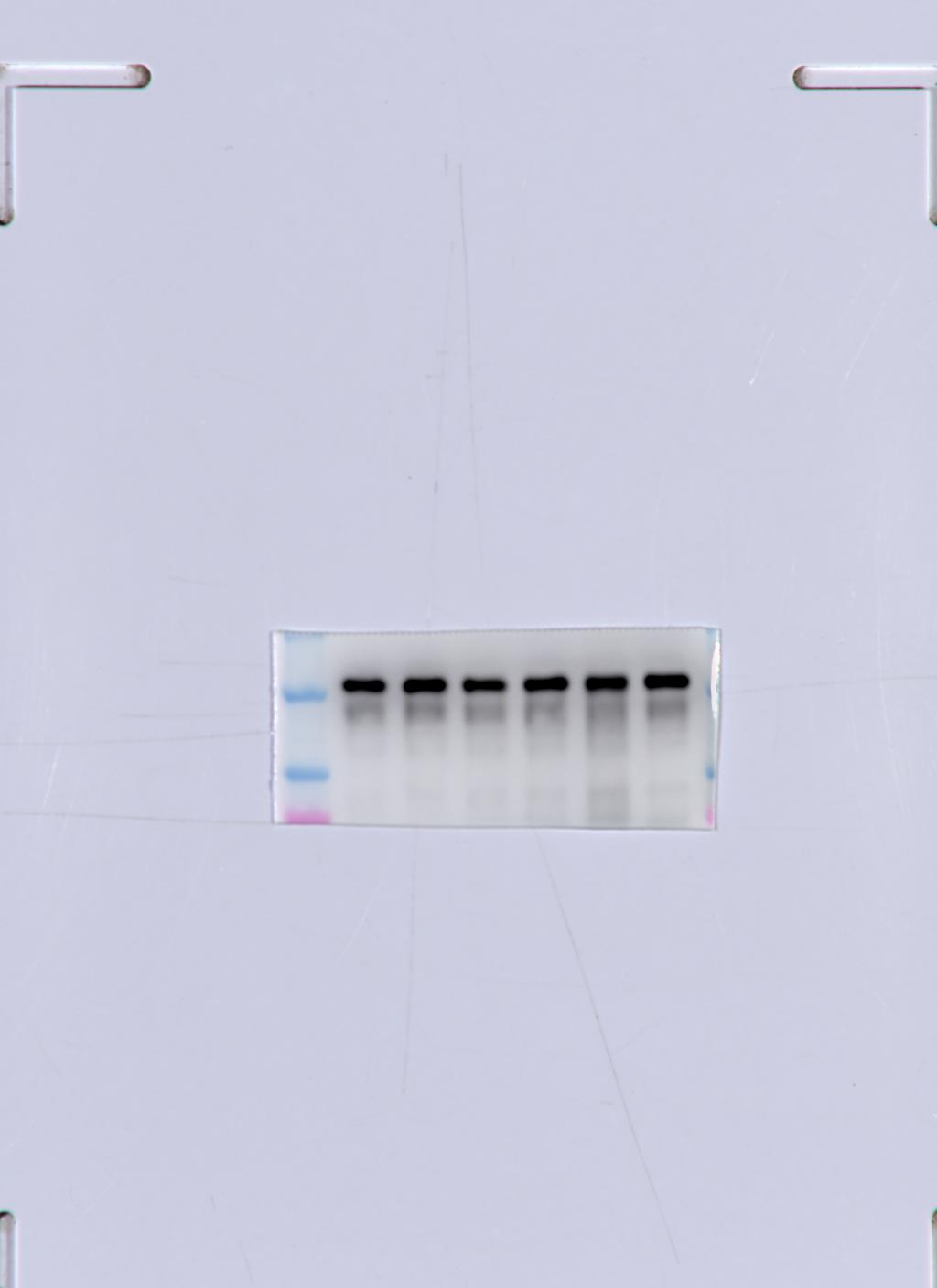

Supplement: Figure 6—source data 4. [file elife-76183-fig6-data4.zip › Figure 6-source data 4/Figure 6D IP-IRS4.jpg]

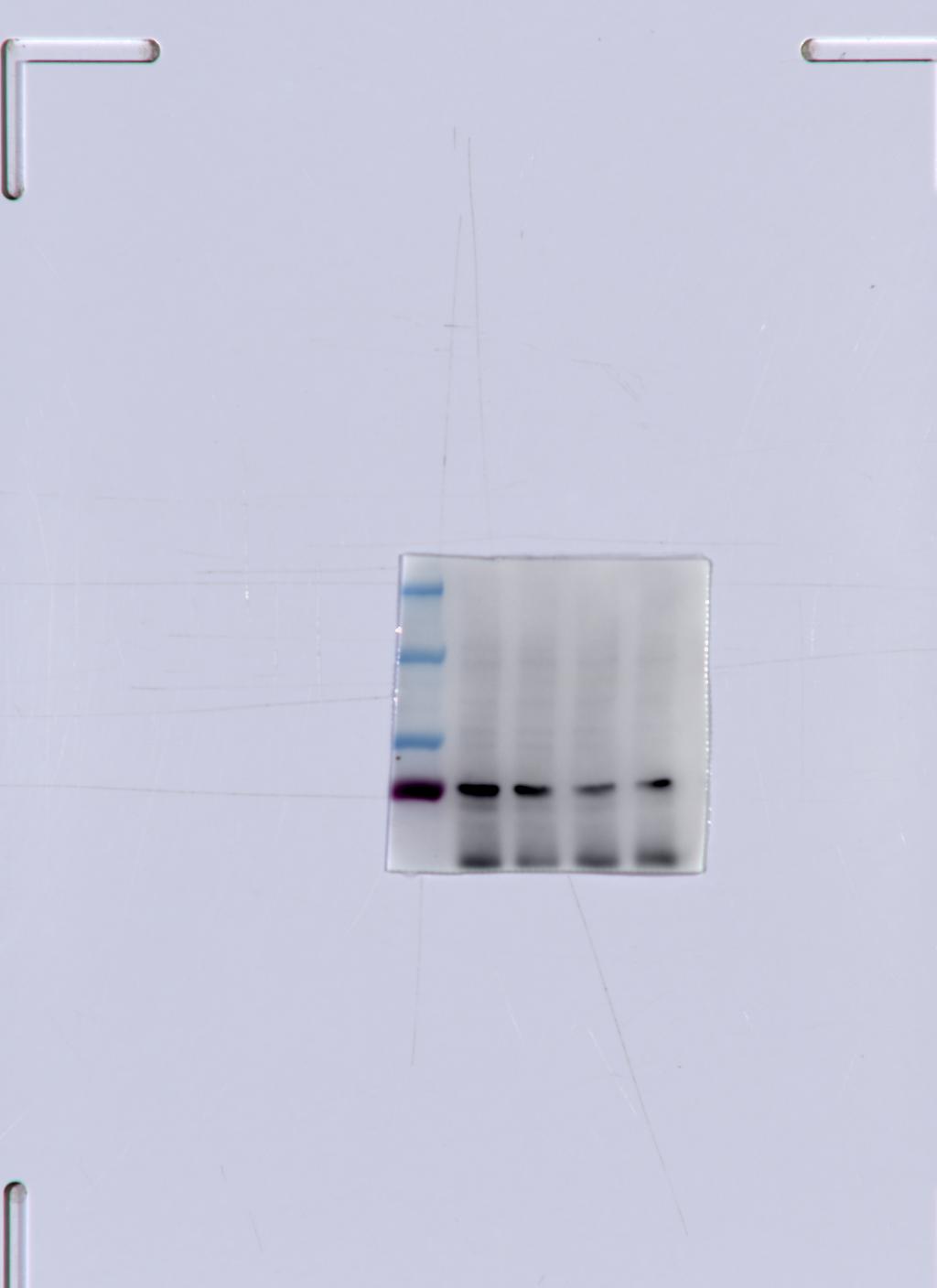

Supplement: Figure 6—source data 4. [file elife-76183-fig6-data4.zip › Figure 6-source data 4/Figure 6D IP-PIK3R2.jpg]

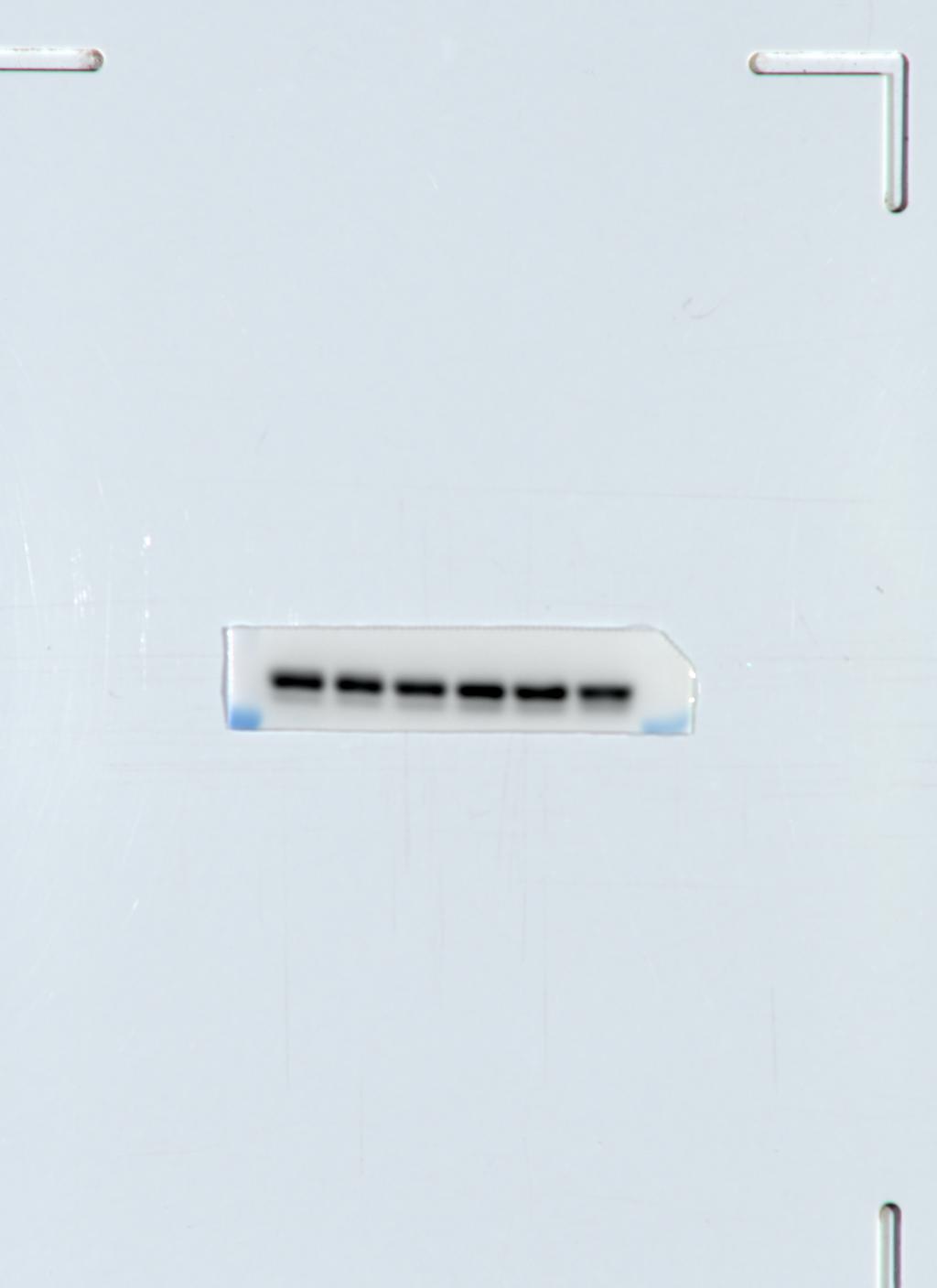

Supplement: Figure 6—figure supplement 1—source data 1. [file elife-76183-fig6-figsupp1-data1.zip › Figure 6-figure supplement 1-source data 1/Figure 6 S1A INPUT-AKT.jpg]

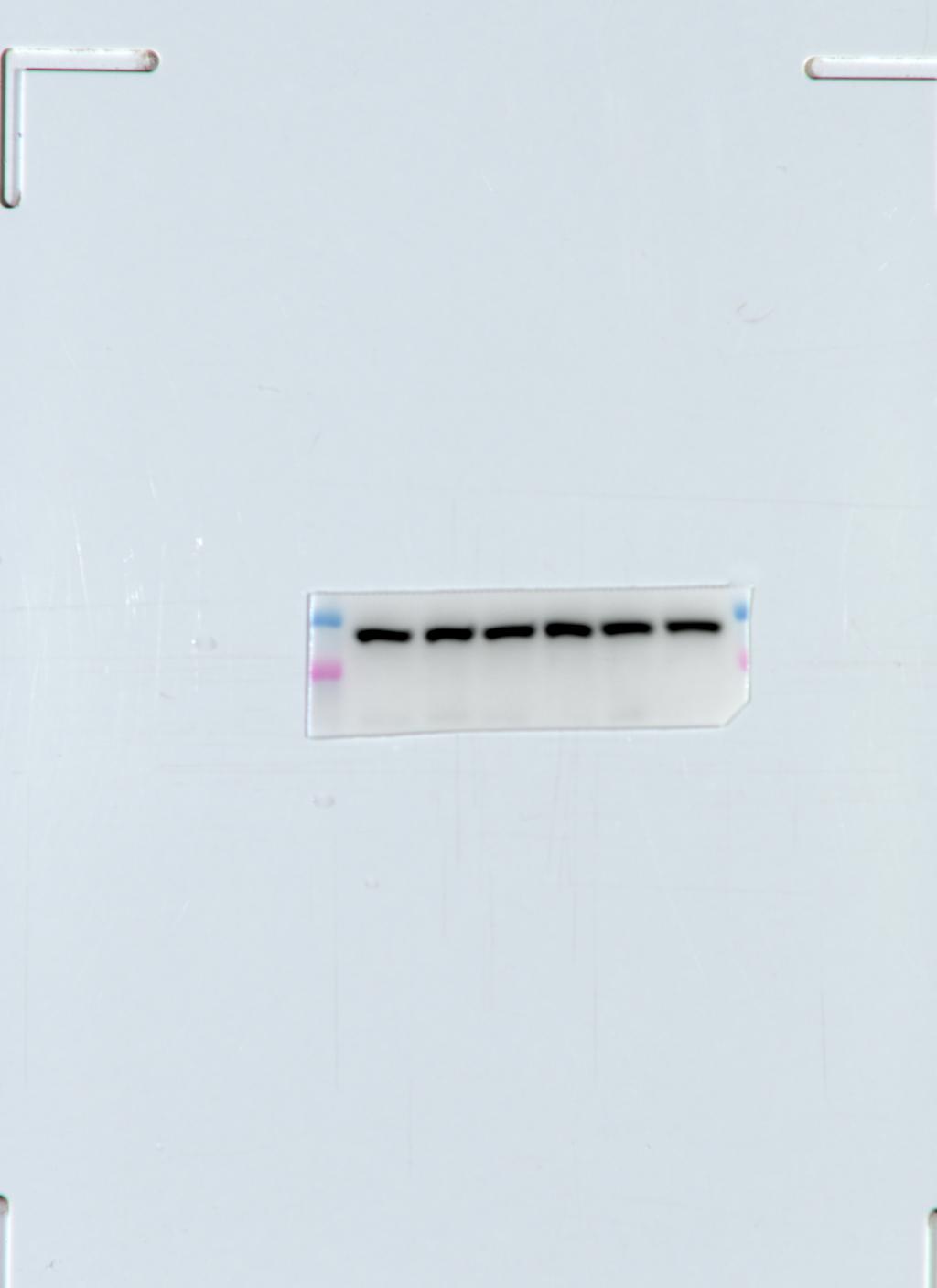

Supplement: Figure 6—figure supplement 1—source data 1. [file elife-76183-fig6-figsupp1-data1.zip › Figure 6-figure supplement 1-source data 1/Figure 6 S1A INPUT-FER.jpg]

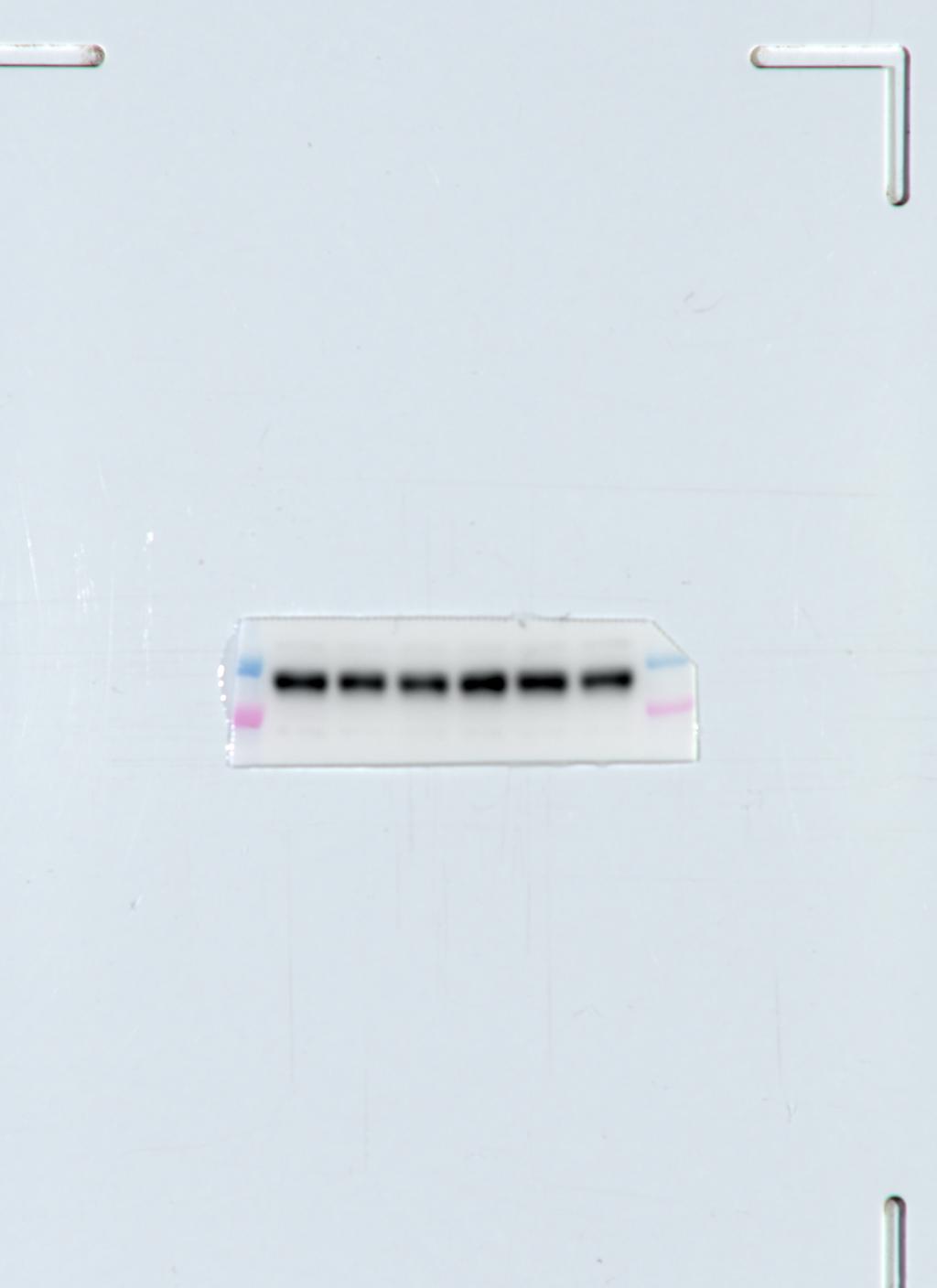

Supplement: Figure 6—figure supplement 1—source data 1. [file elife-76183-fig6-figsupp1-data1.zip › Figure 6-figure supplement 1-source data 1/Figure 6 S1A INPUT-IGF1R.jpg]

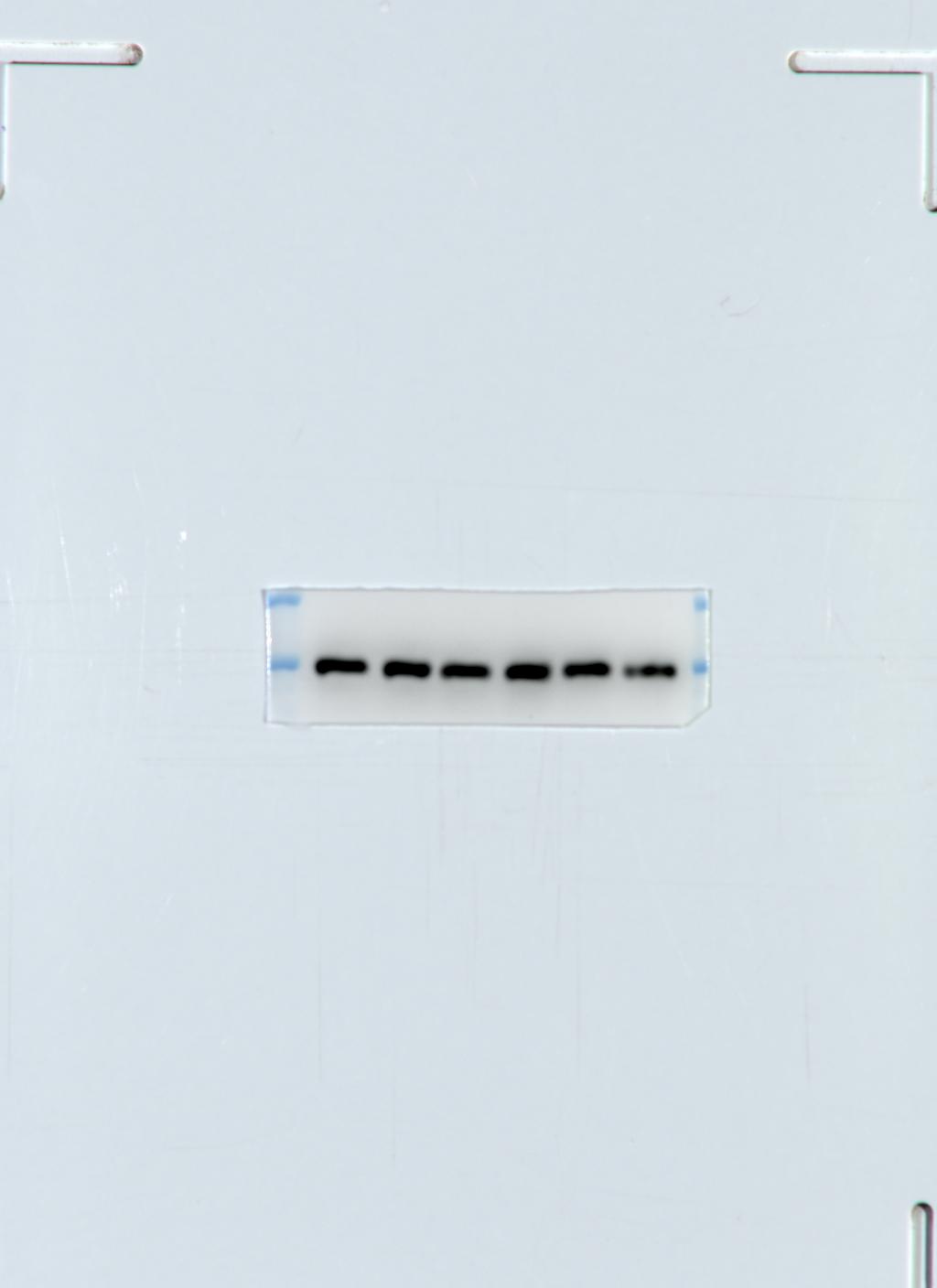

Supplement: Figure 6—figure supplement 1—source data 1. [file elife-76183-fig6-figsupp1-data1.zip › Figure 6-figure supplement 1-source data 1/Figure 6 S1A INPUT-IRS4.jpg]

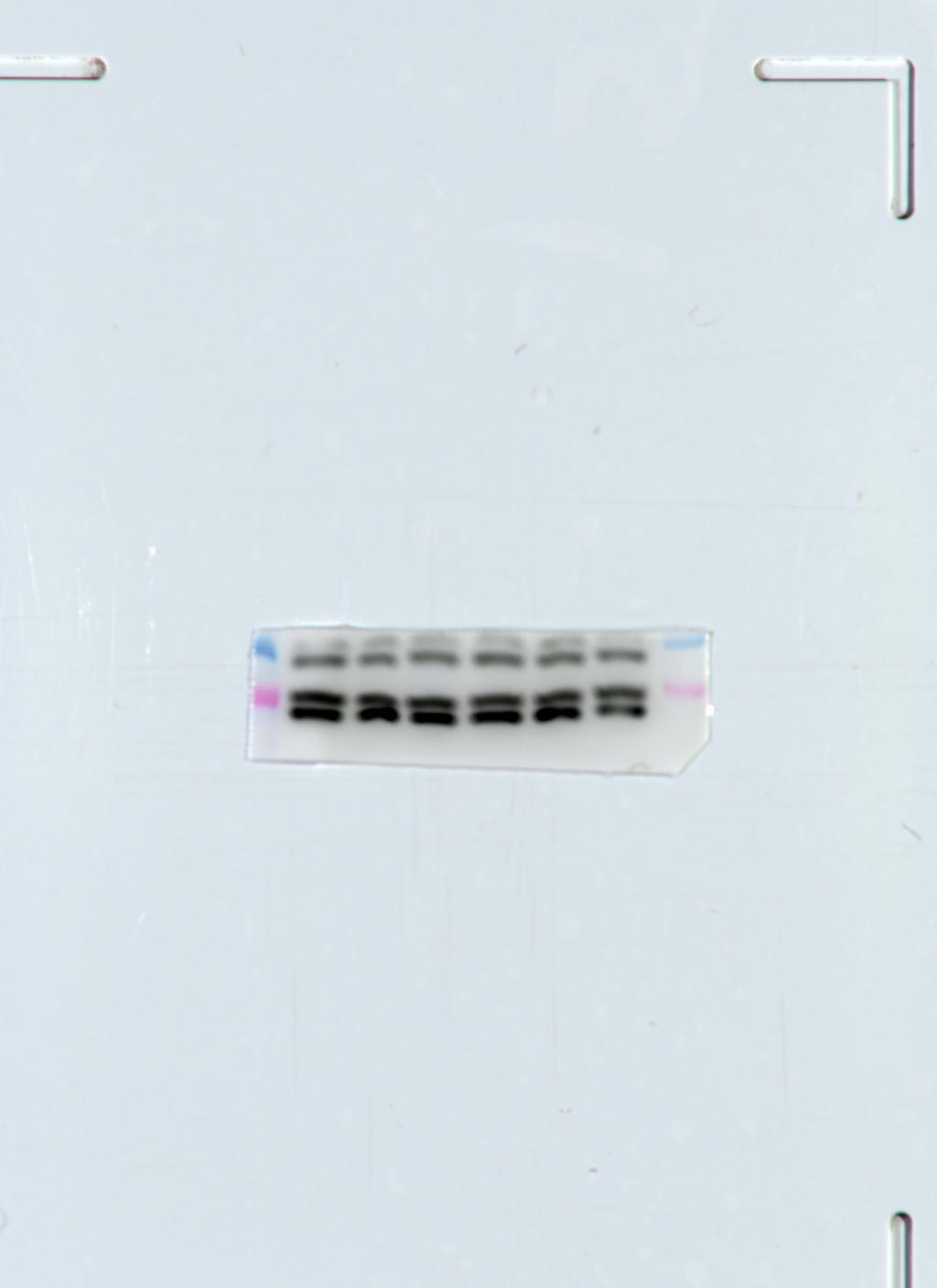

Supplement: Figure 6—figure supplement 1—source data 1. [file elife-76183-fig6-figsupp1-data1.zip › Figure 6-figure supplement 1-source data 1/Figure 6 S1A INPUT-PIK3R2.jpg]

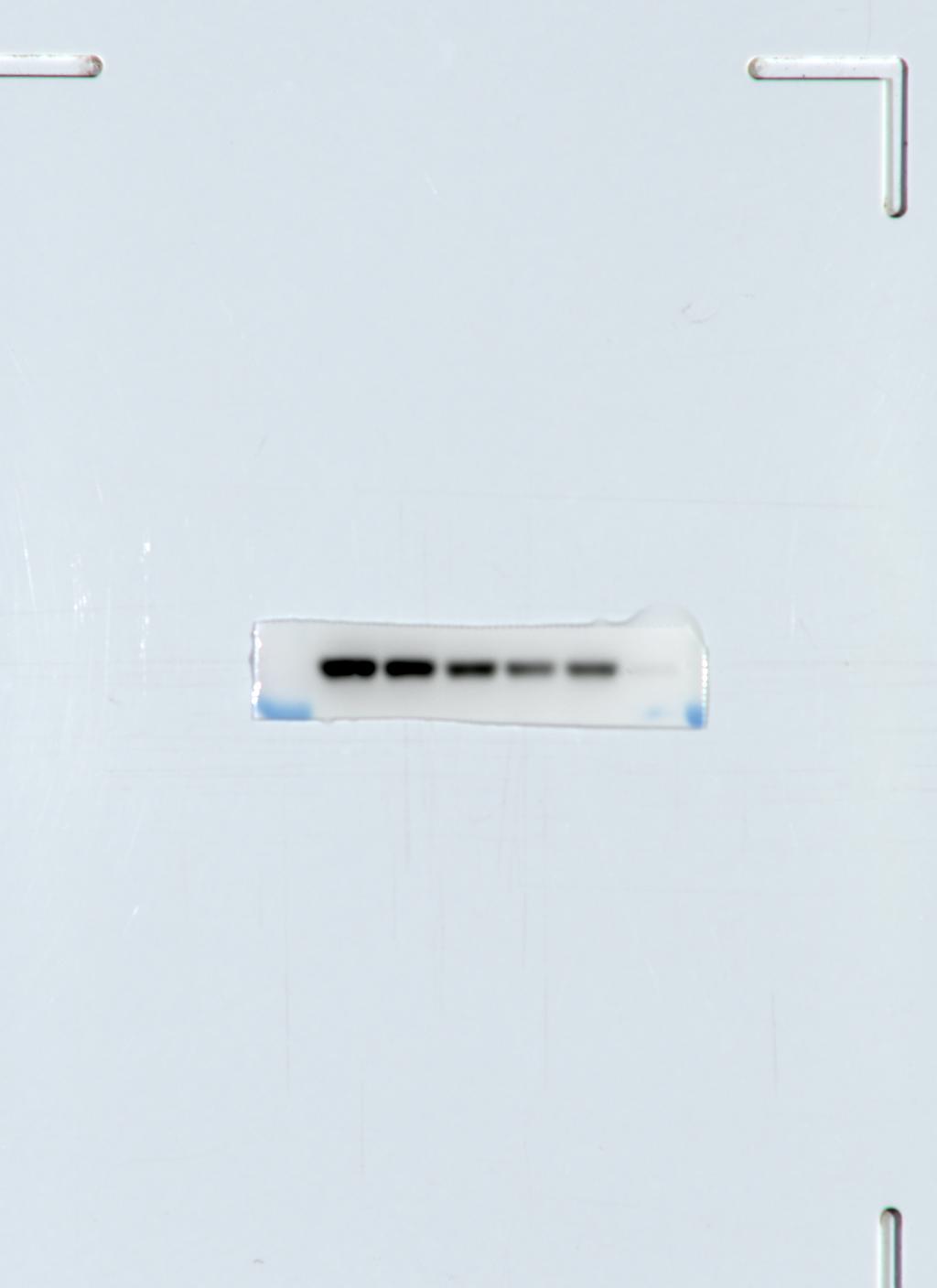

Supplement: Figure 6—figure supplement 1—source data 1. [file elife-76183-fig6-figsupp1-data1.zip › Figure 6-figure supplement 1-source data 1/Figure 6 S1A INPUT-pS473 AKT.jpg]

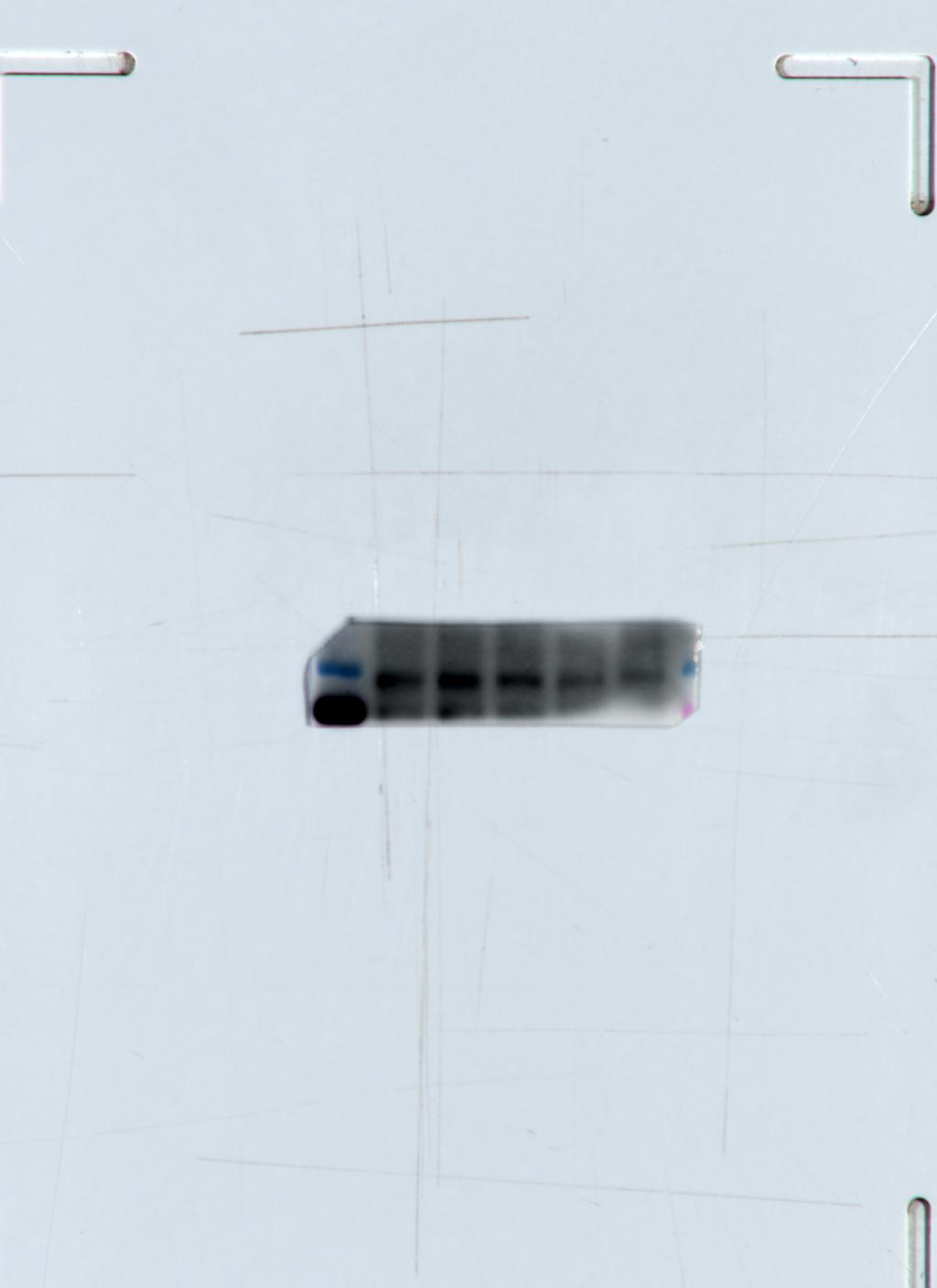

Supplement: Figure 6—figure supplement 1—source data 1. [file elife-76183-fig6-figsupp1-data1.zip › Figure 6-figure supplement 1-source data 1/Figure 6 S1A INPUT-pY1131 IGF1R.jpg]

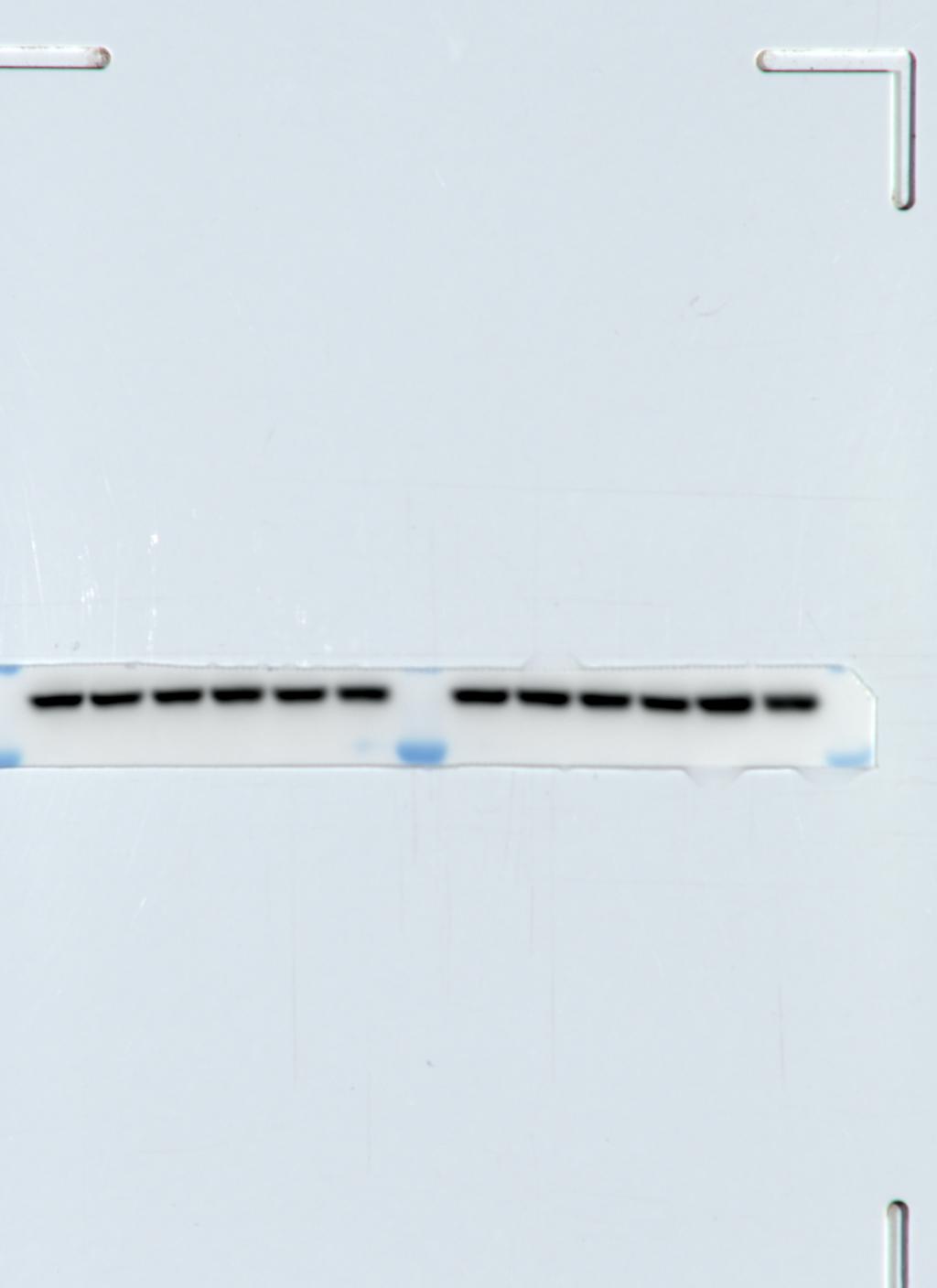

Supplement: Figure 6—figure supplement 1—source data 1. [file elife-76183-fig6-figsupp1-data1.zip › Figure 6-figure supplement 1-source data 1/Figure 6 S1A INPUT-Tubulin.jpg]

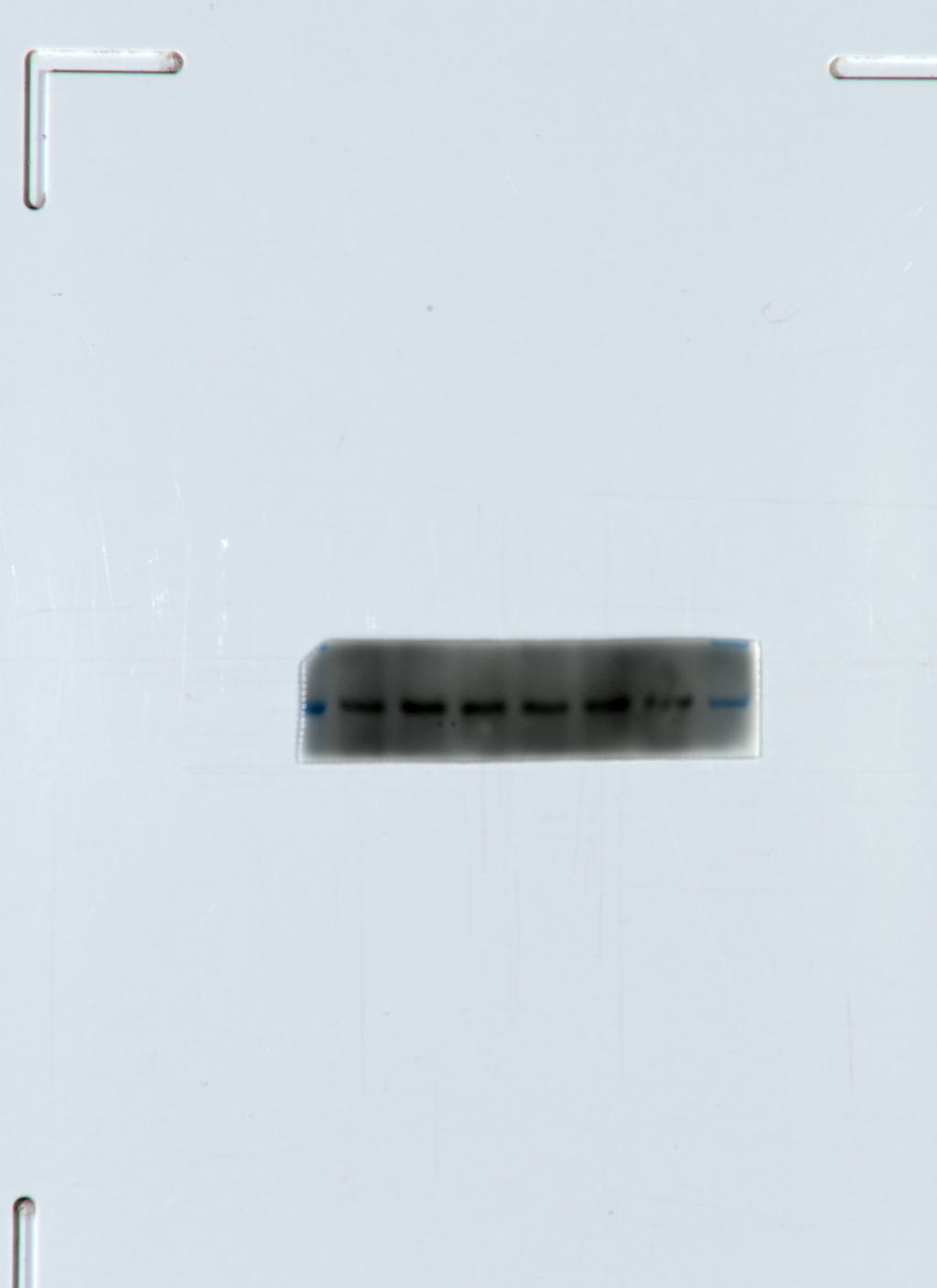

Supplement: Figure 6—figure supplement 1—source data 1. [file elife-76183-fig6-figsupp1-data1.zip › Figure 6-figure supplement 1-source data 1/Figure 6 S1A IP-4G10.jpg]

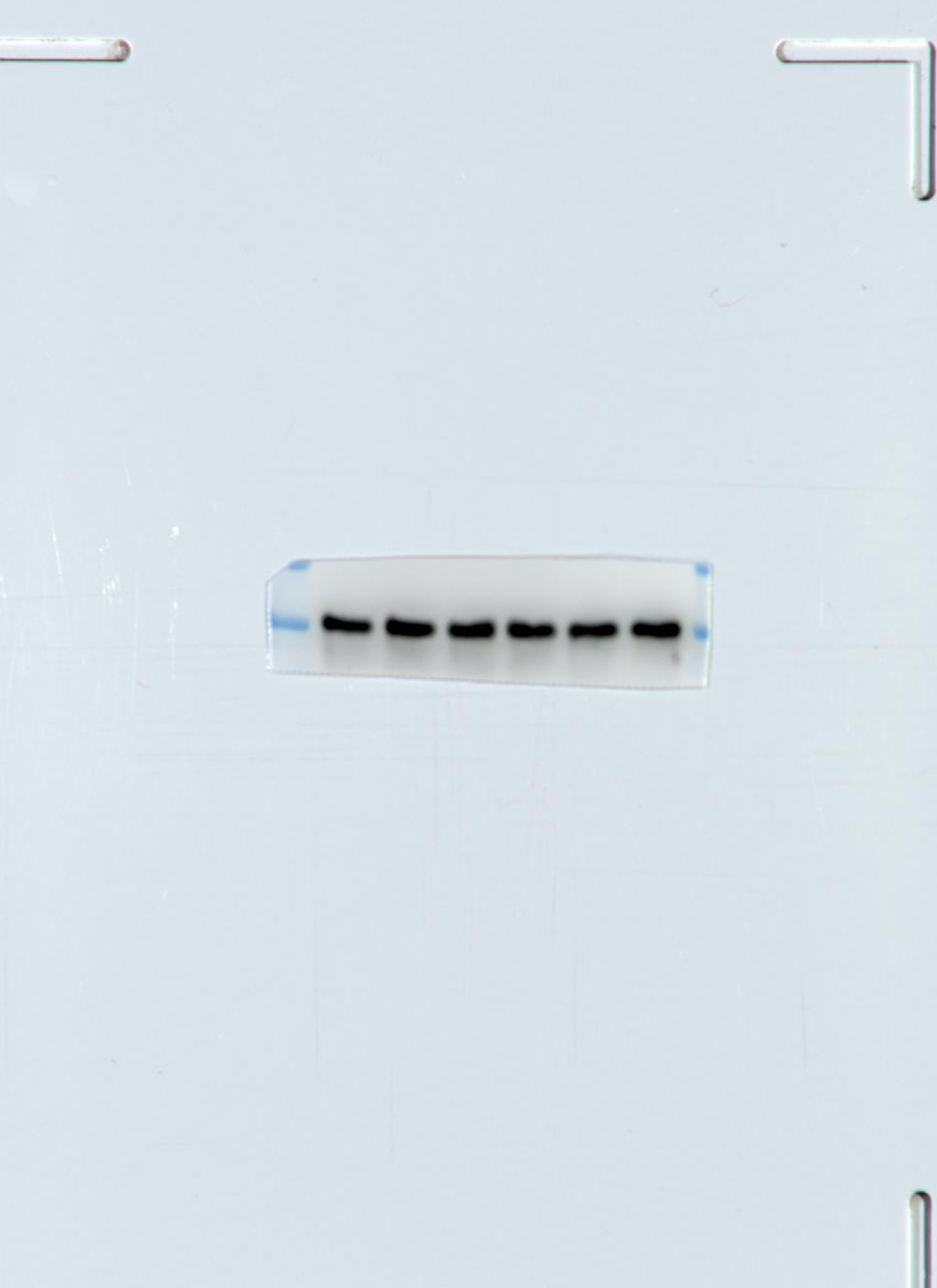

Supplement: Figure 6—figure supplement 1—source data 1. [file elife-76183-fig6-figsupp1-data1.zip › Figure 6-figure supplement 1-source data 1/Figure 6 S1A IP-IRS4.jpg]

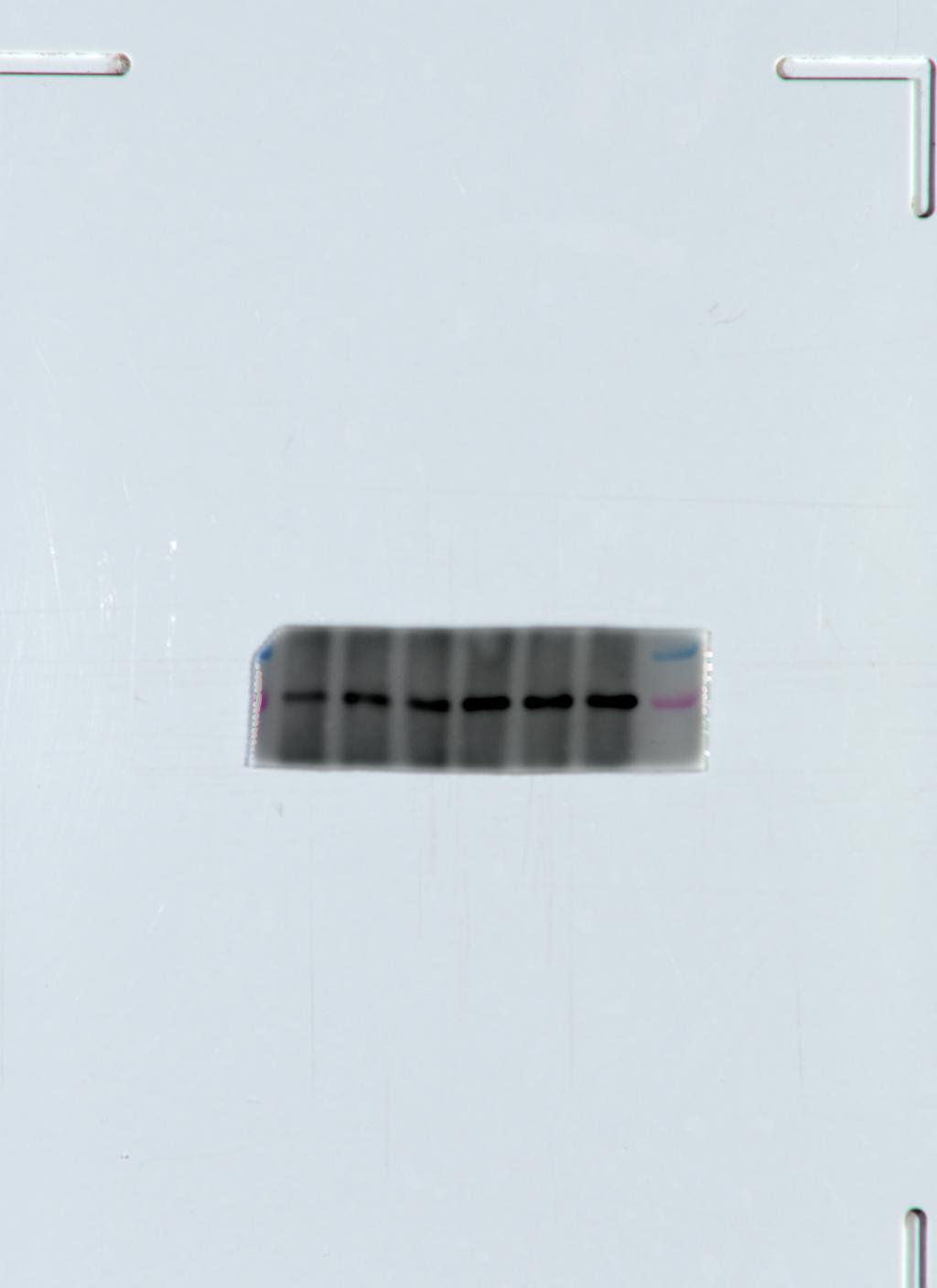

Supplement: Figure 6—figure supplement 1—source data 1. [file elife-76183-fig6-figsupp1-data1.zip › Figure 6-figure supplement 1-source data 1/Figure 6 S1A IP-PIK3R2.jpg]

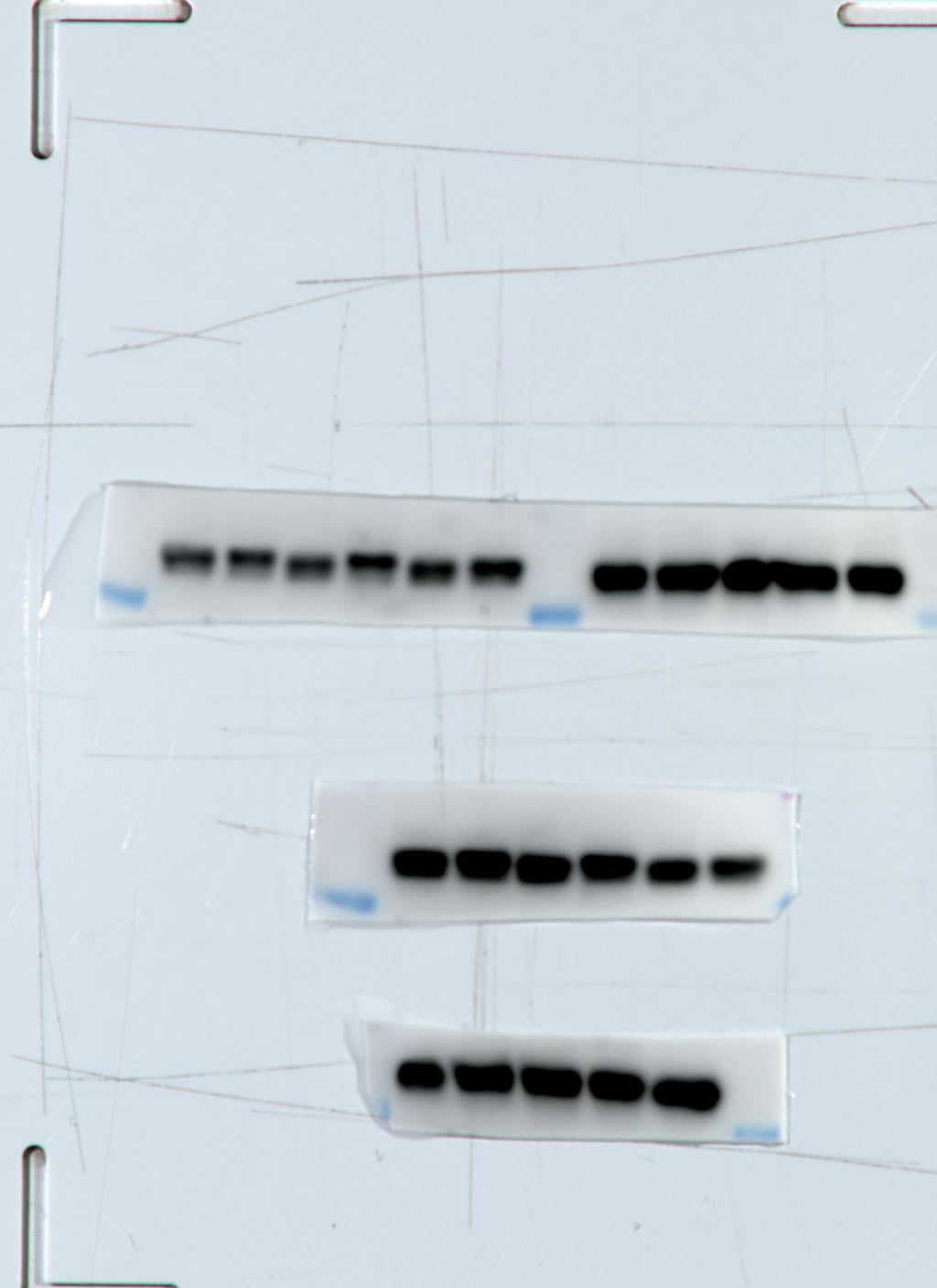

Supplement: Figure 6—figure supplement 1—source data 2. [file elife-76183-fig6-figsupp1-data2.zip › Figure 6-figure supplement 1-source data 2/Figure 6 S1B INPUT-AKT.jpg]

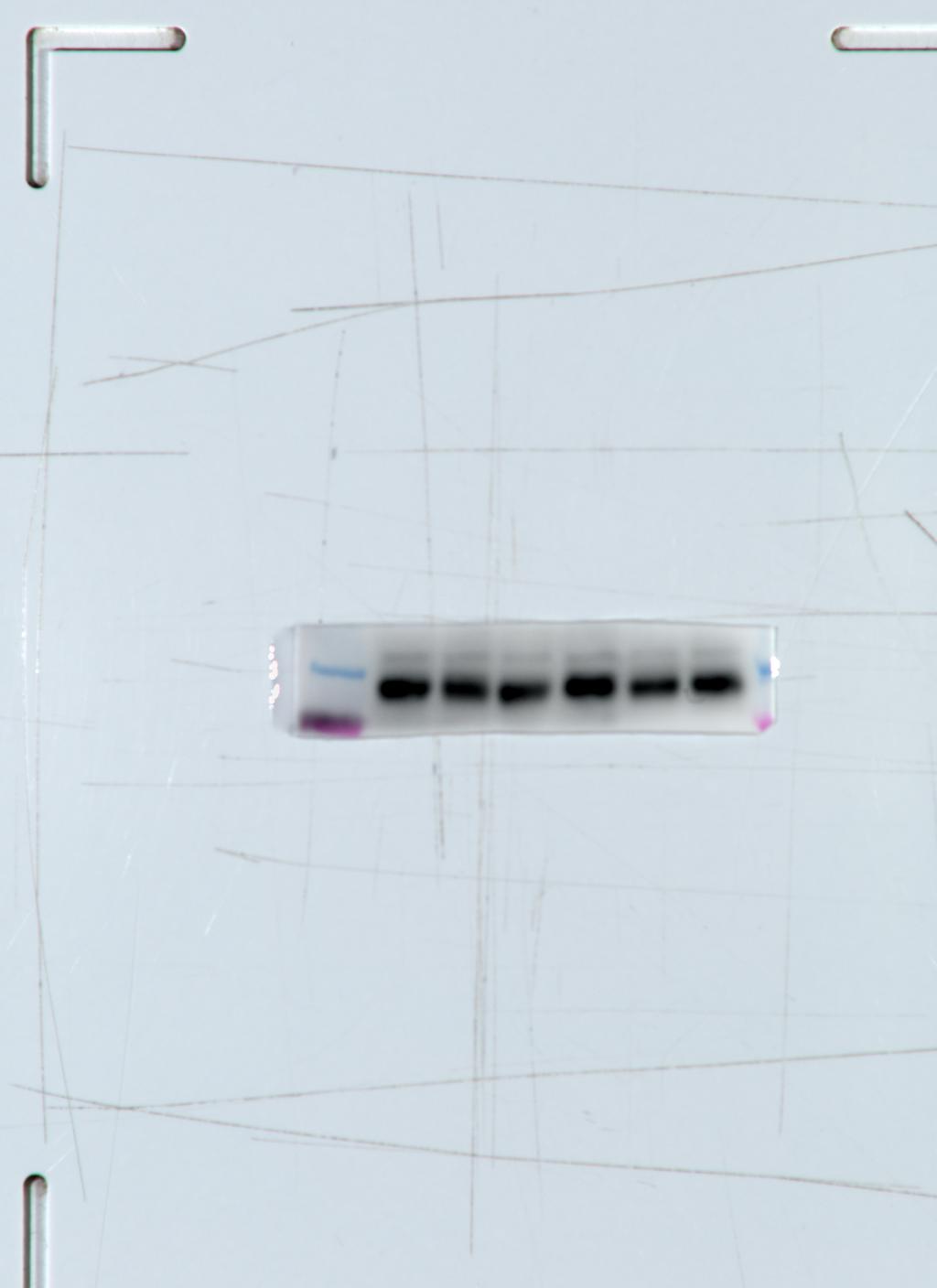

Supplement: Figure 6—figure supplement 1—source data 2. [file elife-76183-fig6-figsupp1-data2.zip › Figure 6-figure supplement 1-source data 2/Figure 6 S1B INPUT-IGF1R.jpg]

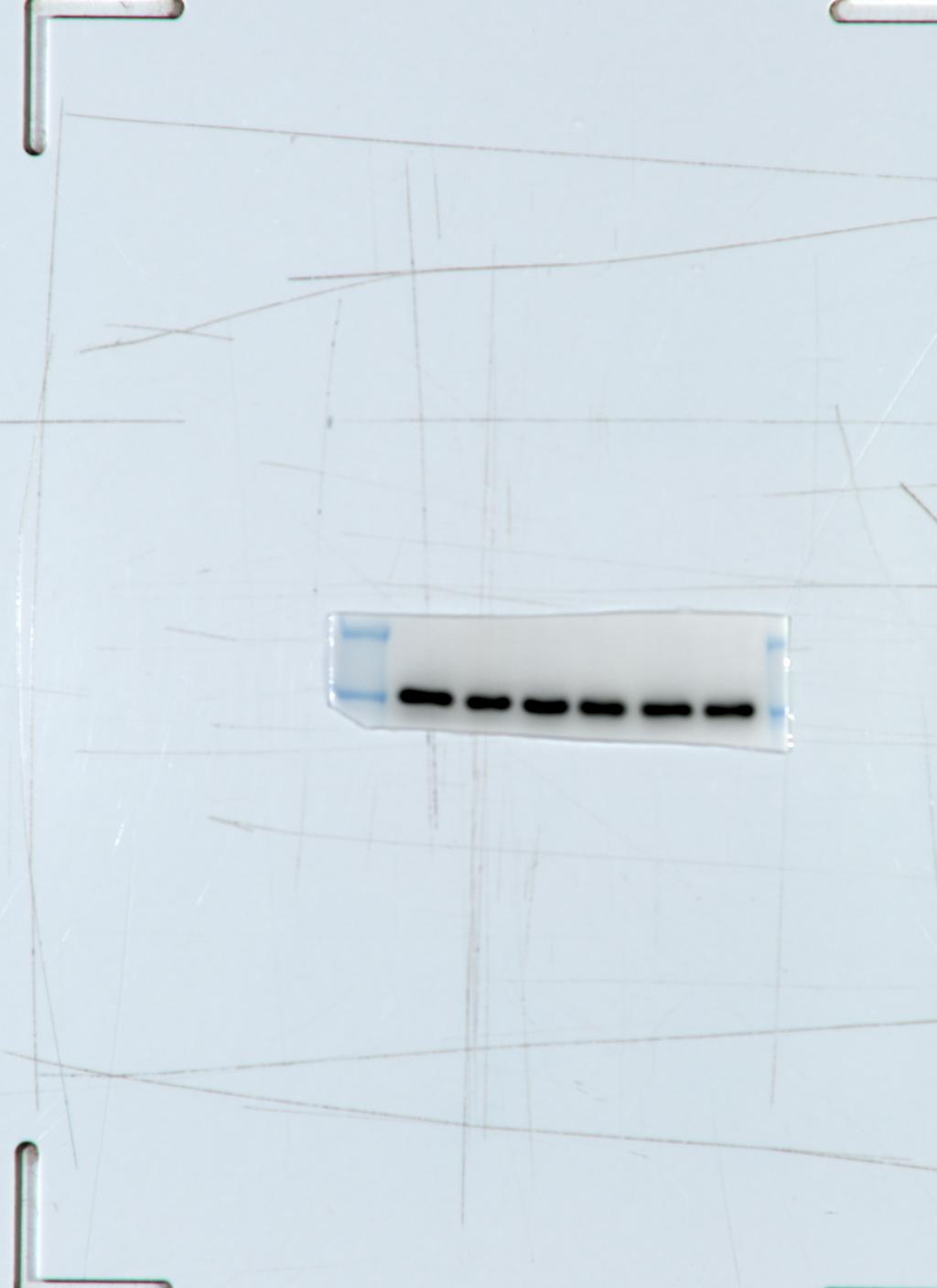

Supplement: Figure 6—figure supplement 1—source data 2. [file elife-76183-fig6-figsupp1-data2.zip › Figure 6-figure supplement 1-source data 2/Figure 6 S1B INPUT-IRS4.jpg]

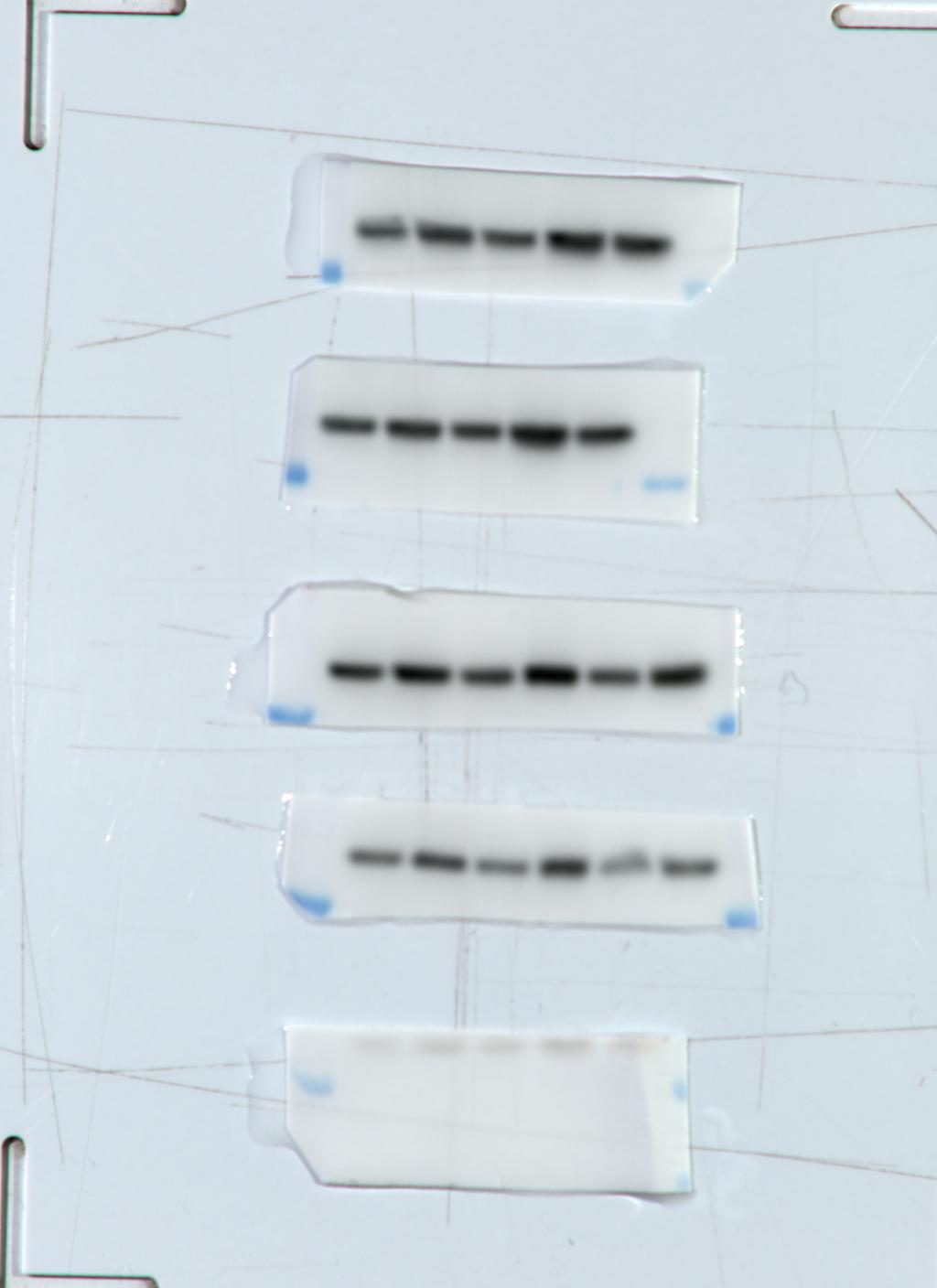

Supplement: Figure 6—figure supplement 1—source data 2. [file elife-76183-fig6-figsupp1-data2.zip › Figure 6-figure supplement 1-source data 2/Figure 6 S1B INPUT-pS473 AKT.jpg]

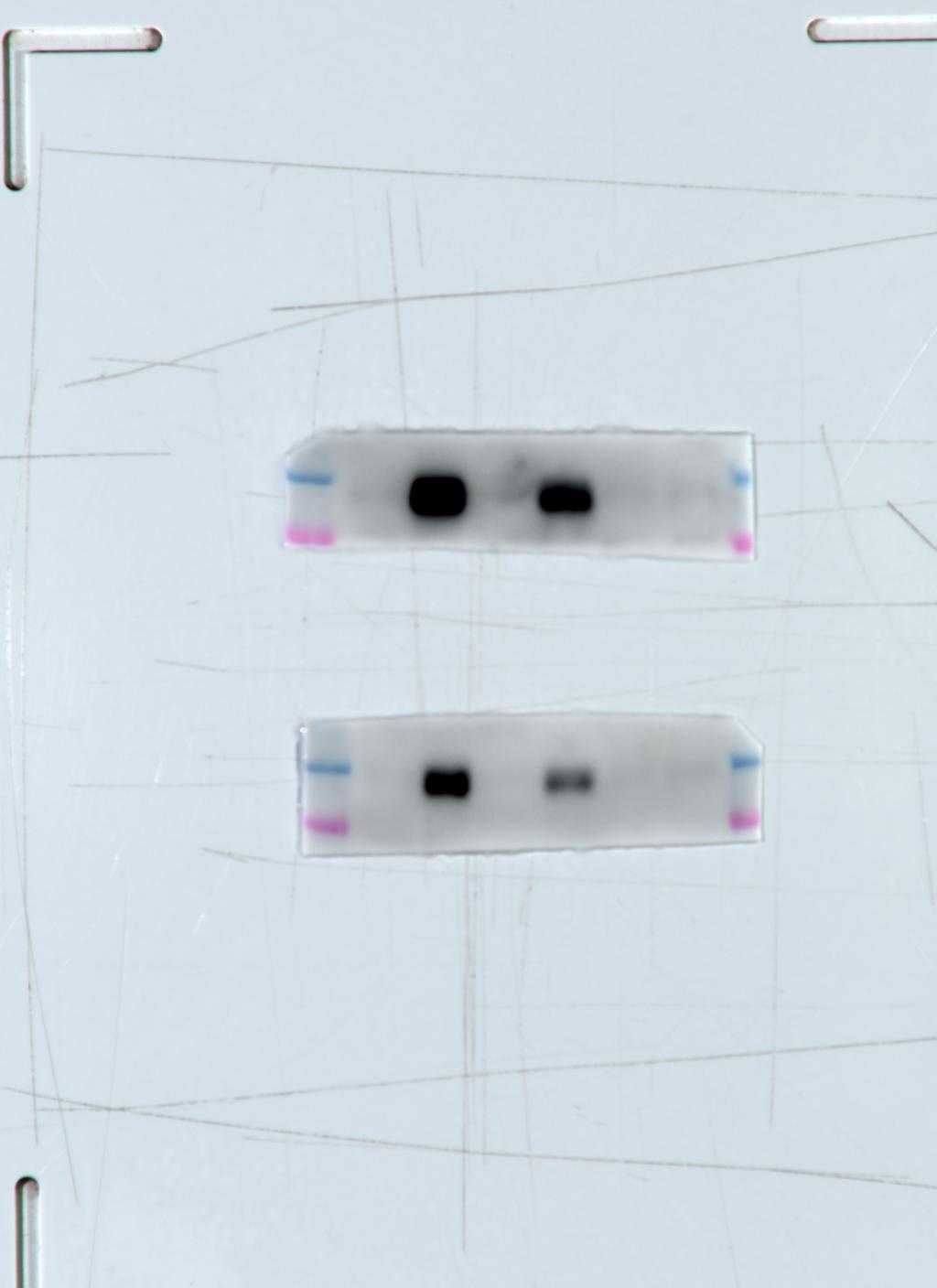

Supplement: Figure 6—figure supplement 1—source data 2. [file elife-76183-fig6-figsupp1-data2.zip › Figure 6-figure supplement 1-source data 2/Figure 6 S1B INPUT-pY1131 IGF1R.jpg]

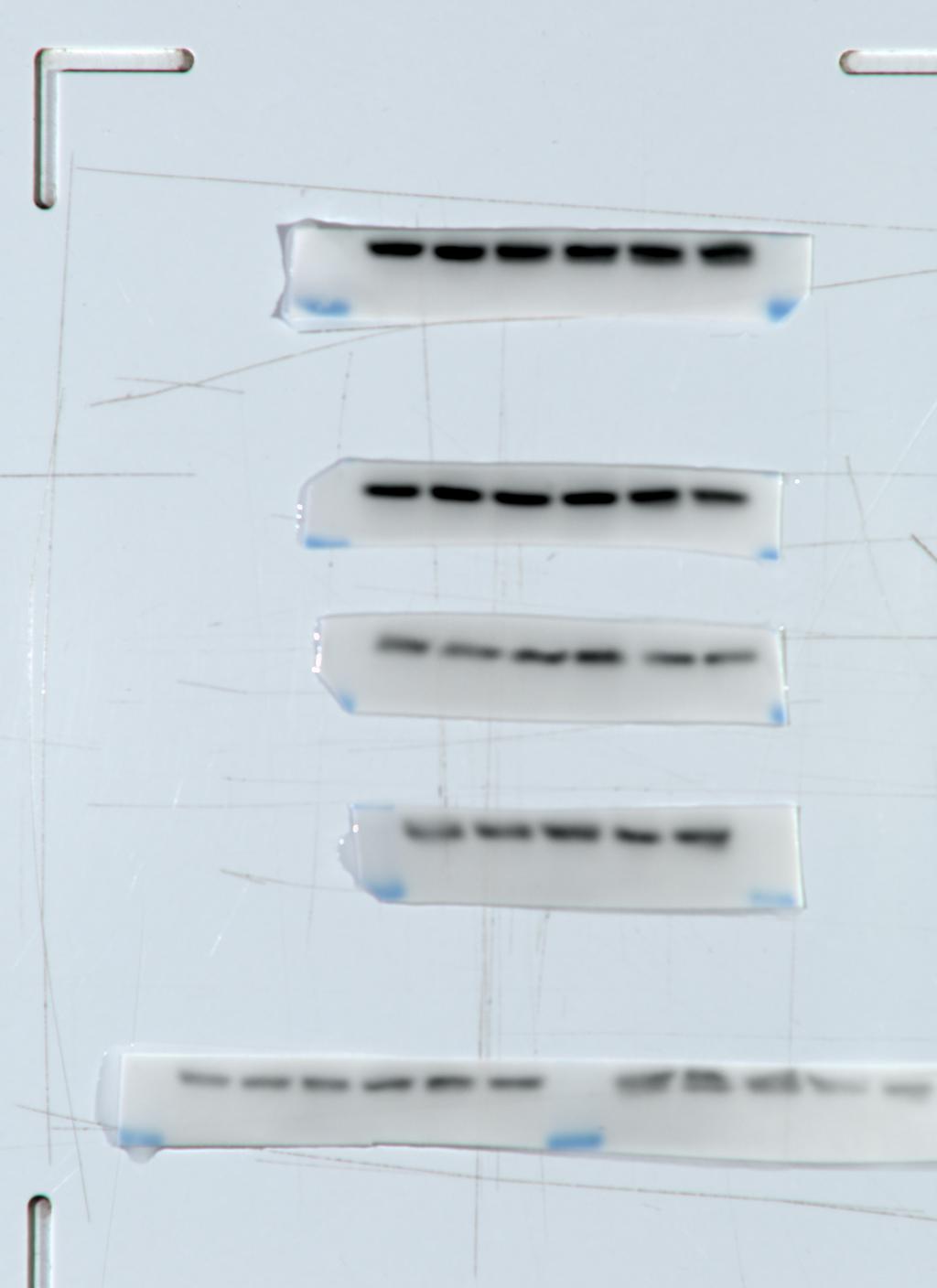

Supplement: Figure 6—figure supplement 1—source data 2. [file elife-76183-fig6-figsupp1-data2.zip › Figure 6-figure supplement 1-source data 2/Figure 6 S1B INPUT-Tubulin.jpg]
