# Supplementary figures and images for: FER-mediated phosphorylation and PIK3R2 recruitment on IRS4 promotes AKT activation and tumorigenesis in ovarian cancer cells (part 3 of 3)
Source: eLife. 2022 May 12;11:e76183. doi: 10.7554/eLife.76183 (PMC9098222; doi:10.7554/eLife.76183)

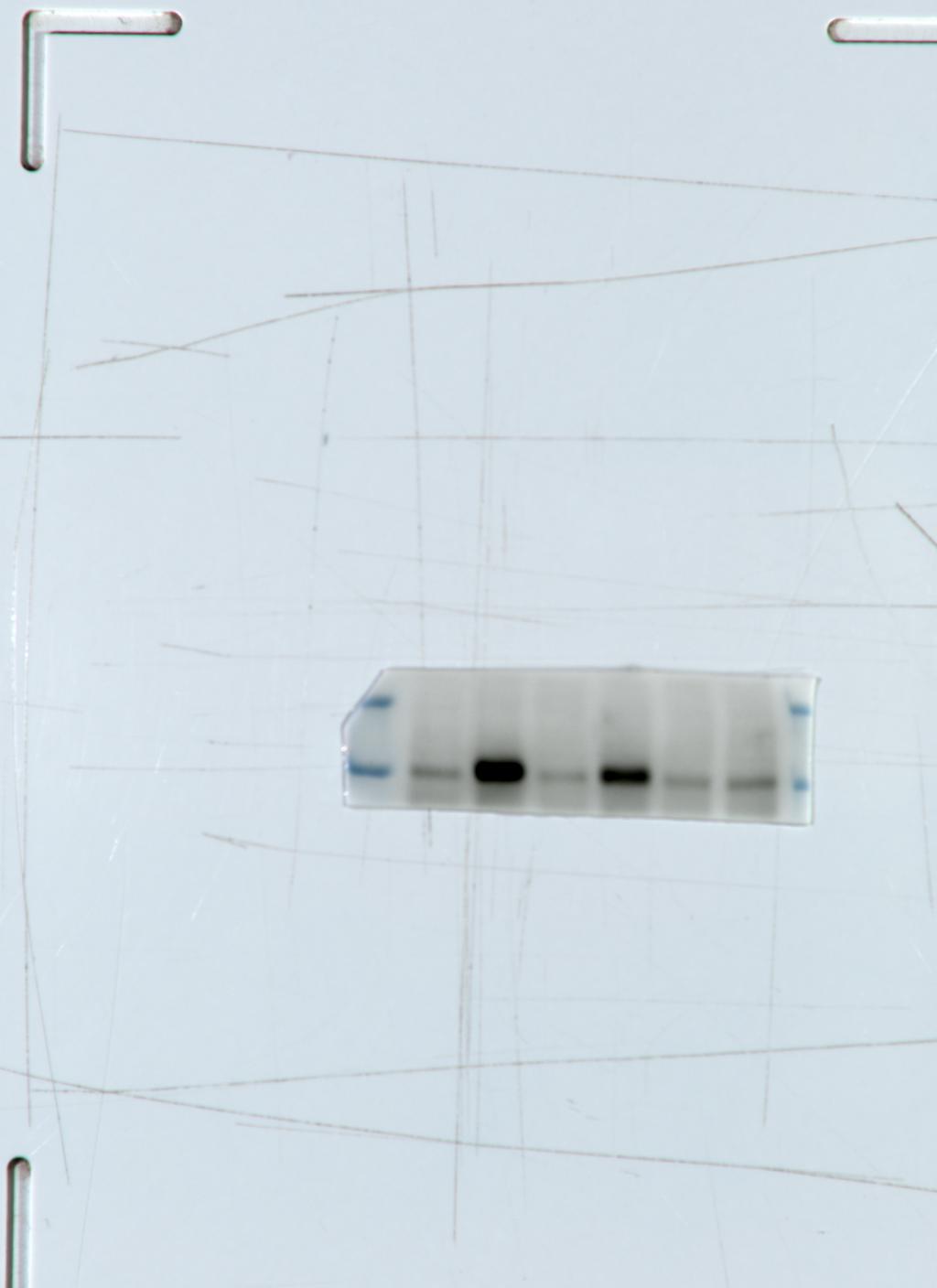

Supplement: Figure 6—figure supplement 1—source data 2. [file elife-76183-fig6-figsupp1-data2.zip › Figure 6-figure supplement 1-source data 2/Figure 6 S1B IP-4G10.jpg]

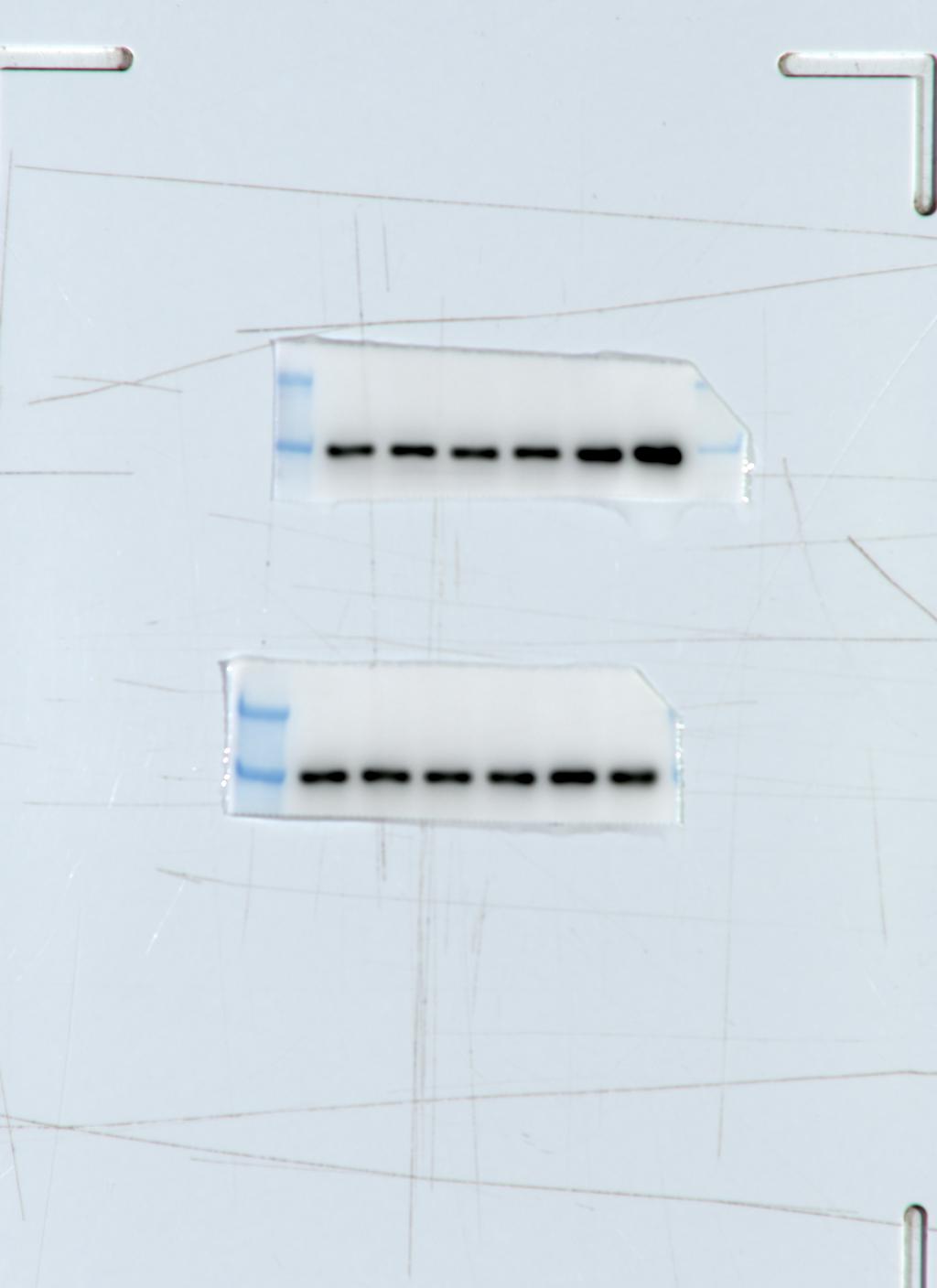

Supplement: Figure 6—figure supplement 1—source data 2. [file elife-76183-fig6-figsupp1-data2.zip › Figure 6-figure supplement 1-source data 2/Figure 6 S1B IP-IRS4.jpg]

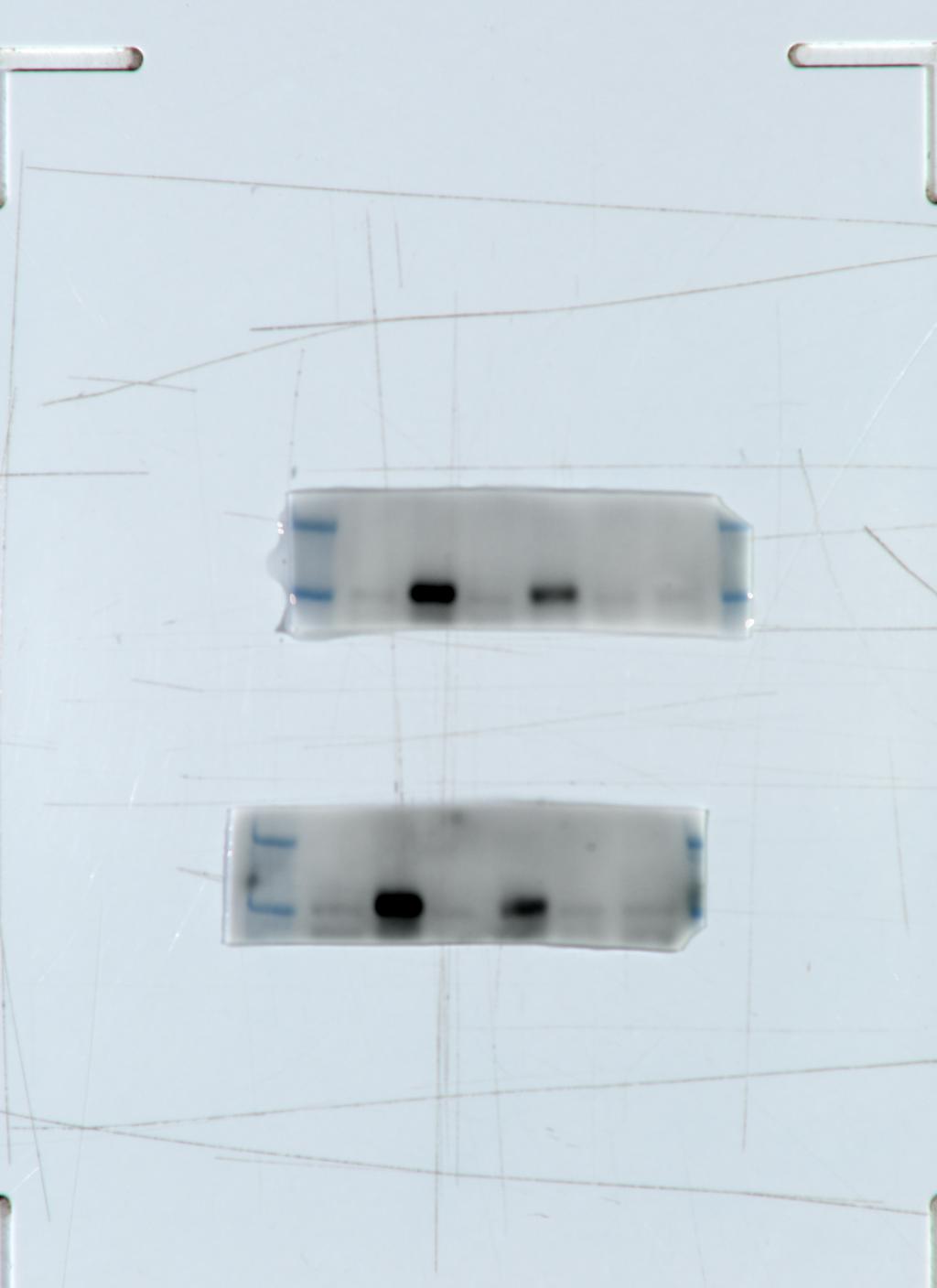

Supplement: Figure 6—figure supplement 1—source data 2. [file elife-76183-fig6-figsupp1-data2.zip › Figure 6-figure supplement 1-source data 2/Figure 6 S1B IP-PY1000.jpg]

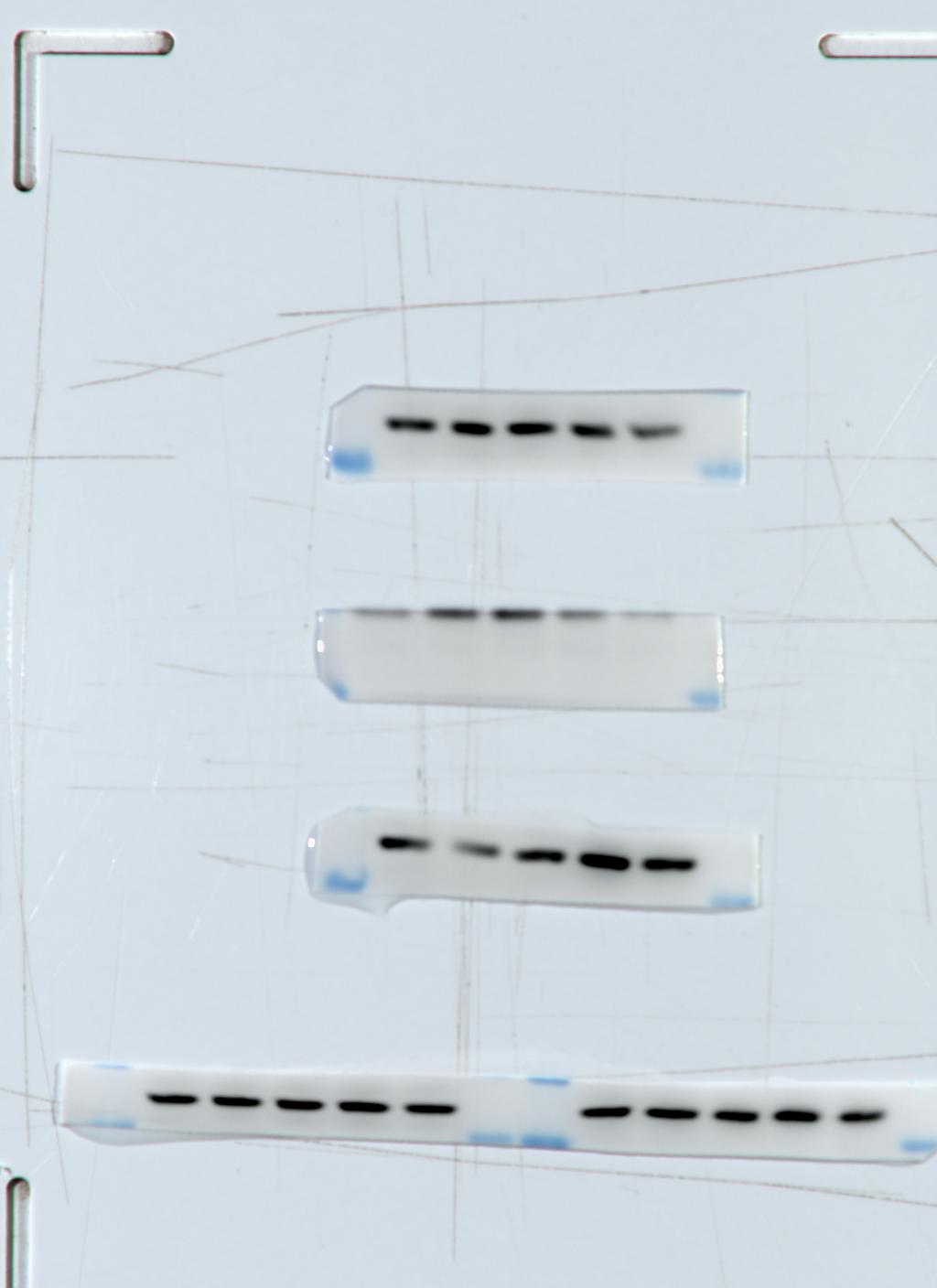

Supplement: Figure 6—figure supplement 1—source data 3. [file elife-76183-fig6-figsupp1-data3.zip › Figure 6-figure supplement 1-source data 3/Figure 6 S1C INPUT-Actin.jpg]

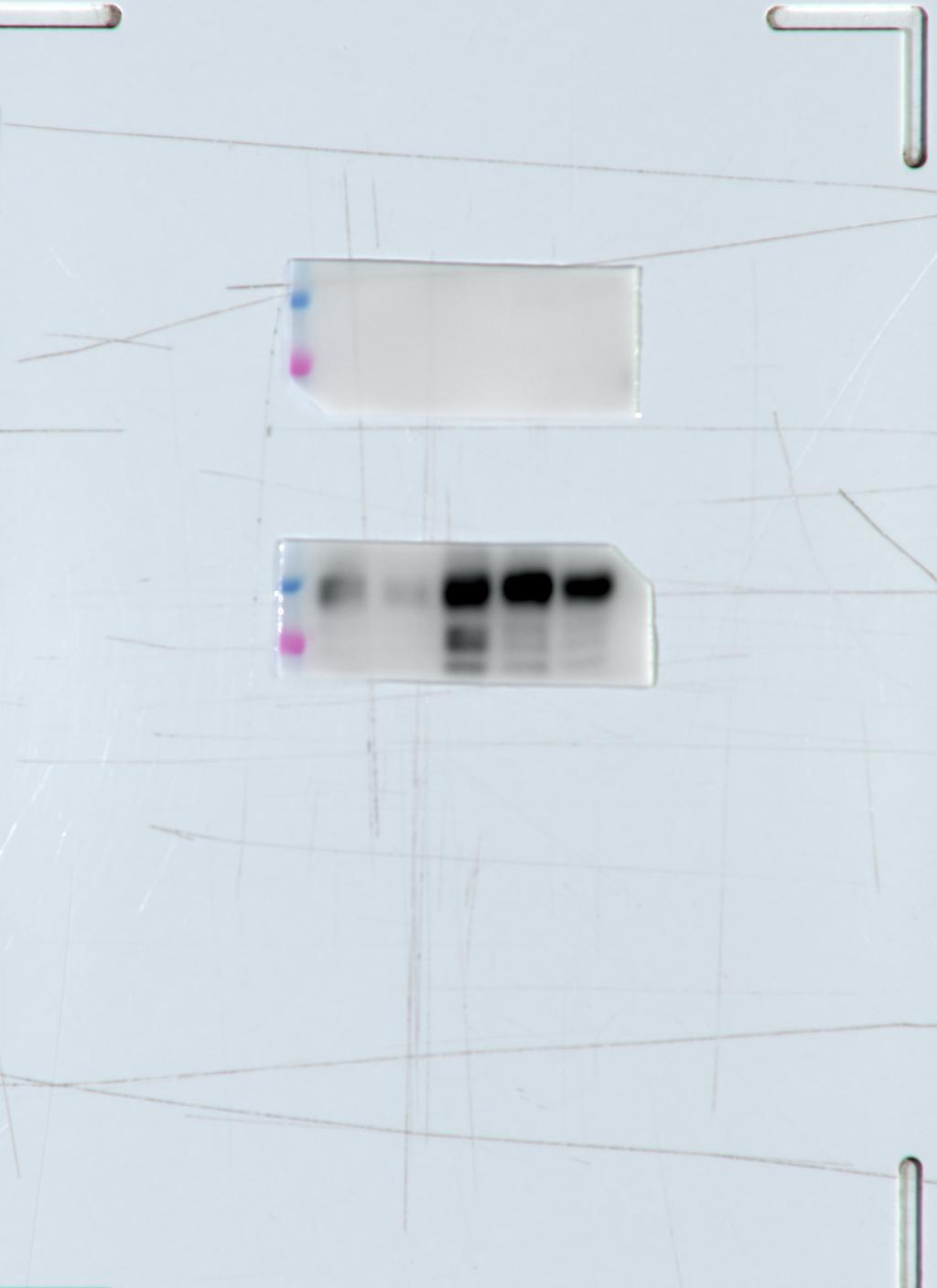

Supplement: Figure 6—figure supplement 1—source data 3. [file elife-76183-fig6-figsupp1-data3.zip › Figure 6-figure supplement 1-source data 3/Figure 6 S1C INPUT-IGF1R.jpg]

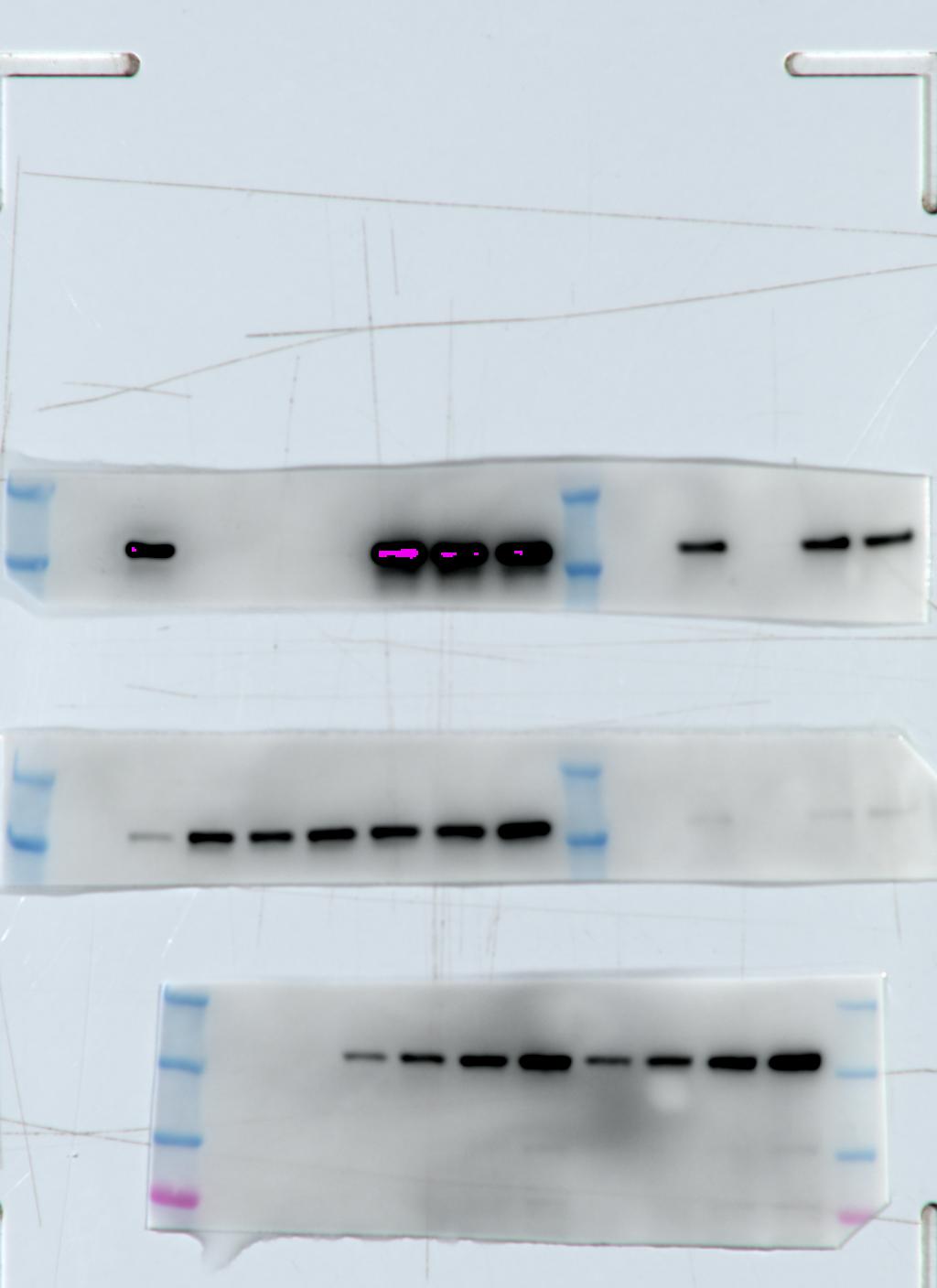

Supplement: Figure 6—figure supplement 1—source data 3. [file elife-76183-fig6-figsupp1-data3.zip › Figure 6-figure supplement 1-source data 3/Figure 6 S1C INPUT-Myc.jpg]

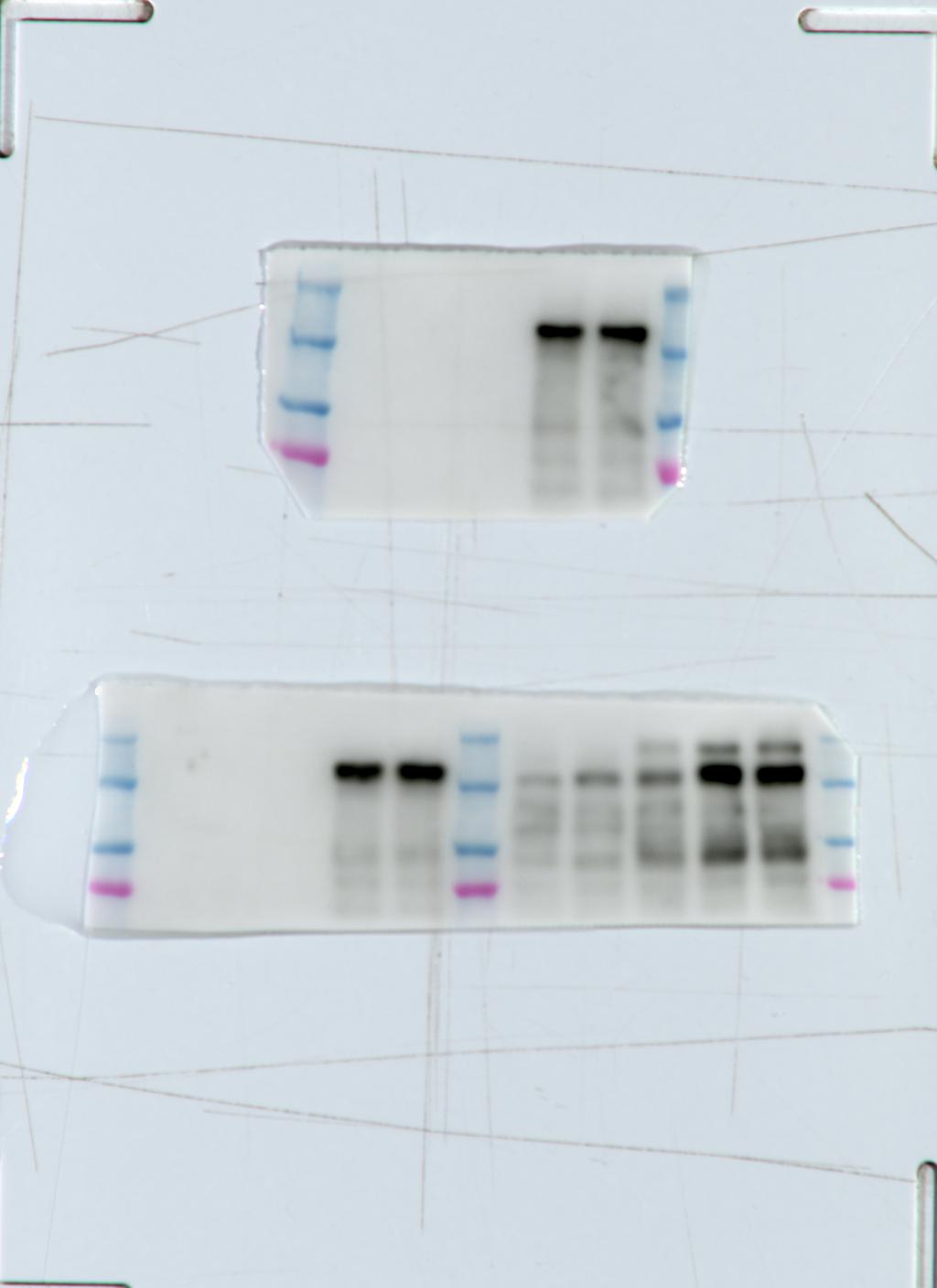

Supplement: Figure 6—figure supplement 1—source data 3. [file elife-76183-fig6-figsupp1-data3.zip › Figure 6-figure supplement 1-source data 3/Figure 6 S1C IP-4G10.jpg]

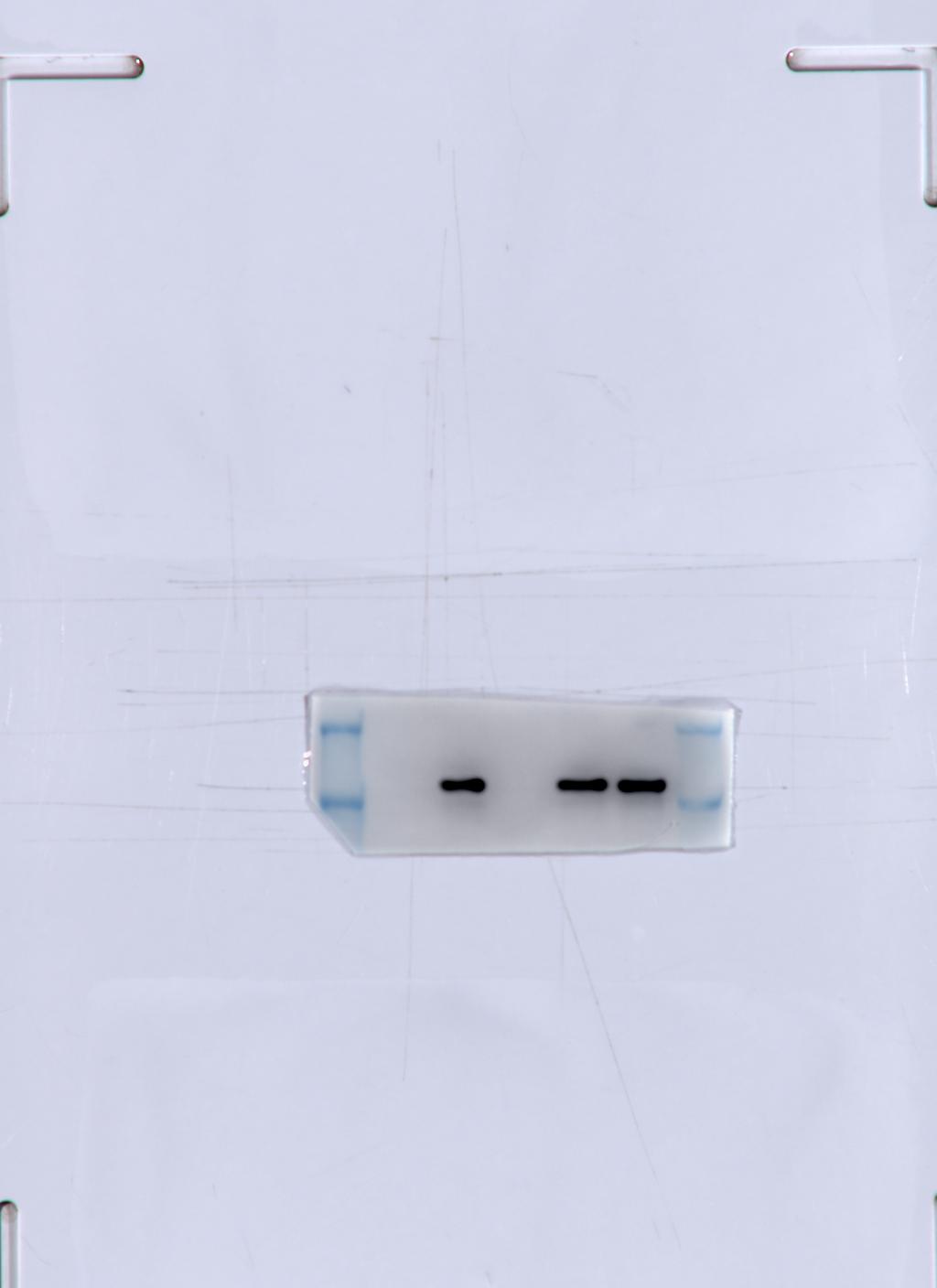

Supplement: Figure 6—figure supplement 1—source data 3. [file elife-76183-fig6-figsupp1-data3.zip › Figure 6-figure supplement 1-source data 3/Figure 6 S1C IP-Myc.jpg]

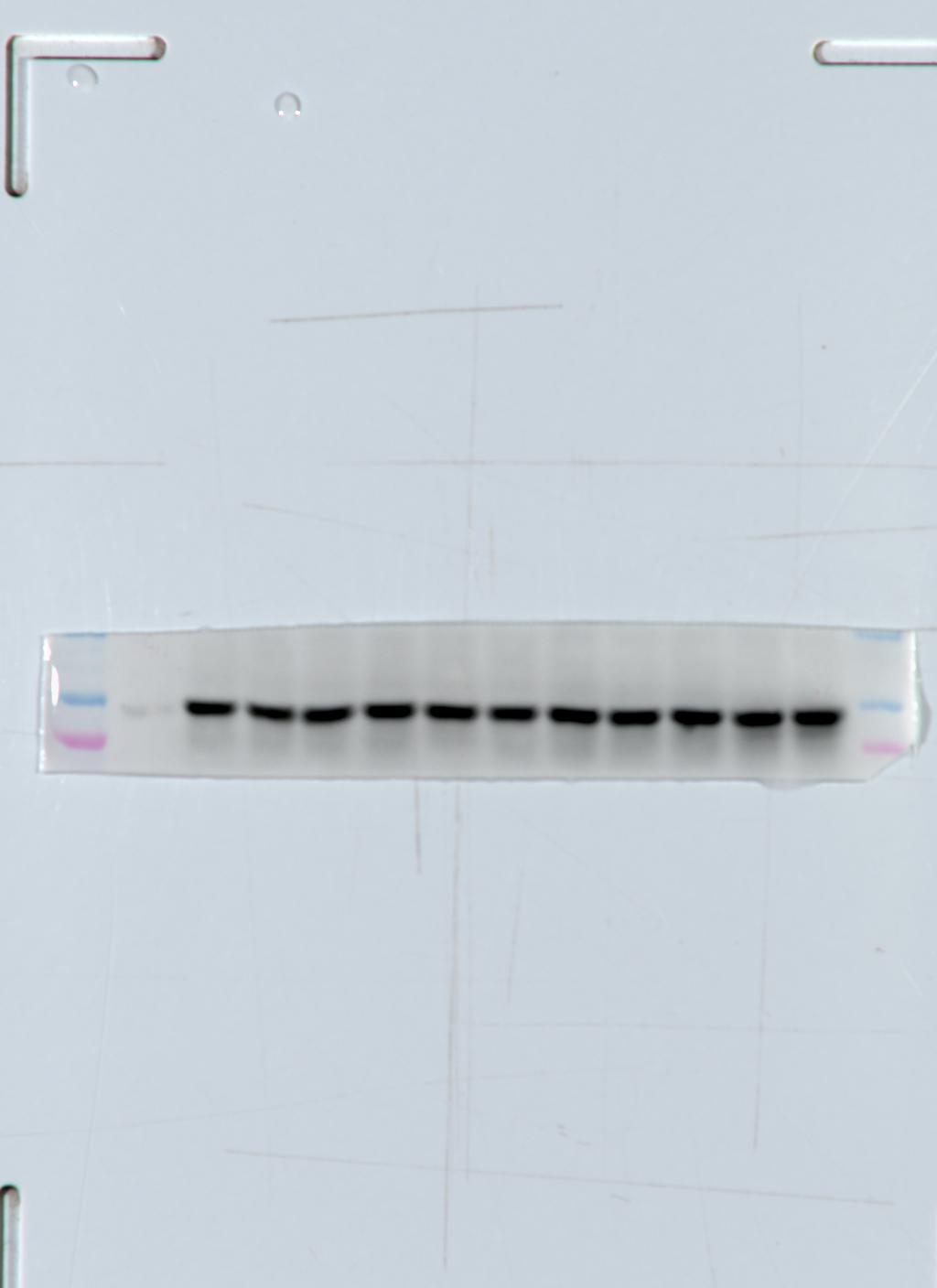

Supplement: Figure 6—figure supplement 2—source data 1. [file elife-76183-fig6-figsupp2-data1.zip › Figure 6-figure supplement 2-source data 1/Figure 6 S2A FER.jpg]

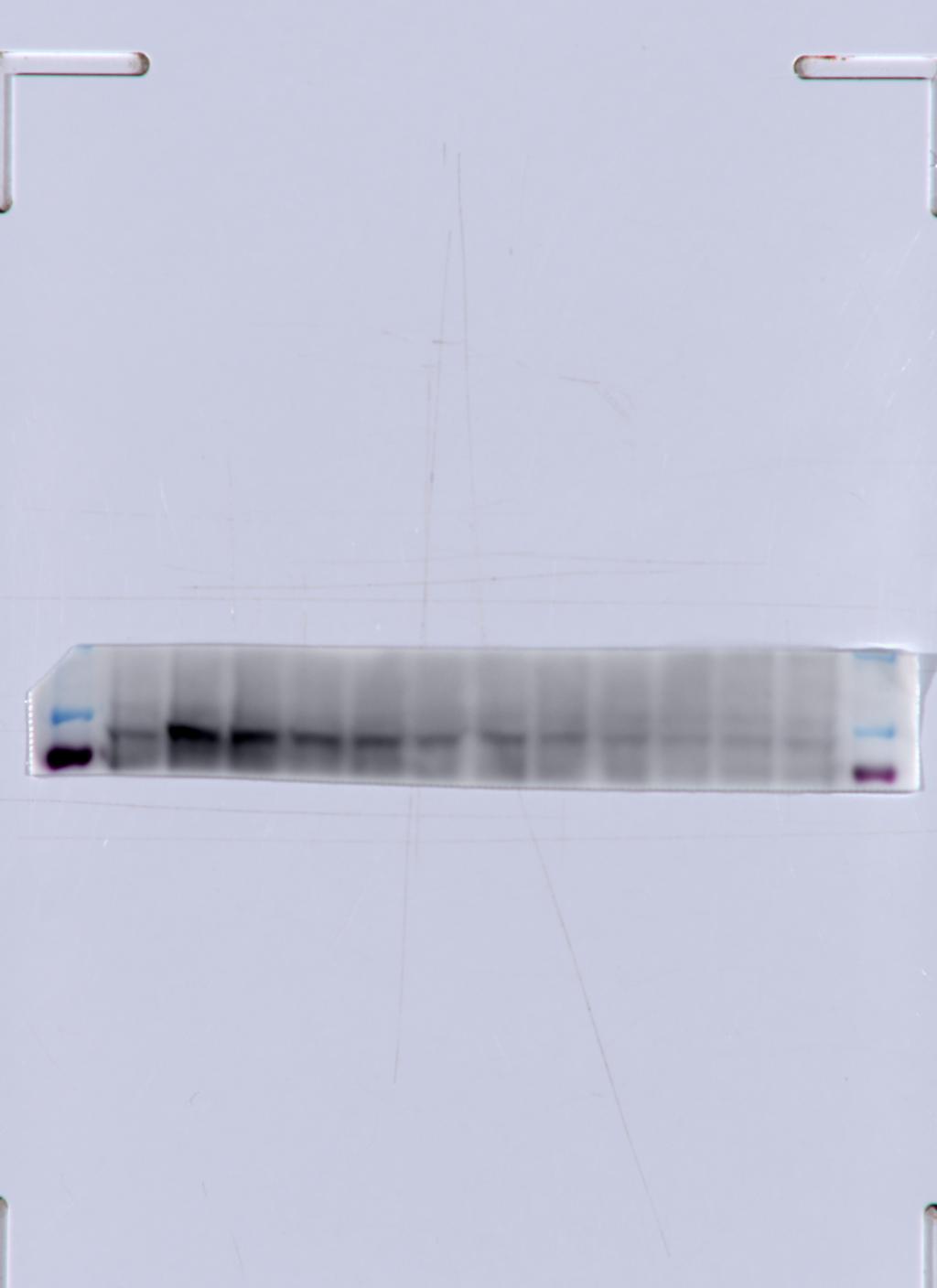

Supplement: Figure 6—figure supplement 2—source data 1. [file elife-76183-fig6-figsupp2-data1.zip › Figure 6-figure supplement 2-source data 1/Figure 6 S2A pY402 FER.jpg]

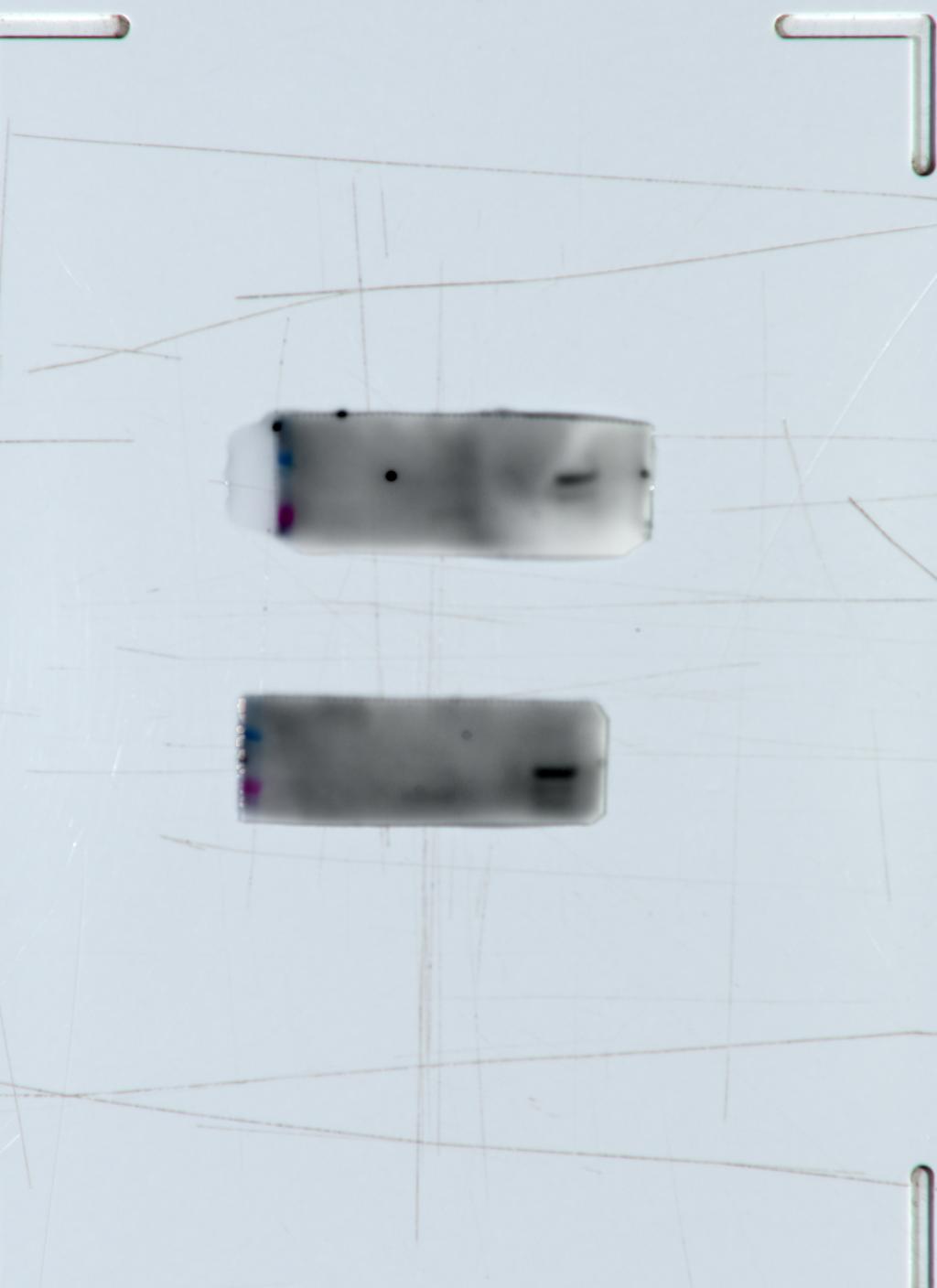

Supplement: Figure 6—figure supplement 2—source data 2. [file elife-76183-fig6-figsupp2-data2.zip › Figure 6-figure supplement 2-source data 2/Figure 6 S2B FES.jpg]

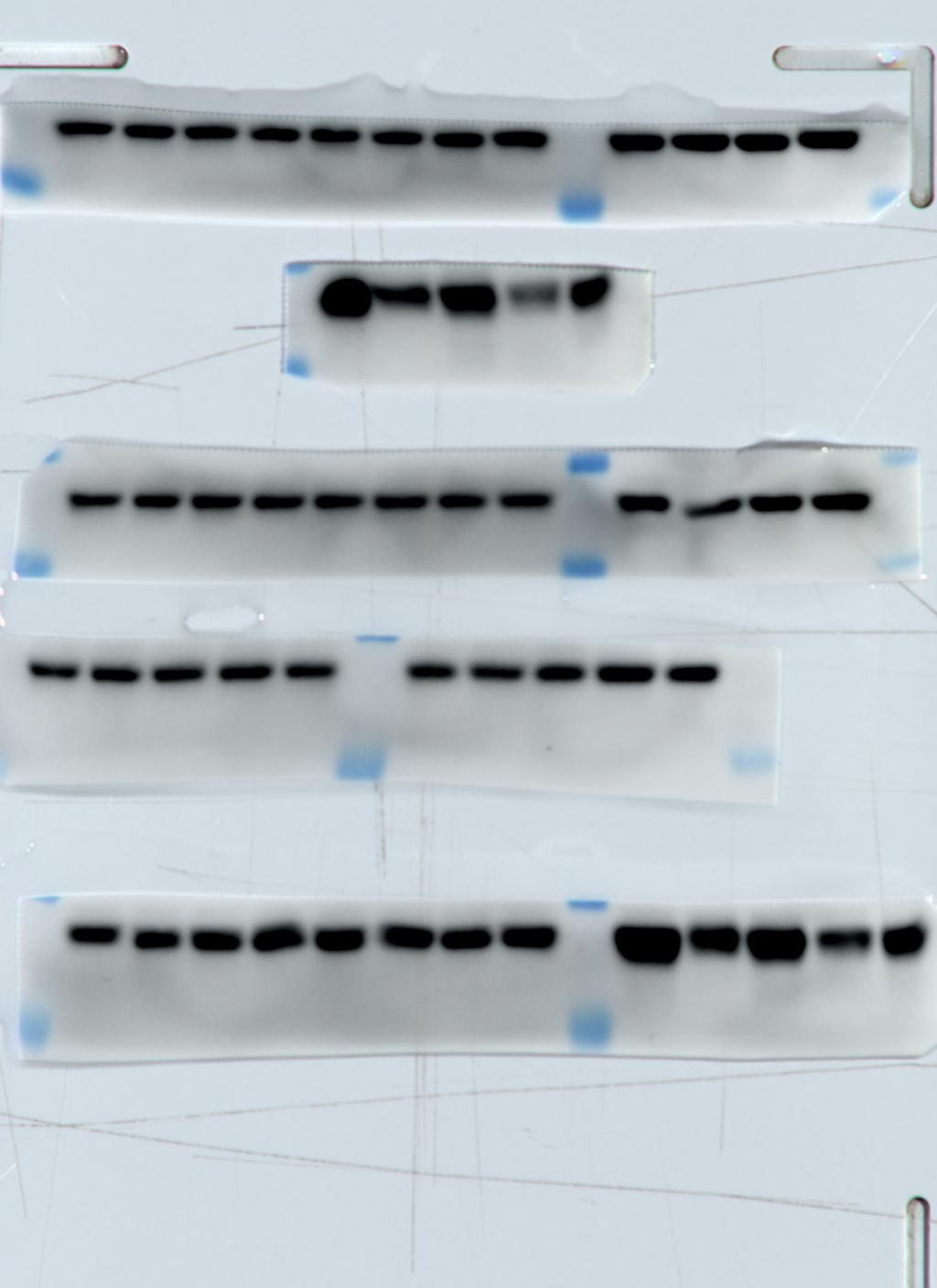

Supplement: Figure 6—figure supplement 2—source data 2. [file elife-76183-fig6-figsupp2-data2.zip › Figure 6-figure supplement 2-source data 2/Figure 6 S2B Tubulin.jpg]
