# Supplementary material for: FER-mediated phosphorylation and PIK3R2 recruitment on IRS4 promotes AKT activation and tumorigenesis in ovarian cancer cells
Source: eLife. 2022 May 12;11:e76183. doi: 10.7554/eLife.76183 (PMC9098222; doi:10.7554/eLife.76183)
Supplement: Transparent reporting form [file elife-76183-transrepform1.doc]

***eLife’s* transparent reporting form**

We encourage authors to provide detailed information *within their submission* to facilitate the interpretation and replication of experiments.Authors can upload supporting documentation to indicate the use of appropriate reporting guidelines for health-related research (see [EQUATOR Network](http://www.equator-network.org/ )), life science research (see the [BioSharing Information Resource](https://biosharing.org/)), or the [ARRIVE guidelines](http://www.plosbiology.org/article/info:doi/10.1371/journal.pbio.1000412) for reporting work involving animal research.Where applicable, authors should refer to any relevant reporting standards documents in this form.

If you have any questions, please consult our Journal Policies and/or contact us:[editorial@elifesciences.org](mailto:editorial@elifesciences.org).

**Sample-size estimation**

- You should state whether an appropriate sample size was computed when the study was being designed
- You should state the statistical method of sample size computation and any required assumptions
- If no explicit power analysis was used, you should describe how you decided what sample (replicate) size (number) to use

Please outline where this information can be found within the submission (e.g., sections or figure legends), or explain why this information doesn’t apply to your submission:

Appropriate sample sizes were computed during the experimental design. No power analysis was used. Sample-size estimation for each experiment could be found within the section of figure legends or within the section of materials and methods.

**Replicates**

- You should report how often each experiment was performed
- You should include a definition of biological versus technical replication
- The data obtained should be provided and sufficient information should be provided to indicate the number of independent biological and/or technical replicates
- If you encountered any outliers, you should describe how these were handled
- Criteria for exclusion/inclusion of data should be clearly stated
- High-throughput sequence data should be uploaded before submission, with a private link for reviewers provided (these are available from both GEO and Array Express)

Please outline where this information can be found within the submission (e.g., sections or figure legends), or explain why this information doesn’t apply to your submission:

No outliers were encountered and excluded. Replicates could be found within the section of figure legends or within the section of materials and methods.

**Statistical reporting**

- Statistical analysis methods should be described and justified
- Raw data should be presented in figures whenever informative to do so (typically when N per group is less than 10)
- For each experiment, you should identify the statistical tests used, exact values of N, definitions of center, methods of multiple test correction, and dispersion and precision measures (e.g., mean, median, SD, SEM, confidence intervals; and, for the major substantive results, a measure of effect size (e.g., Pearson's r, Cohen's d)
- Report exact p-values wherever possible alongside the summary statistics and 95% confidence intervals. These should be reported for all key questions and not only when the p-value is less than 0.05.

Please outline where this information can be found within the submission (e.g., sections or figure legends), or explain why this information doesn’t apply to your submission:

Information on sample-size estimation, number of replicates, data presentation and type of statistical analyses for each experiment could be found within the section of figure legends or within the section of materials and methods. All statistical analyses including standard Student’s T-test or two-way ANOVA multiple comparisons were performed by the GraphPad Prism (v7.00). Data were shown as means ± S.D or SEM. The following indications of significance were used throughout the manuscript and indicated for each experiment in the figure legends: ns = no significance, **p < 0.01, ***p < 0.001, ****p < 0.0001. Exact P-values were presented whenever possible.

(For large datasets, or papers with a very large number of statistical tests, you may upload a single table file with tests, Ns, etc., with reference to sections in the manuscript.)

**Group allocation**

- Indicate how samples were allocated into experimental groups (in the case of clinical studies, please specify allocation to treatment method); if randomization was used, please also state if restricted randomization was applied
- Indicate if masking was used during group allocation, data collection and/or data analysis

Please outline where this information can be found within the submission (e.g., sections or figure legends), or explain why this information doesn’t apply to your submission:

In Figure 6G-I, NSG mice were randomly assigned and divided into three (Figure 6G) or two groups (Figure 6H and 6I). No restricted randomization was applied. Otherwise, we did not conduct specific studies that required allocating and randomizing samples into experimental groups.

**Additional data files (“source data”)**

- We encourage you to upload relevant additional data files, such as numerical data that are represented as a graph in a figure, or as a summary table
- Where provided, these should be in the most useful format, and they can be uploaded as “Source data” files linked to a main figure or table
- Include model definition files including the full list of parameters used
- Include code used for data analysis (e.g., R, MatLab)
- Avoid stating that data files are “available upon request”

Please indicate the figures or tables for which source data files have been provided:

- The raw spectrometry proteomics data have been deposited to the ProteomeXchange Consortium with the dataset identifier PXD022084 (The URL in ProteomeXchange Consortium: http://proteomecentral.proteomexchange.org/cgi/GetDataset? ID=PXD022084. The URL in iProX: https://www.iprox.org/page/project.html? id=IPX0002540000).
- Analyzed MS data was provided in Supplementary File 1-12.
- The original files of the full raw unedited gels or blots as well as figures with the uncropped gels or blots with the relevant bands clearly labelled were provided in Original Files of the Gels and Blots as Source data files.
- Primers were uploaded in Supplementary File 13.
